# Rhodium-catalysed connective synthesis of diverse reactive probes bearing S(VI) electrophilic warheads

**DOI:** 10.3762/bjoc.21.150

**Published:** 2025-09-17

**Authors:** Scott Rice, Julian Chesti, William R T Mosedale, Megan H Wright, Stephen P Marsden, Terry K Smith, Adam Nelson

**Affiliations:** 1 School of Chemistry, University of Leeds, Leeds, LS2 9JT, UK; 2 Astbury Centre for Structural Molecular Biology, University of Leeds, Leeds, LS2 9JT, UK; 3 Schools of Biology and Chemistry, Biomedical Sciences Research Complex, University of St Andrews, St Andrews, KY16 9ST, UK

**Keywords:** covalent probes, molecular diversity, rhodium carbenoids

## Abstract

The value of small molecules that chemically modify proteins is increasingly being recognised and utilised in both chemical biology and drug discovery. The discovery of such chemical tools may be enabled by screening diverse sets of reactive probes. Most existing sets of reactive probes are armed with cysteine-directed warheads, a limitation that we sought to address. A connective synthesis was developed in which α-diazoamide substrates, armed with a S(VI) warhead, were reacted with diverse co-substrates. A high-throughput approach was used to identify promising substrate/co-substrate/catalyst combinations which were then prioritised for purification by mass-directed HPLC to yield a total of thirty reactive probes. The structural diversity of the probe set was increased by the multiplicity of reaction types between rhodium carbenoids and the many different co-substrate classes, and the catalyst-driven selectivity between these pathways. The probes were screened for activity against *Trypanosma brucei*, and four probes with promising anti-trypanosomal activity were identified. Remarkably, the synthetic approach was compatible with building blocks bearing three different S(VI) warheads, enabling the direct connective synthesis of diverse reactive probes armed with non-cysteine-directed warheads. Reactive probes that are synthetically accessible using our approach may be of value in the discovery of small molecule modifiers for investigating and engineering proteins.

## Introduction

Diverse sets of reactive probes can facilitate the discovery of chemical tools and drugs that chemically modify protein targets [1–3]. Established sets of reactive probes are typically armed with electrophilic warheads that have the potential to target nucleophilic amino acid side chains. Most reactive probe sets bear cysteine-directed warheads [3–7], although sets have also been designed to target a wider range of amino acids [8–10]. Sets of reactive probes are generally prepared using robust reactions, most usually amide formation, chosen from the toolkit that currently dominates medicinal chemistry [11] which may, in turn, limit probe structural diversity.

**Figure 1:**
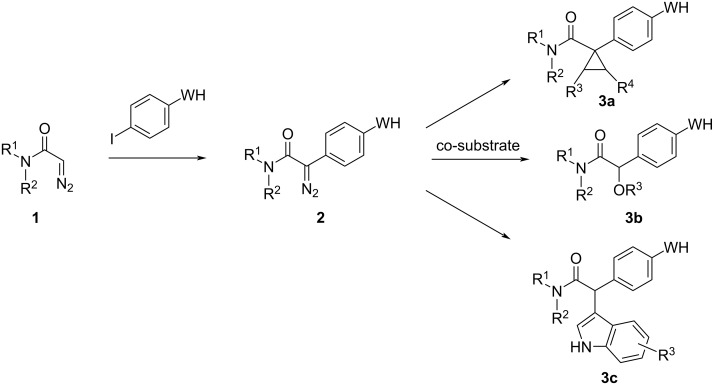
Envisaged connective synthesis of reactive probes **3** bearing S(VI) electrophilic warheads (WH). Diverse probes **3** might be accessible by functionalising α-diazoamide substrates **2** via alternative reaction modes.

We have developed a unified connective approach for the synthesis of structurally diverse reactive probes bearing S(VI) electrophiles. Proteome-wide screens have shown that S(VI) electrophiles predominantly target lysine and tyrosine [12], although other residues (e.g. serine) may also be targeted within enzyme active sites [13]. It was envisaged that the reactive probes would be prepared by dirhodium-catalysed reactions between pairs of building blocks: an α-diazoamide **2** bearing a S(VI) electrophile and a suitable co-substrate (→ **3**) (Figure 1). Here, metal-catalysed carbenoid chemistry was chosen because of the wide range of potentially reactive functional groups that might be incorporated into co-substrates [14]. The richness of potential connective chemistry, and the availability of alternative dirhodium catalysts with distinctive reactivity, was expected to expand the structural diversity of accessible reactive probes. Herein, we describe the successful execution of this approach and the demonstration of biological function of the resulting reactive probes.

## Results and Discussion

**Scheme 1:**
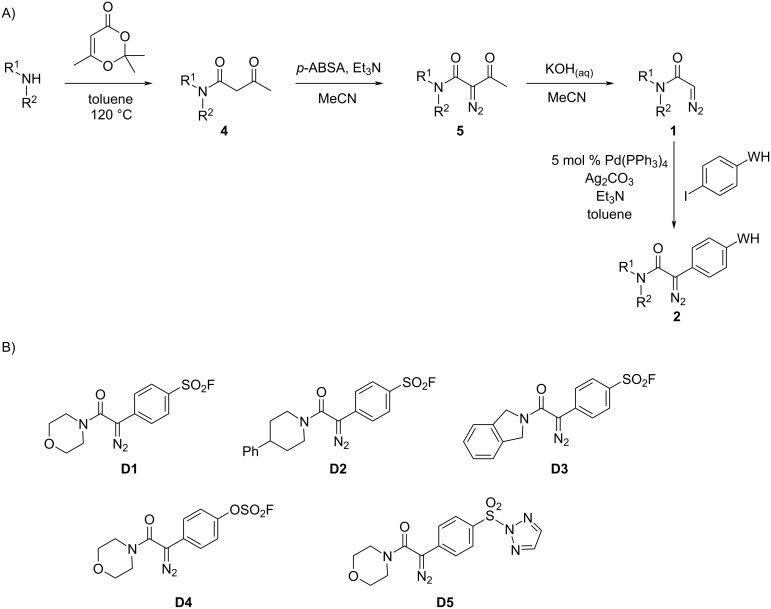
Synthesis of α-diazoamide substrates **D1**–**5** of general structure **2** bearing S(VI) electrophiles. Panel A: Overview of synthesis (see Table 1 for details of synthesis of individual substrates). Panel B: Substrates that were prepared.

We prepared five α-diazoamide substrates bearing S(VI) electrophiles (Scheme 1 and Table 1) [15]. Initially, three amines – morpholine, 4-phenylpiperidine and isoindoline – were reacted with 2,2,6-trimethyl-4*H*-1,3-dioxin-4-one to give the corresponding β-ketoamides **4**. Treatment of the β-ketoamides **4** with 4-acetamidobenzenesulfonyl azide (*p*-ABSA) and triethylamine gave the α-diazo-β-ketoamides **5**. Subsequent KOH-mediated deacetylation yielded the corresponding α-diazoamides **1**. Finally, Pd-catalysed cross-coupling with warhead-substituted phenyl iodides gave, in low to moderate yield, the required α-diazoamide substrates **2** (referred to individually as **D1**–**5** below). Whilst the Pd-catalysed arylation of α-diazoamides and esters is known [15–19], its tolerance of pendant S(VI) electrophiles has not been previously explored and is notable.

**Table 1:** Synthesis of α-diazoamide substrates of general structure **2** bearing S(VI) electrophiles (see Scheme 1).

Amine	Yield **4** (%)	Yield **5** (%)	Yield **1** (%)	WH	Substrate (yield, %)

morpholine	94	80	55	–SO_2_F	**D1** (46)
				–OSO_2_F	**D4** (26)
				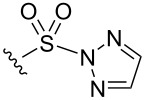	**D5** (23)
4-phenylpiperidine	85	82	87	–SO_2_F	**D2** (53)
isoindoline	88	86	99	–SO_2_F	**D3** (12)

**Figure 2:**
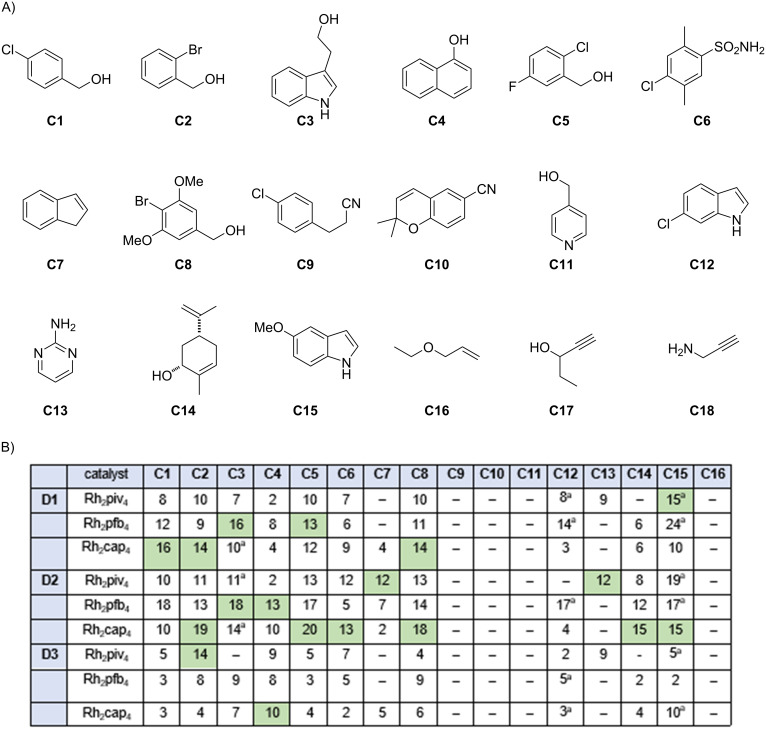
Structures and reactions of co-substrates. Panel A: structures of the 16 selected co-substrates **C1**–**16**, together with two additional co-substrates **C17** and **C18** that were subsequently used. Panel B: yields, estimated by evaporative light scattering detection, of reactions involving combinations of substrates, co-substrates and catalysts (dash: <2% estimated yield). Highlighted combinations (green boxes) were selected for mass-directed purification. ^a^Multiple intermolecular products observed by analytical HPLC.

Due to the relatively large size of the diazo substrates **D1**–**5**, it was decided to design a set of diverse co-substrates with 15 or fewer heavy (non-hydrogen) atoms. It was decided that the set should include co-substrates with the potential to react with metal carbenoids in many different ways [14], for example through O–H, N–H or formal C–H insertion, cyclopropanation, or oxazole [20] formation. The 16 co-substrates, selected from available compounds in our laboratory, are shown in Figure 2 (panel A). Many of these substrates had more than one potentially reactive site to enable, for example, O–H insertion (**C1**–**5**, **C8**, **C11** and **C14**), N–H insertion (**C3**, **C6**, **C12**, **C13** and **C15**), formal C–H insertion (**C1**, **C3, C4**, **C12**, **C15** and **C16**), oxazole formation (**C9** and **C10**) and cyclopropanation (**C7**, **C10**, **C14** and **C16**).

To start with, we investigated reactions of the α-diazoamide substrates **D1**, **D2** and **D3** with the 16 co-substrates **C1**–**16** catalysed by three diverse [21] dirhodium catalysts (Rh_2_piv_4_, Rh_2_pfb_4_ and Rh_2_cap_4_) i.e., an array of 144 reactions. An α-diazoamide substrate (20 μmol; 16 μL of a 1.25 M solution in CH_2_Cl_2_) and a co-substrate (5 equiv; 16 μL of a 6.25 M solution in CH_2_Cl_2_) were added to glass vials in a 96-well reaction block, and the solvent left to evaporate after each addition. Subsequently, a dirhodium catalyst (1 mol %; 200 μL of a 1 mM solution in CH_2_Cl_2_) was also added to each vial. The final volume of each reaction was thus 200 μL, with final concentrations of 100 mM (for substrates), 500 mM (for co-substrates) and 1 mM (for catalysts).

**Figure 3:**
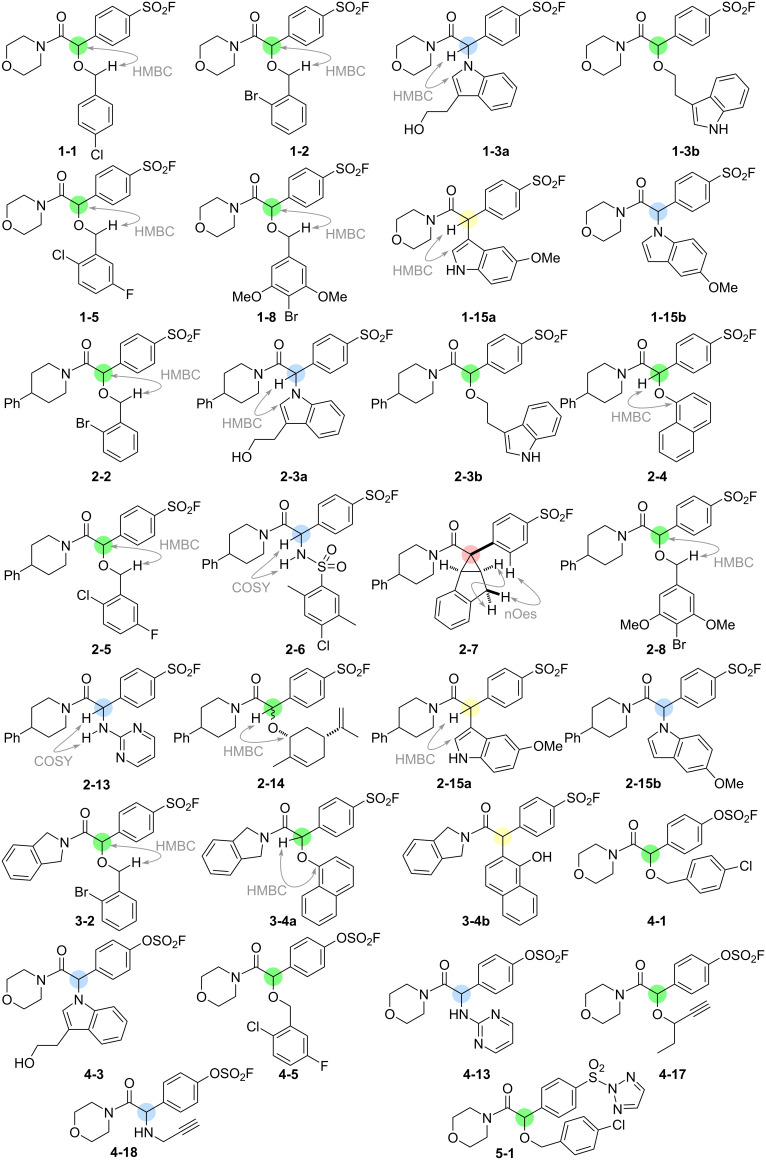
Structures and structure elucidation of intermolecular reaction products. The relevant reactivity modes are indicated by colour: O–H insertion (green); N–H insertion (blue); formal C–H insertion (yellow); and cyclopropanation (pink).

After 48 h, the outcome of the reactions was determined by analytical UPLC–MS with, additionally, evaporative light-scattering detection [22–23] to enable estimation of the yield of each product (Figure 2, panel B). It was found that many reactions involving alcohol- (e.g., **C1**–**5**, **C8**, **C11** and **C14**) and indole- (e.g., **C3**, **C12** and **C15**) containing co-substrates yielded intermolecular products, whilst those involving nitrile-containing co-substrates (**C9** and **10**) and the allylic ether **C16** did not. It is remarkable that S(VI) electrophiles are tolerated. Eighteen substrate/co-substrate combinations gave, with at least one of the catalysts, an intermolecular product in >10% estimated yield (typically corresponding to >1 mg product). For all but one of these reactions, a product with molecular weight consistent with O–H insertion into water was also observed. For these 18 substrate/co-substrate combinations, the reaction with the highest estimated yield was selected for mass-directed purification (Table 2). In total, 23 intermolecular reaction products were isolated and structurally characterised (using, where appropriate, HMBC, COSY and nOe NMR methods; see Figure 3). In general, the yields of these products were rather low, which may stem from poor (co-)substrate solubility in some cases; and/or competitive O–H insertion into adventitious water.

**Table 2:** Outcomes of reactions between α-diazoamide substrates and co-substrates.

Diazo	Co-substrate	Catalyst	Product^a^	Yield^b^

**D1**	**C1**	Rh_2_cap_4_	**1-1**	14
**D1**	**C2**	Rh_2_cap_4_	**1-2**	12
**D1**	**C3**	Rh_2_pfb_4_	**1-3a** **1-3b**	15 1
**D1**	**C5**	Rh_2_pfb_4_	**1-5**	11
**D1**	**C8**	Rh_2_cap_4_	**1-8**	12
**D1**	**C15**	Rh_2_piv_4_	**1-15a** **1-15b**	6 8
**D2**	**C2**	Rh_2_cap_4_	**2-2**	14
**D2**	**C3**	Rh_2_pfb_4_	**2-3a** **2-3b**	13 1
**D2**	**C4**	Rh_2_pfb_4_	**2-4**	11
**D2**	**C5**	Rh_2_cap_4_	**2-5**	14
**D2**	**C6**	Rh_2_cap_4_	**2-6**	10
**D2**	**C7**	Rh_2_piv_4_	**2-7**	13^c^
**D2**	**C8**	Rh_2_cap_4_	**2-8**	13
**D2**	**C13**	Rh_2_piv_4_	**2-13**	12
**D2**	**C14**	Rh_2_cap_4_	**2-14**	10^d^
**D2**	**C15**	Rh_2_cap_4_	**2-15a** **2-15b**	11 1
**D3**	**C2**	Rh_2_piv_4_	**3-2**	13
**D3**	**C4**	Rh_2_cap_4_	**3-4a** **3-4b**	5^e^ 5^e^
**D4**	**C1**	Rh_2_pfb_4_	**4-1**	56
**D4**	**C3**	Rh_2_pfb_4_	**4-3**	23
**D4**	**C5**	Rh_2_cap_4_	**4-5**	8
**D4**	**C13**	Rh_2_piv_4_	**4-13**	35
**D4**	**C17**	Rh_2_pfb_4_	**4-17**	11
**D4**	**C18**	Rh_2_pfb_4_	**4-18**	23
**D5**	**C1**	Rh_2_pfb_4_	**5-1**	26

^a^Reactions were performed in glass vials with an α-diazoamide substrate (20 μmol; limiting reactant), a co-substrate (5 equiv) and 1 mol % dirhodium catalyst. ^b^Isolated yield of purified product. ^c^dr: >95:<5. ^d^dr: 51:49. ^e^Obtained as a 50:50 mixture of inseparable products.

On the basis of these results, additional reactions involving the α-diazoamide substrates **D4** (with a fluorosulfate warhead) and **D5** (with a sulfonyltriazole warhead) were also executed. In addition to using these two α-diazoamide substrates with different warheads, two additional co-substrates bearing an alkyne tag (**C17** and **C18**) were used. The reactions were assembled from stock solutions, with some variation in stock concentrations to improve solubility. After 24 h, the reaction products were analysed by LC–MS, and promising reactions selected for mass-directed purification. Seven additional intermolecular products were obtained (see Figure 3 and Table 2). The marked improvement in product yields, compared to those observed with **D1**–**3**, may reflect the change to the workflow, i.e., variation in stock concentration to improve solubility.

The diversity of the obtained products was increased by the multiple reaction modes of dirhodium carbenoids that were possible [14]. Overall, products were formed via O–H insertion into an alcohol (to give 14 products) or phenol (→ **2-4** and **3-4a**); N–H insertion into an indole (→ **1-3a**, **1-15b**, **2-3a**, **2-15b** and **4-3**), sulfonamide (→ **2-6**), aminopyrimidine (→ **2-13** and **4-13**) or amine **(**→ **4-18**); cyclopropanation (→ **2-7**); and formal C–H insertion into an indole (→ **1-15a** and **2-15a**) or naphthol (→ **2-4** and **3-4b**). In the case of **4** (2-naphthol) and **15** (5-methoxyindole), co-substrates containing functional groups with more than one potentially reactive site, two regioisomeric products were obtained. In the case of co-substrate **3**, which contains both an indole and an alcohol, thus raising chemoselectivity issues, products were observed from both O–H and N–H insertion. It is notable, however, that despite many of the co-substrates having multiple potentially reactive sites, one intermolecular reaction was generally dominant.

We have previously discovered sulfonyl fluoride probes with promising activity against *T. brucei,* a parasitic kinetoplastid that causes vector-borne African trypanosomiasis (sleeping sickness) [24]. We therefore screened the 23 sulfonyl fluoride probes (derived from diazo compounds **1**, **2** and **3**) against *T. brucei* in 96-well plate format (final concentrations: ≈2–50 μM). Four sulfonyl fluorides were found to have promising activity: **2-5** (EC_50_: 9.38 ± 0.06 μM); **2-6** (EC_50_: 6.81 ± 0.07 μM); **2-14** (EC_50_: 9.26 ± 0.06 μM) and **2-15a** (EC_50_: 11.9 ± 0.2 μM). It is notable that all of these active compounds are 4-phenylpiperidinyl amides derived from the same α-diazoamide **2**, suggesting that this feature is important for activity.

## Conclusion

We have developed a connective synthesis of reactive probes bearing S(VI) electrophilic warheads. Each probe was prepared by rhodium-catalysed reaction between an α-diazo amide substrate bearing a warhead, and a co-substrate. The structural diversity of the probe set was increased by the multiple possible reaction modes of rhodium carbenoids, which enabled many different co-substrate classes and catalyst-driven selectivities to be exploited. A high-throughput synthetic approach was harnessed to identify substrate/co-substrate/catalyst combinations, which led to the productive formation of intermolecular reaction products. Overall, the approach enabled the synthesis of thirty diverse reactive probes. The probes were screened for activity against *T. brucei,* a parasitic kinetoplastid that causes vector-borne African trypanosomiasis, and four probes with promising anti-trypanosomal activity were identified. Remarkably, the synthetic approach was compatible with building blocks bearing three different S(VI) warheads, and enabled the direct connective synthesis of diverse reactive probes. We envisage that such probes may enable chemical modification of non-cysteine residues within proteins, and may be valuable in investigating and engineering the biology of proteins.

## Supporting Information

File 1 Experimental part and NMR spectra of synthesised compounds.

## Data Availability

All data that supports the findings of this study is available in the published article and/or the supporting information of this article.
