# Supplementary material for: Rhodium-catalysed connective synthesis of diverse reactive probes bearing S(VI) electrophilic warheads
Source: Beilstein J Org Chem. 2025 Sep 17;21:1924–31. doi: 10.3762/bjoc.21.150 (PMC12456075; doi:10.3762/bjoc.21.150)
Supplement: File 1 — Experimental part and NMR spectra of synthesised compounds. [file Beilstein_J_Org_Chem-21-1924-s001.pdf]

BEILSTEIN JOURNAL OF ORGANIC CHEMISTRY

Supporting Information

for

Rhodium-catalysed connective synthesis of diverse reactive probes bearing S(VI) electrophilic warheads

Scott Rice, Julian Chesti, William R. T. Mosedale, Megan H. Wright, Stephen P. Marsden, Terry K. Smith and Adam Nelson

Beilstein J. Org. Chem. **2025,** *21,* 1924–1931. doi:10.3762/bjoc.21.150

Experimental part and NMR spectra of synthesised compounds

License and Terms: This is a supporting information file under the terms of the Creative Commons Attribution License (https://creativecommons.org/licenses/by/4.0). Please note that the reuse, redistribution and reproduction in particular requires that the author(s) and source are credited and that individual graphics may be subject to special legal provisions.
The license is subject to the *Beilstein Journal of Organic Chemistry* terms and conditions: (https://www.beilstein-journals.org/bjoc/terms)

General experimental

Commercially available starting materials were obtained from Sigma−Aldrich, Fluorochem and Alfa Aesar. All non-aqueous reactions were performed under nitrogen atmosphere unless otherwise stated. Water-sensitive reactions were performed in anhydrous solvents in oven-dried glassware cooled under nitrogen before use. Anhydrous dichloromethane (DCM), anhydrous tetrahydrofuran (THF), anhydrous toluene, anhydrous diethyl ether, anhydrous ethanol, anhydrous methanol and anhydrous acetonitrile were obtained from a PureSolv MD5 Purification System. All other solvents used were of chromatography or analytical grade. An IKA RV 10 rotary evaporator was used to remove the solvents under reduced pressure.

Thin-layer chromatography (TLC) was performed using aluminium backed silica (Merck silica gel 60 F254) plates obtained from Merck. Ultraviolet lamp (λ_{max} = 254 nm) and $KMnO_4$ were used for visualisation. Flash column chromatography was performed using silica gel 60 (35–70 µm particles) supplied by Merck.

Preparative HPLC was performed using a Water (2767) instrument with a Water SQ detector 2. The system used an XBridge C18 19.0 × 100 mm 5 micron OBD column. The general preparation method used a solvent system of MeCN/H_2O (5–95%) + 0.1% formic acid.

A Bruker MaXis Impact spectrometer with electrospray (ES) ionisation source was used for high-resolution mass spectrometry (HRMS). A Bruker Alpha-P ATR FR-IR spectrometer was used to analyse the infrared spectra.

Proton (^{1}H) and carbon (^{13}C) NMR data was collected on a Bruker 300 (AV3 NMR spectrometer operating at 7.05 T and equipped with a 5 mm BBO probe), 400 (AV3HD NMR spectrometer operating at 9.4 T and equipped with a 5 mm BBO probe) and 500 (AV-NEO NMR spectrometer operating at 11.7 T and equipped with a 5 mm DCH cryoprobe) MHz spectrometer. Fluorine (^{19}F) NMR data was collected on a Bruker 500 (AV-NEO NMR spectrometer operating at 11.7 T and equipped with 5 mm TBO (^{1}H/^{19}F/BB) and TXI (^{1}H/^{13}C/^{15}N) probes) MHz spectrometer. Data was collected at 298 K unless otherwise stated. Chemical shifts (δ) are given in parts per million (ppm) and they are referenced to the residual solvent peak. Coupling constants (*J*) are reported in hertz (Hz) and splitting patterns are reported in an abbreviated manner: app. (apparent), s (singlet), d (doublet), t (triplet), q (quartet), pent (pentet), sept (septet), m (multiplet), br (broad). Assignments were made using COSY, DEPT, HSQC, HMBC and NOESY experiments.

General procedure A: implementation of reaction arrays

By modification of an existing procedure,[1a] the reaction arrays were carried out in a 96-well plate (8 × 12) custom made out of PTFE in borosilicate glass vials (vial volume = 750 µL, vial dimensions = 8 × 30 mm, CV-2100-0830 Chemglass). Diazo substrates were typically dissolved in DCM to give 1.25 M stock solutions. Co-substrates were dissolved in DCM to give 6.25 M stock solutions. Stock solutions of the catalysts were then prepared that were 1.00 mM in DCM. Then, 16 µL of the appropriate diazo substrate stock solution was added to the appropriate wells and the solvent was evaporated. Then, 16 µL of the appropriate co-substrate stock solution was added to the appropriate wells and evaporated. This was followed by the addition of 200 µL of the catalyst stock solution. Lastly, each of the reaction wells in the plate was capped. The final volume of the reaction mixture was 200 µL; with final concentrations of catalyst (1 mM), substrate (100 mM) and co-substrate (500 mM). The wells were left to react at rt, without any stirring, for 48 h and the crude mixtures were concentrated under reduced pressure overnight to remove any residual DCM. The wells were re-dissolved in 200 µL of DMSO to give a total product concentration of 100 mM and transferred to a 96-well plate ready for subsequent analysis[1b,1c] and purification.

4-Iodobenzenesulfonyl fluoride

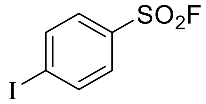

4-Iodobenzenesulfonyl chloride (1.00 g, 3.33 mmol) was dissolved in MeCN (3.33 mL) then a solution of KHF_2 (515 mg, 6.66 mmol) in water (1.67 mL) was added and the resulting biphasic mixture stirred at rt for 18 h. The phases were separated and the aqueous phase was then extracted with EtOAc (3 × 30 mL). The organic phases were combined, washed with sat. aq. $NaHCO_3$ (20 mL) and brine (20 mL), dried ($MgSO_4$), filtered and concentrated under reduced pressure to give sulfonyl fluoride**2** (721 mg, 76%) as a white solid. R_f 0.50 (EtOAc–hexane 10:90). δ_H (500 MHz, $CDCl_3$): 8.01 (2H, d, *J* 8.4 Hz, 3-H and 5-H), 7.71 (2H, d, *J* 8.4 Hz, 2-H and 6-H); δ_C (125 MHz, $CDCl_3$): 139.2 (C_2-3,5), 132.8 (d, *J* 25.6 Hz, C-1), 129.6 (C_2-2,6), 104.2 (C-4); δ_F (470 MHz, $CDCl_3$): 66.2 (SO_2F). All data is consistent with known literature values.[2]

2-((4-Iodophenyl)sulfonyl)-2*H*-1,2,3-triazole

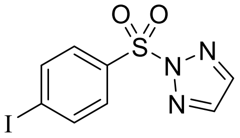

4-Iodobenzenesulfonyl chloride (302 mg, 1 mmol) was dissolved in CH_2Cl_2 (5.0 mL) and the solution was cooled in an ice bath. 1*H*-1,2,3-Triazole (175 µL, 3 mmol) was then added dropwise to the solution followed by *N*,*N*-diisopropylethylamine (347 µL, 2 mmol). The reaction was allowed to stir overnight at room temperature after which the solvent was removed under reduced pressure to give a colourless oil which was purified by flash column chromatography, eluting with CH_2Cl_2, to yield the sulfonyltriazole as a colourless solid (200 mg, 60%). R_f 0.62 (CH_2Cl_2); ν_{max}/cm^{-1} 3137, 3084, 1566, 1470, 1391, 1276, 1192, 1171, 1135, 1085, 1052, 1005, 954, 929, 854, 815, 737, 696, 641, 603, 568, 482 and 470. δ_H (500 MHz, $CDCl_3$) 7.94-7.91 (2H, d, *J* 8.8 Hz, phenyl 3,5-H_2), 7.86 (2H, s, 4,5-H_2), 7.80-7.77 (2H, d, *J* 8.7 Hz,phenyl 2,6-H_2). δ_C (125 MHz, CDCl3) 139.1 (phenyl C_2-3,5), 138.8 (C_2-4,5), 135.6 (phenyl C-1), 129.9 (phenyl C_2-2,6), 104.1 (phenyl C-4). HRMS found MNa^+ 357.9119. $C_8H_6IN_3O_2S$ requires *MNa*, 357.9118.

4-Iodophenyl sulfurofluoridate

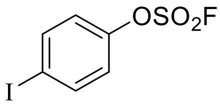

4-Iodophenol (220 mg, 1.0 mmol) was placed under nitrogen and dissolved in CH_2Cl_2 (3.3 mL, 0.2M). Pyridine (161 µL, 2.0 mmol) was added to the stirred solution which was then cooled in an acetone/dry-ice bath. SO_2Cl_2 (2.0 mL, 1.0 M in CH_2Cl_2) was added dropwise to the reaction mixture which was then allowed to reach room temperature. The reaction was allowed to stir overnight and was then diluted with EtOAc (10 mL) and transferred into a separation funnel together with 1 M aqueous hydrochloric acid (30 mL). The organic layer was collected and the aqueous was extracted with further EtOAc (3 × 30 mL). The combined organic layers were dried with Na_2SO_4, filtered, and the removal of solvent under reduced pressure gave the intermediate *sulfurochloridate* as a light brown oil (314 mg, >98%). 4-Iodophenyl sulfurochloridate (314 mg, 1.0 mmol) was dissolved in CH_3CN (3.3 mL) and AgF

(380 mg, 3.0 mmol) was added to the mixture. The reaction was then allowed to stir overnight at room temperature. The solution was then diluted with CH_3CN (5 mL) and filtered over Celite, the Celite cake was washed with EtOAc and the solvent was removed under reduced pressure to yield a light brown oil. The crude product was dissolved in chloroform (3 mL) and the colourless precipitate formed was filtered off and discarded. The filtrate was collected, and the solvent was removed under reduced pressure to yield the *fluorosulfate*[3] as a colourless solid (229 mg, 76%). R_F = 0.26 (hexane). δ_H (500 MHz, $CDCl_3$) 7.82-7.79 (2H, d, J 9.0 Hz, phenyl 3,5-H_2), 7.12-7.09 (2H, dd, J 9.0 and 0.91 Hz, phenyl 2,6-H_2). δ_C (125 MHz, $CDCl_3$) 150.0 (phenyl C-1), 139.7 (phenyl C_2-3,5), 123.0 (phenyl C_2-2,6), 93.6 (phenyl C-4). δ_F (376 MHz, $CDCl_3$) 37.93 (SO_3F). All data is consistent with known literature values.[3]

1-(Morpholin-4-yl)butane-1,3-dione

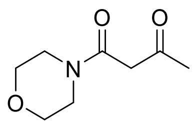

Morpholine (1.00 g, 11.5 mmol) was dissolved in toluene (100 mL) then 2,2,6-trimethyl-4*H*-1,3-dioxin-4-one (2.30 mL, 17.3 mmol) was added dropwise over 5 min and the resulting solution was stirred at rt for 15 min. The reaction mixture was stirred at 110 °C for a further 18 h then concentrated under reduced pressure to give a crude material. This was purified via column chromatography, eluting EtOAc–hexane 50:50 → EtOAc to give 1,3-dicarbonyl derivative[4] (1.85 g, 94%, *keto:enol* 85:15 by ^{1}H NMR) as a pale-yellow oil. R_f 0.17 (EtOAc). δ_H (500 MHz, $CDCl_3$): 14.55 (1H, s, OH^{enol}), 5.06 (1H, s, 2-H^{enol}), 3.64-3.60 (8H, m, morpholinyl 2,6-H_2), 3.59-3.56 (4H, m, morpholinyl 3-H_2 or morpholinyl 5-H_2), 3.51 (2H, s, 2-H_2^{keto}), 3.38-3.34 (4H, m, morpholinyl 3-H_2 or morpholinyl 5-H_2), 2.22 (3H, s, 4-H_3^{keto}), 1.90 (3H, s, 4-H_3^{enol}); δ_C (125 MHz, $CDCl_3$): 202.2 (C-3^{keto}), 175.5 (C-3^{enol}), 170.8 (C-1^{enol}), 165.0 (C-1^{keto}), 86.2 (C-2^{enol}), 66.7 (morpholinyl C_A-2,6), 66.6 (morpholinyl C_B-2,6), 49.8 (C-2^{keto}), 46.8 (morpholinyl C_A-3,5), 42.2 (morpholinyl C_B-3,5) 30.3 (C-4^{keto}), 22.0 (C-4^{enol}). All data is consistent with known literature values.[4]

2-Diazo-1-(morpholin-4-yl)butane-1,3-dione

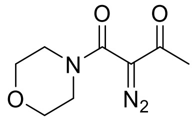

1-(Morpholin-4-yl)butane-1,3-dione (1.85 g, 10.8 mmol) and *p*-ABSA (2.86 g, 11.9 mmol) were dissolved in MeCN (40 mL) then Et_3N (1.66 mL, 11.9 mmol) was added at rt. The resulting solution was at rt for 24 h, then filtered through Celite (eluting with EtOAc) to remove any solids. The filtrate was concentrated under reduced pressure to give a crude material. This was then purified via column chromatography, eluting with EtOAc–hexane 50:50 → EtOAc to give the diazo derivative[4] (1.71 g, 80%) as a yellow oil. R_f 0.26 (EtOAc). δ_H (500 MHz, $CDCl_3$): 3.72-3.68 (4H, m, morpholinyl 2,6-H_2), 3.53-3.45 (4H, m, morpholinyl 3,5-H_2), 2.30 (3H, s, 4-H_3); δ_C (125 MHz, $CDCl_3$): 188.0 (C-3), 160.4 (C-1), 74.7 (C-2), 66.8 (morpholinyl C_2-2,6), 46.1 (morpholinyl C_2-3,5), 27.2 (C-4). All data is consistent with known literature values.[4]

4-[1-Diazo-2-(morpholin-4-yl)-2-oxoethyl]benzene-1-sulfonyl fluoride (D1)

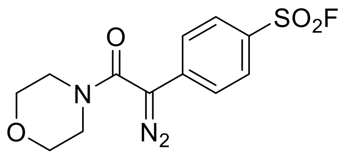

2-Diazo-1-(morpholin-4-yl)butane-1,3-dione (1.70 g, 8.63 mmol) was dissolved in MeCN (20 mL) then 10% aq. KOH (20 mL) was added and the resulting solution allowed to stir at rt for 18 h. EtOAc (50 mL) was added and the phases then separated. The aqueous phase was extracted with EtOAc (3 × 30 mL), organic phases combined, washed with sat. aq. ammonium chloride (40 mL), dried ($MgSO_4$), filtered and concentrated under reduced pressure to give the deacetylate diazo intermediate (736 mg, 55%) as a yellow oil. This intermediate (736 mg, 4.74 mmol) was then dissolved in toluene (20 mL) and $Pd(PPh_3)_4$ (274 mg, 5 mol %), Ag_2CO_3 (654 mg, 2.37 mmol), 4-iodobenzenesulfonyl fluoride (1.76 g, 6.12 mmol) and Et_3N (0.85 mL, 6.12 mmol) were added and the resulting mixture allowed to stir at rt for 4 h. The mixture was filtered through Celite (eluting with EtOAc) and the filtrate concentrated under reduced pressure to give a crude material. This was purified via column chromatography, eluting with EtOAc–hexane 70:30 to give the *sulfonyl fluoride diazo*

derivative ***D1*** (0.68 g, 46%) as a bright yellow oil that solidified upon drying to give a yellow solid. R_f 0.40 (EtOAc–hexane 70:30). ν_{max}/cm^{-1}: 2983, 2922, 2860, 2072, 1639, 1586, 1460, 1212, 1193; δ_H (500 MHz, $CDCl_3$): 7.96 (2H, d, *J* 8.8 Hz, aryl 2,6-H), 7.44 (2H, d, *J* 8.8 Hz, aryl 3,5-H), 3.75-3.70 (4H, m, morpholinyl 2,6-H_2), 3.55-3.52 (4H, m, morpholinyl 3,5-H_2); δ_C (125 MHz, $CDCl_3$): 163.2 (C-2), 136.9 (aryl C-4), 129.4 (aryl C_2-2,6), 128.9 (d, *J* 25.1 Hz, aryl C-1), 123.7 (aryl C_2-3,5), 66.7 (morpholinyl C_2-2,6), 46.2 (morpholinyl C_2-3,5) (C-1 not observed); δ_F (470 MHz, $CDCl_3$): 66.7 (SO_2F); HRMS found MNa^+ 336.0422. $C_{12}H_{12}FN_3O_4S$ requires *MNa*, 336.0425.

4-(1-Diazo-2-morpholino-2-oxoethyl)phenyl sulfurofluoridate (D4)

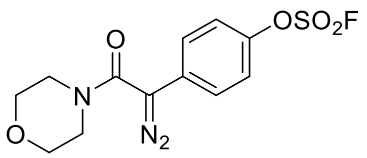

$Pd(PPh_3)_4$ (144 mg, 5 mol %) and Ag_2CO_3 (348 mg, 1.2 mmol) were added to 4-iodobenzenesulfonyl fluoride (1.00 g, 3.3 mmol). A solution of 2-diazo-1-morpholinoethan-1-one (388 mg, 2.5 mmol) in toluene (12.5 mL) was then transferred to the solid starting materials followed by Et_3N (348 µL, 2.5 mmol). The reaction was allowed to stir under nitrogen for 4 h, filtered over Celite and the Celite cake was washed with EtOAc (50 mL). The removal of solvent under reduced pressure yielded a brown oil which was purified by flash column chromatography, eluting EtOAc/hexane 0:100 → 30:70, to give the *sulfurofluoridate diazo derivative* **D4** as an orange oil (211 mg, 26%). R_f = 0.39 (EtOAc/hexane 50:50). ν_{max}/cm^{-1} 3105, 3067, 2964, 2922, 2855, 2063, 1626,1502, 1445, 1410, 1232, 1190, 1143, 1113, 1067, 1015, 988, 910, 839, 812, 766, 729, 646, 585, 543 and 510. δ_H (500 MHz, $CDCl_3$) 7.37-7.32 (4H, m, phenyl 3,5-H_2 and phenyl 2,6-H_2), 3.70 (4H, t, *J* 9.7 and 4.6 Hz, morpholino 2,6-H_2), 3.50 (4H, t, *J* 9.8 and 5.0 Hz, morpholino 3,5-H_2). δ_C (125 MHz, $CDCl_3$) 164.4 (oxoethyl C-2), 147.5 (phenyl C-1), 128.7 (phenyl C-4), 125.6 (phenyl C_2-3,5), 121.8 (phenyl C_2-2,6), 66.6 (morpholino C_2-2,6), 62.3 (oxoethyl C-1), 46.0 (morpholino C_2-3,5); δ_F (376 MHz, $CDCl_3$) 37.67 (SO_3*F*). HRMS found MH^+ 330.0545. $C_{12}H_{13}FN_3O_5S$ requires *MH*, 330.0554.

2-(4-((2*H*-1,2,3-Triazol-2-yl)sulfonyl)phenyl)-2-diazo-1-morpholinoethan-1-one (D5)

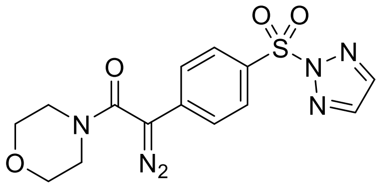

$Pd(PPh_3)_4$ (80 mg, 5 mol %) and Ag_2CO_3 (192 mg, 0.7 mmol) were added to 1-((4-iodophenyl)sulfonyl)-2*H*-1,2,3-triazole (462 mg, 1.4 mmol). A solution of 2-diazo-1-morpholinoethan-1-one (214 mg, 1.4 mmol) in toluene (7 mL) was transferred to the solid reagents followed by Et_3N (192 µL, 1.4 mmol). The reaction was allowed to stir under nitrogen for 4 h, filtered over Celite and the Celite cake was washed with EtOAc (50 mL). The removal of solvent under reduced pressure yielded a brown oil which was purified by flash column chromatography, eluting with EtOAc/hexane 0:100 → 30:70, to give the *sulfonyltriazole diazo derivative* **D5** as an orange oil (115 mg, 23%). R_f = 0.72 (EtOAc/hexane 80:20); ν_{max}/cm^{-1} 2962, 2922, 2856, 2070, 1630, 1585, 1493, 1394, 1321, 1274, 1250, 1187, 1166, 1113, 1092, 986, 954, 931, 835, 742, 672, 609, 576, 548 and 510. δ_H (500 MHz, $CDCl_3$) 8.06-8.04 (2H, m, phenyl 2,6-H_2), 7.84 (2H, s, triazolyl 4,5-H_2), 7.38-7.35 (2H, m, phenyl 3,5-H_2), 3.71-3.69 (4H, m, morpholino 3,5-H_2), 3.52-3.50 (4H, m, morphoplino 2,6-H_2). δ_C (125 MHz, $CDCl_3$) 163.1 (C-1), 138.5 (triazolyl C_2-4,5), 136.5 (phenyl C-1), 131.7 (phenyl C-4), 129.7 (phenyl C_2-2,6), 123.7 (phenyl C_2-3,5), 66.7 (C-2), 63.4 (morpholino C_2-2,6), 46.1 (morpholino C_2-3,5). HRMS found MH^+ 363.0863. $C_{14}H_{14}N_6O_4S$ *MH* requires, 363.0870.

1-(4-Phenylpiperidin-1-yl)butane-1,3-dione

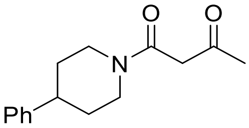

4-Phenylpiperidine (3.00 g, 18.6 mmol) was dissolved in toluene (200 mL) then 2,2,6-trimethyl-4*H*-1,3-dioxin-4-one (3.72 mL, 28.0 mmol) was added dropwise over 5 min and the resulting solution was stirred at rt for 15 min. The reaction mixture was stirred at 120 °C for a further 18 h then concentrated under reduced pressure to give a crude material. This was purified via column chromatography, eluting with EtOAc–hexane 50:50 to give *1,3-dicarbonyl derivative* (3.86 g, 85%, *keto:enol* 83:17 by ^{1}H NMR) as a yellow oil. R_f 0.38 (EtOAc). ν_{max}/cm^{-1}: 3002, 2920, 2855, 1718, 1630, 1491, 1389, 1268, 1226, 1007; δ_H (500 MHz, $CDCl_3$): 15.00 (1H, s, OH^{enol}), 7.34 (4H, t, *J* 7.5 Hz,

phenyl 3,5-H), 7.27-7.20 (6H, m, phenyl 2,6-H and phenyl 4-H), 5.27 (1H, s, 2-H^{enol}), 4.83-4.77 (2H, m, piperidinyl 2-H_A or piperidinyl 6-H_A), 3.88-3.82 (2H, m, piperidinyl 2-H_A or piperidinyl 6-H_A), 3.64 (2H, s, 2-H_2^{keto}), 3.19 (2H, app. td, *J* 13.2 and 2.4 Hz, piperidinyl 2-H_B or piperidinyl 6-H_B), 2.82-2.74 (2H, m, piperidinyl 4-H), 2.71 (2H, app. td, *J* 13.2 and 2.6 Hz, piperidinyl 2-H_B or piperidinyl 6-H_B), 2.32 (3H, s, 4-H_3^{keto}), 2.00 (3H, s, 4-H_3^{enol}), 1.96-1.90 (4H, m, piperidinyl 3,5-H_A), 1.68 (4H, app. pd, *J* 12.8 and 4.2 Hz, piperidinyl 3,5-H_B); δ_C (125 MHz, $CDCl_3$): 202.3 (C-3^{keto}), 175.0 (C-3^{enol}), 170.3 (C-1^{enol}), 164.7 (C-1^{keto}), 144.8 (phenyl C-1), 128.4 (phenyl C_2-3,5), 126.6 (phenyl C_2-2,6), 126.4 (phenyl C-4), 86.3 (C-2^{enol}), 49.9 (C-2^{keto}), 46.9 (piperidinyl C_A-2,6), 42.34 (piperidinyl C_B-2,6), 42.32 (piperidinyl C-4), 33.4 (piperidinyl C_A-3,5), 32.6 (piperidinyl C_B-3,5), 30.1 (C-4^{keto}), 21.9 (C-4^{enol}); HRMS found MH^+ 246.1483. $C_{15}H_{19}NO_2$ requires *MH*, 246.1489.

2-Diazo-1-(4-phenylpiperidin-1-yl)butane-1,3-dione

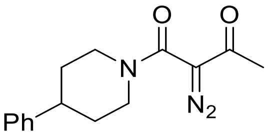

1-(4-Phenylpiperidin-1-yl)butane-1,3-dione (3.86 g, 15.6 mmol) and *p*-ABSA (4.15 g, 17.3 mmol) were dissolved in MeCN (80 mL) then Et_3N (2.41 mL, 17.3 mmol) was added at rt. The resulting solution was stirred at rt for 24 h then filtered through Celite (eluting with EtOAc) to remove any solids. The filtrate was concentrated under reduced pressure to give a crude material. This was then purified via column chromatography, eluting EtOAc–hexane 30:70 to give the *diazo derivative* (3.46 g, 82%) as a yellow oil. R_f 0.63 (EtOAc). ν_{max}/cm^{-1}: 3027, 2920, 2855, 2071, 1718, 1630, 1492, 1358, 1268, 1155, 1068; δ_H (500 MHz, $CDCl_3$): 7.34-7.30 (2H, m, phenyl 3,5-H), 7.25-7.20 (3H, m, phenyl 2,6-H and phenyl 4-H), 4.14 (2H, app. br. s, piperidinyl 2,6-H_A), 3.04 (2H, app. t, *J* 12.7 Hz, piperidinyl 2,6-H_B), 2.78 (1H, tt, *J* 12.2 and 3.6 Hz, piperidinyl 4-H), 2.36 (3H, s, 4-H_3), 1.97-1.91 (2H, m, piperidinyl 3,5-H_A), 1.74 (2H, qd, *J* 12.7 and 4.1 Hz, piperidinyl 3,5-H_B); δ_C (125 MHz, $CDCl_3$): 160.4 (C-1), 145.0 (phenyl C-1), 128.8 (phenyl C_2-3,5), 126.9 (phenyl C_2-2,6), 126.8 (phenyl C-4), 46.5 (piperidinyl C_2-2,6), 42.8 (piperidinyl C-4), 33.3 (piperidinyl C_2-3,5), 27.3 (C-4); HRMS found MNa^+ 294.1212. $C_{15}H_{17}N_3O_2$ requires *MNa*, 294.1213. C-2 and C-3 not observed by ^{13}C NMR (125 MHz).

4-[1-Diazo-2-oxo-2-(4-phenylpiperidin-1-yl)ethyl]benzene-1-sulfonyl fluoride (D2)

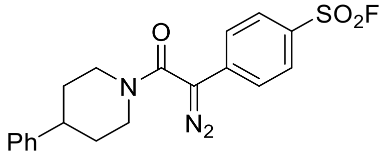

2-Diazo-1-(4-phenylpiperidin-1-yl)butane-1,3-dione (3.40 g, 12.5 mmol) was dissolved in MeCN (40 mL) then 10% aq. KOH (40 mL) was added and the resulting solution allowed to stir at rt for 18 h. EtOAc (80 mL) was added and the phases then separated. The aqueous phase was extracted with EtOAc (3 × 40 mL), organic phases combined, washed with sat. aq. ammonium chloride (40 mL), dried ($MgSO_4$), filtered and concentrated under reduced pressure to give the deacetylate diazo intermediate (2.53 g, 87%) as a yellow oil. This intermediate (2.53 g, 10.9 mmol) was then dissolved in toluene (50 mL) and $Pd(PPh_3)_4$ (485 mg, 5 mol %), Ag_2CO_3 (1.16 g, 4.20 mmol), 4-iodobenzenesulfonyl fluoride (2.40 g, 8.39 mmol) and Et_3N (1.52 mL, 10.9 mmol) were added and the resulting mixture allowed to stir at rt for 4 h. The mixture was filtered through Celite (eluting with EtOAc) and the filtrate concentrated under reduced pressure to give a crude material. This was purified via column chromatography, eluting EtOAc–hexane 20:80 to give the *sulfonyl fluoride diazo derivative* ***D2*** (1.72 g, 53%) as a yellow-orange solid. R_f 0.66 (EtOAc–hexane 50:50). ν_{max}/cm^{-1}: 3060, 2953, 2876, 2069, 1627, 1455, 1396, 1367, 1296, 1264, 1158, 1068; δ_H (500 MHz, $CDCl_3$): 7.96 (2H, d, *J* 8.8 Hz, aryl 2,6-H), 7.46 (2H, d, *J* 8.8 Hz, aryl 3,5-H), 7.36-7.30 (2H, m, phenyl 3,5-H), 7.26-7.19 (3H, m, phenyl 2,6-H and phenyl 4-H), 4.20 (2H, app. br. d, *J* 13.2 Hz, piperidinyl 2,6-H_A), 3.06 (2H, td, *J* 13.2 and 2.4 Hz, piperidinyl 2,6-H_B), 2.80 (1H, tt, *J* 12.1 and 3.5 Hz, piperidinyl 4-H), 1.97 (2H, app. br. d, *J* 13.9 Hz, piperidinyl 3,5-H_A), 1.71 (2H, app. qd, *J* 12.8 and 4.1 Hz, piperidinyl 3,5-H_B); δ_C (125 MHz, $CDCl_3$): 162.9 (oxoethyl C-2), 144.7 (phenyl C-1), 137.4 (aryl C-4), 129.3 (aryl C_2-2,6), 128.8 (phenyl C_2-3,5), 128.6 (d, *J* 24.9 Hz, aryl C-1), 126.9 (phenyl C-4), 126.8 (phenyl C_2-2,6), 123.6 (aryl C_2-3,5), 63.5 (oxoethyl C-1), 46.6 (piperidinyl C_2-2,6), 42.7 (piperidinyl C-4), 33.3 (piperidinyl C_2-3,5); δ_F (470 MHz, $CDCl_3$): 66.8 (SO_2F); HRMS found MH^+ 388.1115. $C_{19}H_{18}FN_3O_3S$ requires *MH*, 388.1126.

1-(2,3-Dihydro-1*H*-isoindol-2-yl)butane-1,3-dione

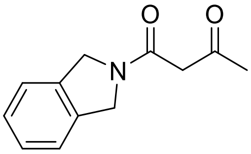

Isoindoline (1.00 g, 8.40 mmol) was dissolved in toluene (80 mL) then 2,2,6-trimethyl-4*H*-1,3-dioxin-4-one (1.67 mL, 12.6 mmol) was added dropwise over 5 min and the resulting solution was stirred at rt for 15 min. The reaction mixture was stirred at 120 °C for a further 18 h then concentrated under reduced pressure to give a crude material. This was purified via column chromatography, eluting EtOAc–hexane 50:50 → EtOAc to give *1,3-dicarbonyl derivative* (1.51 g, 88%, *keto:enol* 75:25 by ^{1}H NMR) as a dark brown solid. R_f 0.38 (EtOAc). ν_{max}/cm^{-1}: 3030, 2908, 2866, 1712, 1632, 1455, 1355, 1224, 1161; δ_H (500 MHz, $CDCl_3$): 14.58 (1H, s, OH^{enol}), 7.35-7.19 (8H, m, isoindolyl 4,5,6,7-H), 5.10 (1H, s, 2-H^{enol}), 4.81 (4H, s, isoindolyl 1,3-H_2^{keto}), 4.74 (4H, s, isoindolyl 1,3-H_2^{enol}), 3.60 (2H, s, 2-H_2^{keto}), 2.34 (3H, s, 4-H_3^{keto}), 1.99 (3H, s, 4-H_3^{enol}); δ_C (125 MHz, $CDCl_3$): 202.2 (C-3^{keto}), 175.0 (C-3^{enol}), 170.7 (C-1^{enol}), 165.4 (C-1^{keto}), 136.3 (isoindolyl C_A-3a,7a^{enol}), 136.1 (isoindolyl C_A-3a,7a^{keto}), 136.0 (isoindolyl C_B-3a,7a^{enol}), 135.8 (isoindolyl C_B-3a,7a^{keto}), 128.0 (isoindolyl C_A-5,6^{keto}), 127.9 (isoindolyl C_A-5,6^{enol}), 127.7 (isoindolyl C_B-5,6^{keto}), 127.6 (isoindolyl C_B-5,6^{enol}), 123.1 (isoindolyl C_2-4,7^{keto}), 122.7 (isoindolyl C_2-4,7^{enol}), 88.5 (C-2^{enol}), 53.1 (isoindolyl C_A-1,3^{keto}), 52.4 (isoindolyl C_B-1,3^{keto}), 52.2 (isoindolyl C_A-1,3^{enol}), 51.4 (isoindolyl C_B-1,3^{enol}), 51.1 (C-2^{keto}), 30.5 (C-4^{keto}), 21.9 (C-4^{enol}); HRMS found MNa^+ 226.0852. $C_{12}H_{13}NO_2$ requires *MNa*, 226.0838.

2-Diazo-1-(2,3-dihydro-1*H*-isoindol-2-yl)butane-1,3-dione

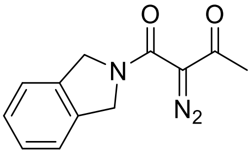

1-(2,3-Dihydro-1*H*-isoindol-2-yl)butane-1,3-dione (1.50 g, 7.39 mmol) and *p*-ABSA (1.95 g, 8.13 mmol) were dissolved in MeCN (40 mL) then Et_3N (1.13 mL, 8.13 mmol) was added at rt. The resulting solution was at rt for 24 h then filtered through Celite (eluting with EtOAc) to remove any solids. The filtrate was concentrated under reduced

pressure to give a crude material. This was then purified via column chromatography, eluting EtOAc–hexane 30:70 to give the *diazo derivative* (1.46 g, 86%) as a pale-yellow oil. R_f 0.62 (EtOAc). ν_{max}/cm^{-1}: 3033, 2930, 2866, 2094, 1650, 1612, 1400, 1355, 1232, 1088; δ_H (400 MHz, $CDCl_3$): 7.31-7.20 (4H, m, isoindolyl 4,5,6,7-H), 4.84 (4H, s, isoindolyl 1,3-H_2), 2.40 (3H, s, 4-H_3); δ_C (100 MHz, $CDCl_3$): 190.0 (C-3), 159.9 (C-1), 153.7 (isoindolyl C_2-3a,7a), 127.9 (isoindolyl C_2-5,6), 122.7 (isoindolyl C_2-4,7), 73.7 (C-2), 53.3 (isoindolyl C_2-1,3), 27.9 (C-4); HRMS found MNa^+ 252.0748. $C_{12}H_{11}N_3O_2$ requires *MNa*, 252.0743.

4-[1-Diazo-2-(2,3-dihydro-1*H*-isoindol-2-yl)-2-oxoethyl]benzene-1-sulfonyl fluoride (D3)

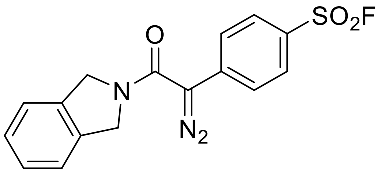

2-Diazo-1-(2,3-dihydro-1*H*-isoindol-2-yl)butane-1,3-dione (1.40 g, 6.11 mmol) was dissolved in MeCN (20 mL) then 10% aq. KOH (20 mL) was added and the resulting solution allowed to stir at rt for 18 h. EtOAc (40 mL) was added and the phases then separated. The aqueous phase was extracted with EtOAc (3 × 30 mL), organic phases combined, washed with sat. aq. ammonium chloride (40 mL), dried ($MgSO_4$), filtered and concentrated under reduced pressure to give the deacetylated diazo intermediate (1.15 g, 99%) as a yellow oil. This intermediate (1.15 g, 6.05 mmol) was then dissolved in toluene (30 mL) and $Pd(PPh_3)_4$ (269 mg, 5 mol %), Ag_2CO_3 (643 mg, 2.33 mmol), 4-iodobenzenesulfonyl fluoride (1.33 g, 4.66 mmol) and Et_3N (0.84 mL, 6.05 mmol) were added and the resulting mixture allowed to stir at rt for 4 h. The mixture was filtered through Celite (eluting with EtOAc) and the filtrate concentrated under reduced pressure to give a crude material. This was purified via column chromatography, eluting EtOAc–hexane 15:85 to give the *sulfonyl fluoride diazo derivative* **D3** (0.26 g, 12%) as a yellow solid. R_f 0.19 (EtOAc–hexane 15:85). ν_{max}/cm^{-1}: 3047, 2857, 2074, 1625, 1585, 1393, 1230, 1210, 1184; δ_H (500 MHz, $CDCl_3$): 7.98 (2H, d, *J* 8.8 Hz, aryl 2,6-H), 7.60 (2H, d, *J* 8.8 Hz, aryl 3,5-H), 7.36-7.28 (4H, m, isoindolyl 4,5,6,7-H), 4.91 (4H, s, isoindolyl 1,3-H_2); δ_C (125 MHz, $CDCl_3$): 161.9 (oxoethyl C-2), 136.7 (aryl C-4), 135.7 (isoindolyl C_2-3a,7a), 129.3 (aryl C_2-2,6), 129.0 (d, *J* 25.0 Hz, aryl C-1), 128.2

(isoindolyl C_2-5,6), 124.3 (aryl C_2-3,5), 122.9 (isoindolyl C_2-4,7), 63.5 (oxoethyl C-1), 53.6 (isoindolyl C_2-1,3); δ_F (470 MHz, $CDCl_3$): 66.7 (SO_2F); HRMS found MNa^+ 368.0470. $C_{16}H_{12}FN_3O_3S$ requires *MNa*, 368.0476.

4-[(1*R,1a*S**,6a*S**)-1-(4-Phenylpiperidine-1-carbonyl)-1*H*,1a*H*,6*H*,6a*H*-cyclopropa[*a*]inden-1-yl]benzene-1-sulfonyl fluoride (2-7)**

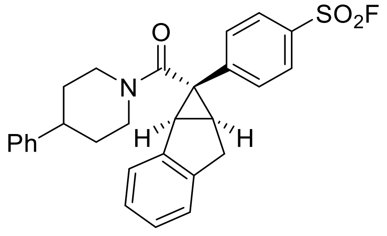

Prepared according to general procedure A – implementation of reaction array, diazo substrate **D2** (100 mM), co-substrate **C7** (500 mM) and $Rh_2(piv)_4$ (1 mM) gave a crude material. This was then purified via preparative HPLC eluting with gradient elution: 5:95 → 55:45 → 75:25 → 95:5 MeCN–H_2O to give the *cyclopropane derivative* ***2-7*** (1.20 mg, 13%, *dr* >95:<5 by ^{1}H NMR) as a colourless oil. R_f 0.49 (EtOAc–hexane 50:50). ν_{max}/cm^{-1}: 2924, 2854, 1634, 1432, 1409, 1236, 1213, 1100; δ_H (500 MHz, $CDCl_3$, some peak broadening due to unresolved rotamers): 7.68 (2H, d, *J* 8.6 Hz, aryl 2,6-H), 7.50-7.44 (1H, m, indenyl 2-H), 7.35-7.23 (4H, m, aryl 3,5-H and phenyl 3,5-H), 7.23-7.16 (2H, m, phenyl 4-H and indenyl 3-H), 7.18-6.90 (3H, m, phenyl 2,6-H and indenyl 4-H), 6.85 (1H, d, *J* 7.5 Hz, indenyl 5-H), 4.70-4.55 (2H, m, piperidinyl 2,6-H_A), 3.30-3.22 (2H, m, indenyl 1a-H and indenyl 6-H_A), 2.96 (1H, app. t, *J* 6.8 Hz, indenyl 6a-H), 2.90-2.60 (3H, m, piperidinyl 2,6-H_B and piperidinyl 4-H), 2.49 (1H, d, *J* 17.9 Hz, indenyl 6-H_B), 2.08-1.50 (4H, br. m, piperidinyl 3,5-H_2); δ_C (125 MHz, $CDCl_3$): 169.0 (C=O), 144.8 (aryl C-4 and phenyl C-1), 143.4 (indenyl C-5a), 141.0 (indenyl C-1b), 131.8 (aryl C_2-3,5), 131.0 (d, *J* 24.6 Hz, aryl C-1), 128.8 (phenyl C_2-3,5), 128.0 (aryl C_2-2,6), 127.4 (indenyl C-4), 127.0 (indenyl C-3), 126.8 (phenyl C-4), 126.7 (phenyl C_2-2,6), 125.2 (indenyl C-5), 124.5 (indenyl C-2), 42.6 (piperidinyl C-4), 40.1 (C-1), 37.1 (indenyl C-1a), 32.9 (indenyl C-6), 32.8 (piperidinyl C_2-3,5), 29.6 (indenyl C-6a); δ_F (470 MHz, $CDCl_3$): 65.9 (SO_2F); HRMS found MH^+ 476.1683. $C_{28}H_{26}FNO_3S$ requires *MH*, 476.1690. Piperidinyl C_2-2,6 not observed by ^{13}C NMR (125 MHz). The relative configuration was determined though NOESY (500 MHz). nOe observed between indenyl 6-H_B and aryl 3,5-H and nOe observed between indenyl 1a-H and indenyl 6a-H.

4-[2-Oxo-2-(4-phenylpiperidin-1-yl)-1-[(pyrimidin-2-yl)amino]ethyl]benzene-1-sulfonyl fluoride (2-13)

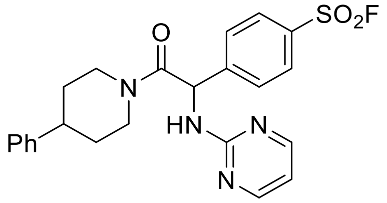

Prepared according to general procedure A – implementation of reaction array, diazo substrate **D2** (100 mM), co-substrate **C13** (500 mM) and $Rh_2(piv)_4$ (1 mM) gave a crude material. This was then purified via preparative HPLC eluting with gradient elution: 5:95 → 55:45 → 75:25 → 95:5 MeCN–H_2O to give the *aminopyrimidine derivative* ***2-13*** (1.10 mg, 12%, *rotamers* 52:48 by ^{1}H NMR) as a colourless oil. R_f 0.10 (EtOAc–hexane 50:50). ν_{max}/cm^{-1}: 3225, 3030, 2924, 2854, 1639, 1581, 1507, 1447, 1210, 1098; δ_H (500 MHz, $CDCl_3$): 8.27 (4H, t, *J* 4.7 Hz, pyrimidinyl 4,6-H), 8.02 (2H, d, *J* 8.3 Hz, aryl 2,6-H^{min}), 7.98 (2H, d, *J* 8.3 Hz, aryl 2,6-H^{maj}), 7.89 (2H, d, *J* 8.3 Hz, aryl 3,5-H^{min}), 7.81 (2H, d, *J* 8.3 Hz, aryl 3,5-H^{maj}), 7.31 (2H, t, *J* 7.5 Hz, phenyl 3,5-H^{maj}), 7.28-7.15 (6H, m, phenyl 3,5-H^{min}, phenyl 2,6-H^{maj} and phenyl 4-H), 6.97 (2H, d, *J* 7.5 Hz, phenyl 2,6-H^{min}), 6.85 (1H, d, *J* 7.5 Hz, NH^{min}), 6.82 (1H, d, *J* 6.8 Hz, NH^{maj}), 6.58 (2H, app. q, *J* 4.7 Hz, pyrimidinyl 5-H), 6.13 (2H, app. t, *J* 7.5 Hz, 1-H), 4.81 (2H, app. d, *J* 13.1 Hz, piperidinyl 2-H_A or piperidinyl 6-H_A) 4.07 (2H, app. t, *J* 13.1 Hz, piperidinyl 2-H_A or piperidinyl 6-H_A), 3.22 (2H, app. t, *J* 12.0 Hz, piperidinyl 2-$H_B{}^{min}$ or piperidinyl 6-$H_B{}^{min}$), 2.90 (2H, app. t, *J* 12.0 Hz, piperidinyl 2-$H_B{}^{min}$ or piperidinyl 6-$H_B{}^{min}$), 2.80-2.64 (4H, m, piperidinyl 2,6-$H_B{}^{maj}$ and piperidinyl 4-H), 1.96-1.64 (6H, m, piperidinyl 3,5-H_A and piperidinyl 3,5-$H_B{}^{min}$), 1.42 (2H, qd, *J* 12.7 and 4.1 Hz, piperidinyl 3-$H_B{}^{maj}$ or piperidinyl 5-$H_B{}^{maj}$), 0.71 (2H, qd, *J* 12.7 and 4.1 Hz, piperidinyl 3-$H_B{}^{maj}$ or piperidinyl 5-$H_B{}^{maj}$); δ_C (125 MHz, $CDCl_3$): 167.5 (C-2^{maj}), 167.4 (C-2^{min}), 160.7 (pyrimidinyl C-2^{min}), 160.6 (pyrimidinyl C-2^{maj}), 158.2 (pyrimidinyl C_2-4,6), 147.5 (phenyl C-1^{min}), 147.0 (phenyl C-1^{maj}), 144.7 (aryl C-4^{min}), 144.4 (aryl C-4^{maj}), 132.6 (d, *J* 24.9 Hz, aryl C-1), 129.6 (aryl C_2-3,5^{min}), 129.4 (aryl C_2-3,5^{maj}), 129.1 (phenyl C_2-3,5^{min}), 129.0 (phenyl C_2-3,5^{maj}), 128.8 (aryl C_2-2,6), 126.9 (phenyl C-4), 126.8 (phenyl C_2-2,6^{maj}), 126.6 (phenyl C_2-2,6^{min}), 111.9 (pyrimidinyl C-5^{min}), 111.8 (pyrimidinyl C-5^{maj}), 54.8 (C-1^{maj}), 54.7 (C-1^{min}), 46.5 (piperidinyl C_A-2,6^{maj}), 46.2 (piperidinyl C_B-2,6^{maj}), 43.7 (piperidinyl C_A-2,6^{min}), 43.6 (piperidinyl C_B-2,6^{min}), 42.7 (piperidinyl C-4^{maj}), 42.3 (piperidinyl C-4^{min}), 33.9 (piperidinyl C_A-3,5^{maj}), 32.9 (piperidinyl C_B-3,5^{maj}),

32.8 (piperidinyl C_A-3,5^{min}), 32.7 (piperidinyl C_B-3,5^{min}); δ_F (470 MHz, $CDCl_3$): 66.2 (SO_2F^{min}), 66.0 (SO_2F^{maj}); HRMS found MH^+ 455.1552. $C_{23}H_{23}FN_4O_3S$ requires *MH*, 455.1548.

4-{1-[(2-Bromophenyl)methoxy]-2-(2,3-dihydro-1*H*-isoindol-2-yl)-2-oxoethyl}benzene-1-sulfonyl fluoride (3-2)

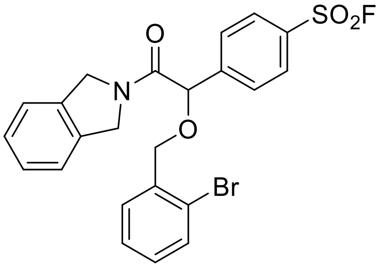

Prepared according to general procedure A – implementation of reaction array, diazo substrate **D3** (100 mM), co-substrate **C2** (500 mM) and $Rh_2(piv)_4$ (1 mM) gave a crude material. This was then purified via preparative HPLC eluting with gradient elution: 5:95 → 55:45 → 75:25 → 95:5 MeCN–H_2O to give the *ether derivative* ***3-2*** (1.30 mg, 13%) as a colourless oil. R_f 0.63 (EtOAc–hexane 50:50). ν_{max}/cm^{-1}: 2920, 2874, 1650, 1412, 1213, 1096; δ_H (500 MHz, $CDCl_3$): 8.30 (2H, d, *J* 8.6 Hz, aryl 2,6-H), 7.85 (2H, d, *J* 8.6 Hz, aryl 3,5-H), 7.58 (1H, dd, *J* 7.6 and 1.1 Hz, bromophenyl 3-H), 7.52 (1H, dd, *J* 7.6 and 1.5 Hz, bromophenyl 6-H), 7.35 (1H, td, *J* 7.6 and 1.1 Hz, bromophenyl 5-H), 7.29-7.24 (3H, m, isoindolyl 5,6-H and isolindolyl 4-H or isolindolyl 7-H), 7.21 (1H, td, *J* 7.6 and 1.5 Hz, bromophenyl 4-H), 7.16 (1H, d, *J* 6.8 Hz, isoindolyl 4-H or isoindolyl 7-H), 5.39 (1H, s, 1-H), 4.92 (2H, d, *J* 15.2 Hz, isoindolyl 1,3-H_A), 4.87-4.80 (2H, m, bromophenylmethoxy 1-H_A and isoindolyl 1-H_B or isoindolyl 3-H_B), 4.76 (1H, d, *J* 12.2 Hz, bromophenylmethoxy 1-H_B), 4.58 (1H, d, *J* 15.2 Hz, isoindolyl 1-H_B or isoindolyl 3-H_B); δ_C (125 MHz, $CDCl_3$): 167.8 (C-2), 144.4 (aryl C-4), 136.2 (isoindolyl C_A-3a,7a), 136.0 (isoindolyl C_B-3a,7a), 135.3 (bromophenyl C-1), 133.1 (bromophenyl C-3), 133.0 (d, *J* 24.9 Hz, aryl C-1), 130.2 (bromophenyl C-6), 130.1 (bromophenyl C-4), 129.0 (aryl C_2-2,6), 128.1 (aryl C_2-3,5), 127.8 (bromophenyl C-5 and isoindolyl C_2-5,6), 123.6 (bromophenyl C-2), 123.0 (isoindolyl C_A-4,7), 122.7 (isoindolyl C_B-4,7), 81.1 (C-1), 72.0 (bromophenylmethoxy C-1), 53.5 (isoindolyl C_A-1,3), 51.9 (isoindolyl C_B-1,3); δ_F (470 MHz, $CDCl_3$): 66.1 (SO_2F); HRMS found MH^+ 504.0267. $C_{23}H_{19}BrFNO_4S$ requires *MH*, 504.0275.

4-{1-[3-(2-Hydroxyethyl)-1*H*-indol-1-yl]-2-(morpholin-4-yl)-2-oxoethyl}benzene-1-sulfonyl fluoride (1-3a) and 4-{1-[2-(1*H*-Indol-3-yl)ethoxy]-2-(morpholin-4-yl)-2-oxoethyl}benzene-1-sulfonyl fluoride (1-3b)

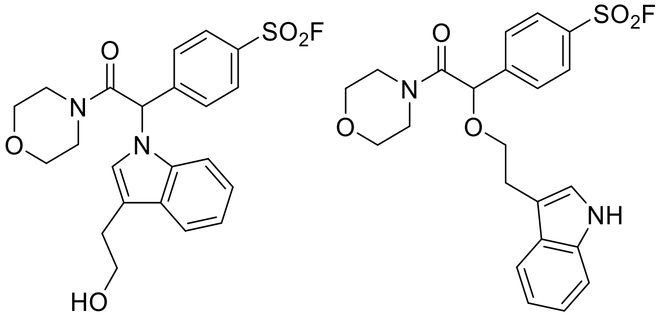

Prepared according to general procedure A – implementation of reaction array, diazo substrate **D1** (100 mM), co-substrate **C3** (500 mM) and $Rh_2(pfb)_4$ (1 mM) gave a crude material. This was then purified via preparative HPLC eluting with gradient elution: 5:95 → 35:65 → 60:40 → 95:5 MeCN–H_2O to give the *indole derivative* ***1-3a*** (1.30 mg, 15%) as a colourless oil. R_f 0.05 (EtOAc–hexane 50:50). ν_{max}/cm^{-1}: 3433, 2925, 2858, 1651, 1459, 1410, 1213, 1115, 1035; δ_H (500 MHz, $CDCl_3$): 7.96 (2H, d, *J* 8.5 Hz, aryl 2,6-H), 7.69-7.67 (1H, m, indolyl 4-H), 7.31 (2H, d, *J* 8.5 Hz, aryl 3,5-H), 7.24-7.14 (3H, m, indolyl 5,6,7-H), 7.09 (1H, s, indolyl 2-H), 6.40 (1H, s, 1-H), 3.94 (2H, t, *J* 6.0 Hz, hydroxyethyl 2-H_2), 3.90-3.84 (1H, m, morpholinyl 3-H_A or morpholinyl 5-H_A), 3.78-3.71 (1H, m, morpholinyl 2-H_A or morpholinyl 6-H_A), 3.68-3.55 (2H, m, morpholinyl 3,5-H_B and morpholinyl 2,6-H_B), 3.48 (1H, ddd, *J* 11.4, 6.0 and 2.9 Hz, morpholinyl 2-H_A or morpholinyl 6-H_A), 3.42-3.34 (1H, m, morpholinyl 3-H_A or morpholinyl 5-H_B), 3.27-3.17 (2H, m, morpholinyl 2,6-H_B and morpholinyl 3,5-H_B), 3.06 (2H, t, *J* 6.0 Hz, hydroxyethyl 1-H_2), 1.45 (1H, br. s, OH); δ_C (125 MHz, $CDCl_3$): 165.8 (C-2), 145.0 (aryl C-4), 136.1 (indolyl C-7a), 133.0 (d, *J* 25.0 Hz, aryl C-1), 128.93 (aryl C_2-2,6), 128.88 (aryl C_2-3,5), 128.7 (indolyl C-3a), 124.3 (indolyl C-2), 123.3 (indolyl C-6), 120.8 (indolyl C-5), 120.0 (indolyl C-4), 114.4 (indolyl C-3), 108.9 (indolyl C-7), 66.9 (morpholinyl C_A-2,6), 66.3 (morpholinyl C_B-2,6), 62.8 (hydroxyethyl C-2), 59.7 (C-1), 46.4 (morpholinyl C_A-3,5), 43.3 (morpholinyl C_B-3,5), 28.8 (hydroxyethyl C-1); δ_F (470 MHz, $CDCl_3$): 66.0 (SO_2F); HRMS found MH^+ 447.1381. $C_{22}H_{23}FN_2O_5S$ requires *MH*, 447.1384.

Also obtained was the *indole derivative* ***1-3b*** (0.10 mg, 1%) as a colourless oil. R_f 0.10 (EtOAc–hexane 50:50). ν_{max}/cm^{-1}: 3432, 2921, 2855, 1651, 1459, 1412, 1213, 1114,

1036; δ_H (500 MHz, $CDCl_3$): 8.03 (1H, br. s, NH), 7.96 (2H, d, *J* 8.6 Hz, aryl 2,6-H), 7.65-7.59 (3H, m, aryl 3,5-H and indolyl 4-H), 7.39 (1H, dt, *J* 8.2 and 0.9 Hz, indolyl 7-H), 7.24-7.20 (1H, m, indolyl 6-H), 7.13 (1H, ddd, *J* 8.2, 7.0 and 1.0 Hz, indolyl 5-H), 7.08 (1H, d, *J* 2.3 Hz, indolyl 2-H), 5.25 (1H, s, 1-H), 3.98 (1H, dt, *J* 8.9 and 6.7 Hz, ethoxyindolyl 2-H_A), 3.91 (1H, dt, *J* 8.9 and 6.1 Hz, ethoxyindolyl 2-H_B), 3.59-3.43 (4H, m, morpholinyl 2-H_2 or morpholinyl 6-H_2 and morpholinyl 3-H_2 or morpholinyl 5-H_2), 3.31-2.94 (6H, m, morpholinyl 2-H_2 or morpholinyl 6-H_2, morpholinyl 3-H_2 or morpholinyl 5-H_2 and ethoxyindolyl 1-H_2); δ_C (125 MHz, $CDCl_3$): 167.7 (C-2), 145.3 (aryl C-4), 136.2 (indolyl C-7a), 128.8 (aryl C_2-2,6), 127.0 (aryl C_2-3,5), 122.4 (indolyl C-6), 122.2 (indolyl C-2), 120.7 (indolyl C-3a), 119.6 (indolyl C-5), 118.8 (indolyl C-4), 112.8 (indolyl C-3), 111.4 (indolyl C-7), 83.0 (C-1), 71.4 (ethoxyindolyl C-2), 66.8 (morpholinyl C_A-2,6), 66.2 (morpholinyl C_B-2,6), 45.4 (morpholinyl C_A-3,5), 42.8 (morpholinyl C_B-3,5), 26.0 (ethoxyindolyl C-1); δ_F (470 MHz, $CDCl_3$): 66.1 (SO_2F); HRMS found MNa^+ 469.1204. $C_{22}H_{23}FN_2O_5S$ requires *MNa*, 469.1204. Aryl C-1 not observed by ^{13}C NMR.

4-{1-[(2-Chloro-5-fluorophenyl)methoxy]-2-(morpholin-4-yl)-2-oxoethyl}benzene-1-sulfonyl fluoride (1-5)

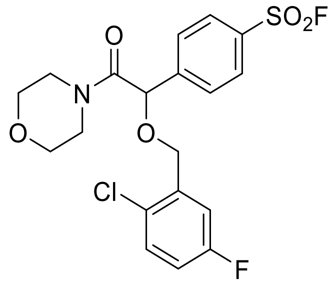

Prepared according to general procedure A – implementation of reaction array, diazo substrate **D1** (100 mM), co-substrate **C5** (500 mM) and $Rh_2(pfb)_4$ (1 mM) gave a crude material. This was then purified via preparative HPLC eluting with gradient elution: 5:95 → 35:65 → 60:40 → 95:5 MeCN–H_2O to give the *ether derivative **1-5*** (1.00 mg, 11%) as a colourless oil. R_f 0.34 (EtOAc–hexane 50:50). ν_{max}/cm^{-1}: 2964, 2925, 2857, 1650, 1412, 1301, 1213, 1115; δ_H (500 MHz, $CDCl_3$): 8.05 (2H, d, *J* 8.6 Hz, aryl 2,6-H), 7.75 (2H, d, *J* 8.6 Hz, aryl 3,5-H), 7.37 (1H, dd, *J* 8.8 and 5.0 Hz, chlorophenyl 3-H), 7.23 (1H, dd, *J* 8.8 and 3.0 Hz, chlorophenyl 6-H), 7.04-7.00 (1H, m, chlorophenyl 4-H), 5.41 (1H, s, 1-H), 4.81 (1H, d, *J* 12.7 Hz, chlorophenylmethoxy 1-H_A), 4.73 (1H,

d, *J* 12.7 Hz, chlorophenylmethoxy 1-H_B), 3.69-3.60 (4H, m, morpholinyl 2-H_2 or morpholinyl 6-H_2 and morpholinyl 3-H_2 or morpholinyl 5-H_2), 3.59-3.51 (1H, m, morpholinyl 3-H_A or morpholinyl 5-H_A), 3.49-3.42 (1H, m, morpholinyl 2-H_A or morpholinyl 6-H_A), 3.40-3.29 (2H, m, morpholinyl 3-H_B or morpholinyl 5-H_B and morpholinyl 2-H_B or morpholinyl 6-H_B); δ_C (125 MHz, $CDCl_3$): 167.2 (C-2), 161.6 (d, *J* 247.4 Hz, chlorophenyl C-5), 144.7 (aryl C-4), 136.4 (d, *J* 7.5 Hz, chlorophenyl C-1), 133.1 (d, *J* 25.1 Hz, aryl C-1), 131.1 (d, *J* 8.2 Hz, chlorophenyl C-3), 129.1 (aryl C_2-2,6), 127.9 (d, *J* 3.3 Hz, chlorophenyl C-2), 127.3 (aryl C_2-3,5), 116.7 (d, *J* 22.8 Hz, chlorophenyl C-4), 116.4 (d, *J* 24.1 Hz, chlorophenyl C-6), 82.3 (C-1), 69.5 (chlorophenylmethoxy C-1), 66.9 (morpholinyl C_A-2,6), 66.5 (morpholinyl C_B-2,6), 45.9 (morpholinyl C_A-3,5), 43.1 (morpholinyl C_B-3,5); δ_F (470 MHz, $CDCl_3$): 66.1 (SO_2F), -114.1 (chlorophenyl CF); HRMS found MNa^+ 468.0457. $C_{19}H_{18}ClF_2NO_5S$ requires *MNa*, 468.0454.

4-{1-[3-(2-Hydroxyethyl)-1*H*-indol-1-yl]-2-oxo-2-(4-phenylpiperidin-1-yl)ethyl}benzene-1-sulfonyl fluoride (2-3a) and 4-{1-[2-(1*H*-indol-3-yl)ethoxy]-2-oxo-2-(4-phenylpiperidin-1-yl)ethyl}benzene-1-sulfonyl fluoride (2-3b)

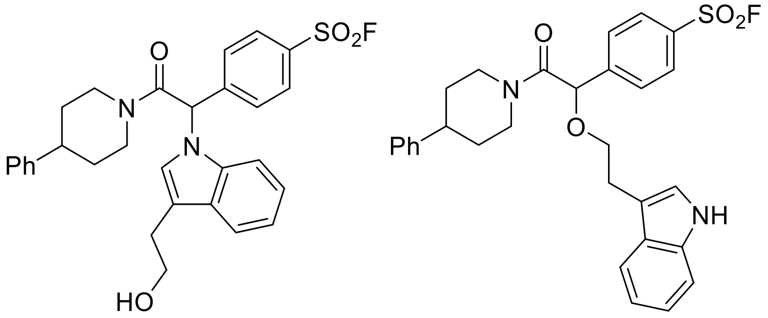

Prepared according to general procedure A – implementation of reaction array, diazo substrate **D2** (100 mM), co-substrate **C3** (500 mM) and $Rh_2(pfb)_4$ (1 mM) gave a crude material. This was then purified via preparative HPLC eluting with gradient elution: 5:95 → 55:45 → 75:25 → 95:5 MeCN–H_2O to give the *indole derivative **2-3a*** (1.40 mg, 13%, *rotamers* 38:62 by ^{1}H NMR) as a colourless oil. R_f 0.16 (EtOAc–hexane 50:50). ν_{max}/cm^{-1}: 3434, 3060, 2924, 2856, 1645, 1458, 1410, 1212, 1099; δ_H (500 MHz, $CDCl_3$): 7.98 (2H, d, *J* 8.6 Hz, aryl 2,6-H^{min}), 7.95 (2H, d, *J* 8.6 Hz, aryl 2,6-H^{maj}), 7.69-7.66 (2H, m, indolyl 4-H), 7.37 (2H, d, *J* 8.6 Hz, aryl 3,5-H^{min}), 7.34-7.30 (4H, m, aryl 3,5-H^{maj} and phenyl 4-H), 7-26-7.14 (14H, m, phenyl 2,6-H^{min}, phenyl 3,5-H, indolyl

2,5,6,7-H), 6.96-6.93 (2H, m, phenyl 2,6-H^{maj}), 6.51 (1H, s, 1-H^{min}), 6.50 (1H, s, 1-H^{maj}), 4.89-4.78 (2H, m, piperidinyl 2-H_A or piperidinyl 6-H_A), 3.93 (4H, q, *J* 6.6 Hz, hydroxyethyl 2-H_2), 3.86-3.76 (2H, m, piperidinyl 2-H_A or piperidinyl 6-H_A), 3.18 (1H, td, *J* 13.1 and 2.6 Hz, piperidinyl 2-H_B^{maj} or piperidinyl 6-H_B^{maj}), 3.09-3.03 (4H, m, hydroxyethyl 1-H_2), 2.99-2.93 (1H, m, piperidinyl 2-H_B^{min} or piperidinyl 6-H_B^{min}), 2.86-2.60 (4H, m, piperidinyl 2-H_B or piperidinyl 6-H_B and piperidinyl 4-H), 2.03-1.97 (1H, m, piperidinyl 3-H_A^{min} or piperidinyl 5-H_A^{min}), 1.93-1.88 (2H, m, piperidinyl 3-H_B or piperidinyl 5-H_B), 1.71 (1H, qd, *J* 12.8 and 4.2 Hz, piperidinyl 3-H_A^{maj} or piperidinyl 5-H_A^{maj}), 1.55-1.45 (5H, m, piperidinyl 3-H_A or piperidinyl 5-H_A, piperidinyl 3-H_B or piperidinyl 5-H_B and piperidinyl 3,5-H_B^{min}), 1.42 (2H, s, OH), 0.76 (1H, qd, *J* 12.8 and 4.2 Hz, piperidinyl 3-H_B^{maj} or piperidinyl 5-H_B^{maj}); δ_C (125 MHz, $CDCl_3$): 165.7 (C-2^{min}), 165.4 (C-2^{maj}), 145.5 (phenyl C-1^{maj}), 145.3 (phenyl C-1^{min}), 144.6 (aryl C-4^{maj}), 144.5 (aryl C-4^{min}), 136.3 (indolyl C-$7a^{min}$), 136.2 (indolyl C-$7a^{maj}$), 133.0 (d, *J* 25.1 Hz, aryl C-1^{min}), 132.8 (d, *J* 25.1 Hz, aryl C-1^{maj}), 129.0 (indolyl C-$3a^{maj}$), 129.0 (indolyl C-$3a^{min}$), 128.8 (aryl C_2-$2,6^{maj}$), 128.84 (aryl C_2-$3,5^{min}$), 128.7 (phenyl C-4), 126.9 (phenyl C_2-$3,5^{min}$), 126.8 (phenyl C_2-$3,5^{maj}$), 126.73 (phenyl C_2-$2,6^{min}$), 126.68 (phenyl C_2-$2,6^{maj}$), 124.8 (indolyl C-2^{maj}), 124.5 (indolyl C-2^{min}), 123.2 (indolyl C-6^{maj}), 123.1 (indolyl C-6^{min}), 120.6 (indolyl C-5), 120.0 (indolyl C-4^{maj}), 119.9 (indolyl C-4^{min}), 114.3 (indolyl C-3^{maj}), 113.9 (indolyl C-3^{min}), 109.2 (indolyl C-7^{maj}), 109.0 (indolyl C-7^{min}), 62.9 (hydroxyethyl C-2^{maj}), 62.8 (hydroxyethyl C-2^{min}), 59.9 (C-1^{maj}), 59.7 (C-1^{min}), 47.2 (piperidinyl C_A-$2,6^{maj}$), 46.4 (piperidinyl C_A-$2,6^{min}$), 44.1 (piperidinyl C_B-$2,6^{maj}$), 43.6 (piperidinyl C_B-$2,6^{min}$), 42.5 (piperidinyl C-4^{maj}), 42.3 (piperidinyl C-4^{min}), 33.8 (piperidinyl C_A-$3,5^{min}$), 33.1 (piperidinyl C_A-$3,5^{maj}$), 33.1 (piperidinyl C_B-$3,5^{maj}$), 32.8 (piperidinyl C_B-$3,5^{min}$), 28.9 (hydroxyethyl C-1^{min}), 28.8 (hydroxyethyl C-1^{maj}); δ_F (470 MHz, $CDCl_3$): 66.1 (SO_2F); HRMS found MH^+ 521.1908. $C_{29}H_{29}FN_2O_4S$ requires *MH*, 521.1905.

Also obtained was the *indole derivative* ***2-3b*** (0.10 mg, 1%, *rotamers* 45:55 by ^{1}H NMR) as a colourless oil. R_f 0.34 (EtOAc–hexane 50:50). ν_{max}/cm^{-1}: 3419, 3059, 2924, 2858, 1645, 1458, 1412, 1213, 1098; δ_H (500 MHz, $CDCl_3$): 7.97 (2H, d, *J* 8.6 Hz, aryl 2,6-H^{min}), 7.96 (2H, d, *J* 8.6 Hz, aryl 2,6-H^{maj}), 7.92 (2H, br. s, NH), 7.67 (4H, app. t, *J* 8.6 Hz, aryl 3,5-H), 7.61 (2H, app. t, *J* 8.0 Hz, indolyl 4-H), 7.37-7.15 (10H, m, indolyl 6,7-H and phenyl 3,4,5-H), 7.14-7.10 (2H, m, indolyl 5-H), 7.09-7.05 (4H, indolyl 2-H and phenyl 2,6-H^{min}), 7.00 (2H, d, *J* 7.3 Hz, phenyl 2,6-H^{maj}), 5.31 (1H, s,

1-H^{maj}), 5.29 (1H, s, 1-H^{min}), 4.70-4.62 (2H, m, piperidinyl 2-H_A or piperidinyl 6-H_A), 4.09-3.85 (6H, m, piperidinyl 2-H_A or piperidinyl 6-H_A and ethoxyindolyl 2-H_2), 3.23-3.15 (4H, m, ethoxyindolyl 1-H_2), 2.70-2.48 (6H, m, piperidinyl 2,6-H_B and piperidinyl 4-H), 1.91-1.79 (2H, m, piperidinyl 3-H_A or piperidinyl 5-H_A), 1.50-1.21 (4H, m, piperidinyl 3-H_A or piperidinyl 5-H_A and piperidinyl 3-H_B or piperidinyl 5-H_B), 0.89-0.77 (2H, m, piperidinyl 3-H_B or piperidinyl 5-H_B); δ_C (125 MHz, $CDCl_3$): 167.7 (C-2^{maj}), 167.5 (C-2^{min}), 146.01 (phenyl C-1^{maj}), 145.97 (phenyl C-1^{min}), 144.4 (aryl C-4^{min}), 144.3 (aryl C-4^{maj}), 136.4 (indolyl C-7a^{maj}), 136.1 (indolyl C-7a^{min}), 128.8 (indolyl C-3a), 128.7 (aryl C_2-2,6 and phenyl C-4), 127.4 (aryl C_2-3,6), 127.0 (phenyl C_2-3,5^{min}), 126.8 (phenyl C_2-3,5^{maj}), 126.71 (phenyl C_2-2,6^{min}), 126.67 (phenyl C_2-2,6^{maj}), 122.4 (indolyl C-6), 122.2 (indolyl C-2), 119.6 (indolyl C-5), 118.9 (indolyl C-4), 112.9 (indolyl C-3), 111.4 (indolyl C-7), 83.6 (C-1^{min}), 82.7 (C-1^{maj}), 71.7 (ethoxyindolyl C-2^{maj}), 70.9 (ethoxyindolyl C-2^{min}), 45.6 (piperidinyl C_A-2,6^{maj}), 45.4 (piperidinyl C_A-2,6^{min}), 43.5 (piperidinyl C_B-2,6), 42.4 (piperidinyl C-4), 33.4 (piperidinyl C_A-3,5^{min}), 33.1 (piperidinyl C_A-3,5^{maj}), 33.0 (piperidinyl C_B-3,5^{maj}), 32.8 (piperidinyl C_B-3,5^{min}), 26.1 (ethoxyindolyl C-1^{maj}), 26.0 (ethoxyindolyl C-1^{min}); δ_F (470 MHz, $CDCl_3$): 66.2 (SO_2F^{min}), 66.1 (SO_2F^{maj}); HRMS found MNa^+ 453.1720. $C_{29}H_{29}FN_2O_4S$ requires *MNa*, 543.1724. Aryl C-1 not observed by ^{13}C NMR (125 MHz).

4-[1-(Naphthalen-1-yloxy)-2-oxo-2-(4-phenylpiperidin-1-yl)ethyl]benzene-1-sulfonyl fluoride (2-4)

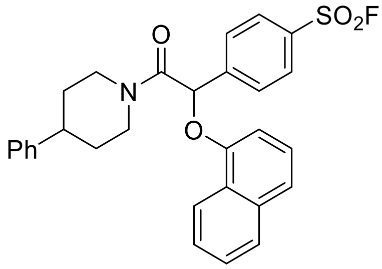

Prepared according to general procedure A – implementation of reaction array, diazo substrate **D2** (100 mM), co-substrate **C4** (500 mM) and $Rh_2(pfb)_4$ (1 mM) gave a crude material. This was then purified via preparative HPLC eluting with gradient elution: 5:95 → 55:45 → 75:25 → 95:5 MeCN–H_2O to give the *napthalene derivative* ***2-4*** (1.10 mg, 11%, *rotamers* 44:56 by ^{1}H NMR) as a colourless oil. R_f 0.65 (EtOAc–hexane 50:50). ν_{max}/cm^{-1}: 3060, 2932, 2853, 1646, 1493, 1412, 1264, 1214, 1100; δ_H (500

MHz, CDCl$_3$): 8.43-8.32 (2H, m, napthanenyl 8-H), 8.12 (4H, d, *J* 8.5 Hz, aryl 2,6-H), 8.01-7.98 (4H, m, aryl 3,5-H), 7.90-7.95 (2H, m, napthanenyl 5-H), 7.60-7.52 (6H, m, napthanenyl 4,6,7-H), 7.41 (1H, t, *J* 8.0 Hz, napthanenyl 3-H^{maj}), 7.37 (1H, t, *J* 8.0 Hz, napthanenyl 3-H^{min}), 7.30-2.25 (2H, m, phenyl 3,5-H^{min}), 7.22-7.09 (4H, m, phenyl 4-H and phenyl 3,5-H^{maj}), 7.08-7.02 (3H, m, phenyl 2,6-H^{min} and napthanenyl 2-H^{maj}), 6.95 (1H, d, *J* 7.7 Hz, napthanenyl 2-H^{min}), 6.73 (2H, d, *J* 7.0 Hz, phenyl 2,6-H^{maj}), 6.29 (2H, s, 1-H), 4.73 (2H, app. t, *J* 11.7 Hz, piperidinyl 2-H$_A$ or piperidinyl 6-H$_A$), 4.32 (2H, app. d, *J* 13.2 Hz, piperidinyl 2-H$_A$ or piperidinyl 6-H$_A$), 3.04 (1H, td, *J* 13.2 and 2.3 Hz, piperidinyl 2-H$_B^{min}$ or piperidinyl 6-H$_B^{min}$), 2.83 (1H, td, *J* 13.2 and 2.3 Hz, piperidinyl 2-H$_B^{maj}$ or piperidinyl 6-H$_B^{maj}$), 2.74-2.51 (4H, m, piperidinyl 2-H$_B$ or piperidinyl 6-H$_B$ and piperidinyl 4-H), 1.90 (1H, app. d, *J* 13.2 Hz, piperidinyl 3-H$_A^{min}$ or piperidinyl 5-H$_A^{min}$), 1.79 (1H, app. d, *J* 13.2 Hz, piperidinyl 3-H$_A^{maj}$ or piperidinyl 5-H$_A^{maj}$), 1.67-1.57 (2H, m, piperidinyl 3-H$_A$ or piperidinyl 5-H$_A$), 1.36-1.23 (2H, m, piperidinyl 3-H$_B$ or piperidinyl 5-H$_B$), 1.13-1.00 (2H, m, piperidinyl 3-H$_B$ or piperidinyl 5-H$_B$); δ$_C$ (125 MHz, CDCl$_3$): 166.7 (C-2^{maj}), 166.6 (C-2^{min}), 152.5 (napthanenyl C-1^{maj}), 152.2 (napthanenyl C-1^{min}), 144.8 (phenyl C-1^{min}), 144.7 (phenyl C-1^{maj}), 144.6 (aryl C-4^{min}), 144.5 (aryl C-4^{maj}), 134.9 (napthanenyl C-4a), 133.1 (d, *J* 26.2 Hz, aryl C-1), 129.2 (aryl C$_2$-2,6^{maj}), 129.1 (aryl C$_2$-2,6^{min}), 128.8 (phenyl C$_2$-3,5^{min}), 128.6 (phenyl C$_2$-3,5^{maj}), 128.1 (napthanenyl C-5), 127.2 (aryl C$_2$-3,5^{min}), 127.1 (aryl C$_2$-3,5^{maj}), 126.8 (phenyl C-4), 126.7 (phenyl C$_2$-2,6^{min}), 126.6 (phenyl C$_2$-2,6^{maj}), 126.24 (napthanenyl C-6), 126.16 (napthanenyl C-7), 125.9 (napthanenyl C-3), 125.5 (napthanenyl C-8a^{min}), 125.4 (napthanenyl C-8a^{maj}), 122.3 (naphthanenyl C-4), 121.5 (napthanenyl C-8^{min}), 121.4 (napthanenyl C-8^{maj}), 106.5 (napthanenyl C-2), 80.5 (C-1^{maj}), 80.0 (C-1^{min}), 46.2 (piperidinyl C$_A$-2,6^{maj}), 45.8 (piperidinyl C$_A$-2,6^{min}), 44.2 (piperidinyl C$_B$-2,6^{maj}), 43.8 (piperidinyl C$_B$-2,6^{min}), 42.4 (piperidinyl C-4^{maj}), 42.3 (piperidinyl C-4^{min}), 33.39 (piperidinyl C$_A$-3,5^{maj}), 33.35 (piperidinyl C$_A$-3,5^{min}), 32.9 (piperidinyl C$_B$-3,5); δ$_F$ (470 MHz, CDCl$_3$): 66.3 (SO$_2$F^{min}), 66.1 (SO$_2$F^{maj}); HRMS found MH$^+$ 504.1638. $C_{29}H_{26}FNO_4S$ requires *MH*, 504.1639.

4-{1-[(4-Chlorophenyl)methoxy]-2-(morpholin-4-yl)-2-oxoethyl}benzene-1-sulfonyl fluoride (1-1)

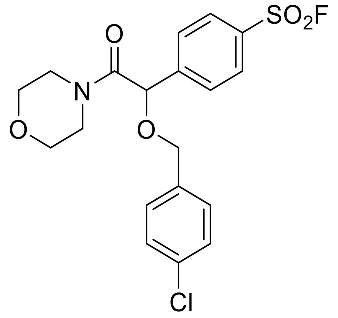

Prepared according to general procedure A – implementation of reaction array, diazo substrate **D1** (100 mM), co-substrate **C1** (500 mM) and $Rh_2(cap)_4$ (1 mM) gave a crude material. This was then purified via preparative HPLC eluting with gradient elution: 5:95 → 35:65 → 60:40 → 95:5 MeCN–H_2O to give the *ether derivative **1-1*** (1.20 mg, 14%) as a colourless oil. R_f 0.21 (EtOAc–hexane 50:50). ν_{max}/cm^{-1}: 2921, 2861, 1650, 1492, 1439, 1242, 1213, 1115, 1095; δ_H (500 MHz, $CDCl_3$): 8.03 (2H, d, *J* 8.6 Hz, aryl 2,6-H), 7.72 (2H, d, *J* 8.6 Hz, aryl 3,5-H), 7.37 (2H, d, *J* 8.5 Hz, chlorophenyl 3,5-H), 7.31 (2H, d, *J* 8.5 Hz, chlorophenyl 2,6-H), 5.33 (1H, s, 1-H), 4.71 (1H, d, *J* 11.7 Hz, chlorophenylmethoxy 1-H_A), 4.65 (1H, d, *J* 11.7 Hz, chlorophenylmethoxy 1-H_B), 3.70-3.57 (4H, m, morpholinyl 2-H_2 or morpholinyl 6-H_2 and morpholinyl 3-H_2 or morpholinyl 5-H_2), 3.56-3.49 (1H, m, morpholinyl 3-H_A or morpholinyl 5-H_A), 3.47-3.40 (1H, m, morpholinyl 2-H_A or morpholinyl 6-H_A), 3.35-3.25 (2H, m, morpholinyl 3-H_B or morpholinyl 5-H_B and morpholinyl 2-H_B or morpholinyl 6-H_B); δ_C (125 MHz, $CDCl_3$): 167.5 (C-2), 145.0 (aryl C-4), 134.8 (chlorophenyl C-1), 134.6 (chlorophenyl C-4), 133.0 (d, *J* 25.0 Hz, aryl C-1), 129.6 (chlorophenyl C_2-2,6), 129.1 (chlorophenyl C_2-3,5), 129.0 (aryl C_2-2,6), 127.2 (aryl C_2-3,5), 81.7 (C-1), 72.0 (chlorophenylmethoxy C-1), 66.9 (morpholinyl C_A-2,6), 66.5 (morpholinyl C_B-2,6), 45.8 (morpholinyl C_A-3,5), 43.1 (morpholinyl C_B-3,5); δ_F (470 MHz, $CDCl_3$): 66.1 (SO_2F); HRMS found MH^+ 450.0548. $C_{19}H_{19}ClFNO_5S$ requires *MH*, 450.0549.

4-{1-[(2-Bromophenyl)methoxy]-2-(morpholin-4-yl)-2-oxoethyl}benzene-1-sulfonyl fluoride (1-2)

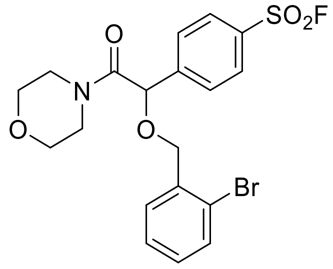

Prepared according to general procedure A – implementation of reaction array, diazo substrate **D1** (100 mM), co-substrate **C2** (500 mM) and $Rh_2(cap)_4$ (1 mM) gave a crude material. This was then purified via preparative HPLC eluting with gradient elution: 5:95 → 55:45 → 75:25 → 95:5 MeCN–H_2O to give the *ether derivative* ***1-2*** (1.10 mg, 12%) as a colourless oil. R_f 0.30 (EtOAc–hexane 50:50). ν_{max}/cm^{-1}: 2917, 2858, 1650, 1439, 1411, 1213, 1115, 1097; δ_H (500 MHz, $CDCl_3$): 8.04 (2H, d, *J* 8.6 Hz, aryl 2,6-H), 7.76 (2H, d, *J* 8.6 Hz, aryl 3,5-H), 7.61 (1H, dd, *J* 8.0 and 1.2 Hz, bromophenyl 3-H), 7.46 (1H, dd, *J* 7.6 and 1.5 Hz, bromophenyl 6-H), 7.36 (1H, td, *J* 7.6 and 1.2 Hz, bromophenyl 5-H), 7.24 (1H, td, *J* 7.8 and 1.5 Hz, bromophenyl 4-H), 5.40 (1H, s, 1-H), 4.82 (1H, d, *J* 12.1 Hz, bromophenylmethoxy 1-H_A), 4.76 (1H, d, *J* 12.1 Hz, bromophenylmethoxy 1-H_B), 3.70-3.60 (4H, m, morpholinyl 2-H_2 or morpholinyl 6-H_2 and morpholinyl 3-H_2 or morpholinyl 5-H_2), 3.56 (1H, ddd, *J* 10.4, 9.3 and 4.4 Hz, morpholinyl 3-H_A or morpholinyl 5-H_A), 3.45 (1H, ddd, *J* 10.8, 6.6 and 2.6 Hz, morpholinyl 2-H_A or morpholinyl 6-H_A), 3.37-3.27 (2H, m, morpholinyl 3-H_B or morpholinyl 5-H_B and morpholinyl 2-H_B or morpholinyl 6-H_B); δ_C (125 MHz, $CDCl_3$): 167.5 (C-2), 145.1 (aryl C-4), 135.8 (bromophenyl C-1), 130.2 (bromophenyl C-3), 132.9 (d, *J* 25.0 Hz, aryl C-1), 130.2 (bromophenyl C_2-4,6), 129.0 (aryl C_2-2,6), 127.8 (bromophenyl C-5), 127.3 (aryl C_2-3,5), 123.7 (bromophenyl C-2), 82.1 (C-1), 72.4 (bromophenylmethoxy C-1), 66.9 (morpholinyl C_A-2,6), 66.6 (morpholinyl C_B-2,6), 45.9 (morpholinyl C_A-3,5), 43.1 (morpholinyl C_B-3,5); δ_F (470 MHz, $CDCl_3$): 66.1 (SO_2F); HRMS found MNa^+ 494.0045. $C_{19}H_{19}BrFNO_5S$ requires *MNa*, 494.0044.

4-{1-[(4-Bromo-3,5-dimethoxyphenyl)methoxy]-2-(morpholin-4-yl)-2-oxoethyl}benzene-1-sulfonyl fluoride (1-8)

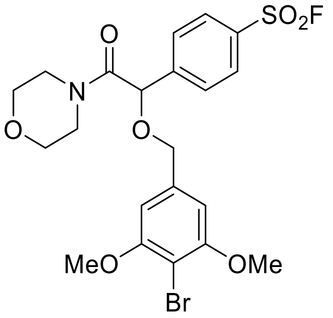

Prepared according to general procedure A – implementation of reaction array, diazo substrate **D1** (100 mM), co-substrate **C8** (500 mM) and $Rh_2(cap)_4$ (1 mM) gave a crude material. This was then purified via preparative HPLC eluting with gradient elution: 5:95 → 35:65 → 60:40 → 95:5 MeCN–H_2O to give the *ether derivative* ***1-8*** (1.30 mg, 12%) as a colourless oil. R_f 0.10 (EtOAc–hexane 50:50). ν_{max}/cm^{-1}: 2916, 2854, 1649, 1590, 1459, 1412, 1236, 1213, 1122, 1034; δ_H (500 MHz, $CDCl_3$): 8.04 (2H, d, *J* 8.6 Hz, aryl 2,6-H), 7.72 (2H, d, *J* 8.6 Hz, aryl 3,5-H), 6.58 (2H, s, dimethoxyphenyl 2,6-H), 5.34 (1H, s, 1-H), 4.71 (1H, d, *J* 12.0 Hz, dimethoxyphenylmethoxy 1-H_A), 4.65 (1H, d, *J* 12.0 Hz, dimethoxyphenylmethoxy 1-H_B), 3.91 (6H, s, OMe), 3.68-3.58 (4H, m, morpholinyl 2-H_2 or morpholinyl 6-H_2 and morpholinyl 3-H_2 or morpholinyl 5-H_2), 3.57-3.49 (1H, m, morpholinyl 3-H_A or morpholinyl 5-H_A), 3.48-3.39 (1H, m, morpholinyl 2-H_A or morpholinyl 6-H_A), 3.36-3.28 (2H, m, morpholinyl 3-H_B or morpholinyl 5-H_B and morpholinyl 2-H_B or morpholinyl 6-H_B); δ_C (125 MHz, $CDCl_3$): 167.5 (C-2), 157.5 (dimethoxyphenyl C_2-3,5), 145.0 (aryl C-4), 137.0 (dimethoxyphenyl C-1), 133.1 (d, *J* 25.1 Hz, aryl C-1), 129.1 (aryl C_2-2,6), 127.2 (aryl C_2-3,5), 104.5 (dimethoxyphenyl C_2-2,6), 101.2 (dimethoxyphenyl C-4), 81.4 (C-1), 72.7 (dimethoxyphenylmethoxy C-1), 66.9 (morpholinyl C_A-2,6), 66.5 (morpholinyl C_B-2,6), 56.7 (OMe), 45.9 (morpholinyl C_A-3,5), 43.1 (morpholinyl C_B-3,5); δ_F (470 MHz, $CDCl_3$): 66.1 (SO_2F); HRMS found MNa^+ 554.0251. $C_{21}H_{23}BrFNO_7S$ requires *MNa*, 554.0251.

4-{1-[(2-Bromophenyl)methoxy]-2-oxo-2-(4-phenylpiperidin-1-yl)ethyl}benzene-1-sulfonyl fluoride (2-2)

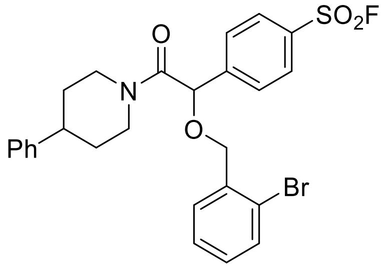

Prepared according to general procedure A – implementation of reaction array, diazo substrate **D2** (100 mM), co-substrate **C2** (500 mM) and $Rh_2(cap)_4$ (1 mM) gave a crude material. This was then purified *via* preparative HPLC eluting with gradient elution: 5:95 → 55:45 → 75:25 → 95:5 MeCN–H_2O to give the *ether derivative* ***2-2*** (1.50 mg, 14%, *rotamers* 48:52 by ^{1}H NMR) as a colourless oil. R_f 0.59 (EtOAc–hexane 50:50). ν_{max}/cm^{-1}: 3030, 2920, 2859, 1643, 1452, 1410, 1270, 1212, 1123, 1095; δ_H (500 MHz, $CDCl_3$): 8.05 (2H, d, *J* 8.4 Hz, aryl 2,6-H^{min}), 8.04 (2H, d, *J* 8.4 Hz, aryl 2,6-H^{maj}), 7.83-7.77 (4H, m, aryl 3,5-H), 7.60 (2H, dd, *J* 8.0 and 2.0 Hz, bromophenyl 3-H), 7.54-7.50 (2H, m, bromophenyl 6-H), 7.38-7.33 (2H, m, bromophenyl 5-H), 7.32-7.26 (4H, m, phenyl 3,5-H), 7.25-7.17 (4H, m, bromophenyl 4-H and phenyl 4-H), 7.11 (2H, d, *J* 7.3 Hz, phenyl 2,6-H^{maj}), 7.04 (2H, d, *J* 7.3 Hz, phenyl 2,6-H^{min}), 5.45 (2H, s, 1-H), 4.91 (1H, d, *J* 12.3 Hz, bromophenylmethoxy 1-H_A^{min}), 4.84-4.71 (5H, m, bromophenylmethoxy 1-H_A^{min}, bromophenylmethoxy 1-H_B and piperidinyl 2-H_A or piperidinyl 6-H_A), 4.21-4.10 (2H, m, piperidinyl 2-H_A or piperidinyl 6-H_A), 3.03 (1H, td, *J* 13.6 and 2.4 Hz, piperidinyl 2-H_B^{min} or piperidinyl 6-H_B^{min}), 2.80-2.59 (5H, m, piperidinyl 2-H_B^{maj} or piperidinyl 6-H_B^{maj}, piperidinyl 2-H_B or piperidinyl 6-H_B and piperidinyl 4-H), 1.98-1.85 (2H, m, piperidinyl 3-H_A or piperidinyl 5-H_A), 1.74 (1H, app. d, *J* 13.1 Hz, piperidinyl 3-H_A^{maj} or piperidinyl 5-H_A^{maj}), 1.67-1.56 (3H, m, piperidinyl 3-H_B or piperidinyl 5-H_B and piperidinyl 3-H_A^{min} or piperidinyl 5-H_A^{min}), 1.45 (1H, qd, *J* 12.7 and 4.1 Hz, piperidinyl 3-H_B^{min} or piperidinyl 5-H_B^{min}), 0.98 (1H, qd, *J* 12.7 and 4.1 Hz, piperidinyl 3-H_B^{maj} or piperidinyl 5-H_B^{maj}); δ_C (125 MHz, $CDCl_3$): 167.3 (C-2^{maj}), 167.2 (C-2^{min}), 145.5 (aryl C-4^{min}), 145.4 (aryl C-4^{min}), 144.9 (phenyl C-1^{maj}), 144.8 (phenyl C-1^{min}), 136.1 (bromophenyl C-1), 132.8 (d, *J* 24.6 Hz, aryl C-1^{min}), 132.7 (d, *J* 24.6 Hz, aryl C-1^{maj}), 130.2 (bromophenyl C-3), 130.02 (bromophenyl C-6^{maj}), 129.99 (bromophenyl C-6^{min}), 129.0 (aryl C_2-2,6^{maj}), 128.9 (aryl C_2-2,6^{min}), 128.8 (phenyl C_2-3,5), 127.8 (bromophenyl C-5), 127.5 (aryl C_2-3,5^{min}), 127.3 (aryl C_2-3,5^{maj}), 126.7

(phenyl C_2-2,6), 126.7 (phenyl C-4), 123.7 (bromophenyl C-2^{maj}), 123.5 (bromophenyl C-2^{min}), 82.4 (C-1^{min}), 82.0 (C-1^{maj}), 72.4 (bromophenylmethoxy C-1^{maj}), 72.1 (bromophenylmethoxy C-1^{min}), 45.9 (piperidinyl C_A-2,6^{maj}), 45.8 (piperidinyl C_A-2,6^{min}), 43.73 (piperidinyl C_B-2,6^{maj}), 43.67 (piperidinyl C_B-2,6^{min}), 42.5 (piperidinyl C-4), 33.5 (piperidinyl C_A-3,5^{min}), 33.3 (piperidinyl C_A-3,5^{maj}), 33.3 (piperidinyl C_B-3,5^{maj}), 32.9 (piperidinyl C_B-3,5^{min}); δ_F (470 MHz, $CDCl_3$): 66.2 (SO_2F^{min}), 66.1 (SO_2F^{maj}); HRMS found MH^+ 546.0743. $C_{26}H_{25}BrFNO_4S$ requires *MH*, 546.0744.

4-{1-[(2-Chloro-5-fluorophenyl)methoxy]-2-oxo-2-(4-phenylpiperidin-1-yl)ethyl}benzene-1-sulfonyl fluoride (2-5)

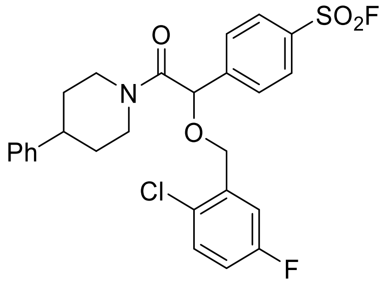

Prepared according to general procedure A – implementation of reaction array, diazo substrate **D2** (100 mM), co-substrate **C5** (500 mM) and $Rh_2(cap)_4$ (1 mM) gave a crude material. This was then purified via preparative HPLC eluting with gradient elution: 5:95 → 55:45 → 75:25 → 95:5 MeCN–H_2O to give the *ether derivative* ***2-5*** (1.40 mg, 14%, *rotamers* 51:49 by ^{1}H NMR) as a colourless oil. R_f 0.66 (EtOAc–hexane 50:50). ν_{max}/cm^{-1}: 3062, 2921, 2858, 1644, 1452, 1411, 1269, 1212, 1096; δ_H (500 MHz, $CDCl_3$): 8.06 (4H, d, *J* 7.1 Hz, aryl 2,6-H), 7.79 (4H, app. t, *J* 9.3 Hz, aryl 3,5-H), 7.39-7.34 (2H, m, chlorophenyl 3-H), 7.32-7.25 (6H, m, chlorophenyl 6-H and phenyl 3,5-H), 7.24-7.19 (2H, m phenyl 4-H), 7.10 (2H, d, *J* 7.4 Hz, phenyl 2,6-H^{maj}), 7.06 (2H, d, *J* 7.4 Hz, phenyl 2,6-H^{min}), 7.03-6.96 (2H, m, chlorophenyl 4-H), 5.46 (2H, s, 1-H), 4.89 (1H, d, *J* 12.9 Hz, chlorophenylmethoxy 1-H_A^{min}), 4.83-4.69 (5H, m, chlorophenylmethoxy 1-H_A^{maj}, chlorophenylmethoxy 1-H_B and piperidinyl 2-H_A or piperidinyl 6-H_A), 4.14 (2H, app. t, *J* 12.7 Hz, piperidinyl 2-H_A or piperidinyl 6-H_A), 3.04 (1H, td, *J* 13.7 and 2.2 Hz, piperidinyl 2-H_B^{min} or piperidinyl 6-H_B^{min}), 2.80 (1H, td, *J* 13.7 and 2.2 Hz, piperidinyl 2-H_B^{maj} or piperidinyl 6-H_B^{maj}), 2.75-2.63 (4H, m, piperidinyl 2-H_B or piperidinyl 6-H_B and piperidinyl 4-H), 1.93 (2H, app. t, *J* 15.4 Hz, piperidinyl 3-

H_A or piperidinyl 5-H_A), 1.77 (1H, app. d, *J* 13.0 Hz, piperidinyl 3-H_A^{maj} or piperidinyl 5-H_A^{maj}), 1.69-1.55 (3H, m, piperidinyl 3-H_A^{min} or piperidinyl 5-H_A^{min} and piperidinyl 3-H_B or piperidinyl 5-H_B), 1.39 (1H, qd, *J* 12.7 and 4.3 Hz, piperidinyl 3-H_B^{maj} or piperidinyl 5-H_B^{maj}), 1.02 (1H, qd, *J* 12.7 and 4.3 Hz, piperidinyl 3-H_B^{min} or piperidinyl 5-H_B^{min}); δ_C (125 MHz, $CDCl_3$): 167.0 (C-2^{maj}), 166.9 (C-2^{min}), 161.6 (d, *J* 247.1 Hz, chlorophenyl C-5), 145.1 (aryl C-4^{maj}), 145.0 (aryl C-4^{min}), 144.8 (phenyl C-1^{maj}), 144.7 (phenyl C-1^{min}), 136.7 (d, *J* 7.6 Hz, chlorophenyl C-1), 133.0 (d, *J* 24.9 Hz, aryl C-1), 131.0 (d, *J* 8.2 Hz, chlorophenyl C-3), 129.1 (aryl C_2-2,6^{min}), 129.0 (aryl C_2-2,6^{maj}), 128.8 (phenyl C_2-3,5), 127.9 (d, *J* 2.9 Hz, chlorophenyl C-2^{min}), 127.7 (d, *J* 2.9 Hz, chlorophenyl C-2^{maj}), 127.5 (aryl C_2-3,5^{maj}), 127.4 (aryl C_2-3,5^{min}), 126.8 (phenyl C_2-2,6), 126.72 (phenyl C-4^{min}), 126.68 (phenyl C-4^{maj}), 116.5 (d, *J* 22.9 Hz, chlorophenyl C-4), 116.4 (d, *J* 21.5 Hz, chlorophenyl C-6^{min}), 116.2 (d, *J* 21.5 Hz, chlorophenyl C-6^{maj}), 82.5 (C-1^{min}), 82.1 (C-1^{maj}), 69.5 (chlorophenylmethoxy C-1^{maj}), 69.2 (chlorophenylmethoxy C-1^{min}), 45.9 (piperidinyl C_A-2,6^{min}), 45.8 (piperidinyl C_A-2,6^{maj}), 43.8 (piperidinyl C_B-2,6^{maj}), 43.7 (piperidinyl C_B-2,6^{min}), 42.5 (piperidinyl C-4), 33.5 (piperidinyl C_A-3,5^{maj}), 33.3 (piperidinyl C_A-3,5^{min}), 33.2 (piperidinyl C_B-3,5^{min}), 32.9 (piperidinyl C_B-3,5^{maj}); δ_F (470 MHz, $CDCl_3$): 66.2 (SO_2F^{maj}), 66.1 (SO_2F^{min}), -114.2 (chlorophenyl CF^{min}), -114.3 (chlorophenyl CF^{maj}); HRMS found MH^+ 520.1160. $C_{26}H_{24}ClF_2NO_4S$ requires *MH*, 520.1155.

4-[1-(4-Chloro-2,5-dimethylbenzenesulfonamido)-2-oxo-2-(4-phenylpiperidin-1-yl)ethyl]benzene-1-sulfonyl fluoride (2-6)

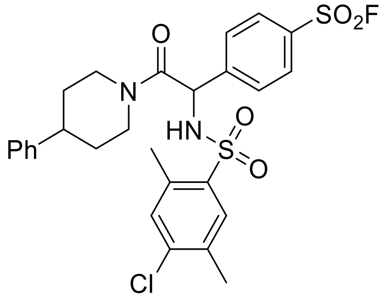

Prepared according to general procedure A – implementation of reaction array, diazo substrate **D2** (100 mM), co-substrate **C6** (500 mM) and $Rh_2(cap)_4$ (1 mM) gave a crude material. This was then purified via preparative HPLC eluting with gradient elution: 5:95 → 55:45 → 75:25 → 95:5 MeCN–H_2O to give the *sulfonamide derivative* ***2-6*** (1.20

mg, 10%, *rotamers* 43:57 by ^{1}H NMR) as a colourless oil. R_f 0.60 (EtOAc–hexane 50:50). ν_{max}/cm^{-1}: 3234, 3031, 2924, 2860, 1644, 1453, 1413, 1270, 1214, 1161, 1089; δ_H (500 MHz, $CDCl_3$): 7.90 (2H, d, *J* 8.5 Hz, aryl 2,6-H^{maj}), 7.89 (2H, d, *J* 8.5 Hz, aryl 2,6-H^{min}), 7.64 (1H, s, sulfonamidophenyl 6-H^{maj}), 7.56 (1H, s, sulfonamidophenyl 6-H^{min}), 7.52-7.48 (4H, m, aryl 3,5-H), 7.34 (2H, app. t, *J* 7.6 Hz, phenyl 3,5-H^{maj}), 7.26-7.21 (4H, m, phenyl 3,5-H^{min} and phenyl 4-H), 7.17 (2H, s, sulfonamidophenyl 3-H), 7.11 (2H, d, *J* 7.6 Hz, phenyl 2,6-H^{maj}), 6.92 (2H, d, *J* 7.6 Hz, phenyl 2,6-H^{min}), 6.54 (2H, app. t, *J* 7.2 Hz, NH), 5.34 (1H, d, *J* 7.2 Hz, 1-H^{min}), 5.31 (1H, d, *J* 7.2 Hz, 1-H^{maj}), 4.67-4.61 (2H, m, piperidinyl 2-H_A or piperidinyl 6-H_A), 3.72-3.64 (2H, m, piperidinyl 2-H_A or piperidinyl 6-H_A), 3.05 (1H, td, *J* 13.2 and 2.6 Hz, piperidinyl 2-H_B^{min} or piperidinyl 6-H_B^{min}), 2.78 (1H, td, *J* 13.2 and 2.6 Hz, piperidinyl 2-H_B^{maj} or piperidinyl 6-H_B^{maj}), 2.69-2.59 (4H, m, piperidinyl 2-H_B or piperidinyl 6-H_B and piperidinyl 4-H), 2.58 (3H, s, sulfonamidophenyl 2-methyl^{maj}), 2.52 (3H, s, sulfonamidophenyl 2-methyl^{min}), 2.30 (3H, s, sulfonamidophenyl 5-methyl^{min}), 2.26 (3H, s, sulfonamidophenyl 5-methyl^{maj}), 1.92-1.81 (3H, m, piperidinyl 3-H_A or piperidinyl 5-H_A and piperidinyl 3-H_A^{maj} or piperidinyl 5-H_A^{maj}), 1.61-1.56 (1H, m, piperidinyl 3-H_A^{min} or piperidinyl 5-H_A^{min}) 1.48-1.20 (3H, m, piperidinyl 3-H_B or piperidinyl 5-H_B and piperidinyl 3-H_B^{maj} or piperidinyl 5-H_B^{maj}), 0.56 (1H, qd, *J* 12.7 and 4.2 Hz, piperidinyl 3-H_B^{min} or piperidinyl 5-H_B^{min}); δ_C (125 MHz, $CDCl_3$): 165.6 (C-2), 144.4 (aryl C-4^{min}), 144.1 (aryl C-4^{maj}), 144.0 (phenyl C-1^{maj}), 143.9 (phenyl C-1^{min}), 139.3 (sulfonamidophenyl C-1^{maj}), 139.2 (sulfonamidophenyl C-1^{min}), 136.5 (sulfonamidophenyl C-2^{maj}), 136.4 (suldonamidophenyl C-2^{min}), 136.2 (sulfonamidophenyl C-4^{maj}), 136.1 (sulfonamidophenyl C-4^{min}), 134.0 (sulfonamidophenyl C-5^{maj}), 133.9 (sulfonamidophenyl C-5^{min}), 133.3 (d, *J* 25.4 Hz, aryl C-1), 133.0 (sulfonamidophenyl C-3^{maj}), 132.8 (sulfonamidophenyl C-3^{min}), 131.5 (sulfonamidophenyl C-6^{maj}), 131.3 (sulfonamidophenyl C-6^{min}), 129.14 (aryl C_2-2,6), 129.06 (aryl C_2-3,5), 129.0 (phenyl C_2-3,5^{maj}), 128.8 (phenyl C_2-3,5^{min}), 127.02 (phenyl C-4^{maj}), 126.99 (phenyl C-4^{min}), 126.7 (phenyl C_2-2,6^{maj}), 126.5 (phenyl C_2-2,6^{min}), 56.7 (C-1^{min}), 56.4 (C-1^{maj}), 46.4 (piperidinyl C_A-2,6^{maj}), 46.0 (piperidinyl C_A-2,6^{min}), 43.9 (piperidinyl C_B-2,6), 42.4 (piperidinyl C-4^{maj}), 42.0 (piperidinyl C-4^{min}), 33.8 (piperidinyl C_A-3,5), 32.6 (piperidinyl C_B-3,5^{maj}), 32.4 (piperidinyl C_B-3,5^{min}), 19.7 (sulfonamidophenyl 2-methyl^{maj}), 19.6 (sulfonamidophenyl 2-methyl^{min}), 19.5 (sulfonamidophenyl 5-methyl); δ_F (470 MHz, $CDCl_3$): 66.2 (SO_2F^{min}), 66.0 (SO_2F^{maj}); HRMS found MH^+ 579.1183. $C_{27}H_{28}ClFN_2O_5S_2$ requires *MH*, 579.1185.

4-{1-[(4-Bromo-3,5-dimethoxyphenyl)methoxy]-2-oxo-2-(4-phenylpiperidin-1-yl)ethyl}benzene-1-sulfonyl fluoride (2-8)

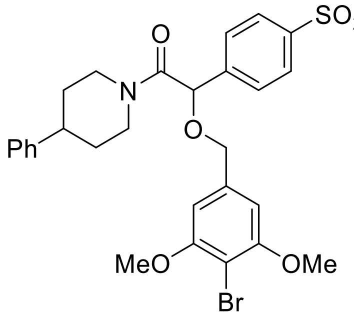

Prepared according to general procedure A – implementation of reaction array, diazo substrate **D2** (100 mM), co-substrate **C8** (500 mM) and $Rh_2(cap)_4$ (1 mM) gave a crude material. This was then purified via preparative HPLC eluting with gradient elution: 5:95 → 55:45 → 75:25 → 95:5 MeCN–H_2O to give the *ether derivative **2-8*** (1.60 mg, 13%, *rotamers* 48:52 by ^{1}H NMR) as a colourless oil. R_f 0.31 (EtOAc–hexane 50:50). ν_{max}/cm^{-1}: 2938, 2861, 1645, 1590, 1494, 1455, 1413, 1213, 1124, 1095; δ_H (500 MHz, $CDCl_3$): 8.05 (2H, d, *J* 8.1 Hz, aryl 2,6-H^{min}), 8.04 (2H, d, *J* 8.1 Hz, aryl 2,6-H^{maj}), 7.77 (2H, d, *J* 8.1 Hz, aryl 3,5-H^{maj}), 7.75 (2H, d, *J* 8.1 Hz, aryl 3,5-H^{min}), 7.32-7.26 (4H, m, phenyl 3,5-H), 7.24-7.18 (2H, m, phenyl 4-H), 7.08 (2H, d, *J* 7.3 Hz, phenyl 2,6-H^{min}), 7.04 (2H, d, *J* 7.3 Hz, phenyl 2,6-H^{maj}), 6.62 (2H, s, dimethoxyphenyl 2,6-H^{maj}), 6.59 (2H, s, dimethoxyphenyl 2,6-H^{min}), 5.39 (2H, s, 1-H), 4.81-4.61 (6H, m, dimethoxyphenylmethoxy 1-H_2 and piperidinyl 2-H_A or piperidinyl 6-H_A), 4.15-4.07 (2H, m, piperidinyl 2-H_A or piperidinyl 6-H_A), 3.89 (6H, s, OMe^{min}), 3.88 (6H, s, OMe^{maj}), 2.98 (1H, td, *J* 13.1 and 2.1 Hz, piperidinyl 2-H_B^{min} or piperidinyl 6-H_B^{min}), 2.77-2.61 (5H, m, piperidinyl 2-H_B^{maj} or piperidinyl 6-H_B^{maj}, piperidinyl 2-H_B or piperidinyl 6-H_B and piperidinyl 4-H), 1.92 (2H, app. t, *J* 14.8 Hz, piperidinyl 3-H_A or piperidinyl 5-H_A), 1.75 (1H, app. d, *J* 13.4 Hz, piperidinyl 3-H_A^{maj} or piperidinyl 5-H_A^{maj}), 1.64 (1H, app. d, *J* 13.4 Hz, piperidinyl 3-H_A^{min} or piperidinyl 5-H_A^{min}), 1.59-1.48 (2H, m, piperidinyl 3-H_B or piperidinyl 5-H_B), 1.37 (1H, qd, *J* 12.8 and 4.2 Hz, piperidinyl 3-H_B^{maj} or piperidinyl 5-H_B^{maj}), 1.00 (1H, qd, *J* 12.8 and 4.2 Hz, piperidinyl 3-H_B^{min} or piperidinyl 5-H_B^{min}); δ_C (125 MHz, $CDCl_3$): 167.3 (C-2^{min}), 167.1 (C-2^{maj}), 157.5 (dimethoxyphenyl C_2-3,5^{maj}), 157.4 (dimethoxyphenyl C_2-3,5^{min}), 145.4 (aryl C-4), 144.7 (phenyl C-1^{maj}), 144.6 (phenyl C-1^{min}), 137.3 (dimethoxyphenyl C-1^{maj}), 137.2 (dimethoxyphenyl C-1^{min}), 132.8 (d, *J* 25.9 Hz, aryl C-1), 129.02 (aryl C_2-2,6^{maj}), 128.95 (aryl C_2-2,6^{min}), 128.8 (phenyl C_2-3,5), 127.6 (aryl C_2-3,5^{maj}), 127.2 (aryl C_2-3,5^{min}), 126.9 (phenyl C-4), 126.6

(phenyl C_2-2,6), 104.6 (dimethoxyphenyl C_2-2,6), 101.0 (dimethoxyphenyl C-4), 82.0 (C-1^{maj}), 81.0 (C-1^{min}), 73.0 (dimethoxyphenylmethoxy C-1^{maj}), 72.5 (dimethoxyphenylmethoxy C-1^{min}), 56.7 (OMe), 45.8 (piperidinyl C_A-2,6), 43.7 (piperidinyl C_B-2,6^{min}), 43.6 (piperidinyl C_B-2,6^{min}), 42.5 (piperidinyl C-4^{min}), 42.4 (piperidinyl C-4^{maj}), 33.7 (piperidinyl C_A-3,5^{maj}), 33.5 (piperidinyl C_A-3,5^{min}), 33.0 (piperidinyl C_B-3,5^{maj}), 32.9 (piperidinyl C_B-3,5^{min}); δ_F (470 MHz, $CDCl_3$): 66.2 (SO_2F^{min}), 66.1 (SO_2F^{maj}); HRMS found MH^+ 606.0953. $C_{28}H_{29}BrFNO_6S$ requires *MH*, 606.0956.

4-[1-{[(1*R*,5*R*)-2-Methyl-5-(prop-1-en-2-yl)cyclohex-2-en-1-yl]oxy}-2-oxo-2-(4-phenylpiperidin-1-yl)ethyl]benzene-1-sulfonyl fluoride (2-14)

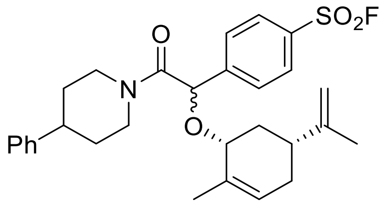

Prepared according to general procedure A – implementation of reaction array, diazo substrate **D2** (100 mM), co-substrate **C14** (500 mM) and $Rh_2(cap)_4$ (1 mM) gave a crude material. This was then purified via preparative HPLC eluting with gradient elution: 5:95 → 55:45 → 75:25 → 95:5 MeCN–H_2O to give the *ether derivative* ***2-14*** (1.00 mg, 10%, *dr* 51:49 by ^1H NMR) as a colourless oil. R_f 0.78 (EtOAc–hexane 50:50). ν_{max}/cm^{-1}: 3029, 2921, 2859, 1639, 1493, 1452, 1410, 1269, 1212, 1124, 1095; δ_H (500 MHz, $CDCl_3$): 8.08-7.98 (4H, m, aryl 2,6-H), 7.86-7.74 (4H, m, aryl 3,5-H), 7.32-7.17 (6H, m, phenyl 3,5-H and phenyl 4-H), 7.14 (2H, d, *J* 7.2 Hz, phenyl 2,6-H^{maj}), 7.02-6.96 (2H, m, phenyl 2,6-H^{min}), 5.78-5.50 (2H, m, cyclohexenyl 3-H), 5.46 (1H, s, 1-H^{min}), 5.42 (1H, s, 1-H^{maj}), 4.82-4.65 (6H, m, propenyl 1-H_2 and piperidinyl 2-H_A or piperidinyl 6-H_A), 4.42-4.14 (4H, m, piperidinyl 2-H_A or piperidinyl 6-H_A and cyclohexenyl 1-H), 3.13-2.94 (1H, m, piperidinyl 2-H_B^{maj} or piperidinyl 6-H_B^{maj}), 2.74-2.54 (5H, m, piperidinyl 2-H_B^{min} or piperidinyl 6-H_B^{min}, piperidinyl 2-H_B or piperidinyl 6-H_B and piperidinyl 4-H), 2.43-2.18 (4H, m, cyclohexenyl 5-H and piperidinyl 3-H_A or piperidinyl 5-H_A), 2.15-1.50 (25H, m, piperidinyl 3-H_A or piperidinyl 5-H_A cyclohexenyl 4,6-H_2, cyclohexenyl 2-methyl, propenyl 3-H_3, piperidinyl 3-H_B or piperidinyl 5-H_B and piperidinyl 3-H_B^{maj} or piperidinyl 5-H_B^{maj}), 0.73 (1H, qd, *J* 12.8 and 4.2 Hz, piperidinyl

3-H_B^{min} or piperidinyl 5-H_B^{min}); δ_C (125 MHz, $CDCl_3$): 169.2 (C-2^{maj}), 168.8 (C-2^{min}), 148.6 (propenyl C-2^{maj}), 148.5 (propenyl C-2^{min}), 146.6 (aryl C-4), 145.1 (phenyl C-1^{maj}), 144.8 (phenyl C-1^{min}), 134.4 (cyclohexenyl C-2^{maj}), 134.3 (cyclohexenyl C-2^{min}), 132.4 (d, *J* 24.9 Hz, aryl C-1), 128.9 (aryl C_2-2,6^{min}), 128.84 (aryl C_2-2,6^{maj}), 128.76 (phenyl-3,5), 126.83 (aryl C_2-3,5^{min}), 126.76 (aryl C_2-3,5^{maj}), 126.7 (phenyl C_2-2,6^{maj}), 126.63 (phenyl C_2-2,6^{min}), 126.58 (phenyl C-4^{maj}), 126.5 (phenyl C-4^{min}), 126.0 (cyclohexenyl C-3^{maj}), 125.9 (cyclohexenyl C-3^{min}), 109.7 (propenyl C-1^{maj}), 109.6 (propenyl C-1^{min}), 83.5 (C-1^{maj}), 83.2 (C-1^{min}), 81.3 (cyclohexenyl C-1^{min}), 80.8 (cyclohexenyl C-1^{maj}), 46.1 (piperidinyl C_A-2,6^{min}), 45.6 (piperidinyl C_A-2,6^{maj}), 43.8 (piperidinyl C_B-2,6^{maj}), 43.5 (piperidinyl C_B-2,6^{min}), 42.5 (piperidinyl C-4), 40.7 (cyclohexenyl C-5^{min}), 40.6 (cyclohexenyl C-5^{maj}), 35.7 (piperidinyl C_A-3,5^{maj}), 34.8 (piperidinyl C_A-3,5^{min}), 33.8 (cyclohexenyl C-6), 33.1 (piperidinyl C_B-3,5^{min}), 32.9 (piperidinyl C_B-3,5^{maj}), 31.1 (cyclohexenyl C-4^{maj}), 31.0 (cyclohexenyl C-4^{maj}), 20.7 (propenyl C-3^{maj}), 20.6 (propenyl C-3^{min}), 19.9 (cyclohexenyl 2-methyl^{maj}), 19.8 (cyclohexenyl 2-methyl^{min}); δ_F (470 MHz, $CDCl_3$): 66.3 (SO_2F^{min}), 66.1 (SO_2F^{maj}); HRMS found MNa^+ 534.2077. $C_{29}H_{34}FNO_4S$ requires *MNa*, 534.2085.

4-[1-(5-Methoxy-1*H*-indol-3-yl)-2-oxo-2-(4-phenylpiperidin-1-yl)ethyl]benzene-1-sulfonyl fluoride (2-15a)

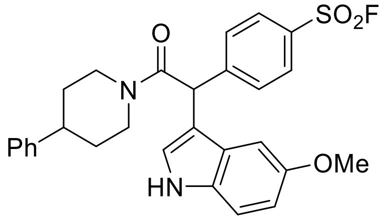

Prepared according to general procedure A – implementation of reaction array, diazo substrate **D2** (100 mM), co-substrate **C15** (500 mM) and $Rh_2(cap)_4$ (1 mM) gave a crude material. This was then purified via preparative HPLC eluting with gradient elution: 5:95 → 55:45 → 75:25 → 95:5 MeCN–H_2O to give the *indole derivative* ***2-15a*** (1.10 mg, 11%, *rotamers* 51:49 by ^{1}H NMR) as a colourless oil. R_f 0.48 (EtOAc–hexane 50:50). ν_{max}/cm^{-1}: 3416, 2925, 1637, 1484, 1440, 1410, 1212, 1166; δ_H (500 MHz, $CDCl_3$): 9.10 (1H, br. s, NH^{maj}), 9.04 (1H, br. s, NH^{min}), 7.96 (2H, d, *J* 8.5 Hz, aryl 2,6-H^{min}), 7.94 (2H, d, *J* 8.5 Hz, aryl 2,6-H^{maj}), 7.58 (2H, d, *J* 8.5 Hz, aryl 3,5-H^{min}), 7.54 (2H, d, *J* 8.5 Hz, aryl 3,5-H^{maj}), 7.35-7.30 (2H, m, phenyl 3,5-H^{maj}), 7.29-7.17 (6H, m,

phenyl 3,5-H^{min}, phenyl 4-H and indolyl 7-H), 7.15 (2H, d, *J* 7.4 Hz, phenyl 2,6-H^{min}), 7.08 (2H, d, *J* 7.4 Hz, phenyl 2,6-H^{maj}), 7.04 (1H, d, *J* 2.5 Hz, indolyl 4-H^{maj}), 7.03 (1H, d, *J* 2.5 Hz, indolyl 4-H^{min}), 6.89-6.84 (2H, m, indolyl 6-H), 6.40 (1H, d, *J* 1.5 Hz, indolyl 2-H^{maj}), 6.37 (1H, d, *J* 1.5 Hz, indolyl 2-H^{min}), 5.66 (1H, s, 1-H^{maj}), 5.64 (1H, s, 1-H^{min}), 4.89-4.82 (2H, m piperidinyl 2-H_A or piperidinyl 6-H_A), 4.29-4.18 (2H, m, piperidinyl 2-H_A or piperidinyl 6-H_A), 3.84 (6H, s, OMe), 3.26 (2H, app. tt, *J* 13.5 and 2.5 Hz, piperidinyl 2-H_B or piperidinyl 6-H_B), 2.83-2.70 (4H, m, piperidinyl 2-H_B or piperidinyl 6-H_B and piperidinyl 4-H), 2.04-1.82 (4H, m piperidinyl 3,5-H_A), 1.73-1.56 (2H, m, piperidinyl 3-H_B or piperidinyl 5-H_B), 1.53-1.39 (2H, m, piperidinyl 3-H_B or piperidinyl 5-H_B); δ_C (125 MHz, $CDCl_3$): 168.6 (C-2), 154.6 (indolyl C-5), 147.7 (aryl C-4^{maj}), 147.6 (aryl C-4^{min}), 144.6 (phenyl C-1^{min}), 144.5 (phenyl C-1^{maj}), 134.4 (indolyl C-$3a^{min}$), 134.3 (indolyl C-$3a^{maj}$), 132.0 (d, J 24.8 Hz, aryl C-1^{maj}), 131.9 (d, J 24.8 Hz, aryl C-1^{min}), 131.80 (indolyl C-$7a^{min}$), 131.77 (indolyl C-$7a^{maj}$), 129.5 (aryl C_2-3,5^{min}), 129.4 (aryl C_2-3,5^{maj}), 129.1 (aryl C_2-2,6^{min}), 129.0 (aryl C_2-2,6^{maj}), 128.9 (phenyl C_2-3,5^{min}), 128.8 (phenyl C_2-3,5^{maj}), 128.5 (indolyl C-3^{min}), 128.4 (indolyl C-3^{maj}), 127.0 (phenyl C-4^{min}), 126.84 (phenyl C-4^{maj}), 126.80 (phenyl C_2-2,6^{maj}), 126.7 (phenyl C_2-2,6-H^{min}), 112.83 (indolyl C-6^{maj}), 112.79 (indolyl C-6^{min}), 112.2 (indolyl C-7), 102.5 (indolyl C-2^{min}), 102.4 (indolyl C-2^{maj}), 102.31 (indolyl C-4^{maj}), 102.28 (indolyl C-4^{min}), 56.0 (OMe), 47.6 (piperidinyl C_A-2,6^{maj}), 47.4 (piperidinyl C_A-2,6^{min}), 47.0 (C-1^{min}), 46.9 (C-1^{maj}), 43.7 (piperidinyl C_B-2,6^{maj}), 43.4 (piperidinyl C_B-2,6^{min}), 42.7 (piperidinyl C-4^{maj}), 42.5 (piperidinyl C-4^{min}), 34.1 (piperidinyl C_A-3,5^{maj}), 33.8 (piperidinyl C_A-3,5^{min}), 33.0 (piperidinyl C_B-3,5^{maj}), 32.9 (piperidinyl C_B-3,5^{min}); δ_F (470 MHz, $CDCl_3$): 66.22 (SO_2F^{min}), 66.16 (SO_2F^{maj}); HRMS found MH^+ 507.1755. $C_{28}H_{27}FN_2O_4S$ requires *MH*, 507.1748.

Also obtained was the *indole derivative* 4-[1-(5-methoxy-1H-indol-1-yl)-2-oxo-2-(4-phenylpiperidin-1-yl)ethyl]benzene-1-sulfonyl fluoride ***2-15b*** (0.10 mg, 1%, *rotamers* 65:35 by ^{1}H NMR) as a colourless oil. R_f 0.43 (EtOAc–hexane 50:50). ν_{max}/cm^{-1}: 2936, 2852, 1640, 1596, 1453, 1412, 1213, 1166, 1099; δ_H (500 MHz, $CDCl_3$): 7.98-7.92 (4H, m, aryl C_2-2,6), 7.34-7.27 (4H, m, aryl C_2-3,5), 7.25-7.22 (6H, m, phenyl 3,5-H and indolyl 2-H), 7.22 (8H, phenyl 2,6-H^{min} phenyl 4-H and indolyl 4,7-H), 6.93 (2H, d, *J* 7.1 Hz, phenyl C_2-2,6^{maj}), 6.90-6.84 (2H, m, indolyl 6-H), 6.63 (1H, dd, *J* 3.2 and 0.7 Hz, indolyl 3-H^{maj}), 6.49 (1H, dd, *J* 3.2 and 0.7 Hz, indolyl 3-H^{min}), 6.47 (2H, s, 1-H), 4.89-4.79 (2H, m, piperidinyl 2-H_A or piperidinyl 6-H_A), 3.86 (3H, s, OMe^{maj}), 3.85 (3H,

s, OMe^{min}), 3.85-3.74 (2H, m piperidinyl 2-H_A or piperidinyl 6-H_A), 3.19 (1H, td, *J* 13.1 and 2.6 Hz, piperidinyl 2-H_B^{maj} or piperidinyl 6-H_B^{maj}), 2.91 (1H, td, *J* 13.1 and 2.6 Hz, piperidinyl 2-H_B^{min} or piperidinyl 6-H_B^{min}), 2.86-2.62 (4H, m, piperidinyl 2-H_B or piperidinyl 6-H_B and piperidinyl 4-H), 2.03-1.96 (1H, m, piperidinyl 3-H_A^{min} or piperidinyl 6-H_A^{min}), 1.91-1.82 (2H, m, piperidinyl 3-H_A or piperidinyl 6-H_A), 1.77-1.53 (2H, m, piperidinyl 3-H_A^{maj} or piperidinyl 6-H_A^{maj}, piperidinyl 3-H_B^{min} or piperidinyl 6-H_B^{min}), 1.50-1.43 (2H, m, piperidinyl 3-H_B or piperidinyl 6-H_B), 0.70 (1H, qd, *J* 12.8 and 4.2 Hz, piperidinyl 3-H_B^{maj} or piperidinyl 6-H_B^{maj}); δ_C (125 MHz, $CDCl_3$): 165.3 (C-2), 155.0 (indolyl C-5), 145.7 (aryl C-4^{min}), 145.5 (aryl C-4^{min}), 144.64 (phenyl C-1^{min}), 144.56 (phenyl C-1^{maj}), 130.9 (indolyl C-7a), 129.7 (indolyl C-$3a^{min}$), 129.6 (indolyl C-$3a^{maj}$), 128.89 (aryl C_2-2,6^{min}), 128.85 (aryl C_2-2,6^{maj}), 128.8 (aryl C_2-3,5^{min}), 128.72 (aryl C_2-3,5^{maj}), 128.69 (indolyl C-2), 127.2 (phenyl C_2-3,5), 126.92 (phenyl C-4^{min}), 126.9 (phenyl C-4^{maj}), 126.8 (phenyl C_2-2,6), 113.2 (indolyl C-6^{maj}), 113.1 (indolyl C-6^{min}), 110.0 (indolyl C-7), 103.8 (indolyl C-3^{maj}), 103.6 (indolyl C-3^{min}), 103.5 (indolyl C-4^{maj}), 103.4 (indolyl C-4^{min}), 60.3 (C-1^{maj}), 60.2 (C-1^{min}), 56.0 (OMe^{maj}), 55.9 (OMe^{min}), 47.3 (piperidinyl C_A-2,6), 44.1 (piperidinyl C_B-2,6^{maj}), 43.6 (piperidinyl C_B-2,6^{min}), 42.6 (piperidinyl C-4^{maj}), 42.3 (piperidinyl C-4^{min}), 33.8 (piperidinyl C_A-3,5), 33.2 (piperidinyl C_B-3,5^{min}), 32.8 (piperidinyl C_B-3,5^{maj}); δ_F (470 MHz, $CDCl_3$): 66.1 (SO_2F); HRMS found MH^+ 507.1751. $C_{28}H_{27}FN_2O_4S$ requires *MH*, 507.1748. Aryl C-1 not observed by ^{13}C NMR (125 MHz).

4-[2-(2,3-Dihydro-1*H*-isoindol-2-yl)-1-(naphthalen-1-yloxy)-2-oxoethyl]benzene-1-sulfonyl fluoride (3-4a) and 4-[2-(2,3-dihydro-1*H*-isoindol-2-yl)-1-(1-hydroxynaphthalen-2-yl)-2-oxoethyl]benzene-1-sulfonyl fluoride (3-4b)

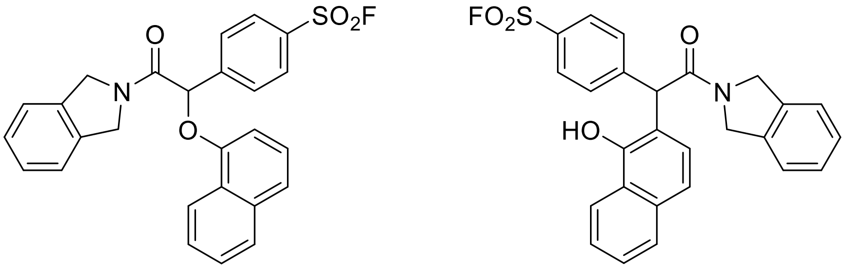

Prepared according to general procedure A – implementation of reaction array, diazo substrate **D3** (100 mM), co-substrate **C4** (500 mM) and $Rh_2(cap)_4$ (1 mM) gave a crude material. This was then purified via preparative HPLC eluting with gradient elution:

5:95 → 55:45 → 75:25 → 95:5 MeCN–H_2O to give the *naphthalene derivative* ***3-4a*** and ***3-4b*** (0.50 mg, 5%, *regioisomers* 51:49 by ^{1}H NMR) as a colourless oil. R_f 0.63 (EtOAc–hexane 50:50). ν_{max}/cm^{-1}: 3061, 2925, 2866, 1642, 1590, 1441, 1410, 1265, 1213, 1099; δ_H (500 MHz, $CDCl_3$): 10.72 (1H, br. s, OH^{min}), 8.48-8.43 (1H, m, napthanenyl 8-H^{min}), 8.35-8.32 (1H, m, napthanenyl 8-H^{maj}), 8.12 (2H, d, *J* 8.7 Hz, phenyl 2,6-H^{maj}), 8.07 (2H, d, *J* 8.7 Hz, napthanenyl 3,5-H^{maj}), 7.92 (2H, d, *J* 8.6 Hz, phenyl 2,6-H^{min}), 7.88-7.85 (1H, m, napthanenyl 5-H^{maj}), 7.81-7.78 (1H, m, napthanenyl 5-H^{min}), 7.65-7.27 (8H, m, phenyl 3,5-H^{min} and napthanenyl 4,6,7-H), 7.40-7.18 (9H, m, napthanenyl 3-H^{maj} and isoindolyl 4,5,6,7-H), 7.09 (1H, d, *J* 7.4 Hz, napthanenyl 3-H^{min}), 6.99 (1H, d, *J* 7.6 Hz, napthanenyl 2-H^{maj}), 6.21 (1H, s, 1-H^{maj}), 5.40 (1H, s, 1-H^{min}), 5.18 (2H, s, isoindolyl 1-H_2^{maj} or isoindolyl 3-H_2^{maj}), 4.08-4.72 (6H, m, isoindolyl 1-H_2^{min} or isoindolyl 3-H_2^{min} and isoindolyl 1,3-H_2); δ_C (125 MHz, $CDCl_3$): 172.7 (C-2^{min}), 167.3 (C-2^{maj}), 153.4 (napthanenyl C-1^{min}), 152.1 (napthanenyl C-1^{maj}), 146.1 (phenyl C-4^{min}), 143.4 (phenyl C-4^{maj}), 136.1 (napthanenyl C-4^{maj}), 135.6 (napthanenyl C-4^{min}), 135.00 (isoindolyl C_A-3a,7a^{maj}), 134.96 (isoindolyl C_A-3a,7a^{min}), 134.9 (isoindolyl C_B-3a,7a^{min}), 134.8 (isoindolyl C_B-3a,7a^{maj}), 133.3 (d, *J* 25.0 Hz, phenyl C-1^{maj}), 132.0 (d, *J* 24.8 Hz, phenyl C-1^{min}), 129.2 (aryl C-H), 129.13 (aryl C-H), 129.09 (aryl C-H), 128.9 (aryl C-H), 128.7 (aryl C-H), 128.2 (aryl C-H), 128.14 (aryl C-H), 128.05 (aryl C-H), 127.8 (aryl C-H), 127.6 (aryl C-H), 127.29 (aryl C-H), 127.27 (aryl C-H), 127.1 (aryl C-H), 126.3 (aryl C-H), 125.9 (aryl C-H), 125.8 (aryl C-H), 125.4 (aryl C_q), 123.3 (aryl C-H), 123.2 (aryl C-H), 122.9 (aryl C-H), 122.8 (aryl C-H), 122.7 (aryl C-H), 122.5 (aryl C-H), 121.4 (aryl C-H), 120.3 (aryl C-H), 114.5 (aryl C_q), 106.5 (napthanenyl C-2^{maj}), 80.1 (C-1^{maj}), 55.2 (C-1^{min}), 53.9 (isoindolyl C_A-1,3^{min}), 53.8 (isoindolyl C_A-1,3^{maj}), 53.2 (isoindolyl C_B-1,3^{maj}), 51.9 (isoindolyl C_B-1,3^{min}); δ_F (470 MHz, $CDCl_3$): 66.14 (SO_2F^{min}), 66.13 (SO_2F^{maj}); HRMS found MH^+ 462.1167. $C_{26}H_{20}FNO_4S$ requires *MH*, 462.1170. Full assignment of ^{13}C NMR not possible due to complexity of the NMR spectra.

4-[1-(5-Methoxy-1*H*-indol-3-yl)-2-(morpholin-4-yl)-2-oxoethyl]benzene-1-sulfonyl fluoride (1-15a) and 4-[1-(5-methoxy-1*H*-indol-1-yl)-2-(morpholin-4-yl)-2-oxoethyl]benzene-1-sulfonyl fluoride (1-15b)

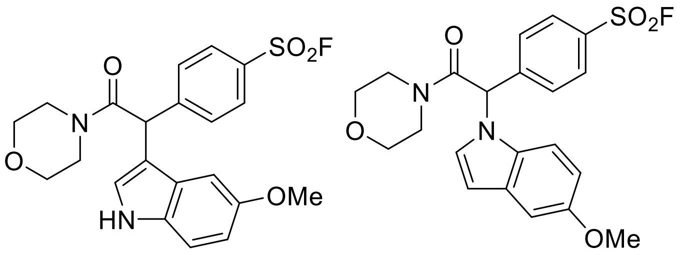

Prepared according to general procedure A – implementation of reaction array, diazo substrate **D1** (100 mM), co-substrate **C15** (500 mM) and $Rh_2(piv)_4$ (1 mM) gave a crude material. This was then purified via preparative HPLC eluting with gradient elution: 5:95 → 35:65 → 60:40 → 95:5 MeCN–H_2O to give the *indole derivative* ***1-15a*** (0.50 mg, 6%) as a colourless oil. R_f 0.09 (EtOAc–hexane 50:50). ν_{max}/cm^{-1}: 3325, 2923, 2856, 1640, 1485, 1439, 1409, 1212, 1115, 1033; δ_H (500 MHz, $CDCl_3$): 8.88 (1H, br. s, NH), 7.94 (2H, d, *J* 8.5 Hz, aryl 2,6-H), 7.51 (2H, d, *J* 8.5 Hz, aryl 3,5-H), 7.24 (1H, d, *J* 8.8 Hz, indolyl 7-H), 7.03 (1H, d, *J* 2.4 Hz, indolyl 4-H), 6.86 (1H, dd, *J* 8.8 and 2.4 Hz, indolyl 6-H), 6.37 (1H, d, *J* 1.8 Hz, indolyl 2-H), 5.53 (1H, s, 1-H), 3.84 (3H, s, OMe), 3.81-5.51 (8H, m, morpholinyl 2,6-H_2 and morpholinyl 3,5-H_2); δ_C (125 MHz, $CDCl_3$): 168.9 (C-2), 154.7 (indolyl C-5), 147.1 (aryl C-4), 133.8 (indolyl C-3a), 132.2 (d, *J* 24.9 Hz, aryl C-1), 131.8 (indolyl C-7a), 129.5 (aryl C_2-3,5), 129.1 (aryl C_2-2,6), 128.4 (indolyl C-3), 113.0 (indolyl C-6), 112.2 (indolyl C-7), 102.6 (indolyl C-2), 102.3 (indolyl C-4), 66.9 (morpholinyl C_A-2,6), 66.7 (morpholinyl C_B-2,6), 56.0 (OMe), 47.1 (morpholinyl C_A-3,5), 47.0 (C-1), 42.9 (morpholinyl C_B-3,5); δ_F (470 MHz, $CDCl_3$): 66.2 (SO_2F); HRMS found MH^+ 433.1223. $C_{21}H_{21}FN_2O_5S$ requires *MH*, 433.1228.

Also obtained was the *indole derivative* ***1-15b*** (0.70 mg, 8%) as a colourless oil. R_f 0.14 (EtOAc–hexane 50:50). ν_{max}/cm^{-1}: 2925, 2857, 1655, 1576, 1479, 1411, 1242, 1213, 1115, 1033; δ_H (500 MHz, $CDCl_3$): 7.94 (2H, d, *J* 8.5 Hz, aryl 2,6-H), 7.25 (2H, d, *J* 8.5 Hz, aryl 3,5-H), 7.17 (1H, d, *J* 3.3 Hz, indolyl 2-H), 7.14 (1H, d, *J* 2.4 Hz, indolyl 4-H), 7.07 (1H, d, *J* 8.9 Hz, indolyl 7-H), 6.89 (1H, dd, *J* 8.9 and 2.4 Hz, indolyl 6-H), 6.61 (1H, dd, *J* 3.3 and 0.7 Hz, indolyl 3-H), 6.36 (1H, s, 1-H), 3.92-3.86 (1H, m, morpholinyl 3-H_A or morpholinyl 5-H_A), 3.85 (3H, s, OMe), 3.75 (1H, ddd, *J* 10.9, 5.5 and 2.8 Hz, morpholinyl 2-H_A or morpholinyl 6-H_A), 3.65-3.54 (2H, m, morpholinyl 3-

H_B or morpholinyl 5-H_B and morpholinyl 2-H_B or morpholinyl 6-H_B), 3.49 (1H, ddd, *J* 11.6, 5.5 and 3.1 Hz, morpholinyl 2-H_A or morpholinyl 6-H_A), 3.38 (1H, ddd, *J* 13.1, 7.6 and 3.1 Hz, morpholinyl 3-H_A or morpholinyl 5-H_A), 3.22 (1H, ddd, *J* 13.1, 5.5 and 3.1 Hz, morpholinyl 3-H_B or morpholinyl 5-H_B), 3.19-3.13 (1H, m, 2-H_B or morpholinyl 6-H_B); δ_C (125 MHz, $CDCl_3$): 165.7 (C-2), 155.1 (indolyl C-5), 145.2 (aryl C-4), 132.9 (d, *J* 24.9 Hz, aryl C-1), 130.7 (indolyl C-7a), 129.7 (indolyl C-3a), 128.9 (aryl C_2-2,6), 128.8 (aryl C_2-3,5), 126.7 (indolyl C-2), 113.3 (indolyl C-6), 109.7 (indolyl C-7), 104.0 (indolyl C-3), 103.5 (indolyl C-4), 66.8 (morpholinyl C_A-2,6), 66.3 (morpholinyl C_B-2,6), 60.1 (C-1), 55.9 (OMe), 46.4 (morpholinyl C_A-3,5), 43.3 (morpholinyl C_B-3,5); δ_F (470 MHz, $CDCl_3$): 66.1 (SO_2F); HRMS found MH^+ 433.1226. $C_{21}H_{21}FN_2O_5S$ requires *MH*, 433.1228.

4-(1-((4-Chlorobenzyl)oxy)-2-morpholino-2-oxoethyl)phenyl sulfurofluoridate (4-1)

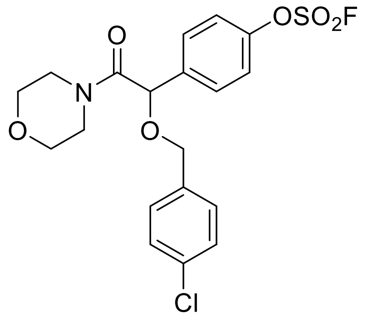

Prepared according to general procedure A - implementation of reaction array with diazo substrate **D4** (100 mM), co-substrate **C1** (500 mM) and $Rh_2(pfb)_4$ (1.0 mM) gave a crude material which was purified by reverse phase MDAP-HPLC eluting with H_2O/CH_3CN (with 0.1% formic acid) over 12 min to give the *sulfurofluoridate* as an orange oil (5.2 mg, 56%). ν_{max}/cm^{-1} 2922, 2854, 1647, 1500, 1450, 1367, 1272, 1234, 1181, 1144, 1115, 1089, 1016, 916, 813, 578 and 541. δ_H (500 MHz, CD_3OD) 7.65-7.62 (2H, m, phenyl 3,5-H_2), 7.51-7.49 (2H, m, phenyl 2,6-H_2), 7.40-7.36 (4H, m, chlorobenzyl 3,5-H_2, 2,6-H_2), 5.44 (1H, s, oxoethyl 1-H), 4.65 (1H, d, *J* 11.9 Hz, benzylic 1-H_A), 4.58 (1H, d, *J* 11.7 Hz, benzylic 1-H_B), 3.64-3.39 (8H, m, morpholino 2,6-H_4 and 2,5-H_4). δ_C (125 MHz, CD_3OD) 170.1 (oxoethyl C-2), 151.4 (phenyl C-1), 139.1 (phenyl C-4), 137.4 (chlorobenzyl C-1), 134.9 (chlorobenzyl C-4), 130.9 (phenyl C_2-3,5), 130.5 (chlorobenzyl C_2-2,6), 129.6 (chlorobenzyl C_2-3,5), 122.5 (phenyl C_2-2,6), 80.3 (oxoethyl C-1), 72.3 (benzylic C), 67.7 (morpholino C_2-2,6),

67.4 (morpholino C_2-2,6), 47.1 (morpholino C_2-3,5), 44.0 (morpholino C_2-5,3); δ_F (376 MHz, CD_3OD) 35.7 (SO_3*F*). HRMS found MH^+ 444.0688. $C_{19}H_{19}ClFNO_6S$ requires *MH* 444.0678.

4-(1-(3-(2-Hydroxyethyl)-1H-indol-1-yl)-2-morpholino-2-oxoethyl)phenyl sulfurofluoridate (4-3)

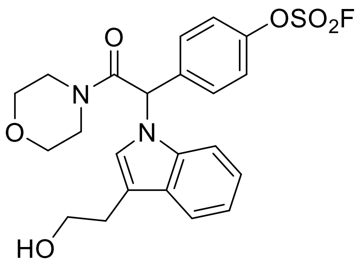

Prepared according to general procedure A - implementation of reaction array with diazo substrate **D4** (100 mM), co-substrate **C3** (500 mM) and $Rh_2(pfb)_4$ (1.0 mM) gave a crude material which was purified by reverse phase MDAP-HPLC eluting with H_2O/CH_3CN (with 0.1% formic acid) over 12 min to give the *sulfurofluoridate* as a colourless oil (2.12 mg, 23%). ν_{max}/cm^{-1} 3444, 2924, 2856, 1650, 1503, 1449, 1361, 1302, 1273, 1233, 1184, 1144, 1114, 1036, 1018, 916, 816, 744, 580,570, 543, 515 and 428. δ_H (500 MHz, CD_3OD) 7.63-7.61 (1H, m, indolyl 4-H), 7.46-7.43 (2H, m, phenyl 2,6-H_2), 7.38-7.34, (2H, m, phenyl 3,5-H_2), 7.19-7.16 (1H, m, indolyl 6-H), 7.12-7.09 (1H, m, indolyl 5-H), 7.01 (1H, s, indolyl 2-H), 6.86 (1H, s, oxoethyl 1-H), 3.82 (2H, t, *J* 7.0, hydroxyethyl 1-H_2), 3.80-3.76 (1H, m, morpholino 3-H^a or 5-H^b), 3.72-3.68 (1H, m, morpholino 2-H^a or 6-H^b), 3.66-3.57 (2H, m, morpholino 3-H^b or 5-H^a and 2-H^b or 6-H^a), 3.47-3.38 (3H, m, morpholino 3,5-$H_2^{a\ or\ b}$ and 2- or 6-$H^{a\ or\ b}$), 3.28-3.22 (1H, m, morpholino 2- or 6-$H^{a\ or\ b}$), 3.02-2.93 (2H, m, hydroxyethyl 2-H_2). δ_C (125 MHz, CD_3OD) 169.0 (oxoethyl C-2), 151.2 (phenyl C-1), 139.1 (phenyl C-4), 137.9 (indolyl C-7a), 131.4 (phenyl C_2-3,5), 129.9 (indolyl C-3a), 125.8 (indolyl C-2), 122.4 (indolyl C-6), 122.3 (phenyl C_2-2,6) 120.9 (indolyl C-5), 120.2 (indolyl C-4), 114.8 (indolyl C-3), 110.5 (indolyl C-7), 67.7 (morpholino C-2 or C-6), 67.3 (morpholino C-2 or C-6), 63.3 (hydroxyethyl C-1), 60.2 (oxoethyl C-1), 47.6 (morpholino C-3 or C-5), 44.3 (morpholino C-3 or C-5), 29.6 (hydroxyethyl C-2). δ_F (376 MHz, CD_3OD) 35.7 (SO_3*F*).

4-(1-((2-Chloro-5-fluorobenzyl)oxy)-2-morpholino-2-oxoethyl)phenyl sulfurofluoridate (4-5)

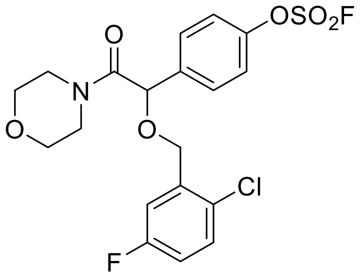

Prepared according to general procedure A - implementation of reaction array with diazo substrate **D4** (100 mM), co-substrate **C5** (500 mM) and $Rh_2(cap)_4$ (1.0 mM) gave a crude material which was purified by reverse phase MDAP-HPLC eluting with H_2O/CH_3CN (with 0.1% formic acid) over 12 min to give the *sulfurofluoridate* as a colourless oil (0.77 mg, 8%). ν_{max}/cm^{-1} 2924, 2854, 1649, 1501, 1450, 1271, 1234, 1182, 1145, 1116, 1018, 916, 813 and 578. δ_H (500 MHz, CD_3OD) 7.68-7.65 (2H, m, phenyl 3,5-H_2), 7.53-7.50 (2H, m, phenyl 2,6-H_2), 7.41 (1H, dd, *J* 8.8 Hz and 5.0 Hz, benzyl 3-H), 7.34 (1H, dd, *J* 9.3 Hz and 3.1 Hz, benzyl 6-H), 7.10-7.06 (1H, m, benzyl 4-H), 5.6 (1H, s, oxoethyl 1-H), 4.75 (1H, d, *J* 12.7 Hz, benzylic-H_A), 4.67 (1H, d, *J* 12.8 Hz, benzylic-H_B), 3.68-3.54 (4H, m, morpholino 2,6-$H_2^{a\ or\ b}$), 3.52-3.46 (2H, m, morpholino 3,5-$H_2^{a\ or\ b}$ or 2,6-$H_2^{a\ or\ b}$), 3.44-3.36 (2H, m, morpholino 3,5-$H_2^{a\ or\ b}$). δ_C (125 MHz, CD_3OD) 151.5 (phenyl C-1), 138.8 (phenyl C-4), 132.0 (d, *J* 8.4 Hz, benzyl C-1), 130.7 (phenyl C_2-3,5), 122.6 (phenyl C_2-2,6), 117.4 (d, *J* 24.6 Hz, benzyl C-4) 117.1 (dd, *J* 27.6 and 23.2 Hz, benzyl C-6), 80.6 (oxoethyl C-1), 69.7 (benzylic C-1), 67.7 (morpholino C-2 or C-6), 67.4 (morpholino C-2 or C-6), 47.2 (morpholino C-3 or C-5), 44.0 (morpholino C-3 or C-5). δ_F (376 MHz, CD_3OD) 35.8 (SO_3*F*), - 116.9 (C*F*). HRMS found MNH_4^+ 479.0855. $C_{19}H_{18}ClF_2NO_6S$ requires *MNH_4^+*, 479.0850. N.B: Benzyl carbons C-2, C-3 and C-5 were not identified due to low sample concentration and fluorine splitting.

4-(2-Morpholino-2-oxo-1-(pyrimidin-2-ylamino)ethyl)phenyl sulfurofluoridate (4-13)

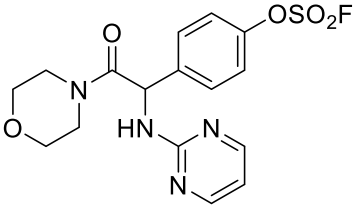

Prepared according to general procedure A - implementation of reaction array with diazo substrate **D4** (100 mM), co-substrate **C13** (500 mM) and $Rh_2(piv)_4$ (1.0 mM) gave a crude material which was purified by reverse phase MDAP-HPLC eluting with H_2O/CH_3CN (with 0.1% formic acid) over 12 min to give the *sulfurofluoridate* as an orange solid (2.75 mg, 35%). ν_{max}/cm^{-1} 2921, 2855, 1653, 1585, 1502, 1448, 1357, 1300, 1234, 1184, 1142, 1115, 1034, 915, 861, 800, 721, 599, 544 and 512. δ_H (500 MHz, CD_3OD) 8.32 (2H, d, *J* 4.8 Hz, pyrimidinyl 4,6-H_2), 7.71-7.68 (2H, m, phenyl 3,5-H_2), 7.49-7.46 (2H, m, phenyl 2,6-H_2), 6.68 (1H, t, *J* 4.8 Hz, pyrimidinyl 5-H), 6.12 (1H, s, oxoethyl 1-H), 3.73-3.55 (6H, m, morpholino 3- H_2, 5-H_2, 2-H_A and 6-H_A), 3.48-3.43 (1H, m, morpholino 2-H_B) and 3.29 (1H, m, morpholino 6-H_B). δ_C (125 MHz, CD_3OD) 170.7 (oxoethyl C-2), 162.3 (pyrimidinyl C-2), 159.3 (pyrimidinyl C_2-4,6), 151.2 (phenyl C-1) 140.2 (phenyl C-4), 131.6 (phenyl C_2-3,5), 122.5 (phenyl C_2-2,6), 122.7 (pyrimidinyl C-5), 67.6 (morpholino C-2 or C-6), 67.4 (morpholino C-2 or C-6), 55.9 (oxoethyl C-1), 47.4 (morpholino C-3 or C-5), 44.1 (morpholino C-3 or C-5); δ_F (376 MHz, CD_3OD) 35.7 (SO_3F). HRMS found MNa^+ 419.0795. $C_{16}H_{17}FN_4O_5S$ requires *MNa^+* 419.0796.

4-(2-Morpholino-2-oxo-1-(pent-1-yn-3-yloxy)ethyl)phenyl sulfurofluoridate (4-17)

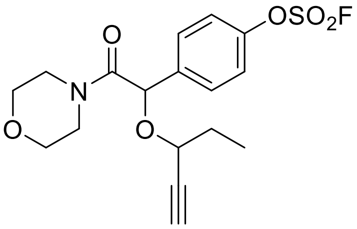

Prepared according to general procedure C - implementation of reaction array with diazo substrate **D4** (100 mM), co-substrate **C17** (500 mM) and $Rh_2(pfb)_4$ (1.0 mM) gave a crude material which was purified by reverse phase MDAP-HPLC eluting with

H_2O/CH_3CN (with 0.1% formic acid) over 12 min to give the *sulfurofluoridate* as a colourless oil (0.81 mg, 11%; 70:30 mixture of diastereomers A and B), v_{max}/cm^{-1} 3297, 3245, 3067, 2970, 2926, 2857, 1646, 1501, 1449, 1301, 1273, 1234, 1180, 1143, 1115, 1018, 916, 813, 665, 641, 595 and 542. δ_H (500 MHz, CD_3OD) 7.64-7.60 (2H, m, phenyl 3,5-H_2^{A+B}), 7.51-7.48 (2H, m, phenyl 2,6-H_2^{A+B}), 5.75 (0.3 H, s, ethyl 1-H^B), 5.61 (0.7 H, s, ethyl 1-H^A), 4.31-4.28 (0.3 H, app. m, pentynyl 3-H^B), 4.03 (0.7 H, td, *J* 6.3 and 2.0 Hz, pentynyl 3-H^A), 3.76-3.39 (8H, m, morpholine 2-, 3-, 5- and 6-H_2^{A+B}), 3.08 (0.7H, d, *J* 2.1 Hz, pentynyl 1-H^A), 3.04 (0.30, d, *J* 2.1 Hz, pentynyl H-1^B), 1.91-1.74 (2H, m, pentynyl 4-H_2^{A+B}), 1.07 (1H, t, *J* 7.5 Hz, pentynyl 5-H_3^B) and 1.03 (2H, t, *J* 7.4 Hz, pentynyl 5-H_3^A). δ_C (125 MHz, CD_3OD) (diasteromer A assingnments) 170.0 (oxoethyl C-2), 139.3 (phenyl C-1), 130.9 (phenyl C_2-3,5), 130.3 (phenyl C-4), 122.3 (phenyl C_2-2,6), 79.0 (pentynyl C-2), 77.4 (oxoethyl C-1), 71.2 (pentynyl C-3), 67.6 (morpholino C_2-2,6), 47.5 (morpholino C-3 or C-5), 44.1 (morpholino C-3 or C-5), 29.9 (pentynyl C-4), 9.8 (pentynyl C-5); δ_F (376 MHz, CD_3OD) 35.7 (SO_3F). HRMS found MNa^+ 408.0882. $C_{17}H_{20}FNO_6S$ [M+Na] requires *MNa^+*, 408.0898.

4-(2-Morpholino-2-oxo-1-(prop-2-yn-1-ylamino)ethyl)phenyl sulfurofluoridate (4-18)

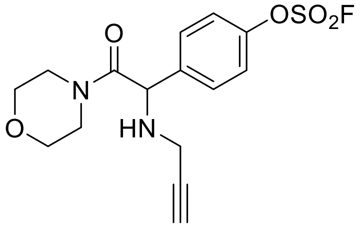

Prepared according to general procedure A - implementation of reaction array with diazo substrate **D4** (100 mM), co-substrate **C18** (500 mM) and $Rh_2(pfb)_4$ (1.0 mM) gave a crude material which was purified by reverse phase MDAP-HPLC eluting with H_2O/CH_3CN (with 0.1% formic acid) over 12 min to give the *sulfurofluoridate* as a yellow oil (1.65 mg, 23%). v_{max}/cm^{-1} 3303, 2974, 2925, 2860, 1657, 1504, 1452, 1362, 1303, 1272, 1235, 1188, 1145, 1115, 1068, 1019, 917, 861, 833, 798, 760, 721, 589, 542 and 527. δ_H (500 MHz, CD_3OD) 7.60 (2H, d, *J* 8.8 Hz, phenyl 3,5-H_2), 7.49 (2H, d, *J* 8.8 Hz, phenyl 2,6-H_2), 5.01 (1H, s, oxoethyl 1-H), 3.65-3.25 (10H, m, morpholino 2,3,5,6-H_8 and propynyl 1-H_2), 2.70 (1H, t, *J* 2.4 Hz, propynyl 3-H). δ_C (125 MHz, CD_3OD) 171.2 (oxoethyl C-2), 151.3 (phenyl C-1), 140.2 (phenyl C-4), 131.5 (phenyl

C_2-3,5), 122.6 (phenyl C_2-2,6), 81.6 (propynyl C-2), 74.05 (propynyl C-3), 67.6 (morpholino C-2 or C-6), 67.4 (morpholino C-2 or C-6), 60.9 (C-1), 47.1 (morpholino C-3 or C-5), 44.0 (morpholino C-3 or C-5), 36.7 (propynyl C-1). δ_F (376 MHz, CD_3OD) 35.7 (SO_3*F*). HRMS found MNa^+ 379.0722. $C_{15}H_{17}FN_2O_5S$ requires *MNa*$^+$ 379.0734.

2-(4-((2*H*-1,2,3-Triazol-2-yl)sulfonyl)phenyl)-2-((4-chlorobenzyl)oxy)-1-morpholinoethan-1-one (5-1)

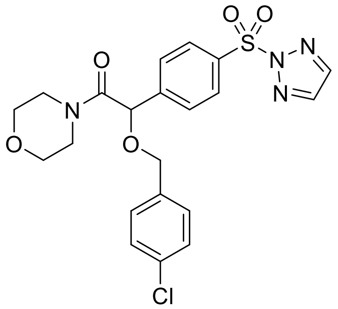

Prepared according to general procedure A - implementation of reaction array with diazo substrate **D5** (100 mM), co-substrate **C1** (500 mM) and $Rh_2(pfb)_4$ (1.0 mM) gave a crude material which was purified by reverse phase MDAP-HPLC eluting with H_2O/CH_3CN (with 0.1% formic acid) over 12 min to give the *sulfonyl triazole* as a colourless solid (7.56 mg, 26%). ν_{max}/cm^{-1} 3419, 2958, 2923, 2857, 2074, 1787, 1644, 1501, 1447, 1404, 1362, 1272, 1233, 1194, 1143, 1114, 1036, 1013, 954, 916, 844, 811, 765, 737, 659, 605 and 573. δ_H (500 MHz, CD_3OD) 8.07 (2H, d, *J* 8.6 Hz, phenyl 2,6-H_2), 8.02 (2H, s, triazolyl, 4,5-H_2), 7.71-7.69 (2H, m, phenyl 3,5-H_2), 7.36 (4H, s, benzyl 2,3,5,6-H_4), 5.46 (1H, s, ethanone 2-H), 4.64 (1H, d, *J* 11.8 Hz, benzylic-H_A), 4.56 (1H, d, *J* 11.8 Hz, benzylic-H_B), 3.61-3.34 (8H, m, morpholine, 2,3,5,6-H_8). δ_C (125 MHz, CD_3OD) 169.5, 146.2, 140.3, 137.2, 137.1, 135.0, 130.9, 130.0, 129.7, 129.4, 80.6, 72.5, 67.6, 67.4, 47.1, 44.0. HRMS found MH^+ 477.0988. $C_{21}H_{21}ClN_4O_5S$ requires *MH*, 477.0994.

General procedure for phenotypic *Trypanosoma brucei* cell culture

a. *Trypanosoma brucei brucei* culture

Trypanosoma brucei brucei (*T. b. brucei*) bloodstream form strain 427 were grown at 37 °C, in HMI-11 media in vented flasks in an atmosphere containing 5% CO_2. The cells were passaged by transferring into fresh media as to not exceed a cell density of 2 × 106 cells/mL.

b. Cryogenic storage of *Trypanosoma brucei brucei*

T. b. brucei (5 mL) at confluent density (2 × 106 cells/mL) was centrifuged at 800*g* for 5 minutes. The supernatant was removed, and cells resuspended in fresh media to a density of around 2 × 107 cells/mL. A sterile solution of 60% glycerol in water was added to the cell suspension to give a final concentration of 10% glycerol. The cell suspension was transferred to a cryovial, into a Mr Frosty (ThermoFisher Scientific Cat No. 5100-0001) containing isopropan-1-ol, which was then surrounded with dry ice and left for 24 hours. Cryovials were then stored at −170 °C in the liquid nitrogen vapour phase for long term storage.

Cells were revived from cryogenic storage by first removing them from liquid nitrogen onto ice for 15 minutes. The cells were then allowed to warm to room temperature in a culture hood. Once defrosted, the cells were transferred into a non-vented 25 mL cell culture flask with fresh medium (9.4 mL) and left to grow in a 37 °C shaking incubator.

c. Standard *Trypanosoma brucei brucei* resazurin cell viability assay

Cell viability assays were carried out in 96 well plates with 200 µL of culture per well. Cells were seeded at 5 × 103 cells/mL and incubated with drug for 66 hours (in the same conditions as culturing). The plate included wells containing the positive control pentamidine (100 nM) and the negative control of 0.5% DMSO. After 66 hours, 10 µL of 1.1 mg/mL resazurin sodium salt (in PBS) was added and incubated for a further 6 hours (for a total assay duration of 72 hours). Plates were then read on a plate reader using excitation/emission 560/590 nm.

Dose–response curves against *Trypanosoma brucei brucei*

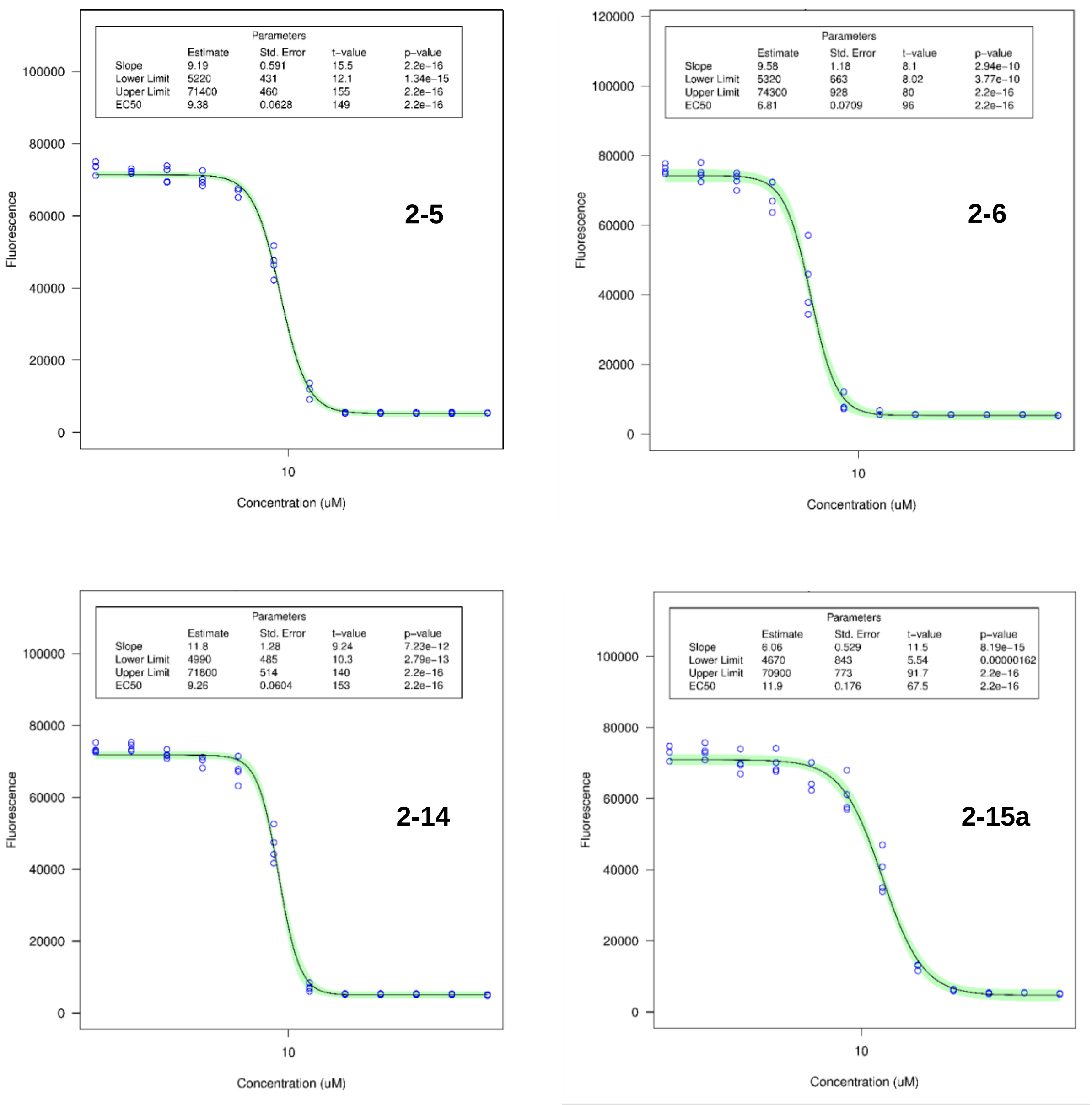

Figure S1: The dose–response curves for the most active chemical probes **2-5**, **2-6**, **2-14** and **2-15a** used to determine the EC_{50} values for these probes against *T. brucei*.

NMR spectra

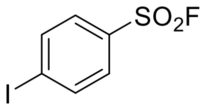
SO2F
I

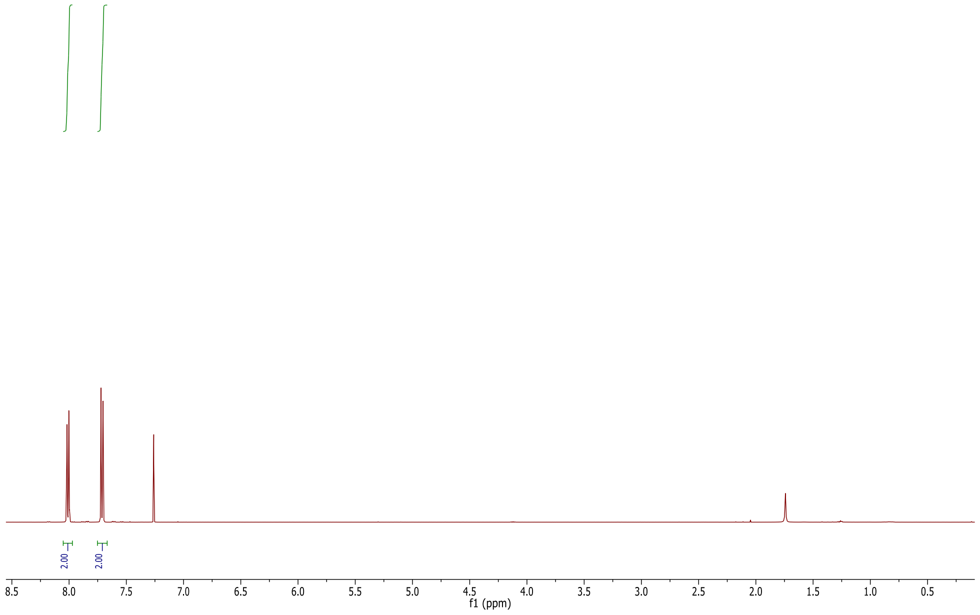
2.00
2.00
8.5 8.0 7.5 7.0 6.5 6.0 5.5 5.0 4.5 4.0 3.5 3.0 2.5 2.0 1.5 1.0 0.5
f1 (ppm)

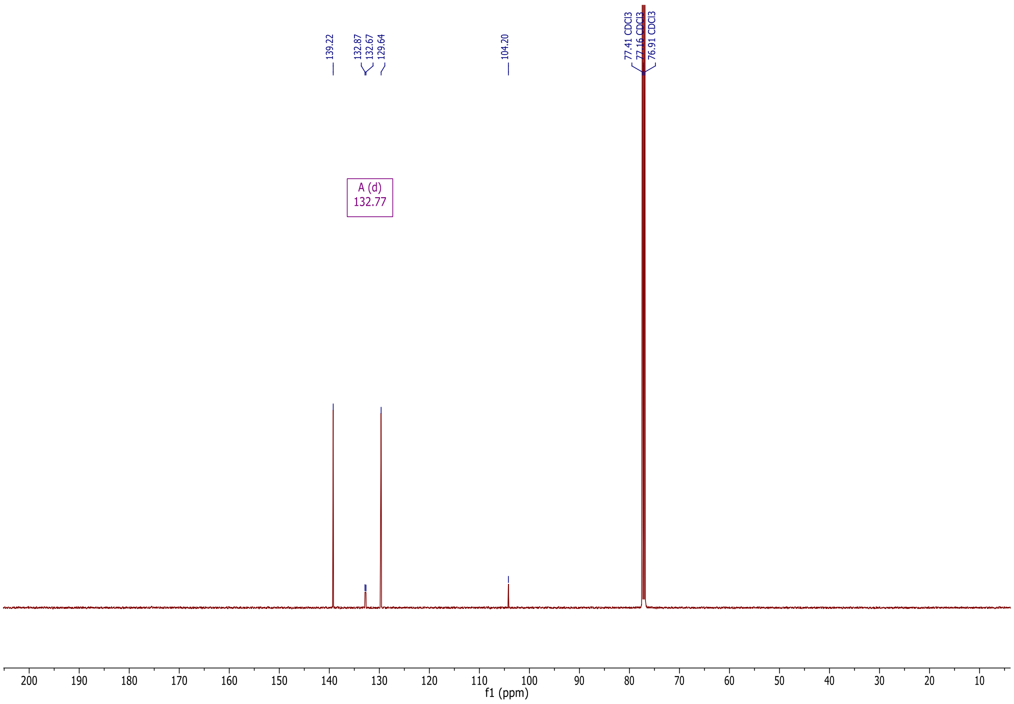
139.22
132.87
132.67
129.64
104.20
77.41 CDCl3
77.16 CDCl3
76.91 CDCl3
A (d)
132.77
200 190 180 170 160 150 140 130 120 110 100 90 80 70 60 50 40 30 20 10
f1 (ppm)

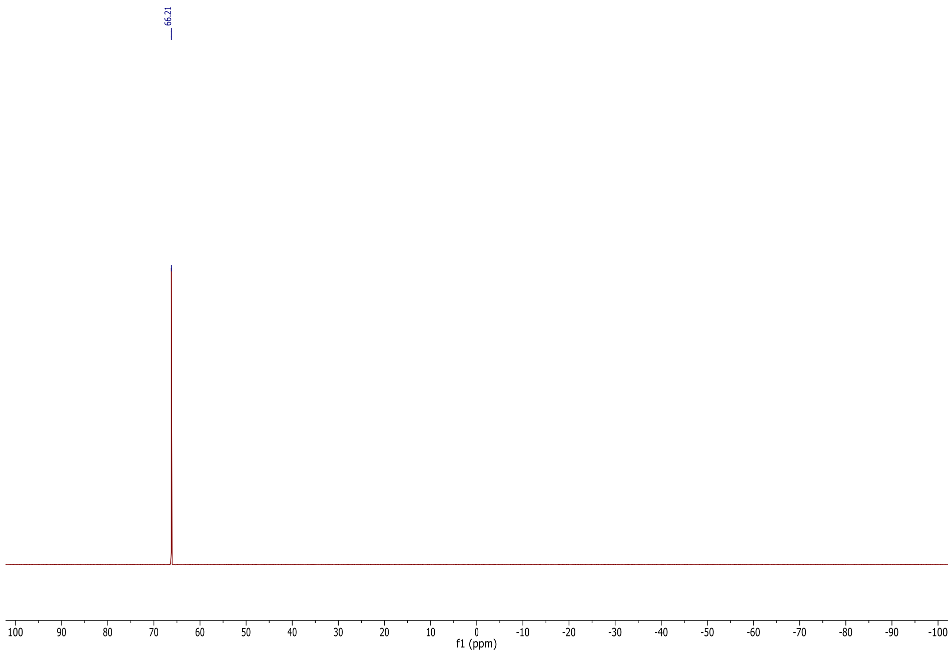
66.21
100
90
80
70
60
50
40
30
20
10
0
-10
-20
-30
-40
-50
-60
-70
-80
-90
-100
f1 (ppm)

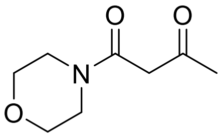

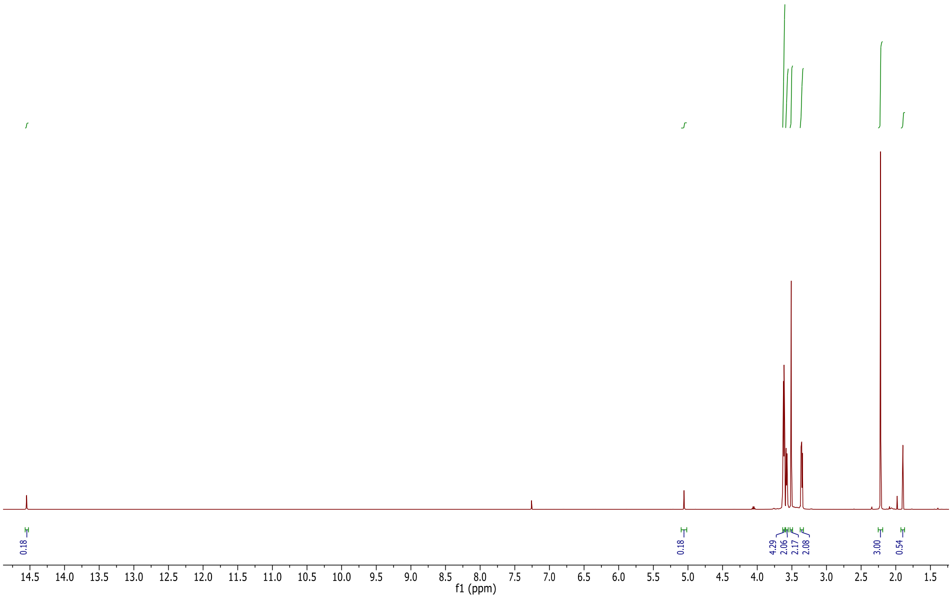
0.18
0.18
4.29
2.06
2.17
2.08
3.00
0.54
14.5 14.0 13.5 13.0 12.5 12.0 11.5 11.0 10.5 10.0 9.5 9.0 8.5 8.0 7.5 7.0 6.5 6.0 5.5 5.0 4.5 4.0 3.5 3.0 2.5 2.0 1.5
f1 (ppm)

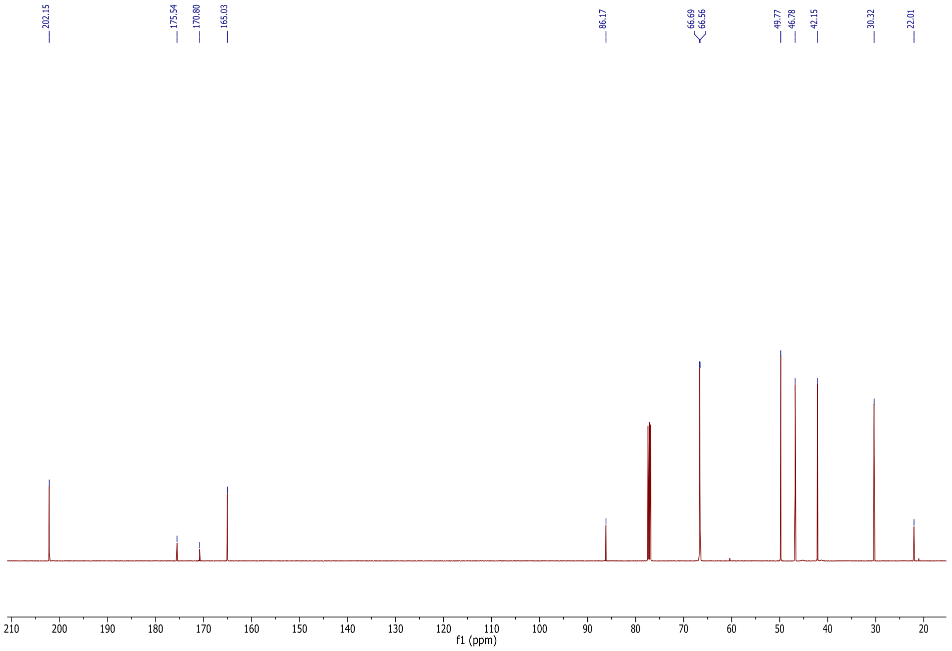
202.15
175.54
170.80
165.03
86.17
66.69
66.56
49.77
46.78
42.15
30.32
22.01
210 200 190 180 170 160 150 140 130 120 110 100 90 80 70 60 50 40 30 20
f1 (ppm)

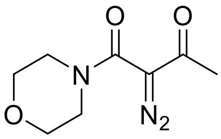

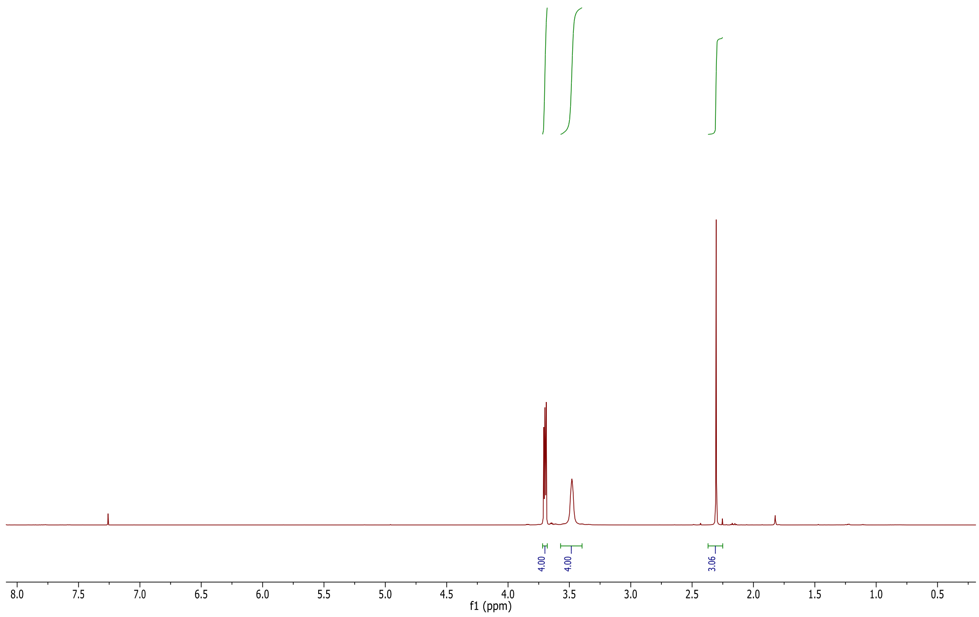
4.00
4.00
3.06
8.0
7.5
7.0
6.5
6.0
5.5
5.0
4.5
4.0
3.5
3.0
2.5
2.0
1.5
1.0
0.5
f1 (ppm)

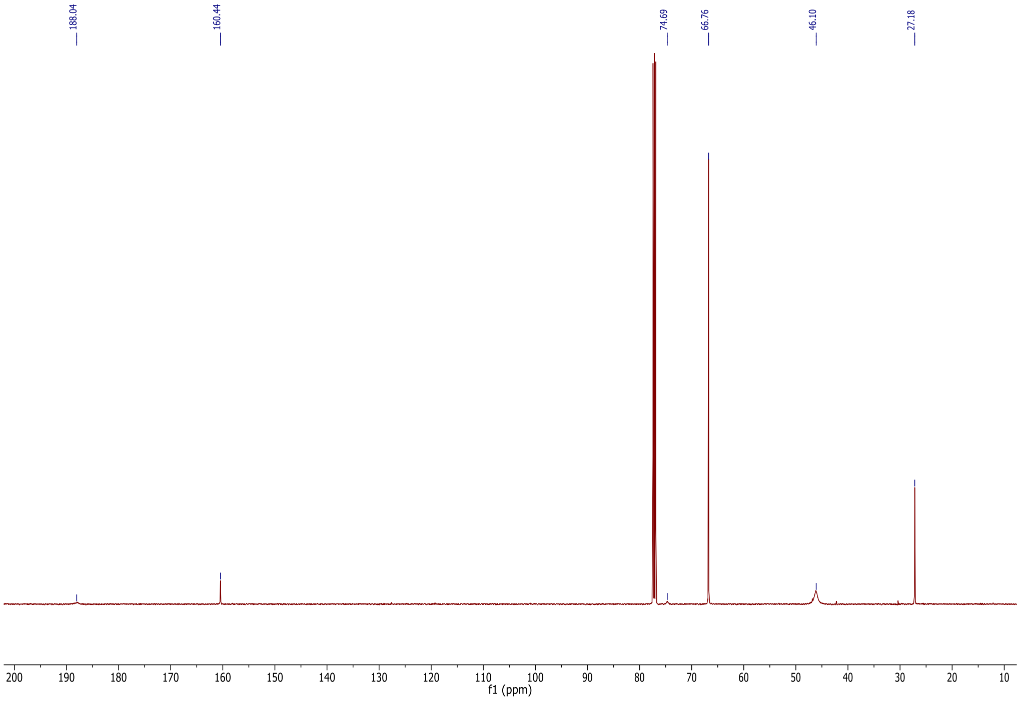
188.04
160.44
74.69
66.76
46.10
27.18
200
190
180
170
160
150
140
130
120
110
100
90
80
70
60
50
40
30
20
10
f1 (ppm)

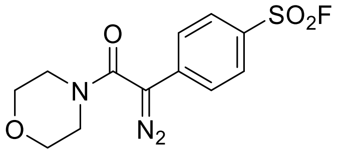

D1

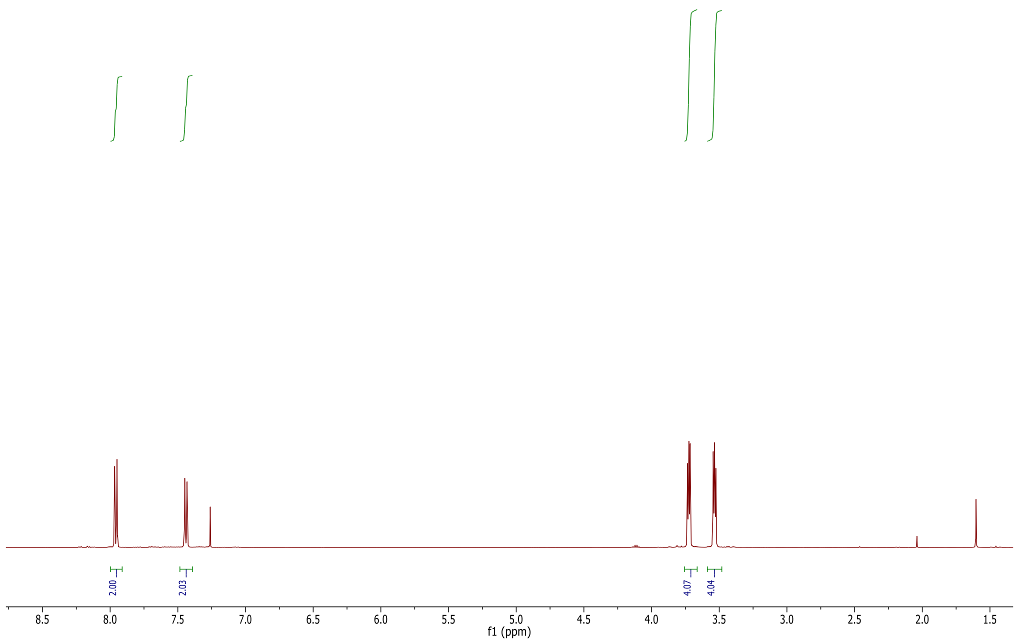
2.00
2.03
4.07
4.04
8.5
8.0
7.5
7.0
6.5
6.0
5.5
5.0
4.5
4.0
3.5
3.0
2.5
2.0
1.5
f1 (ppm)

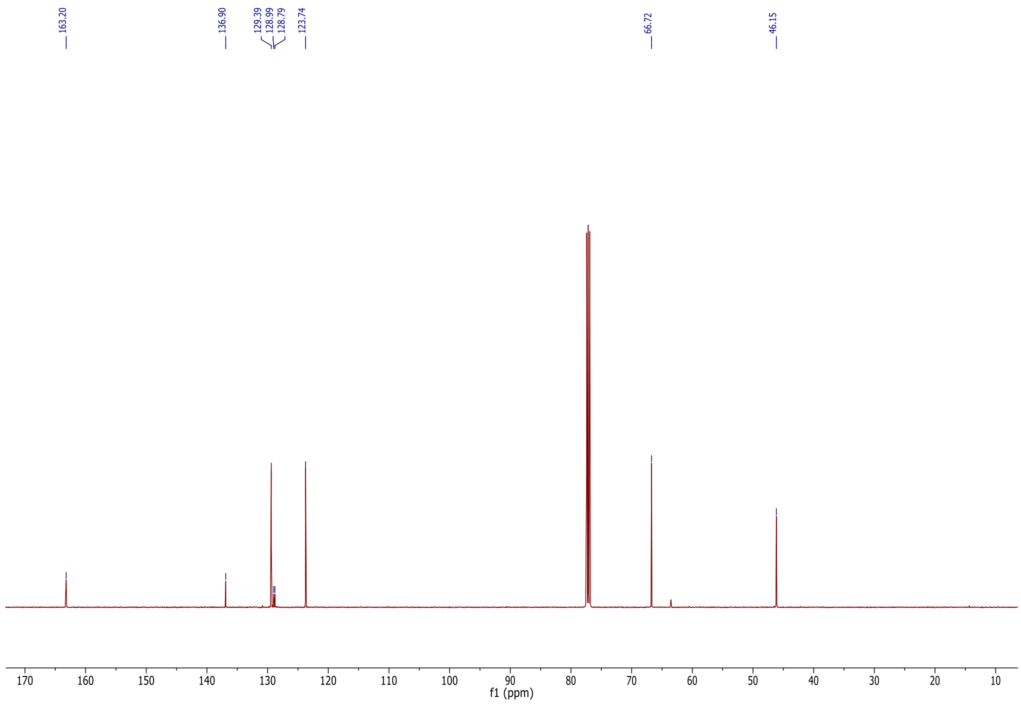
163.20
136.90
129.39
128.99
128.79
123.74
66.72
46.15
170
160
150
140
130
120
110
100
90
80
70
60
50
40
30
20
10
f1 (ppm)

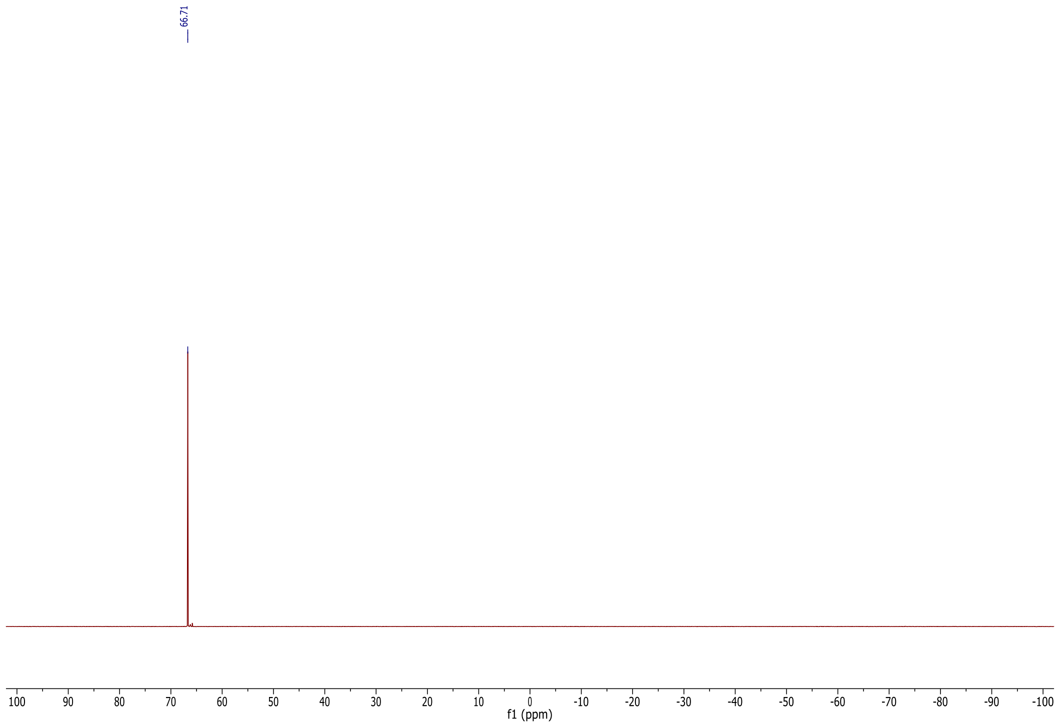
66.71
100 90 80 70 60 50 40 30 20 10 0 -10 -20 -30 -40 -50 -60 -70 -80 -90 -100
f1 (ppm)

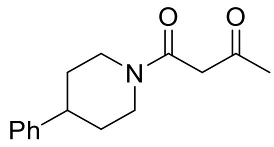
O
O
N
Ph

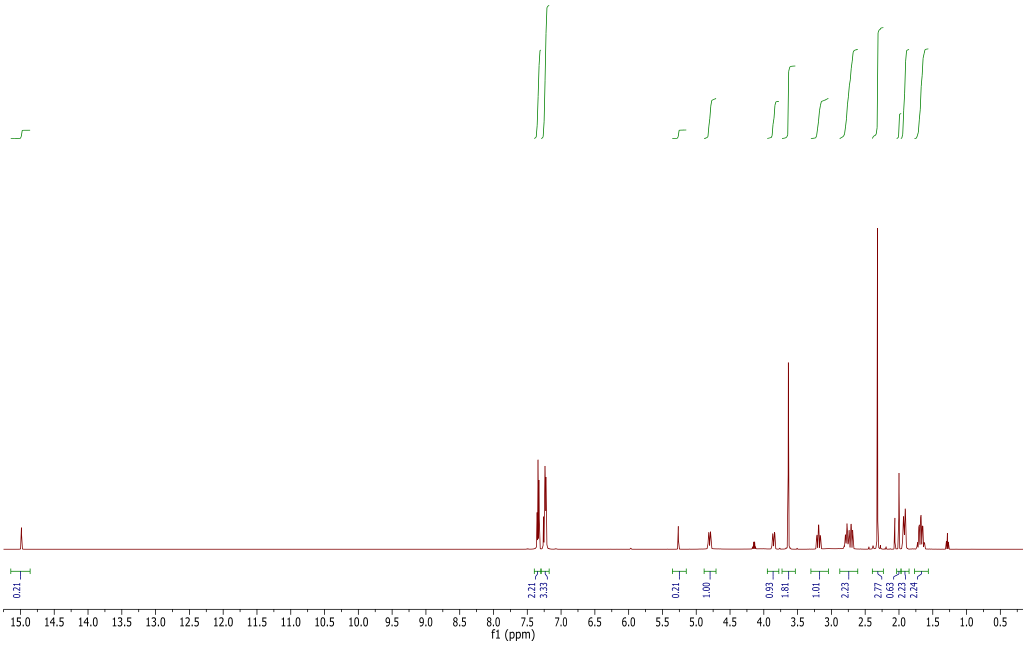
0.21
2.21
3.33
0.21
1.00
0.93
1.81
1.01
2.23
2.77
0.63
2.23
2.24
15.0 14.5 14.0 13.5 13.0 12.5 12.0 11.5 11.0 10.5 10.0 9.5 9.0 8.5 8.0 7.5 7.0 6.5 6.0 5.5 5.0 4.5 4.0 3.5 3.0 2.5 2.0 1.5 1.0 0.5
f1 (ppm)

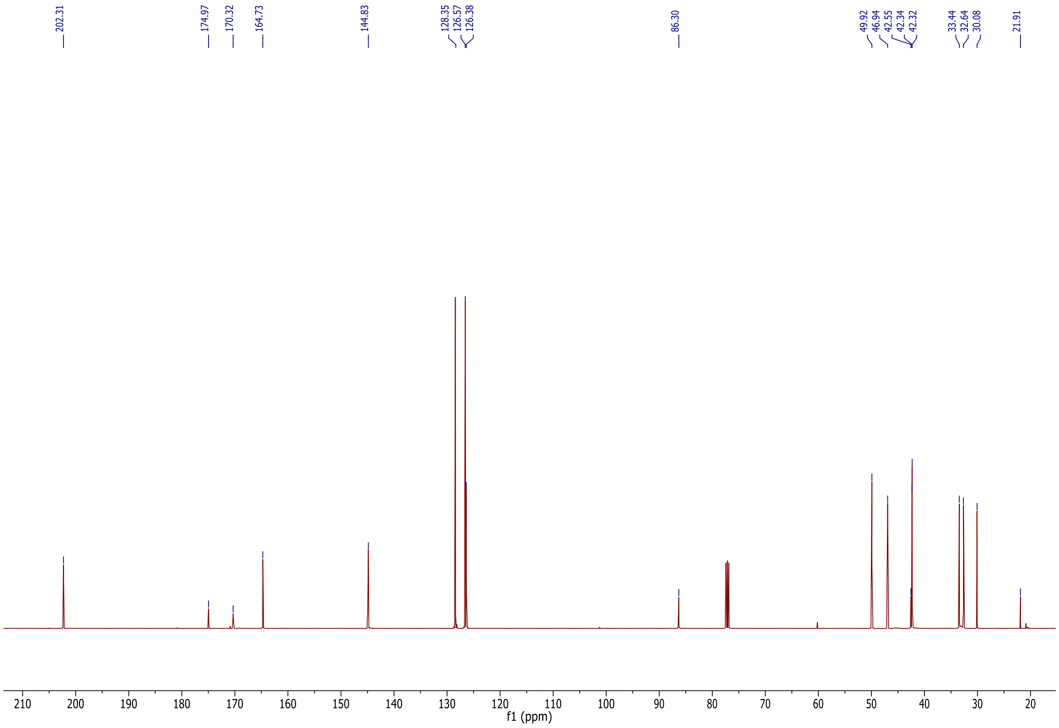
202.31
174.97
170.32
164.73
144.83
128.35
126.57
126.38
86.30
49.92
46.94
42.55
42.34
42.32
33.44
32.64
30.08
21.91
210 200 190 180 170 160 150 140 130 120 110 100 90 80 70 60 50 40 30 20
f1 (ppm)

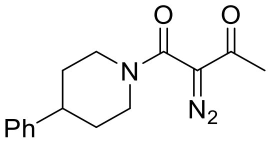

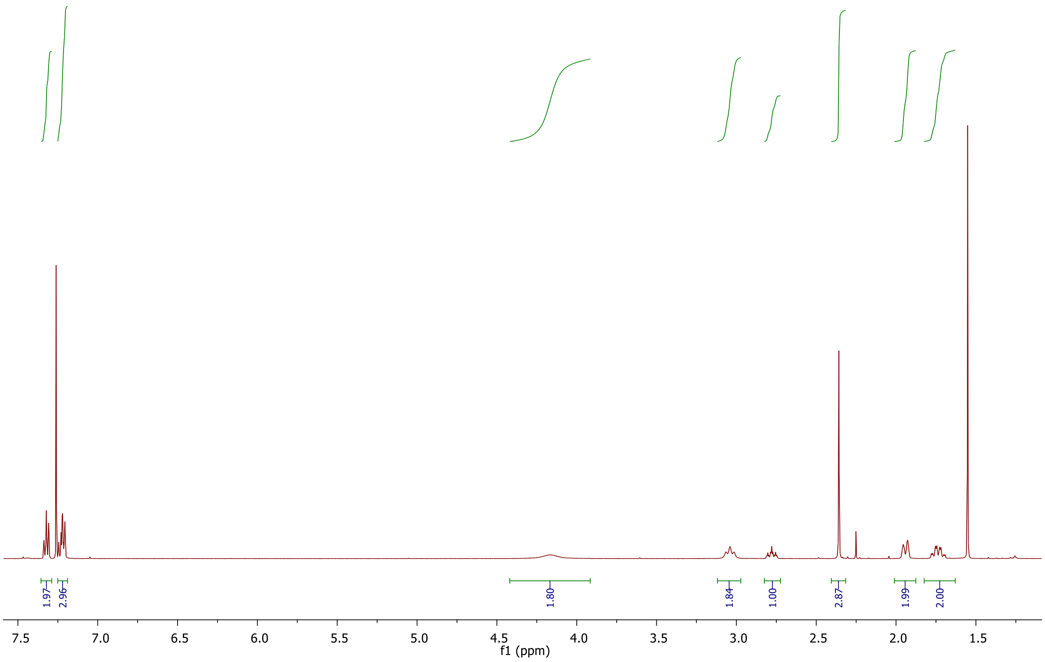

1.97
2.96
1.80
1.84
1.00
2.87
1.99
2.00
7.5
7.0
6.5
6.0
5.5
5.0
4.5
4.0
3.5
3.0
2.5
2.0
1.5
f1 (ppm)

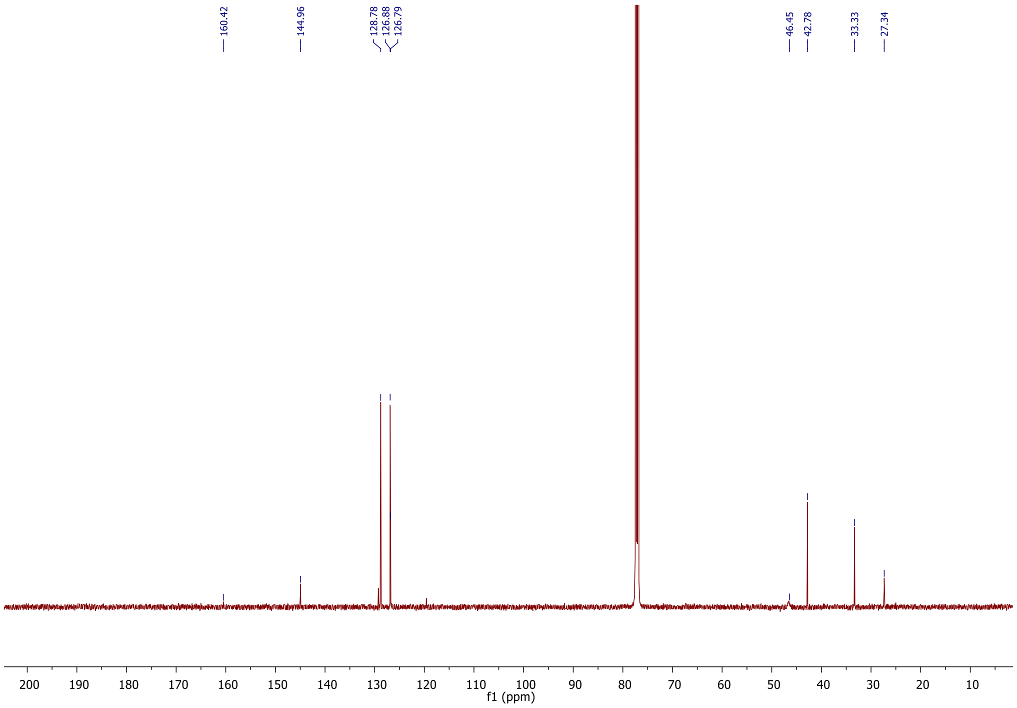

160.42
144.96
128.78
126.88
126.79
46.45
42.78
33.33
27.34
200
190
180
170
160
150
140
130
120
110
100
90
80
70
60
50
40
30
20
10
f1 (ppm)

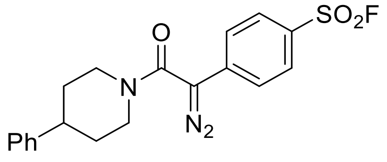
SO2F
O
N
N2
Ph

D2

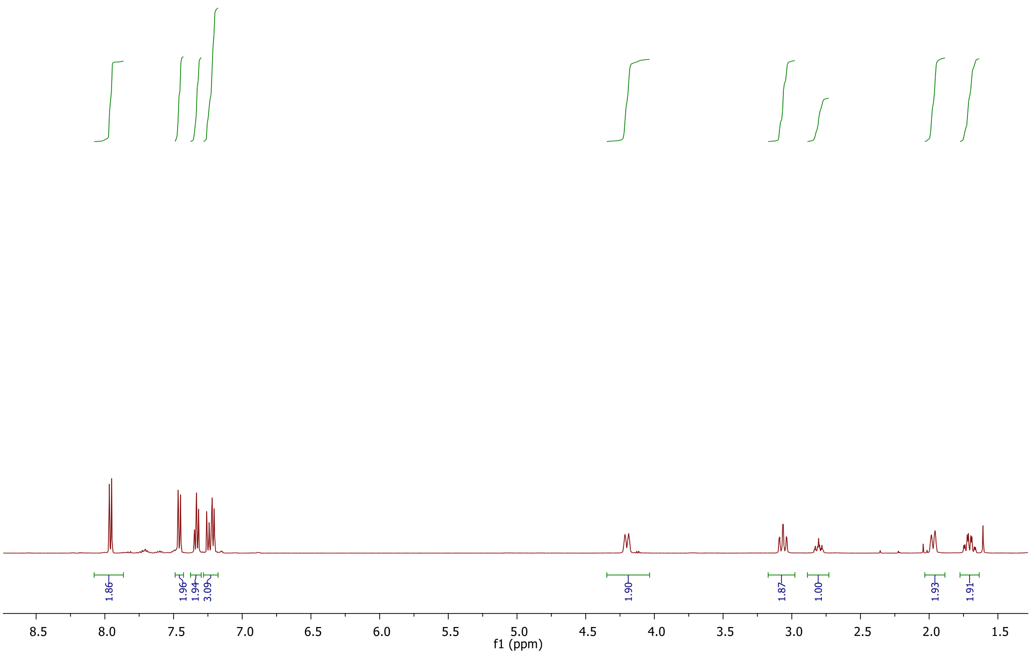
1.86
1.96
1.94
3.09
1.90
1.87
1.00
1.93
1.91
8.5
8.0
7.5
7.0
6.5
6.0
5.5
5.0
4.5
4.0
3.5
3.0
2.5
2.0
1.5
f1 (ppm)

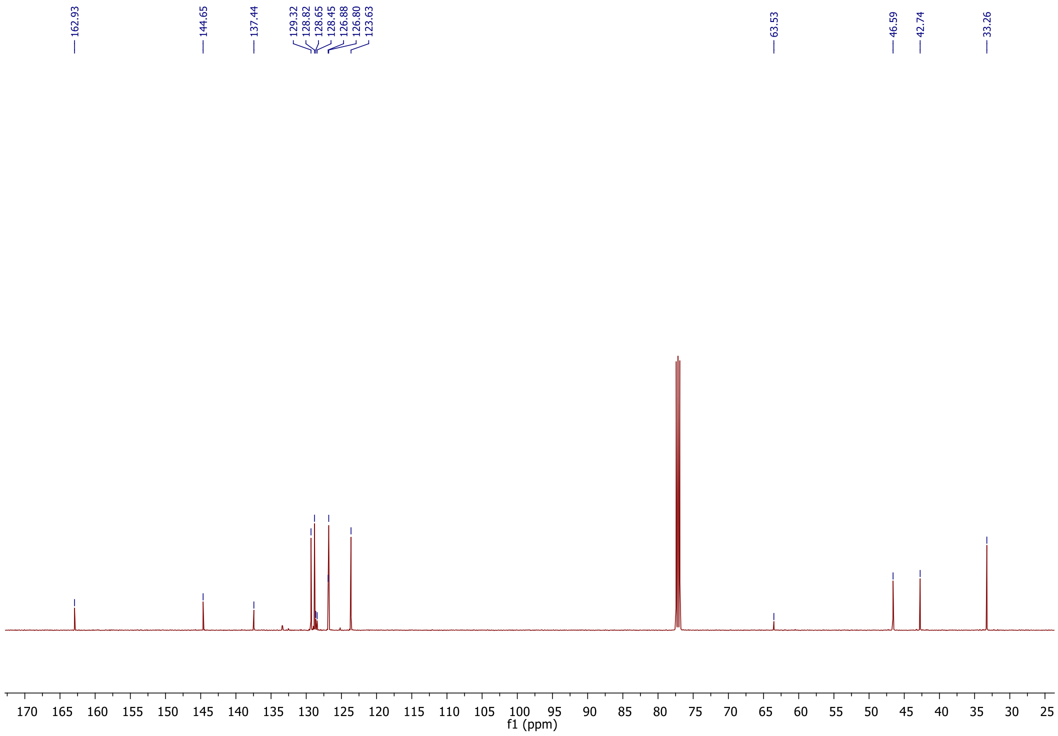
162.93
144.65
137.44
129.32
128.82
128.65
128.45
126.88
126.80
123.63
63.53
46.59
42.74
33.26
170
165
160
155
150
145
140
135
130
125
120
115
110
105
100
95
90
85
80
75
70
65
60
55
50
45
40
35
30
25
f1 (ppm)

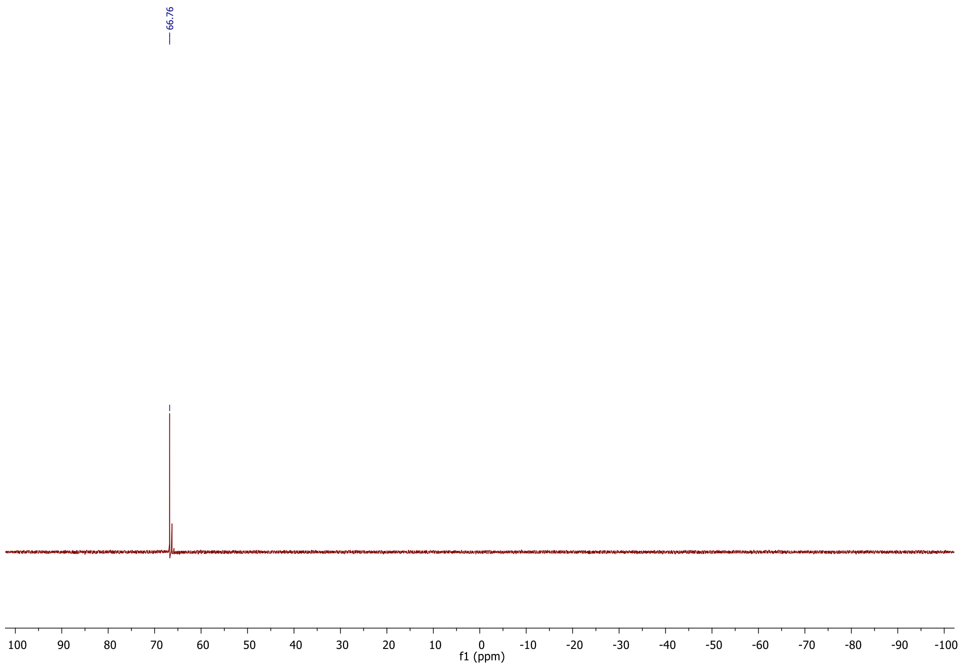

66.76
100
90
80
70
60
50
40
30
20
10
0
-10
-20
-30
-40
-50
-60
-70
-80
-90
-100
f1 (ppm)

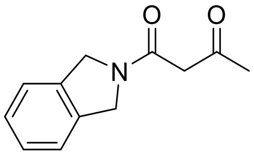
O
O
N

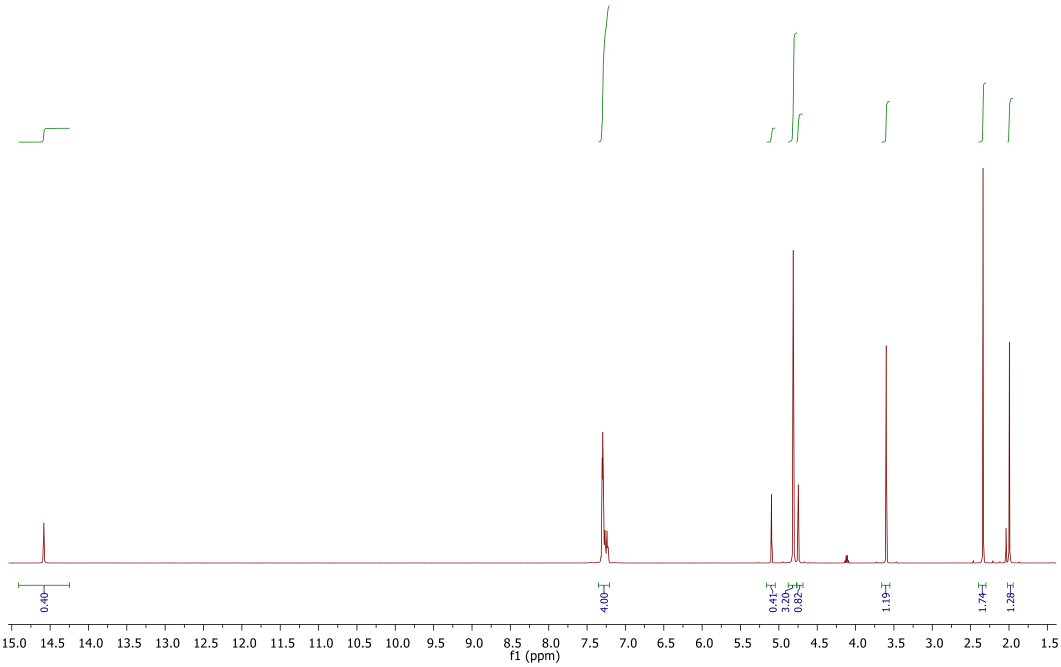
0.40
4.00
0.41
3.20
0.82
1.19
1.74
1.28
15.0 14.5 14.0 13.5 13.0 12.5 12.0 11.5 11.0 10.5 10.0 9.5 9.0 8.5 8.0 7.5 7.0 6.5 6.0 5.5 5.0 4.5 4.0 3.5 3.0 2.5 2.0 1.5
f1 (ppm)

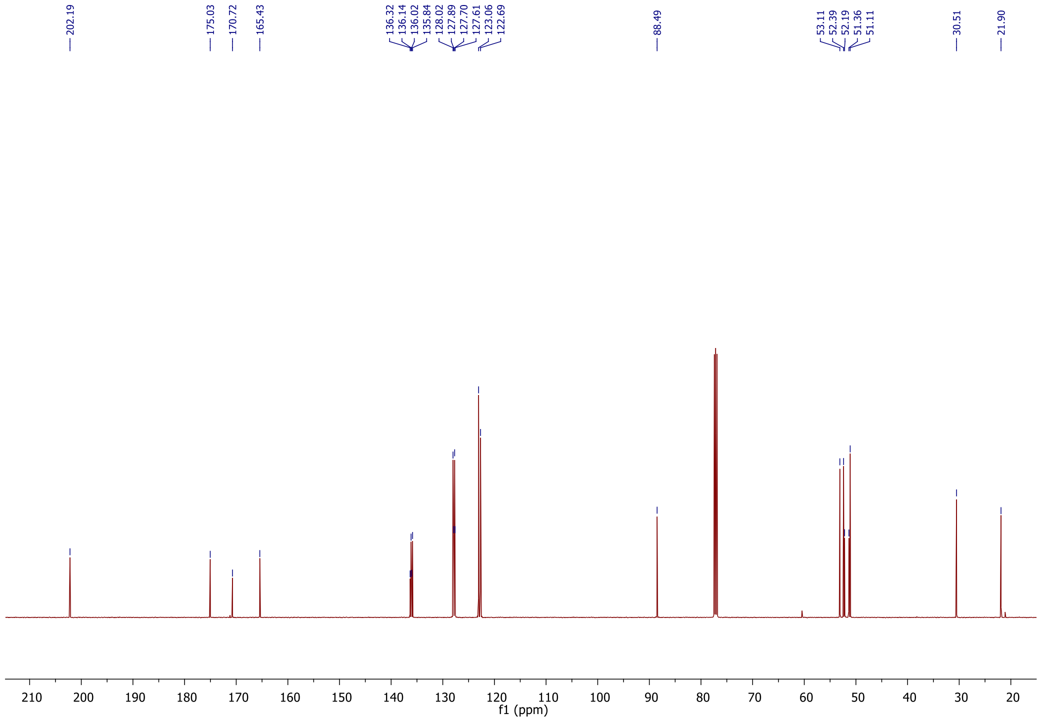
202.19
175.03
170.72
165.43
136.32
136.14
136.02
135.84
128.02
127.89
127.70
127.61
123.06
122.69
88.49
53.11
52.39
52.19
51.36
51.11
30.51
21.90
210 200 190 180 170 160 150 140 130 120 110 100 90 80 70 60 50 40 30 20
f1 (ppm)

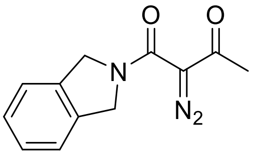
O
O
N
N2

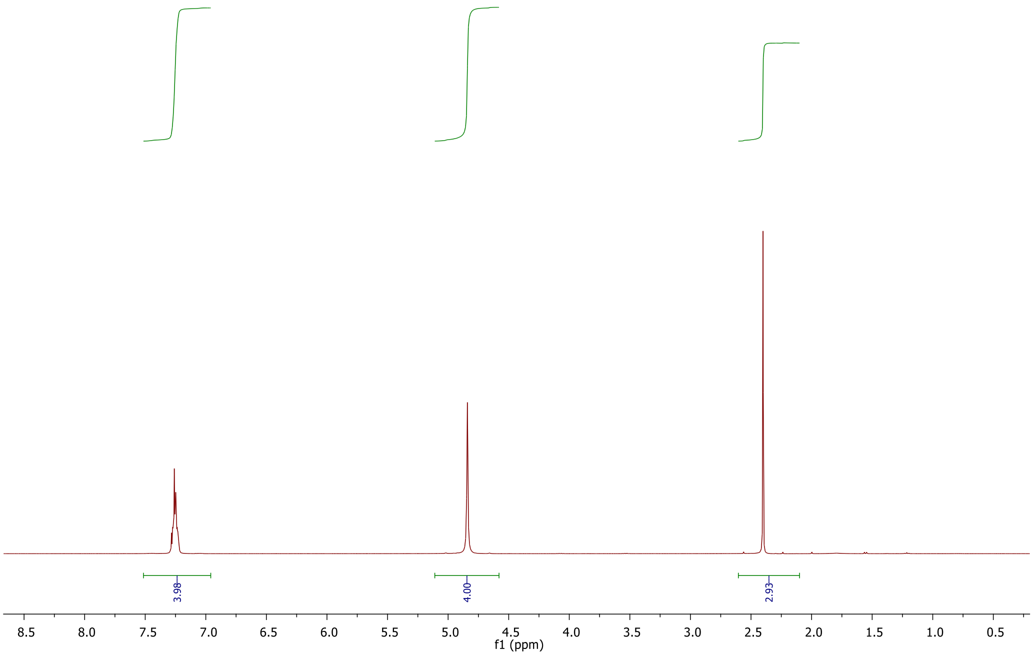
3.98
4.00
2.93
8.5 8.0 7.5 7.0 6.5 6.0 5.5 5.0 4.5 4.0 3.5 3.0 2.5 2.0 1.5 1.0 0.5
f1 (ppm)

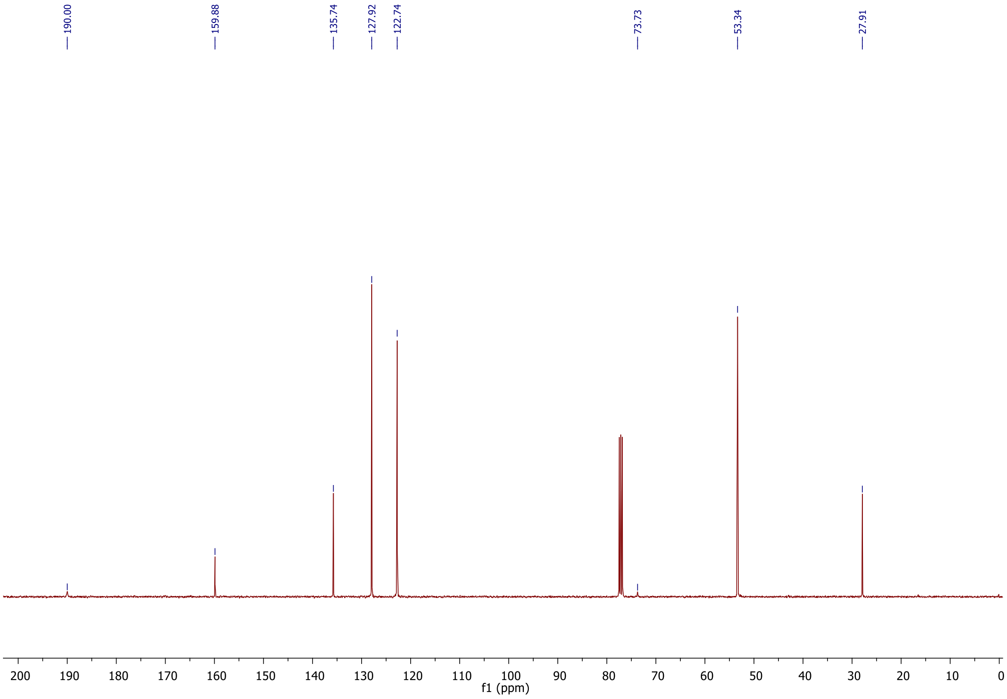
190.00
159.88
135.74
127.92
122.74
73.73
53.34
27.91
200 190 180 170 160 150 140 130 120 110 100 90 80 70 60 50 40 30 20 10 0
f1 (ppm)

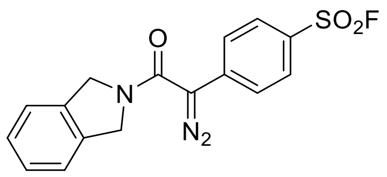

D3

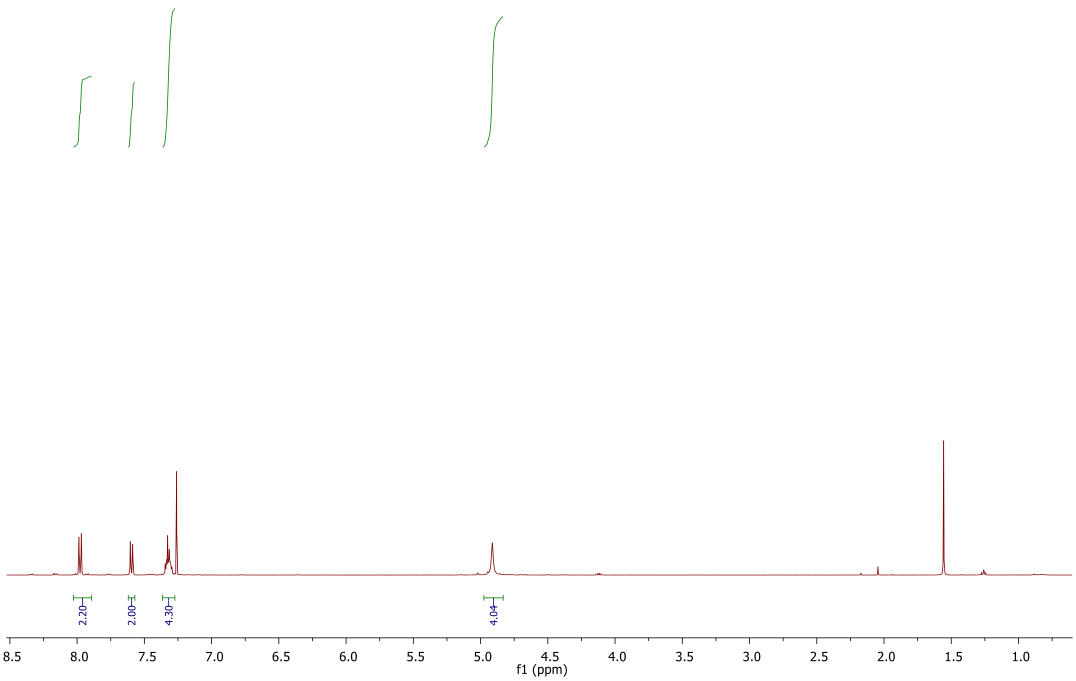

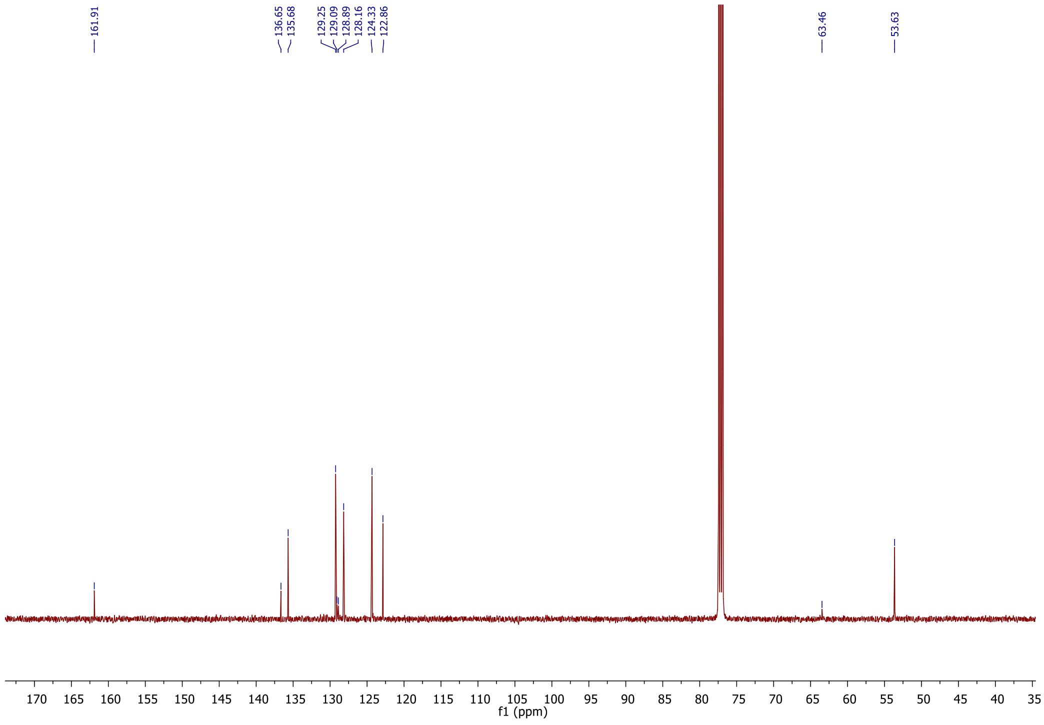

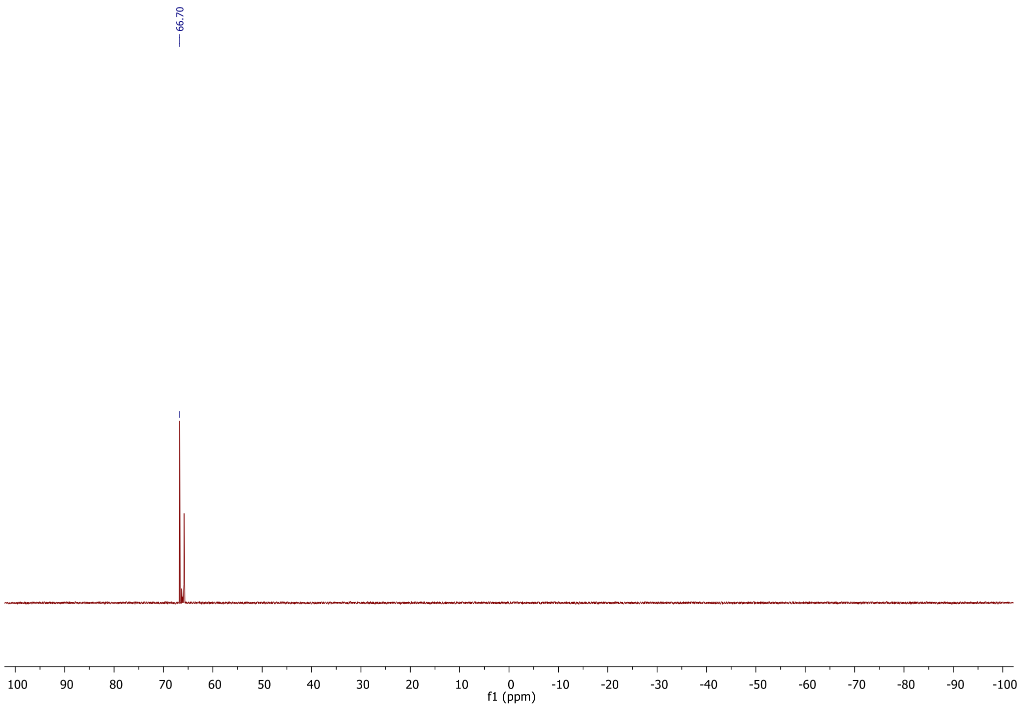
66.70
100 90 80 70 60 50 40 30 20 10 0 -10 -20 -30 -40 -50 -60 -70 -80 -90 -100
f1 (ppm)

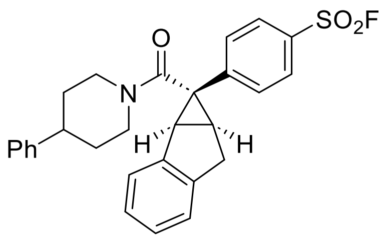
SO2F
O
N
Ph
H
H

2-7

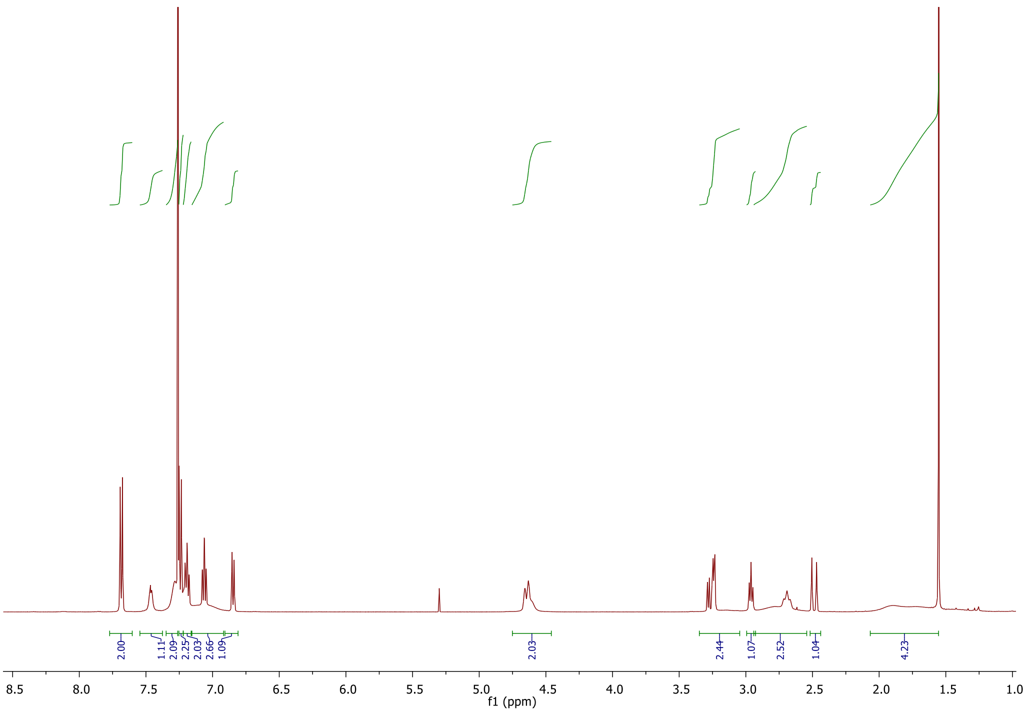
2.00
1.11
2.09
2.25
2.03
2.66
1.09
2.03
2.44
1.07
2.52
1.04
4.23
8.5 8.0 7.5 7.0 6.5 6.0 5.5 5.0 4.5 4.0 3.5 3.0 2.5 2.0 1.5 1.0
f1 (ppm)

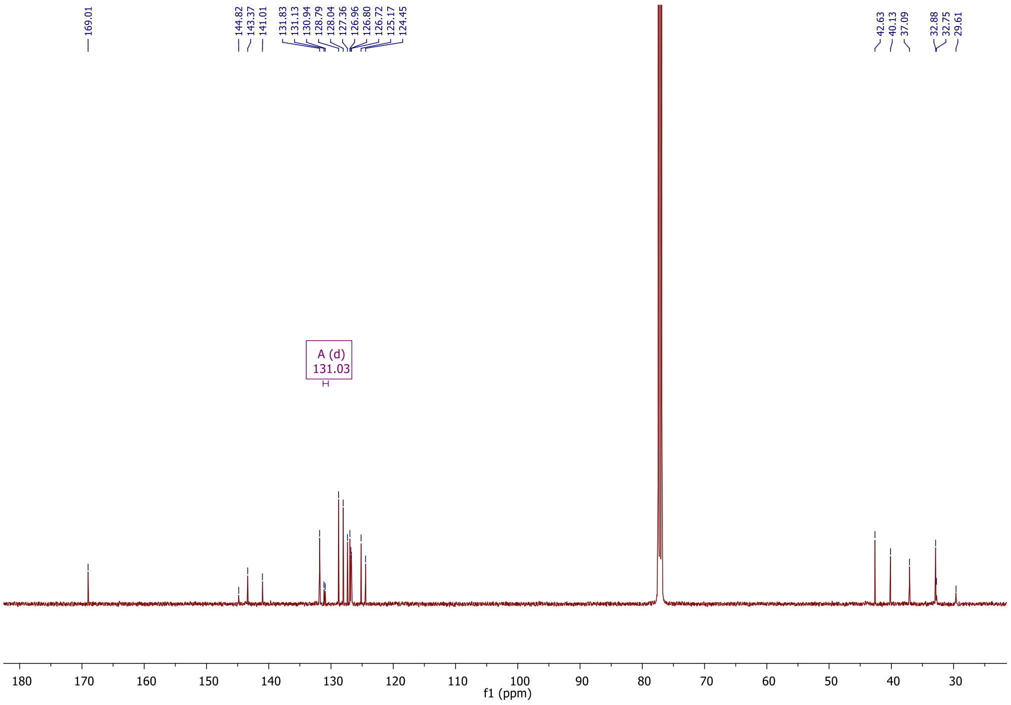
169.01
144.82
143.37
141.01
131.83
131.13
130.94
128.79
128.04
127.36
126.96
126.80
126.72
125.17
124.45
42.63
40.13
37.09
32.88
32.75
29.61
A (d)
131.03
180 170 160 150 140 130 120 110 100 90 80 70 60 50 40 30
f1 (ppm)

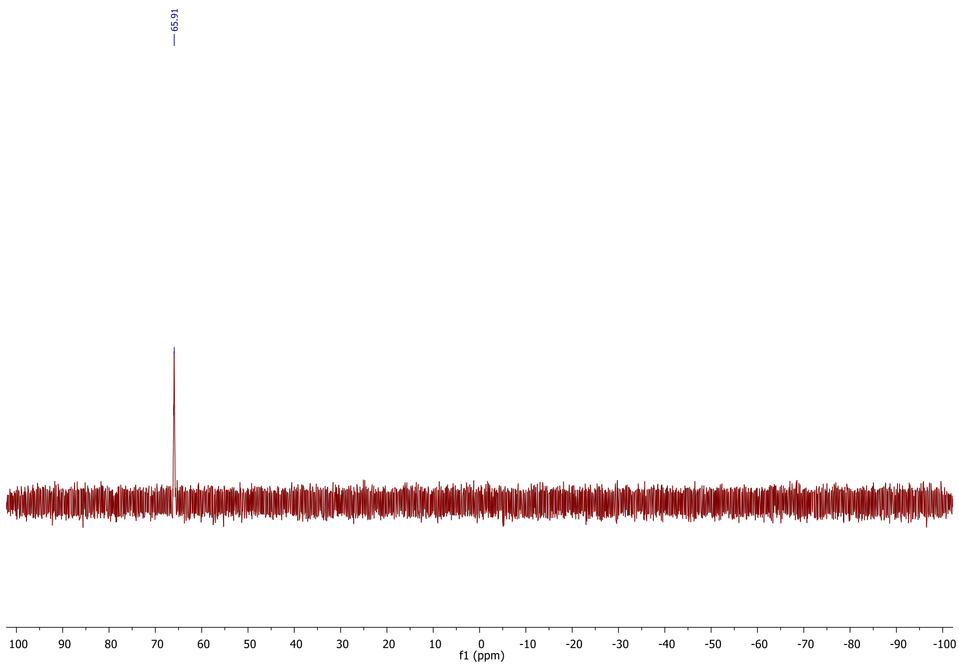
65.91
100
90
80
70
60
50
40
30
20
10
0
-10
-20
-30
-40
-50
-60
-70
-80
-90
-100
f1 (ppm)

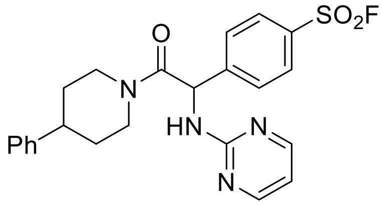
SO2F
O
N
HN
N
N
Ph

2-13

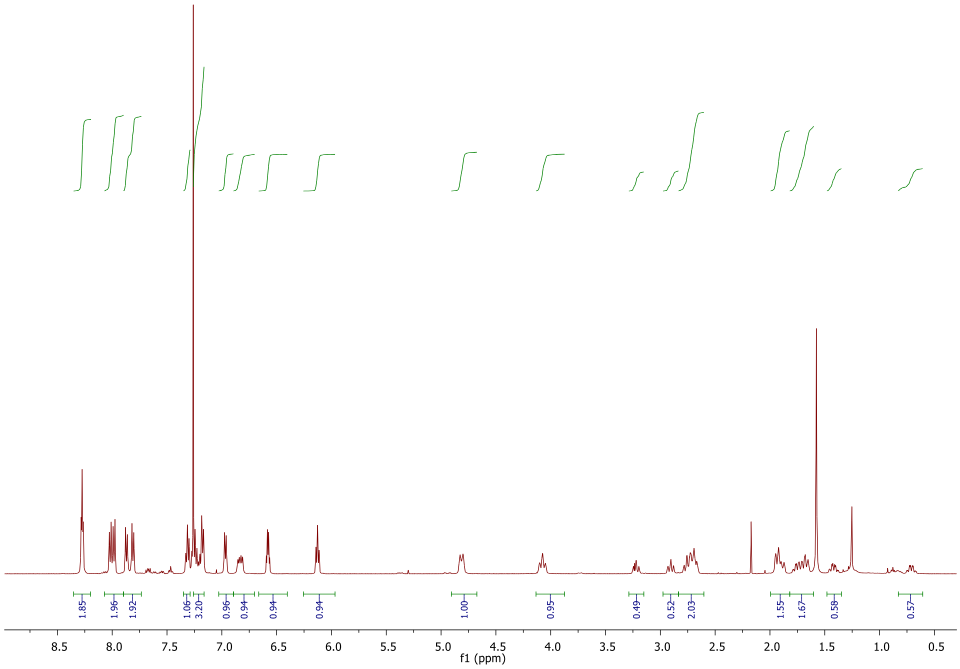
1.85
1.96
1.92
1.06
3.20
0.96
0.94
0.94
0.94
1.00
0.95
0.49
0.52
2.03
1.55
1.67
0.58
0.57
8.5 8.0 7.5 7.0 6.5 6.0 5.5 5.0 4.5 4.0 3.5 3.0 2.5 2.0 1.5 1.0 0.5
f1 (ppm)

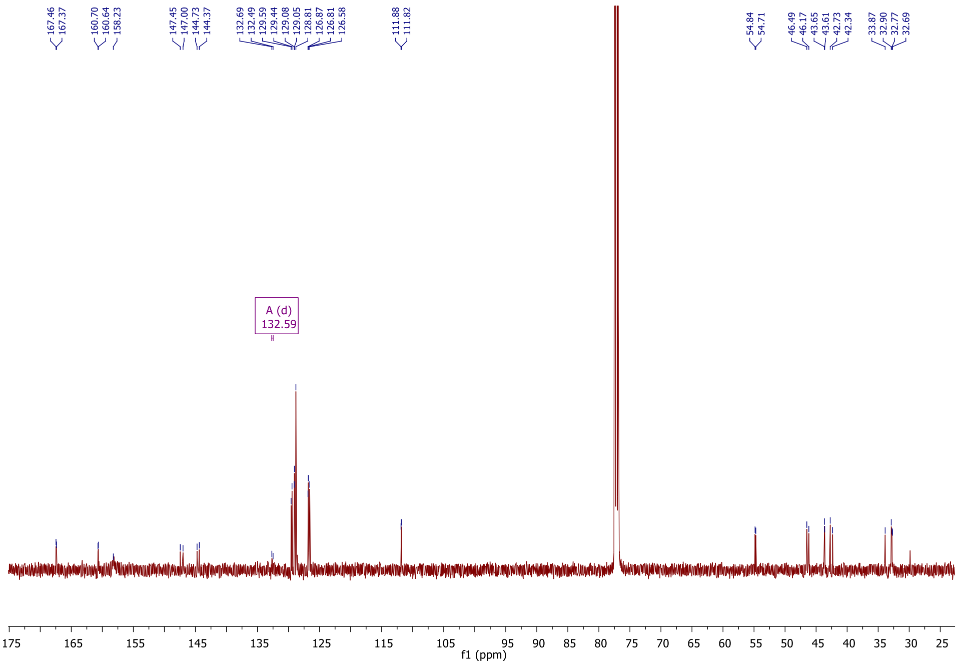
167.46
167.37
160.70
160.64
158.23
147.45
147.00
144.73
144.37
132.69
132.49
129.59
129.44
129.08
129.05
128.81
126.87
126.81
126.58
111.88
111.82
54.84
54.71
46.49
46.17
43.65
43.61
42.73
42.34
33.87
32.90
32.77
32.69
A (d)
132.59
175 165 155 145 135 125 115 105 95 90 85 80 75 70 65 60 55 50 45 40 35 30 25
f1 (ppm)

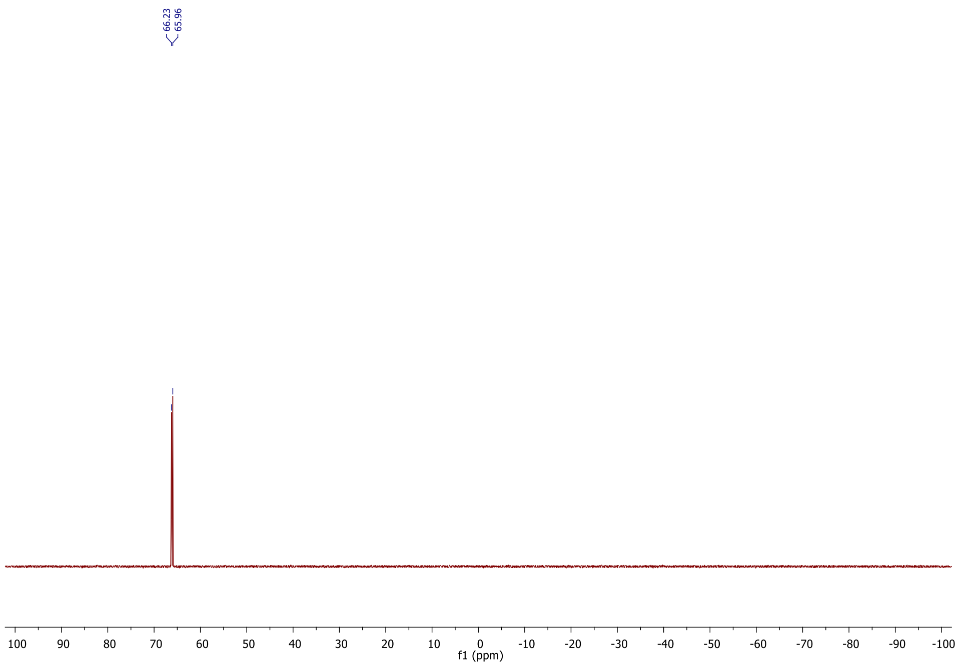
66.23
65.96
100 90 80 70 60 50 40 30 20 10 0 -10 -20 -30 -40 -50 -60 -70 -80 -90 -100
f1 (ppm)

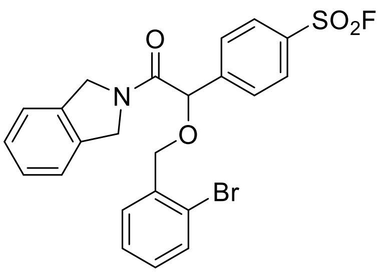
SO2F
O
N
O
Br

3-2

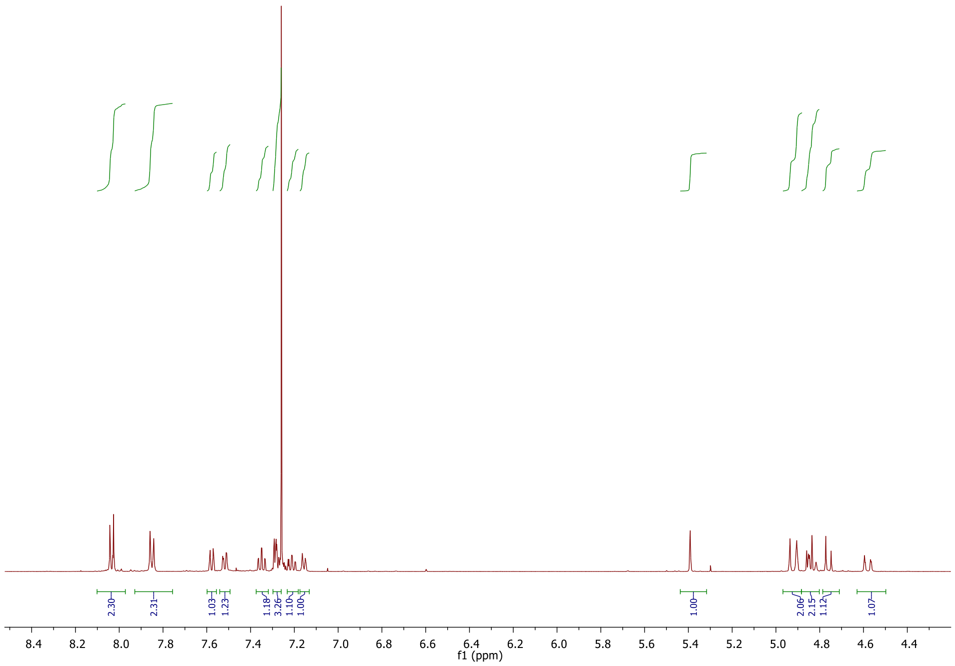
2.30
2.31
1.03
1.23
1.18
3.26
1.10
1.00
1.00
2.06
2.15
1.12
1.07
8.4 8.2 8.0 7.8 7.6 7.4 7.2 7.0 6.8 6.6 6.4 6.2 6.0 5.8 5.6 5.4 5.2 5.0 4.8 4.6 4.4
f1 (ppm)

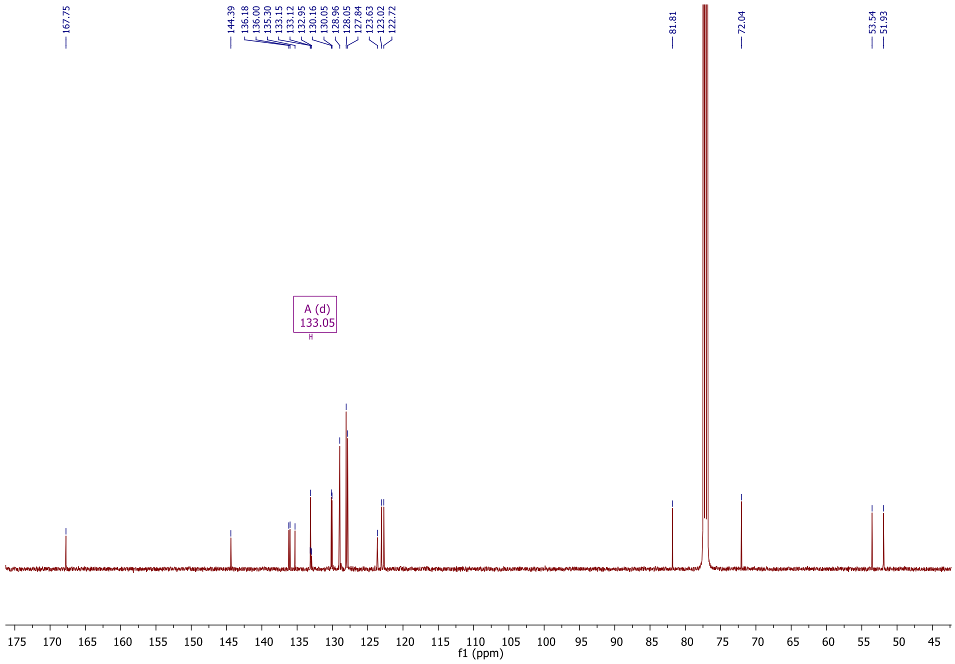
167.75
144.39
136.18
136.00
135.30
133.15
133.12
132.95
130.16
130.05
128.96
128.05
127.84
123.63
123.02
122.72
81.81
72.04
53.54
51.93
A (d)
133.05
175 170 165 160 155 150 145 140 135 130 125 120 115 110 105 100 95 90 85 80 75 70 65 60 55 50 45
f1 (ppm)

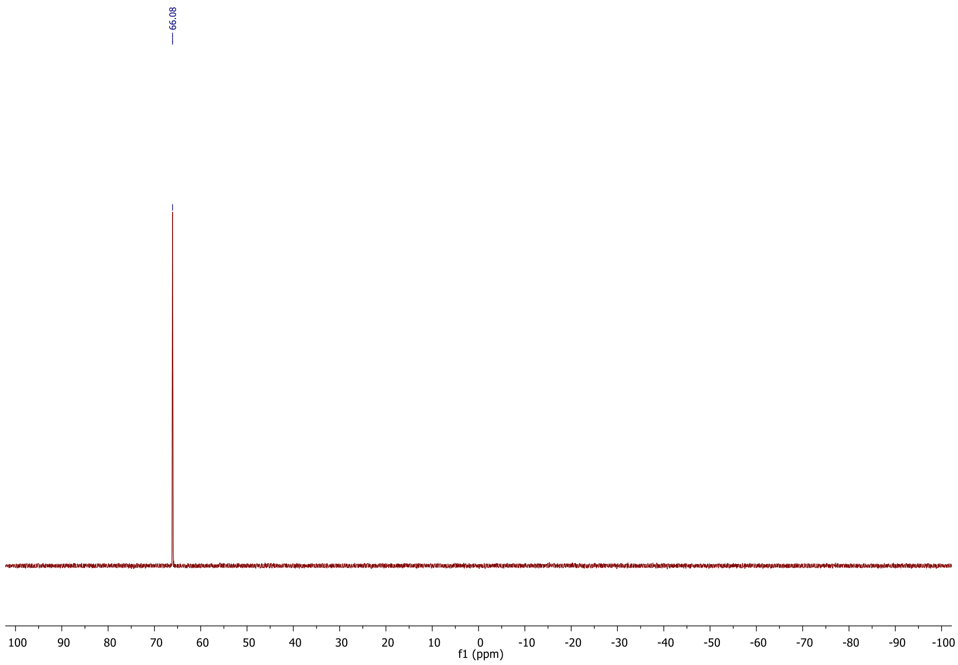

66.08
100 90 80 70 60 50 40 30 20 10 0 -10 -20 -30 -40 -50 -60 -70 -80 -90 -100
f1 (ppm)

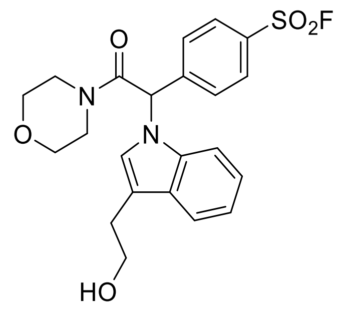
SO2F
O
N
O
N
HO

1-3a

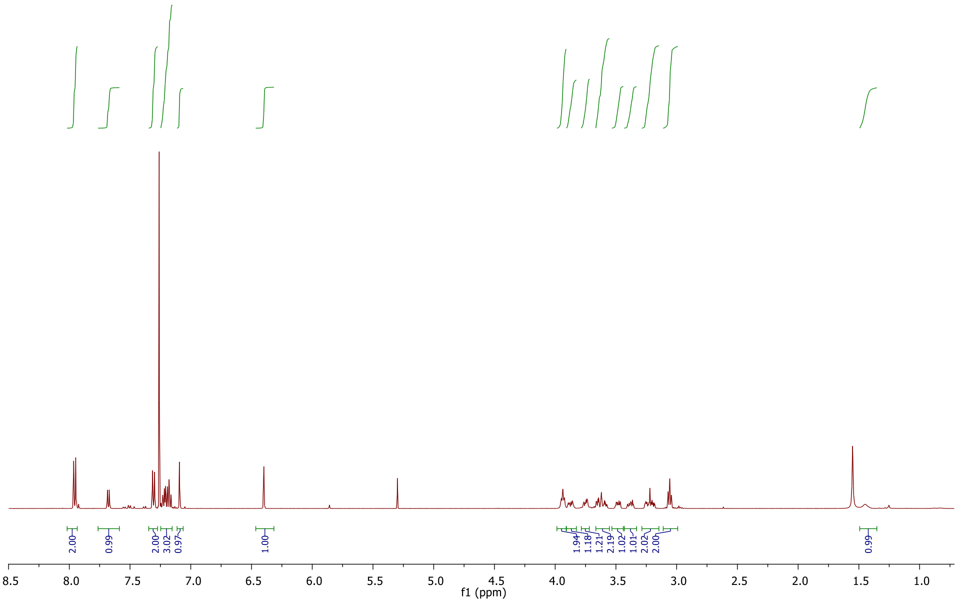
2.00
0.99
2.00
3.02
0.97
1.00
1.94
1.18
1.21
2.19
1.02
1.01
2.02
2.00
0.99
8.5 8.0 7.5 7.0 6.5 6.0 5.5 5.0 4.5 4.0 3.5 3.0 2.5 2.0 1.5 1.0
f1 (ppm)

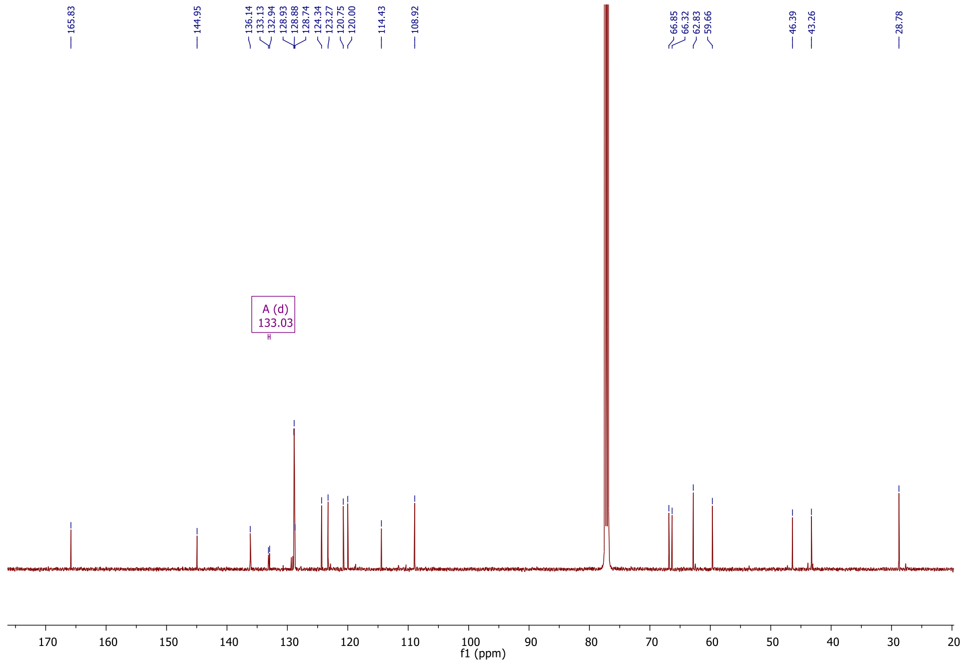
165.83
144.95
136.14
133.13
132.94
128.93
128.88
128.74
124.34
123.27
120.75
120.00
114.43
108.92
66.85
66.32
62.83
59.66
46.39
43.26
28.78
A (d)
133.03
170 160 150 140 130 120 110 100 90 80 70 60 50 40 30 20
f1 (ppm)

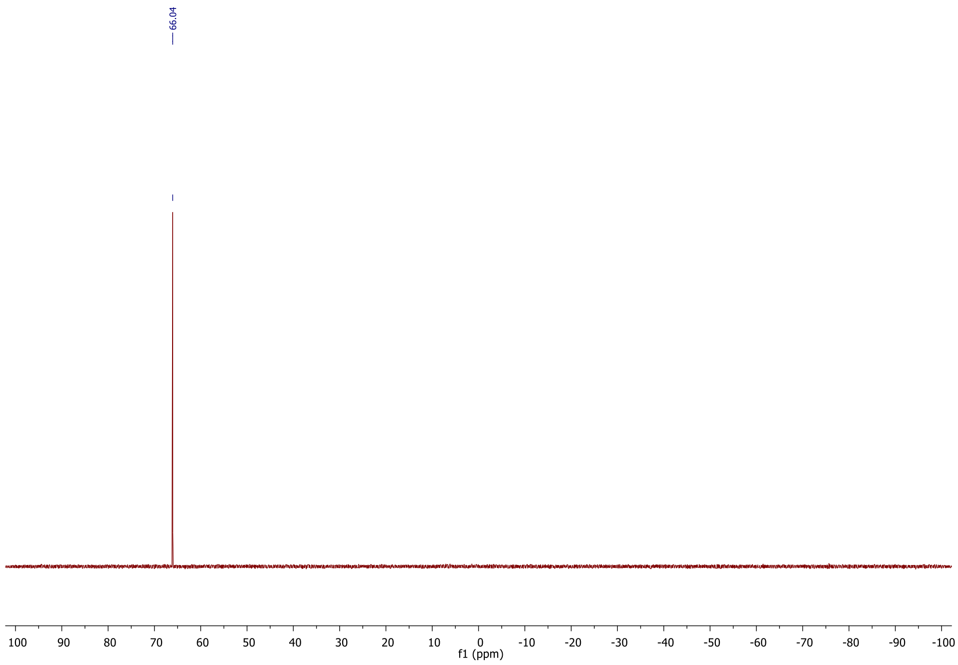

66.04
100
90
80
70
60
50
40
30
20
10
0
-10
-20
-30
-40
-50
-60
-70
-80
-90
-100
f1 (ppm)

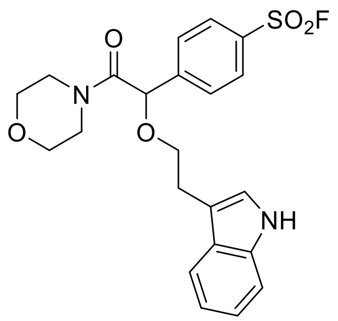
SO2F
O
N
O
O
NH

1-3b

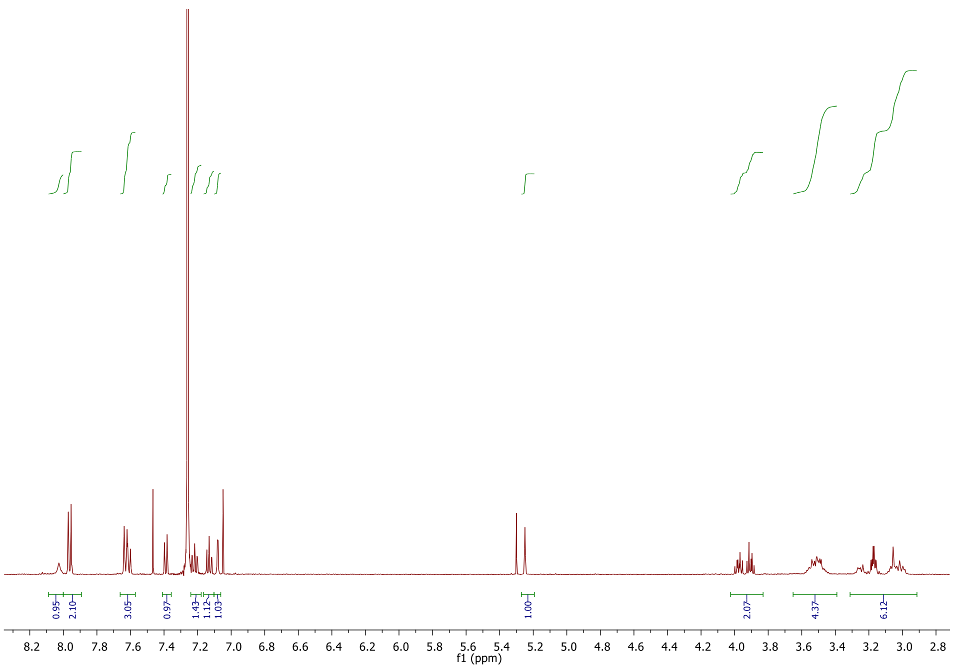
0.95
2.10
3.05
0.97
1.43
1.12
1.03
1.00
2.07
4.37
6.12
f1 (ppm)

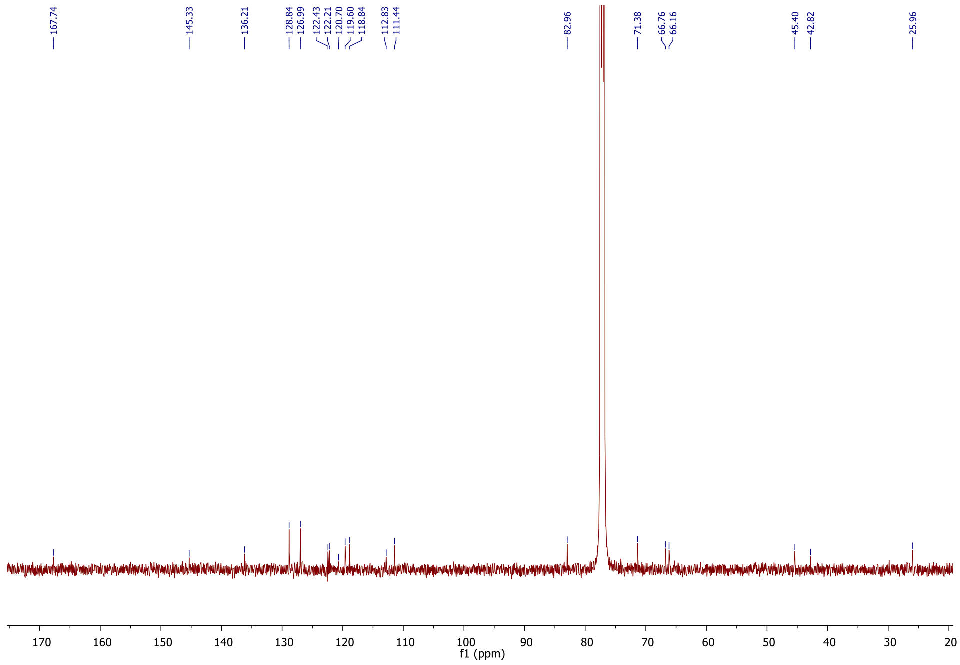
167.74
145.33
136.21
128.84
126.99
122.43
122.21
120.70
119.60
118.84
112.83
111.44
82.96
71.38
66.76
66.16
45.40
42.82
25.96
f1 (ppm)

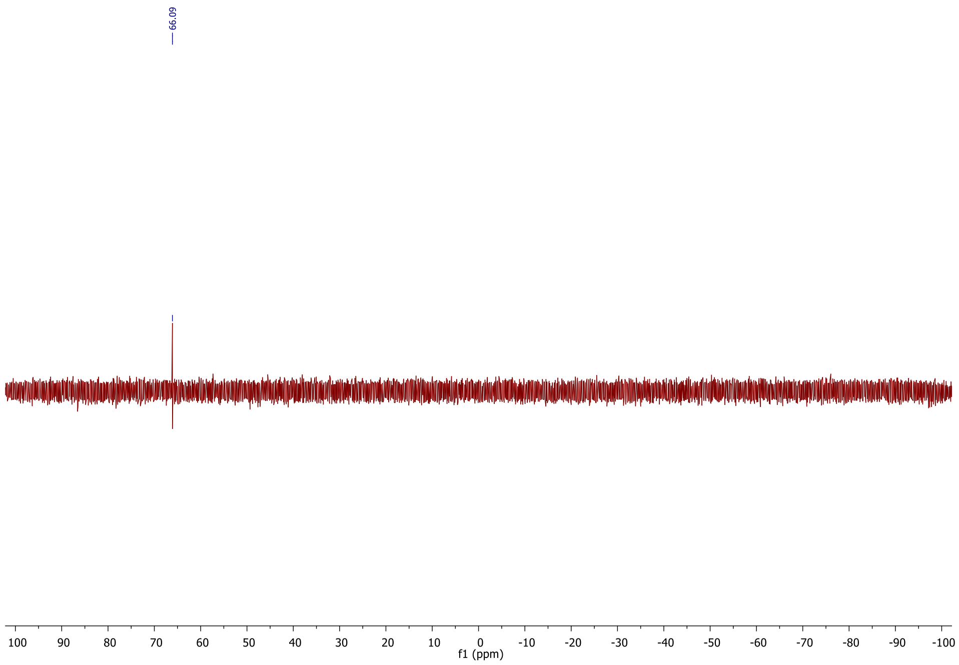

66.09
100 90 80 70 60 50 40 30 20 10 0 -10 -20 -30 -40 -50 -60 -70 -80 -90 -100
f1 (ppm)

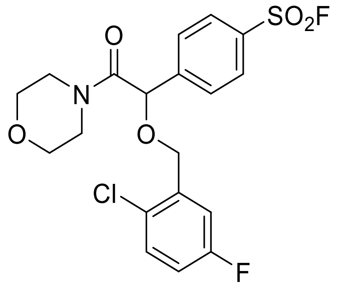

1-5

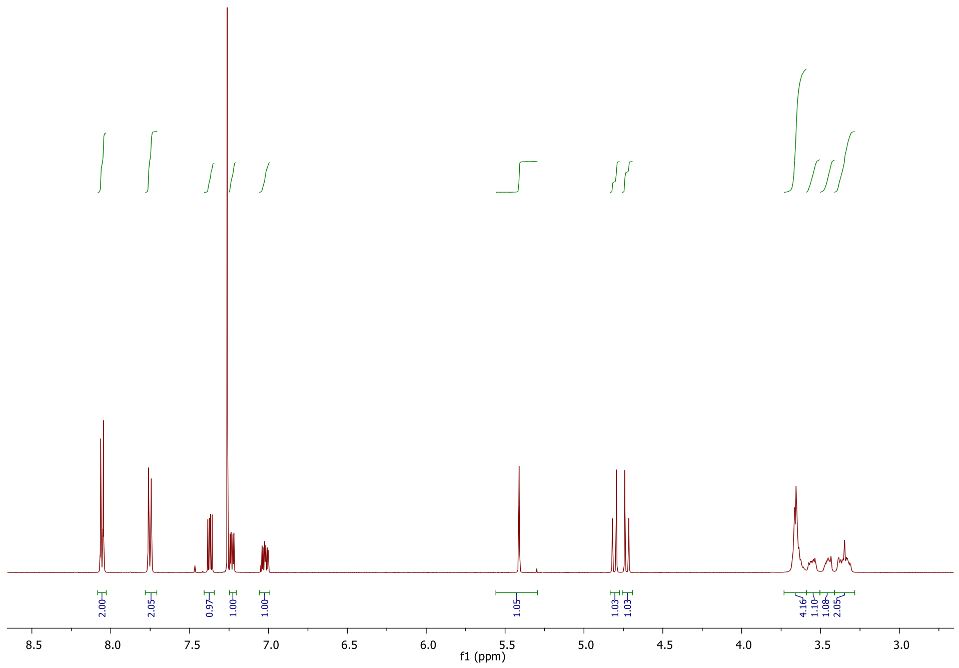

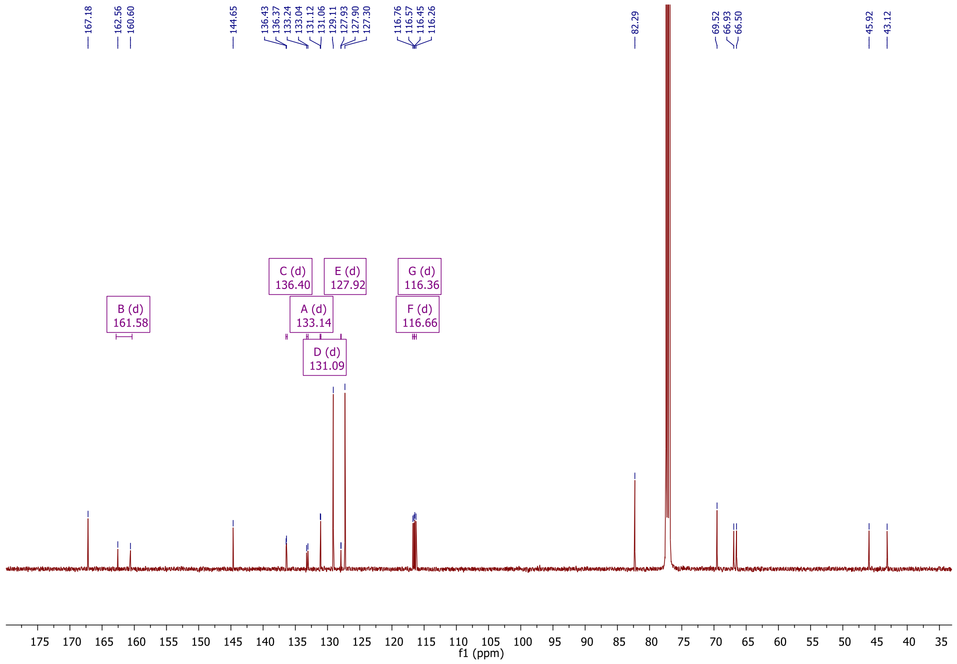

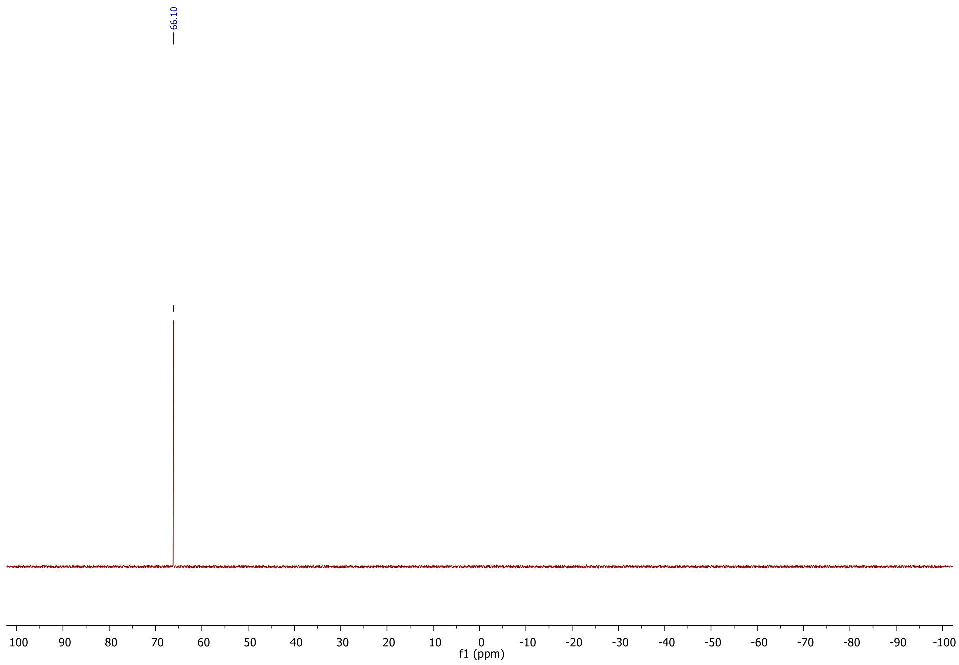
66.10
100 90 80 70 60 50 40 30 20 10 0 -10 -20 -30 -40 -50 -60 -70 -80 -90 -100
f1 (ppm)

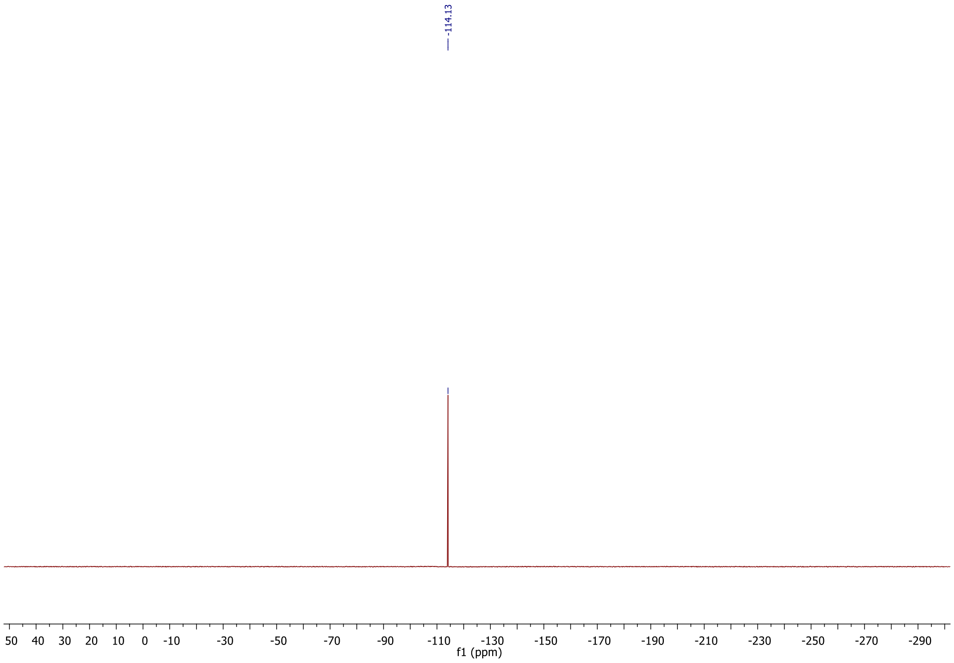
-114.13
50 40 30 20 10 0 -10 -30 -50 -70 -90 -110 -130 -150 -170 -190 -210 -230 -250 -270 -290
f1 (ppm)

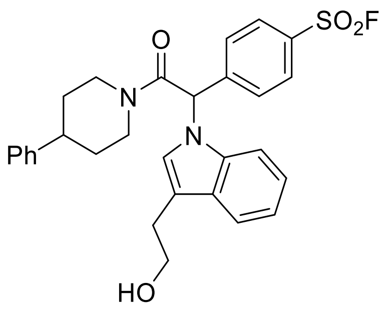
SO2F
O
N
N
Ph
HO

2-3a

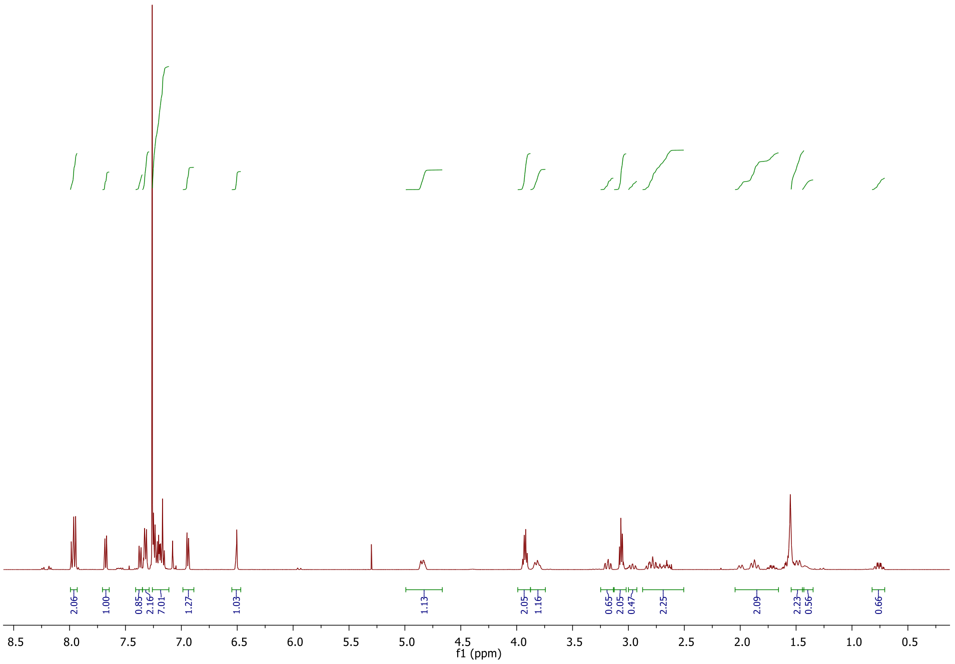
2.06
1.00
0.85
2.16
7.01
1.27
1.03
1.13
2.05
1.16
0.65
2.05
0.47
2.25
2.09
2.23
0.56
0.66
8.5 8.0 7.5 7.0 6.5 6.0 5.5 5.0 4.5 4.0 3.5 3.0 2.5 2.0 1.5 1.0 0.5
f1 (ppm)

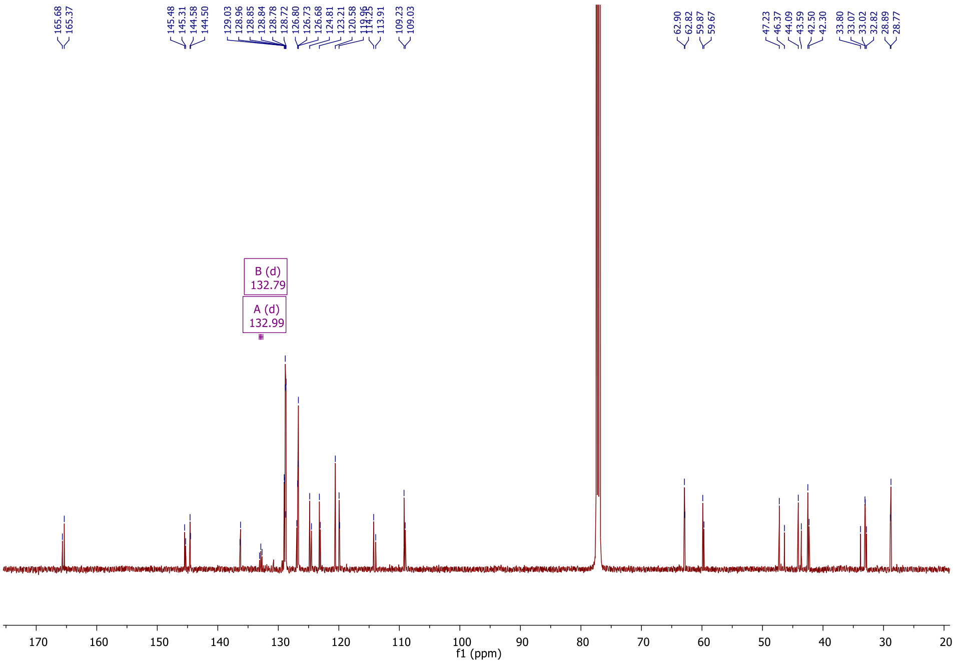
165.68
165.37
145.48
145.31
144.58
144.50
129.03
128.96
128.85
128.84
128.78
128.72
126.80
126.73
126.68
124.81
123.21
120.58
119.96
114.25
113.91
109.23
109.03
62.90
62.82
59.87
59.67
47.23
46.37
44.09
43.59
42.50
42.30
33.80
33.07
33.02
32.82
28.89
28.77
B (d)
132.79
A (d)
132.99
170 160 150 140 130 120 110 100 90 80 70 60 50 40 30 20
f1 (ppm)

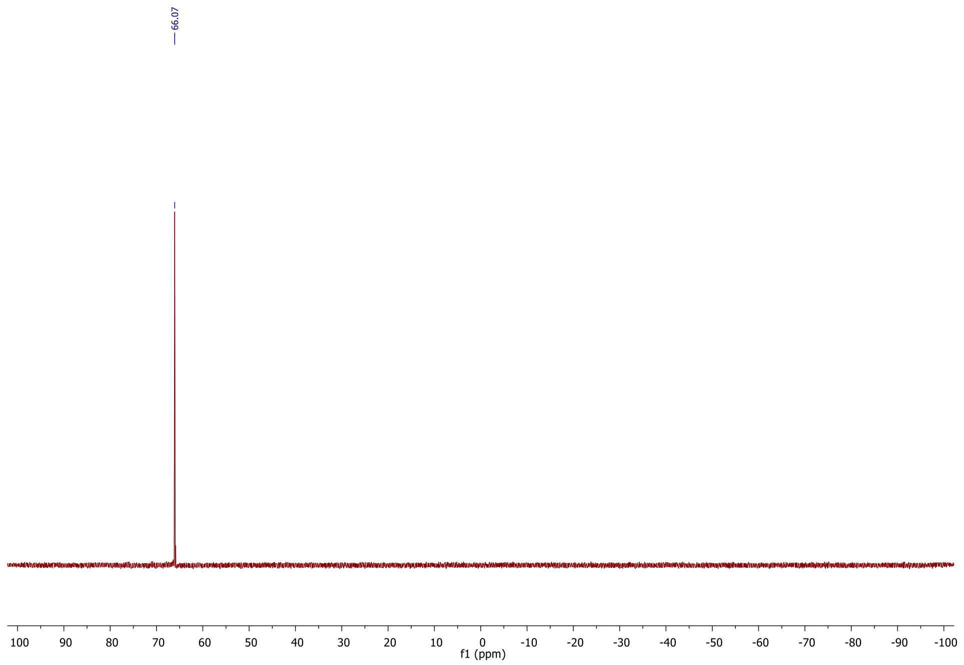
66.07
100
90
80
70
60
50
40
30
20
10
0
-10
-20
-30
-40
-50
-60
-70
-80
-90
-100
f1 (ppm)

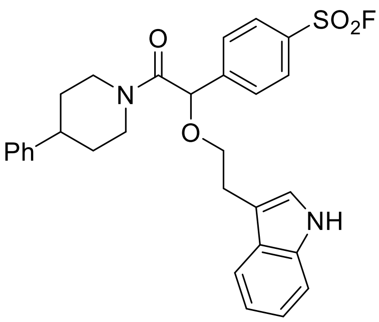

SO2F
O
N
Ph
O
NH

2-3b

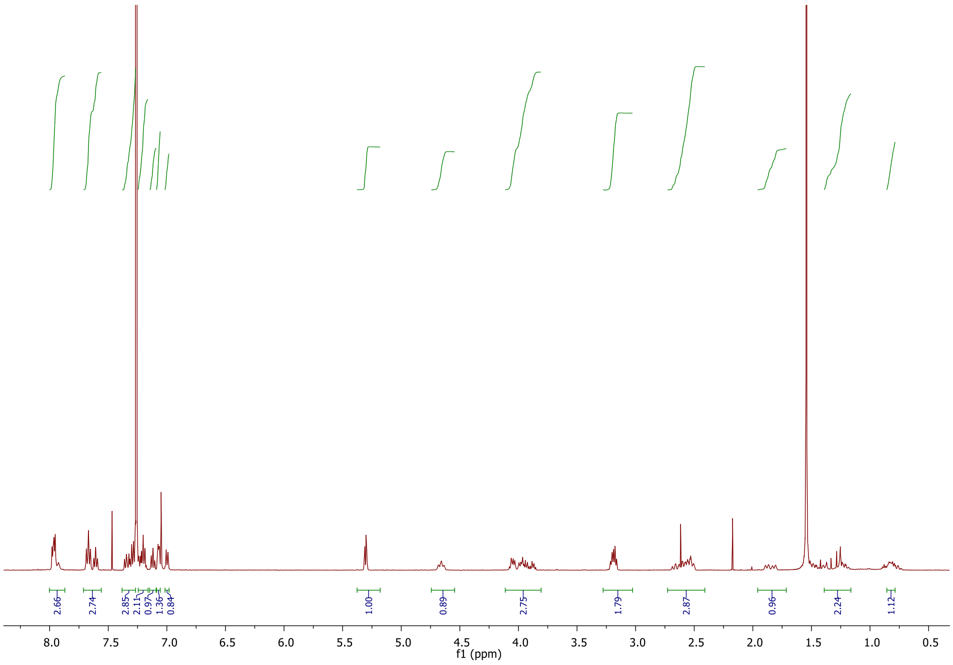

2.66
2.74
2.85
2.11
0.97
1.36
0.84
1.00
0.89
2.75
1.79
2.87
0.96
2.24
1.12
8.0 7.5 7.0 6.5 6.0 5.5 5.0 4.5 4.0 3.5 3.0 2.5 2.0 1.5 1.0 0.5
f1 (ppm)

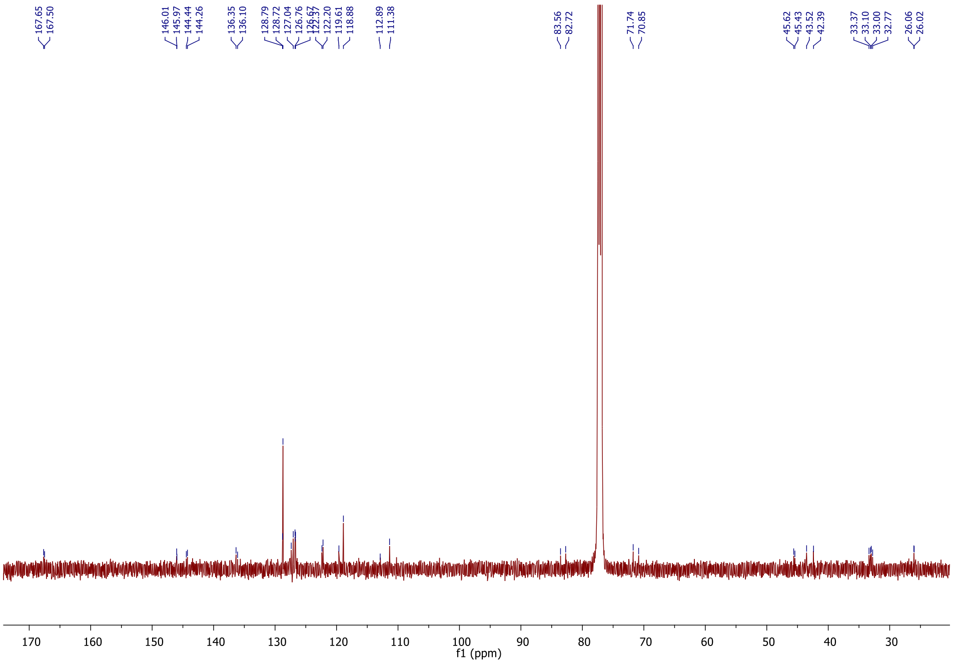

167.65
167.50
146.01
145.97
144.44
144.26
136.35
136.10
128.79
128.72
127.04
126.76
126.67
122.37
122.20
119.61
118.88
112.89
111.38
83.56
82.72
71.74
70.85
45.62
45.43
43.52
42.39
33.37
33.10
33.00
32.77
26.06
26.02
170 160 150 140 130 120 110 100 90 80 70 60 50 40 30
f1 (ppm)

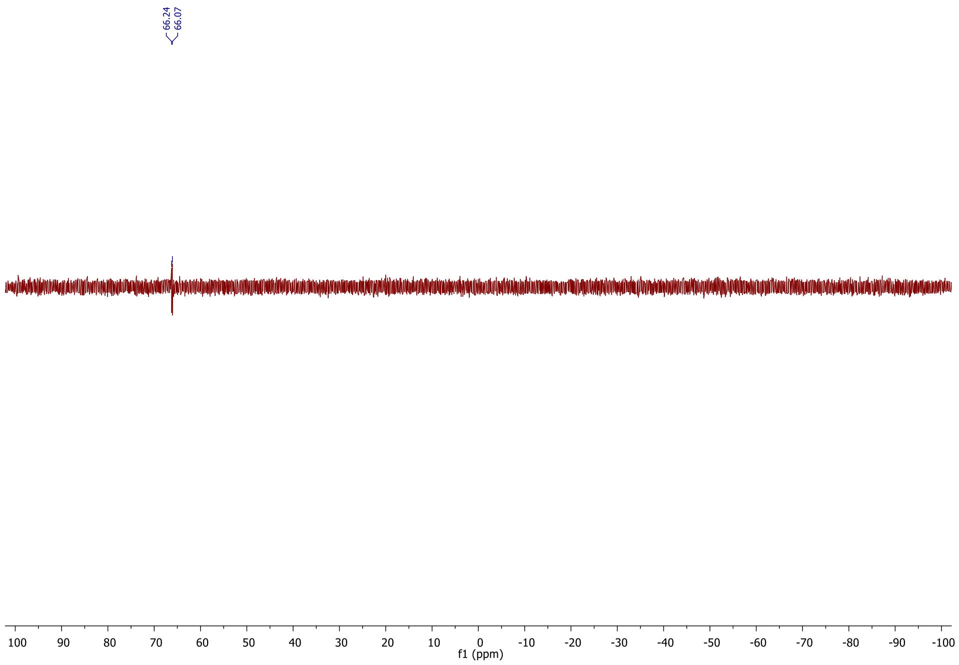
66.24
66.07
100
90
80
70
60
50
40
30
20
10
0
-10
-20
-30
-40
-50
-60
-70
-80
-90
-100
f1 (ppm)

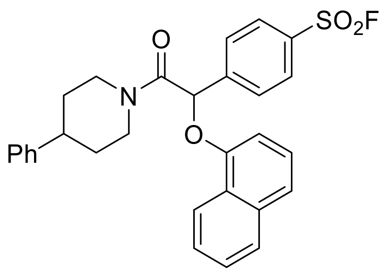

2-4

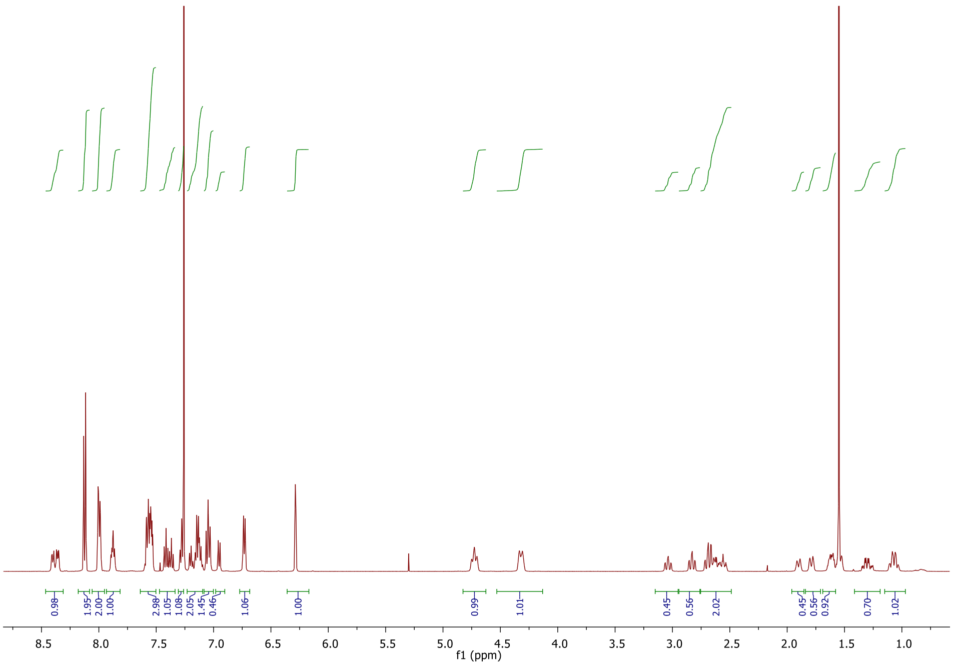

0.98
1.95
2.00
1.00
2.98
1.05
1.08
2.05
1.45
0.46
1.06
1.00
0.99
1.01
0.45
0.56
2.02
0.45
0.56
0.92
0.70
1.02
f1 (ppm)

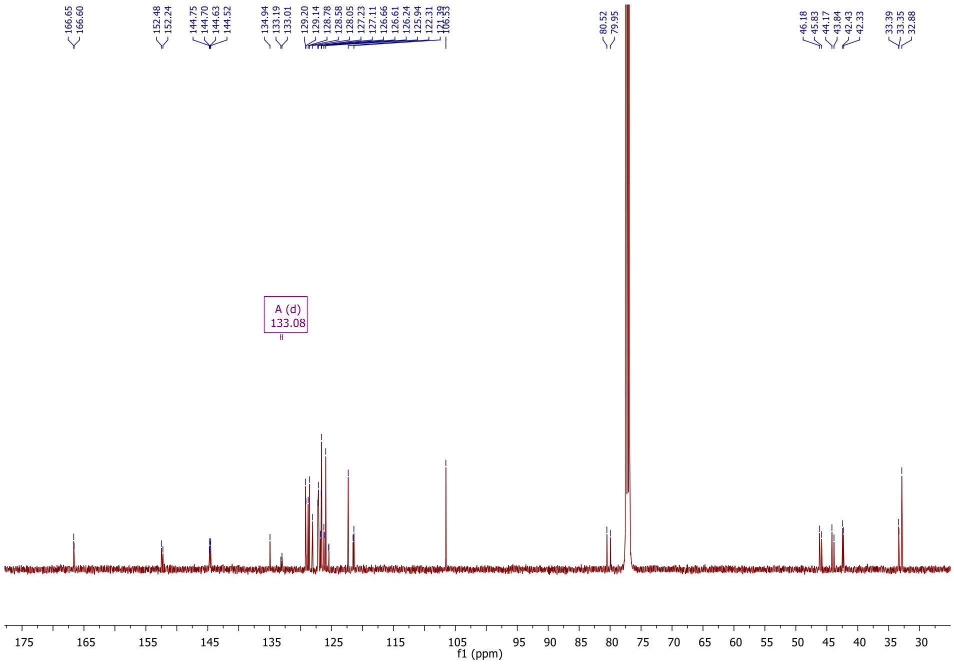

166.65
166.60
152.48
152.24
144.75
144.70
144.63
144.52
134.94
133.19
133.01
129.20
129.14
128.78
128.58
128.05
127.23
127.11
126.66
126.61
126.24
125.94
122.31
106.53
80.52
79.95
46.18
45.83
44.17
43.84
42.43
42.33
33.39
33.35
32.88
A (d)
133.08
f1 (ppm)

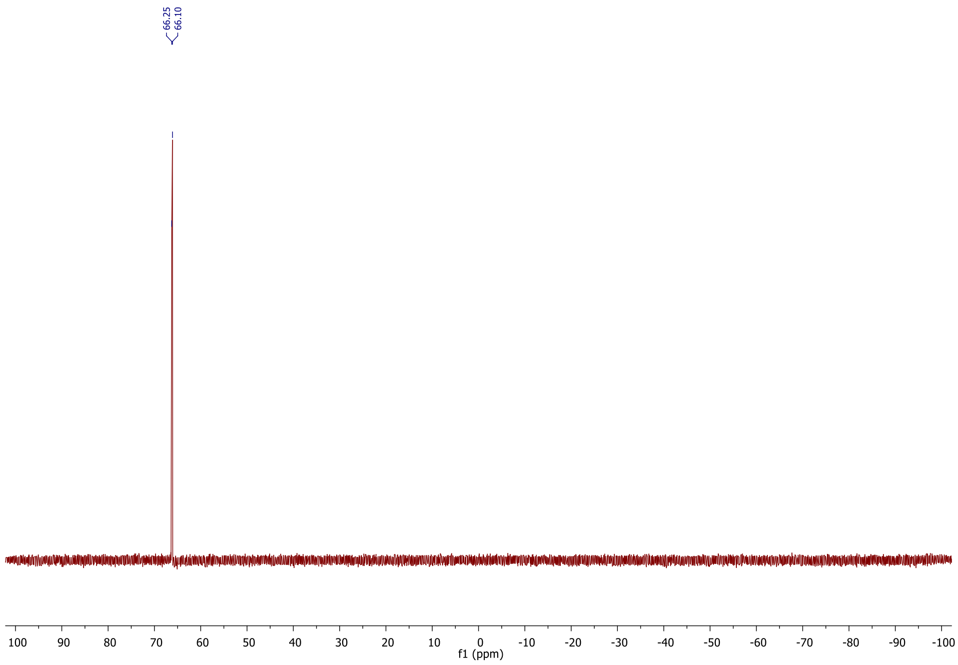
66.25
66.10
100
90
80
70
60
50
40
30
20
10
0
-10
-20
-30
-40
-50
-60
-70
-80
-90
-100
f1 (ppm)

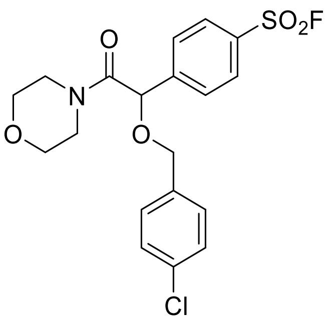

SO2F
O
N
O
O
Cl

1-1

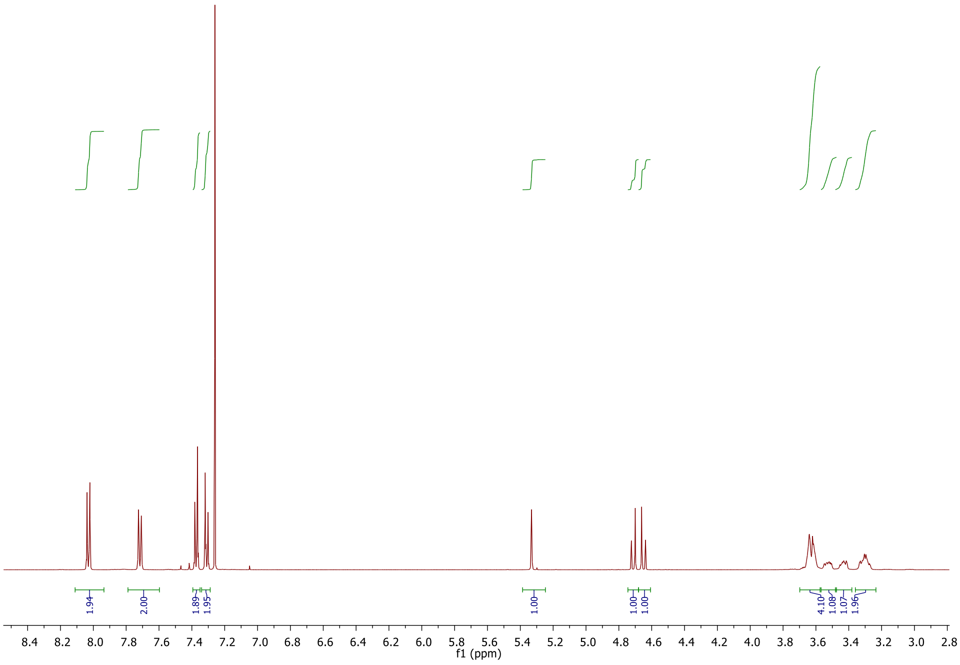

1.94
2.00
1.89
1.95
1.00
1.00
1.00
4.10
1.08
1.07
1.96
8.4 8.2 8.0 7.8 7.6 7.4 7.2 7.0 6.8 6.6 6.4 6.2 6.0 5.8 5.6 5.4 5.2 5.0 4.8 4.6 4.4 4.2 4.0 3.8 3.6 3.4 3.2 3.0 2.8
f1 (ppm)

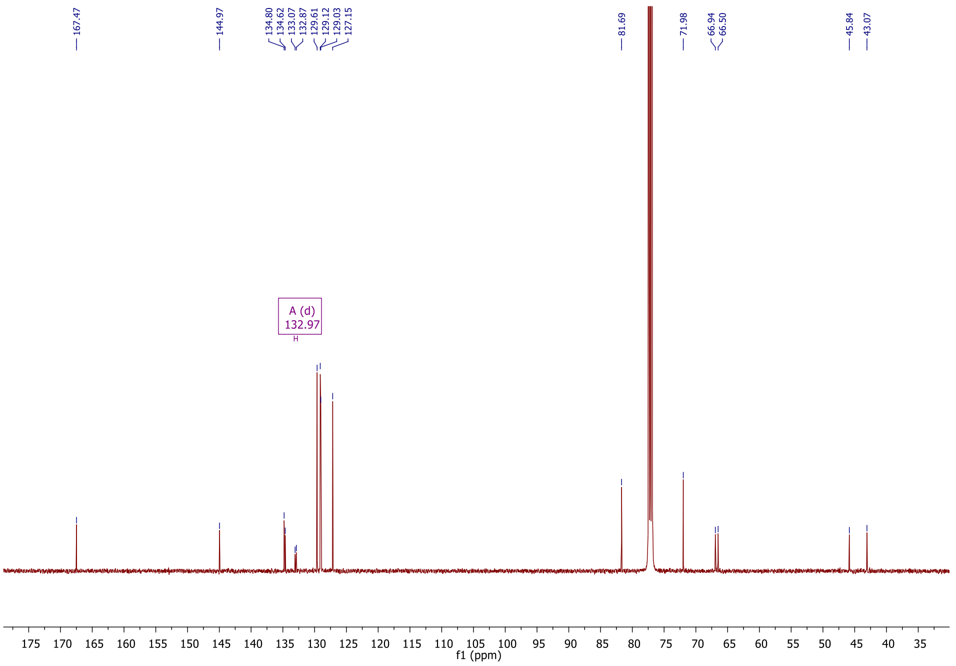

167.47
144.97
134.80
134.62
133.07
132.87
129.61
129.12
129.03
127.15
81.69
71.98
66.94
66.50
45.84
43.07
A (d)
132.97
H
175 170 165 160 155 150 145 140 135 130 125 120 115 110 105 100 95 90 85 80 75 70 65 60 55 50 45 40 35
f1 (ppm)

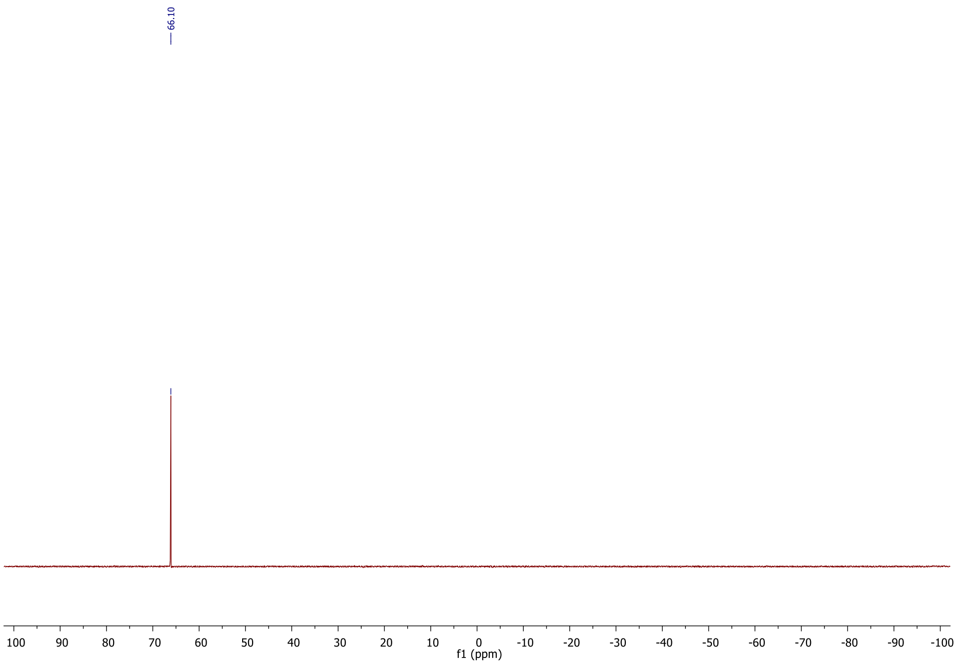
66.10
100 90 80 70 60 50 40 30 20 10 0 -10 -20 -30 -40 -50 -60 -70 -80 -90 -100
f1 (ppm)

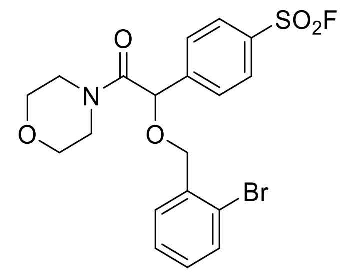
SO2F
O
N
O
O
Br

1-2

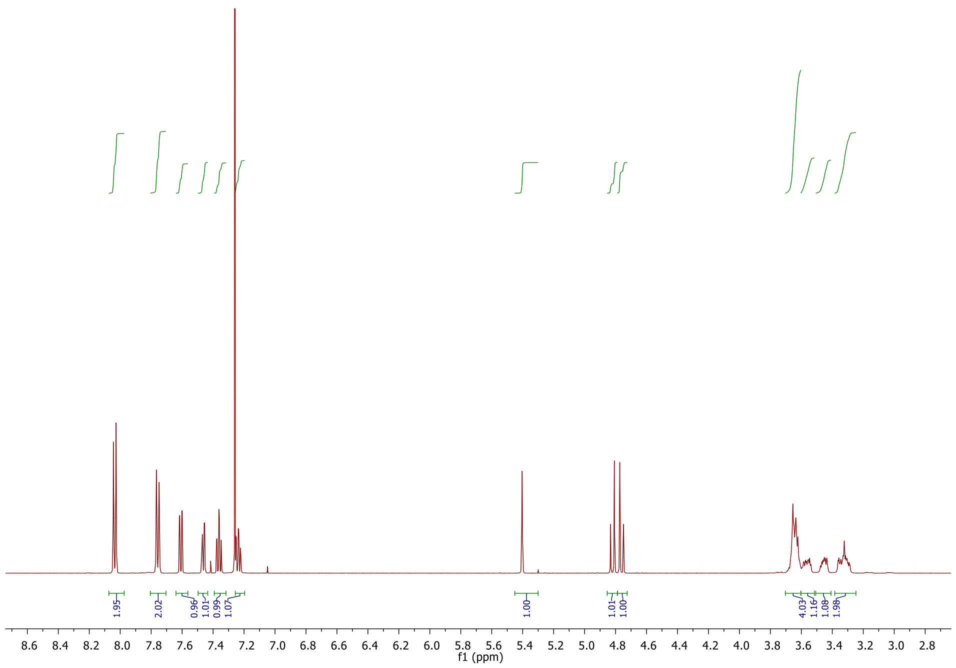
1.95
2.02
0.96
1.01
0.99
1.07
1.00
1.01
1.00
4.03
1.16
1.08
1.98
8.6 8.4 8.2 8.0 7.8 7.6 7.4 7.2 7.0 6.8 6.6 6.4 6.2 6.0 5.8 5.6 5.4 5.2 5.0 4.8 4.6 4.4 4.2 4.0 3.8 3.6 3.4 3.2 3.0 2.8
f1 (ppm)

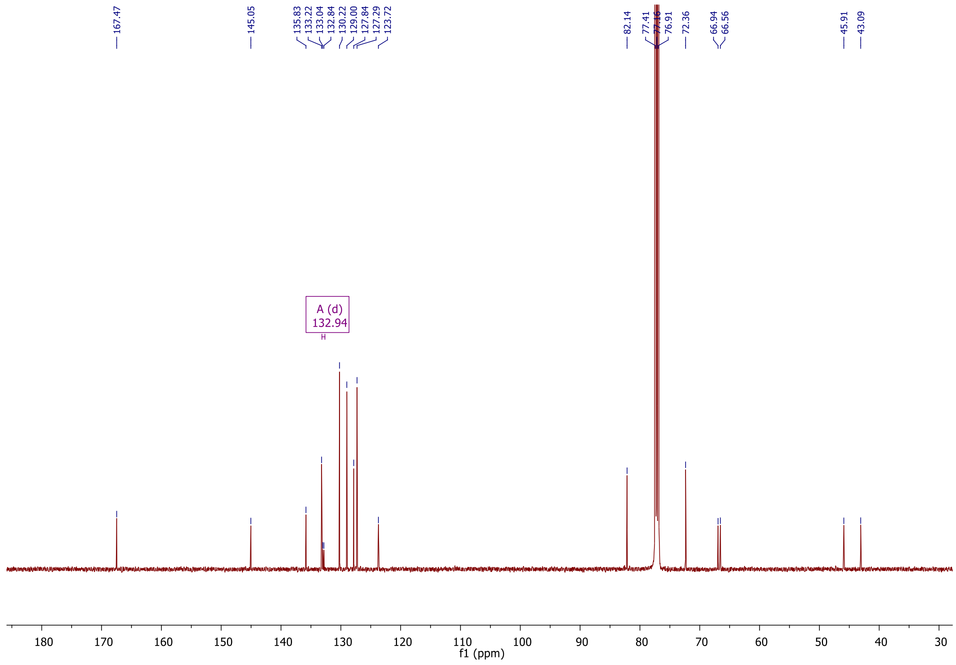
167.47
145.05
135.83
133.22
133.04
132.84
130.22
129.00
127.84
127.29
123.72
82.14
77.41
77.16
76.91
72.36
66.94
66.56
45.91
43.09
A (d)
132.94
180 170 160 150 140 130 120 110 100 90 80 70 60 50 40 30
f1 (ppm)

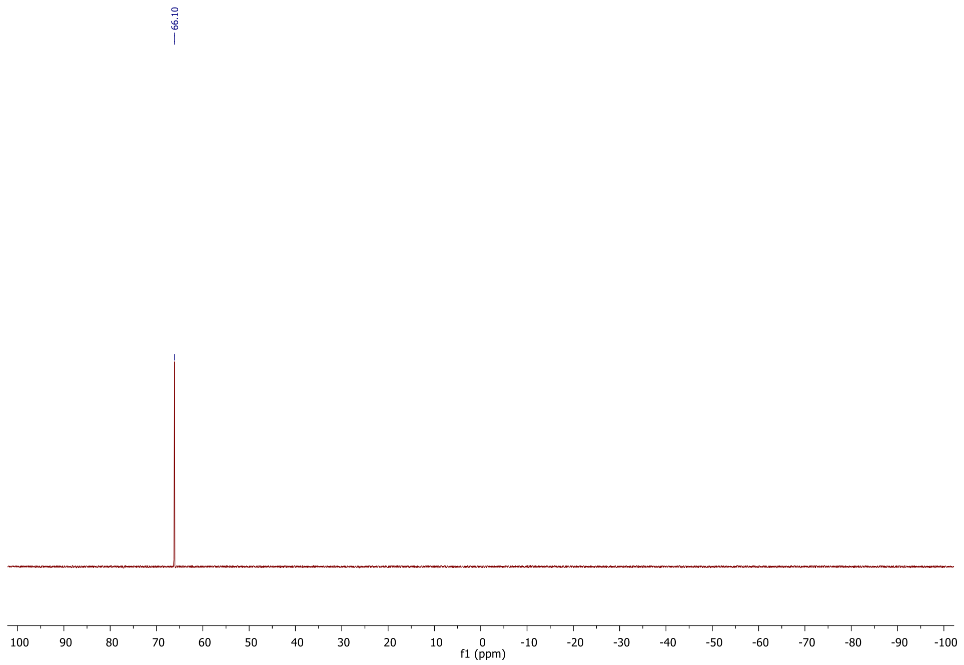

66.10
100
90
80
70
60
50
40
30
20
10
0
-10
-20
-30
-40
-50
-60
-70
-80
-90
-100
f1 (ppm)

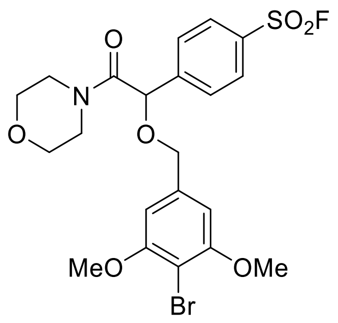
SO2F
O
N
O
O
MeO
OMe
Br

1-8

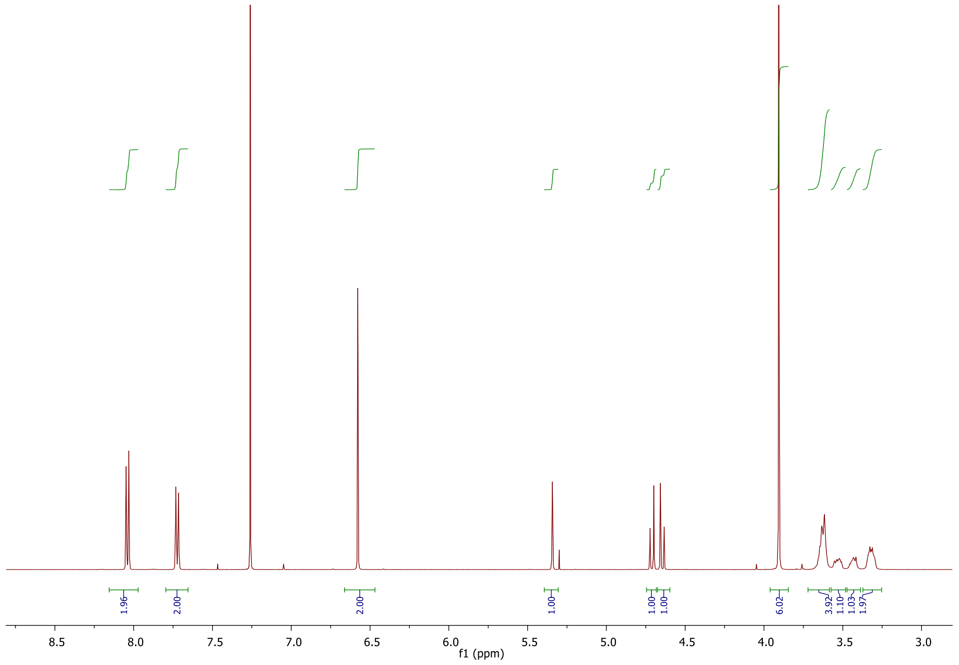
1.96
2.00
2.00
1.00
1.00
1.00
6.02
3.92
1.10
1.03
1.97
8.5
8.0
7.5
7.0
6.5
6.0
5.5
5.0
4.5
4.0
3.5
3.0
f1 (ppm)

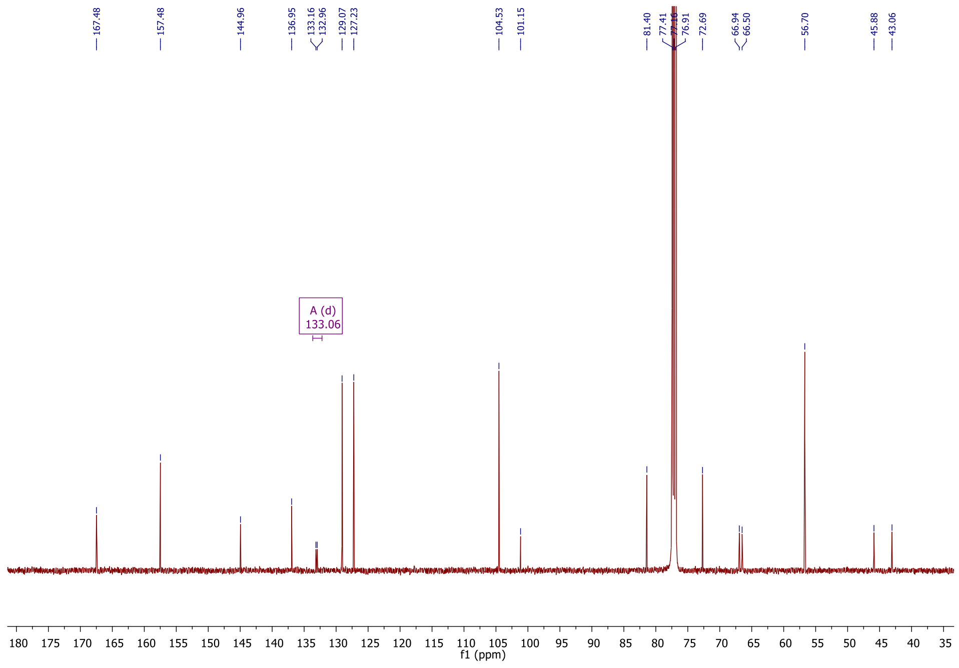
167.48
157.48
144.96
136.95
133.16
132.96
129.07
127.23
104.53
101.15
81.40
77.41
77.16
76.91
72.69
66.94
66.50
56.70
45.88
43.06
A (d)
133.06
180
175
170
165
160
155
150
145
140
135
130
125
120
115
110
105
100
95
90
85
80
75
70
65
60
55
50
45
40
35
f1 (ppm)

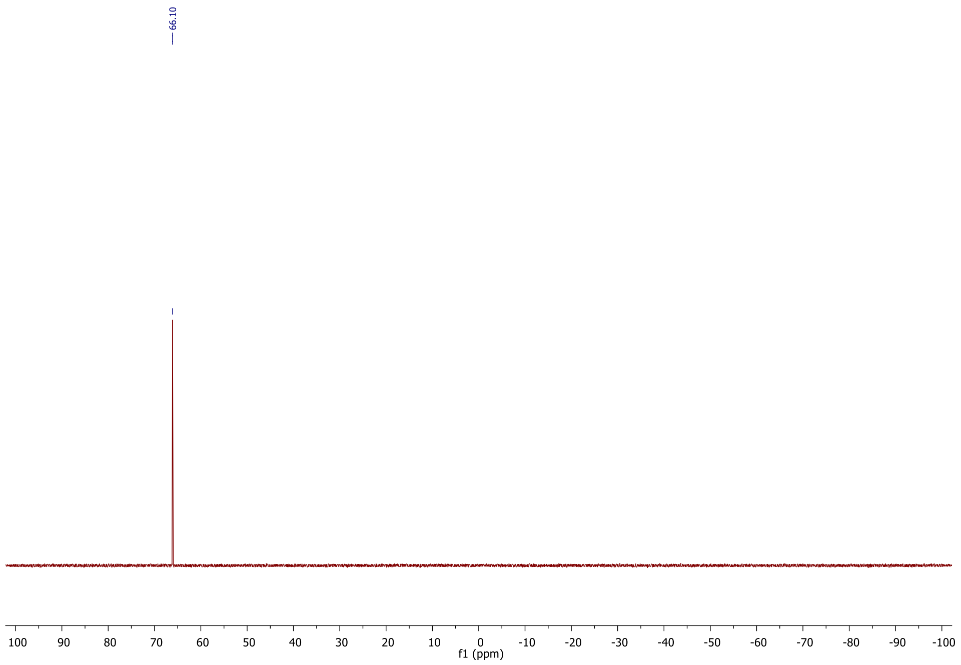
66.10
100
90
80
70
60
50
40
30
20
10
0
-10
-20
-30
-40
-50
-60
-70
-80
-90
-100
f1 (ppm)

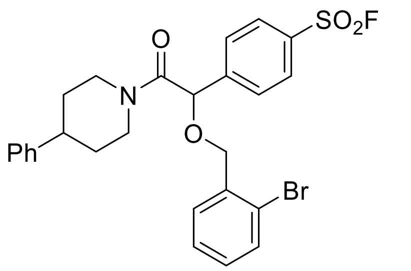

SO2F
O
N
Ph
O
Br

2-2

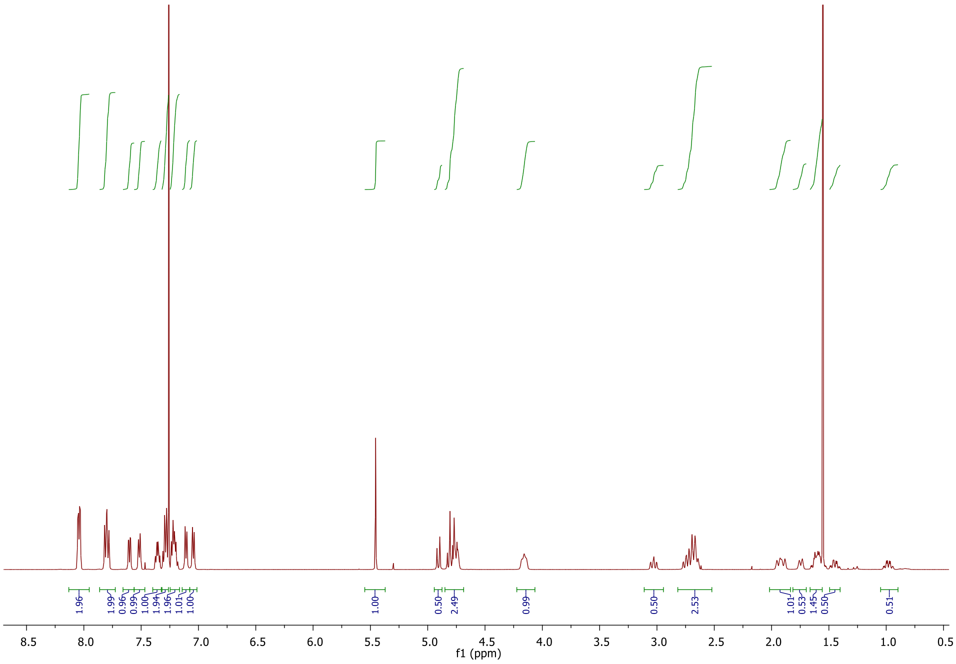

1.96
1.99
0.96
0.99
1.00
1.94
1.96
1.01
1.00
1.00
0.50
2.49
0.99
0.50
2.53
1.01
0.53
1.45
0.50
0.51
8.5 8.0 7.5 7.0 6.5 6.0 5.5 5.0 4.5 4.0 3.5 3.0 2.5 2.0 1.5 1.0 0.5
f1 (ppm)

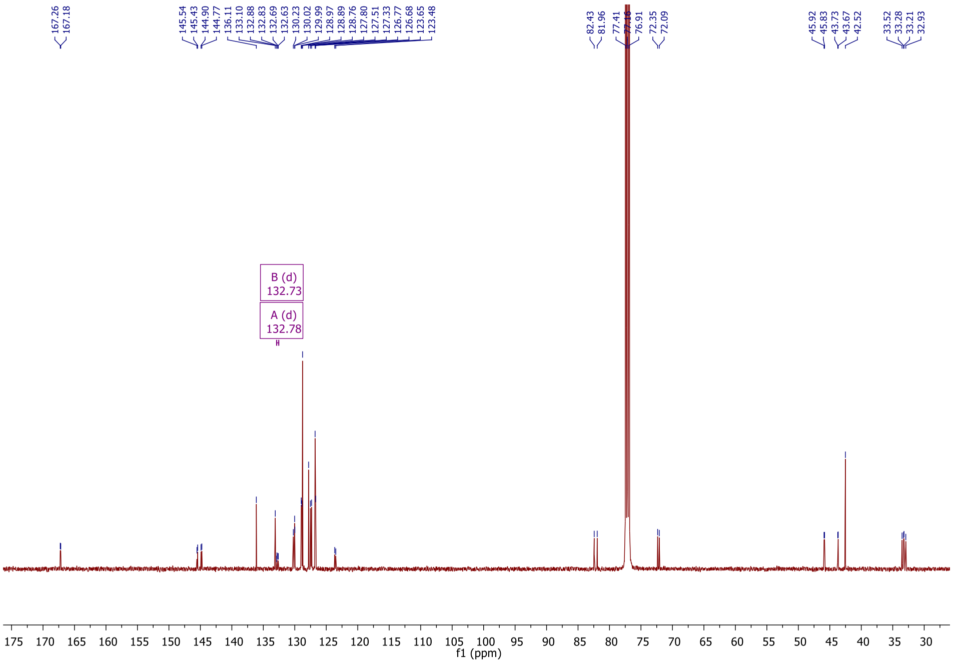

167.26
167.18
145.54
145.43
144.90
144.77
136.11
133.10
132.88
132.83
132.69
132.63
130.23
130.02
129.99
128.97
128.89
128.76
127.80
127.51
127.33
126.77
126.68
123.65
123.48
82.43
81.96
77.41
77.16
76.91
72.35
72.09
45.92
45.83
43.73
43.67
42.52
33.52
33.28
33.21
32.93
B (d)
132.73
A (d)
132.78
175 170 165 160 155 150 145 140 135 130 125 120 115 110 105 100 95 90 85 80 75 70 65 60 55 50 45 40 35 30
f1 (ppm)

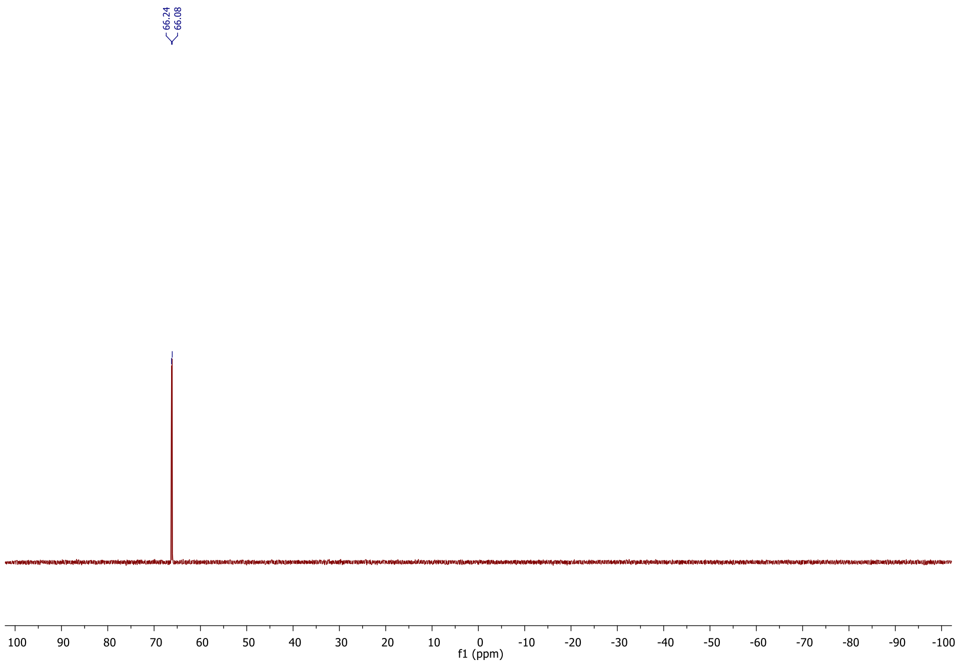

66.24
66.08
100 90 80 70 60 50 40 30 20 10 0 -10 -20 -30 -40 -50 -60 -70 -80 -90 -100
f1 (ppm)

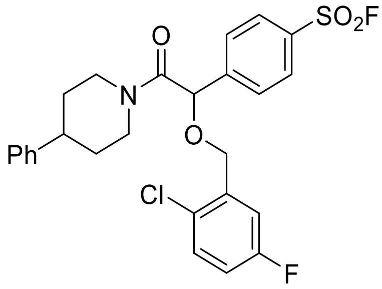

2-5

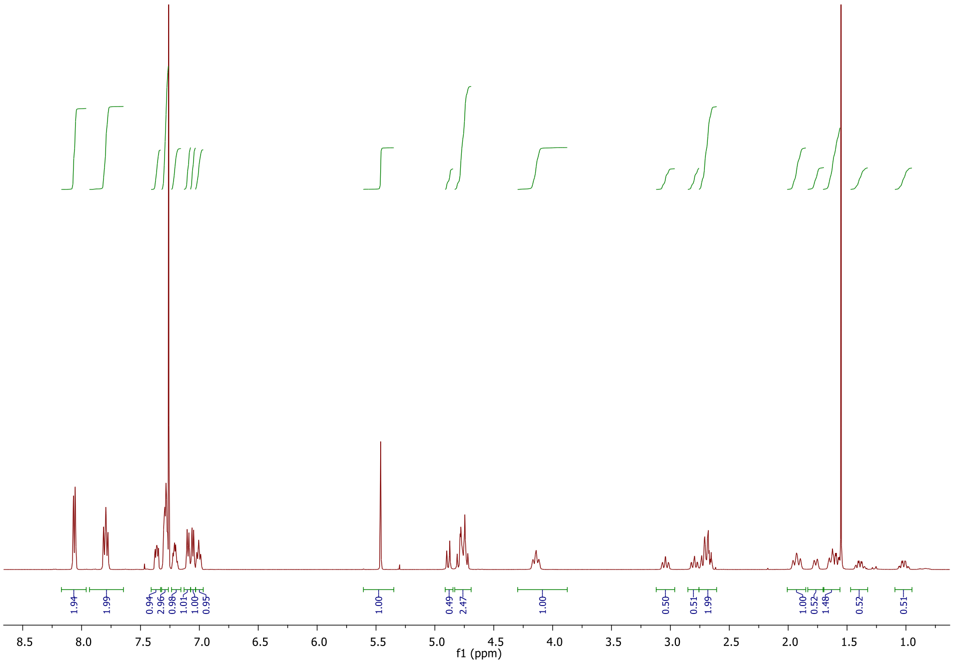

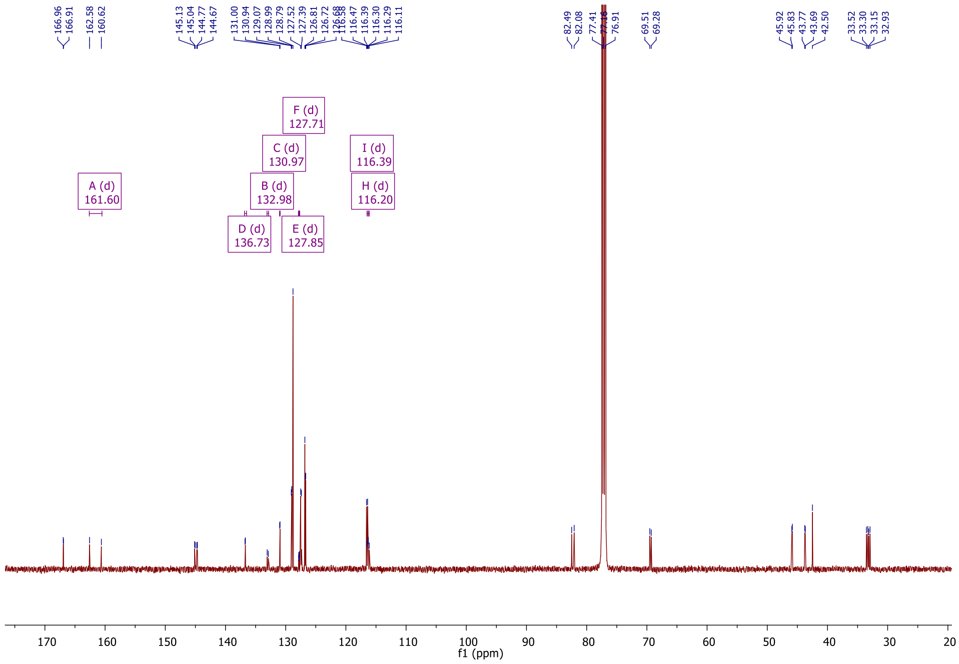

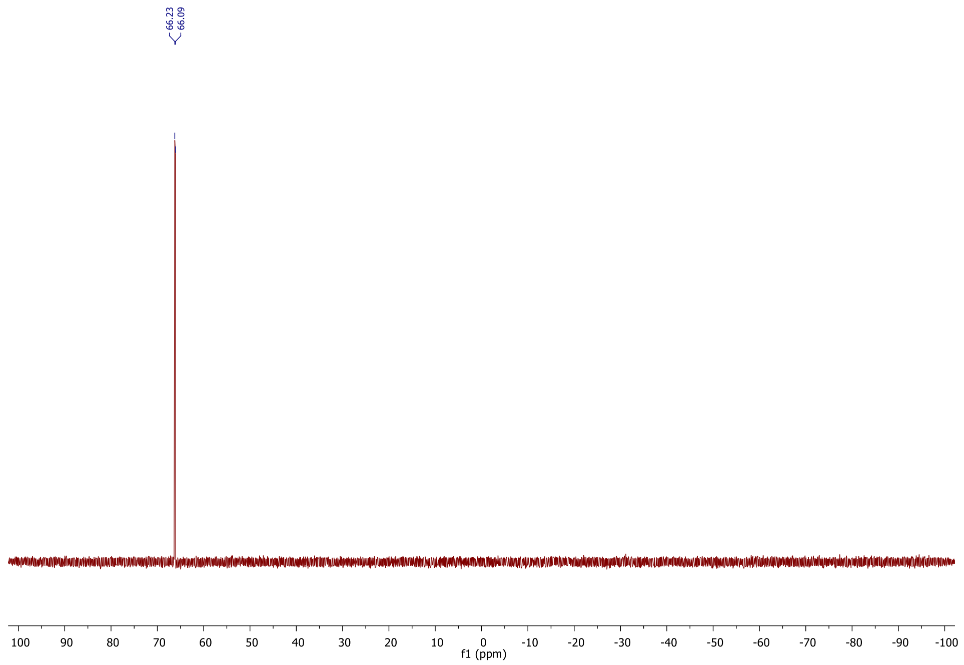
66.23
66.09
100 90 80 70 60 50 40 30 20 10 0 -10 -20 -30 -40 -50 -60 -70 -80 -90 -100
f1 (ppm)

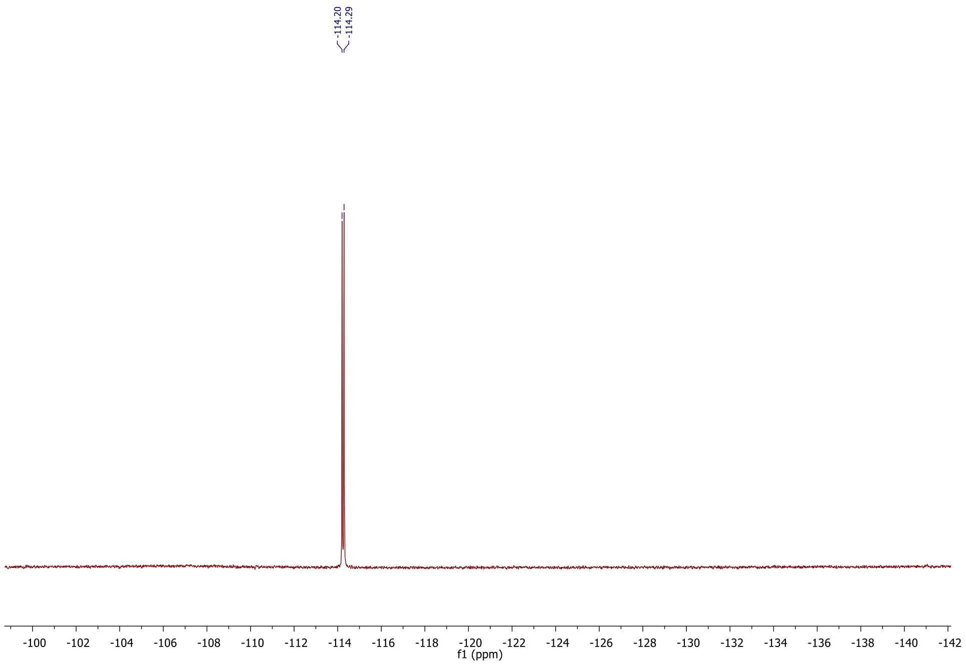
-114.20
-114.29
-100 -102 -104 -106 -108 -110 -112 -114 -116 -118 -120 -122 -124 -126 -128 -130 -132 -134 -136 -138 -140 -142
f1 (ppm)

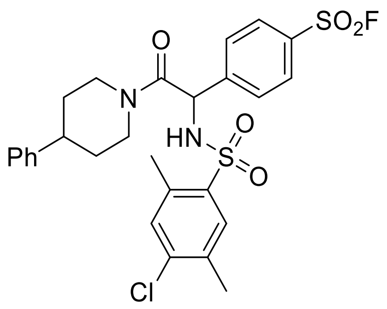

2-6

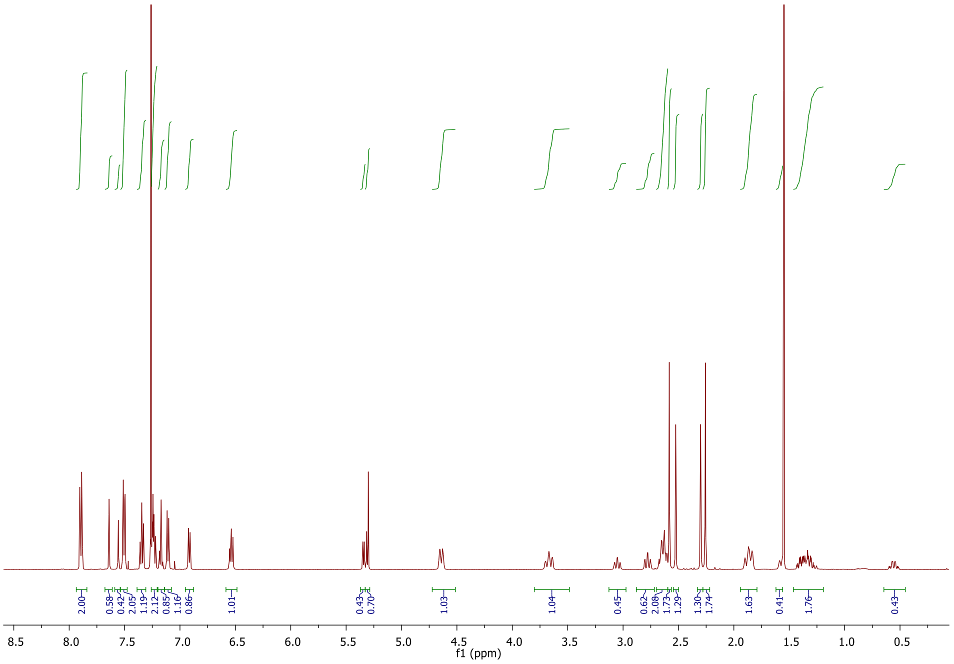

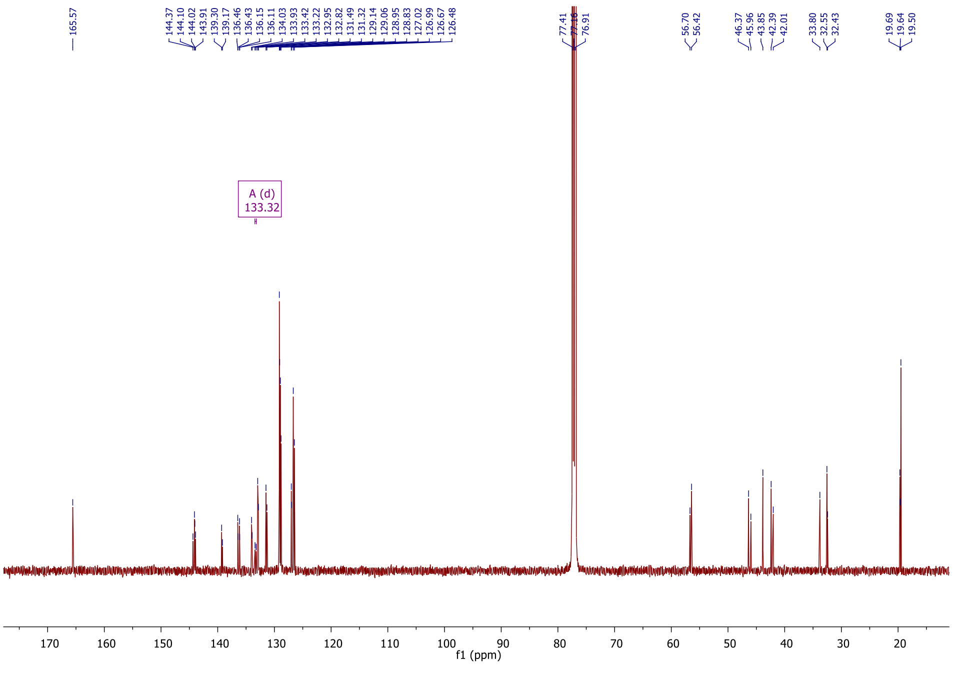

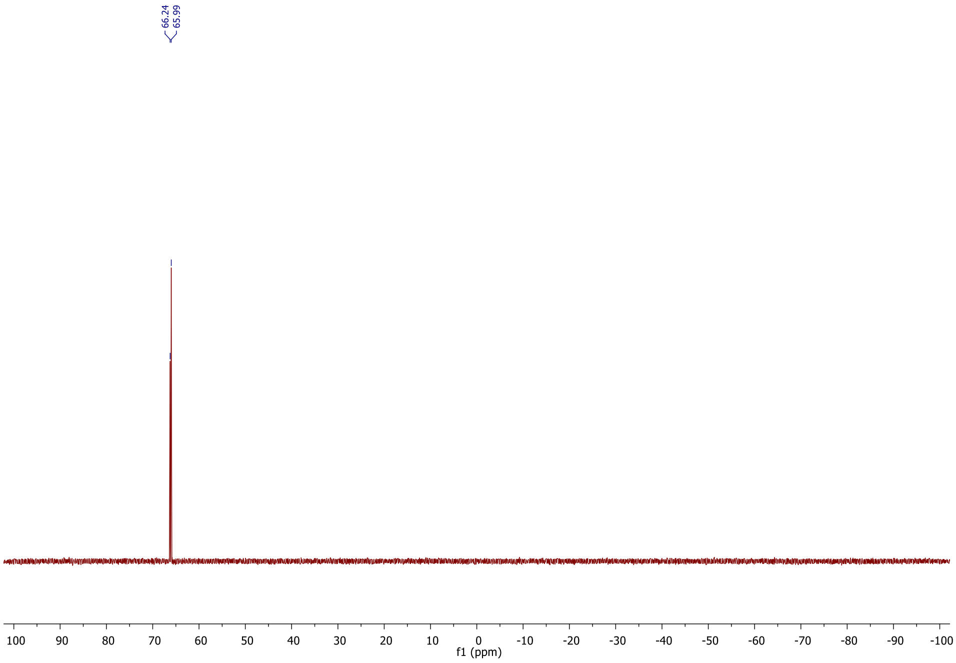
66.24
65.99
100
90
80
70
60
50
40
30
20
10
0
-10
-20
-30
-40
-50
-60
-70
-80
-90
-100
f1 (ppm)

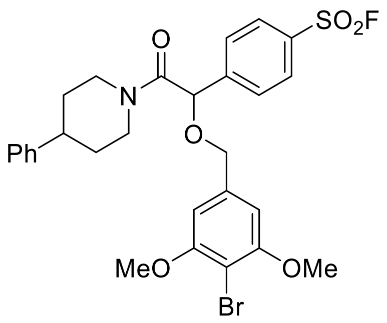

SO2F
O
N
Ph
O
MeO
OMe
Br

2-8

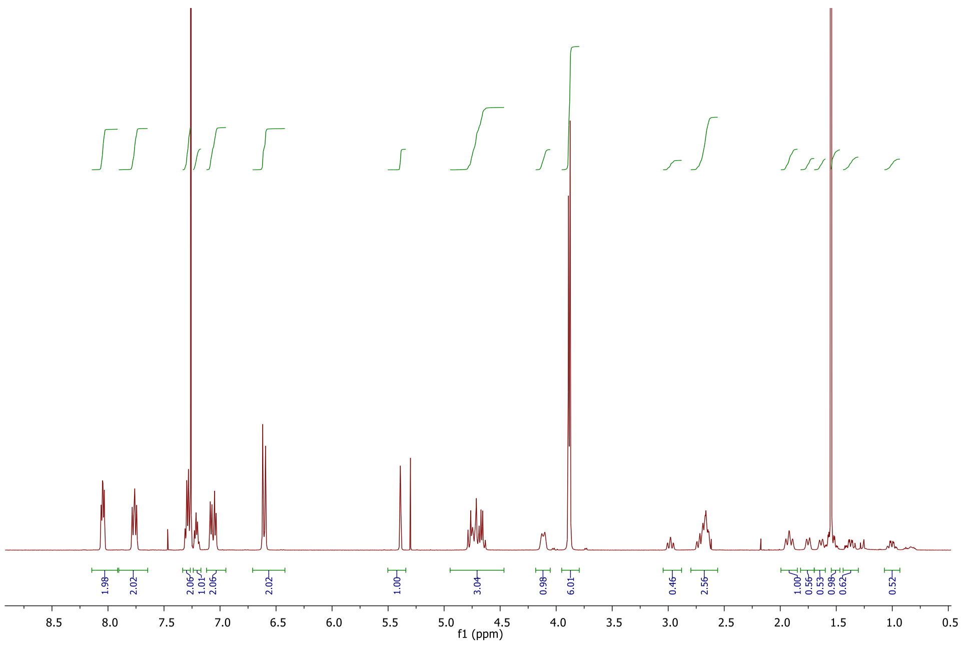

1.98
2.02
2.06
1.01
2.06
2.02
1.00
3.04
0.98
6.01
0.46
2.56
1.00
0.56
0.53
0.98
0.62
0.52
8.5 8.0 7.5 7.0 6.5 6.0 5.5 5.0 4.5 4.0 3.5 3.0 2.5 2.0 1.5 1.0 0.5
f1 (ppm)

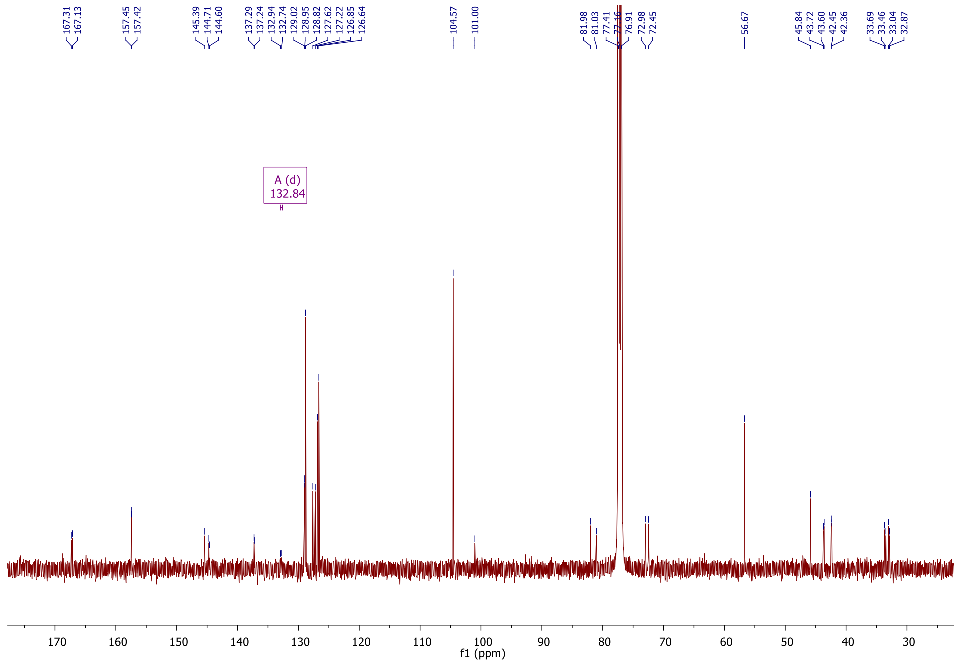

167.31
167.13
157.45
157.42
145.39
144.71
144.60
137.29
137.24
132.94
132.74
129.02
128.95
128.82
127.62
127.22
126.85
126.64
104.57
101.00
81.98
81.03
77.41
77.16
76.91
72.98
72.45
56.67
45.84
43.72
43.60
42.45
42.36
33.69
33.46
33.04
32.87
A (d)
132.84
170 160 150 140 130 120 110 100 90 80 70 60 50 40 30
f1 (ppm)

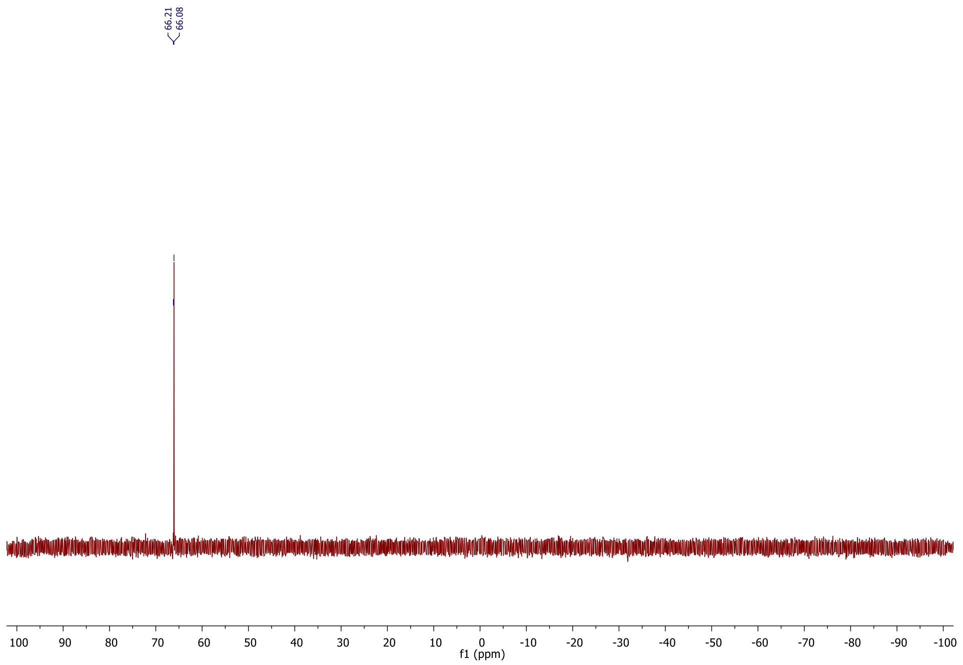
66.21
66.08
100 90 80 70 60 50 40 30 20 10 0 -10 -20 -30 -40 -50 -60 -70 -80 -90 -100
f1 (ppm)

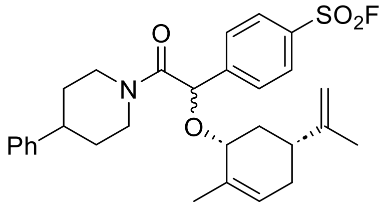

2-14

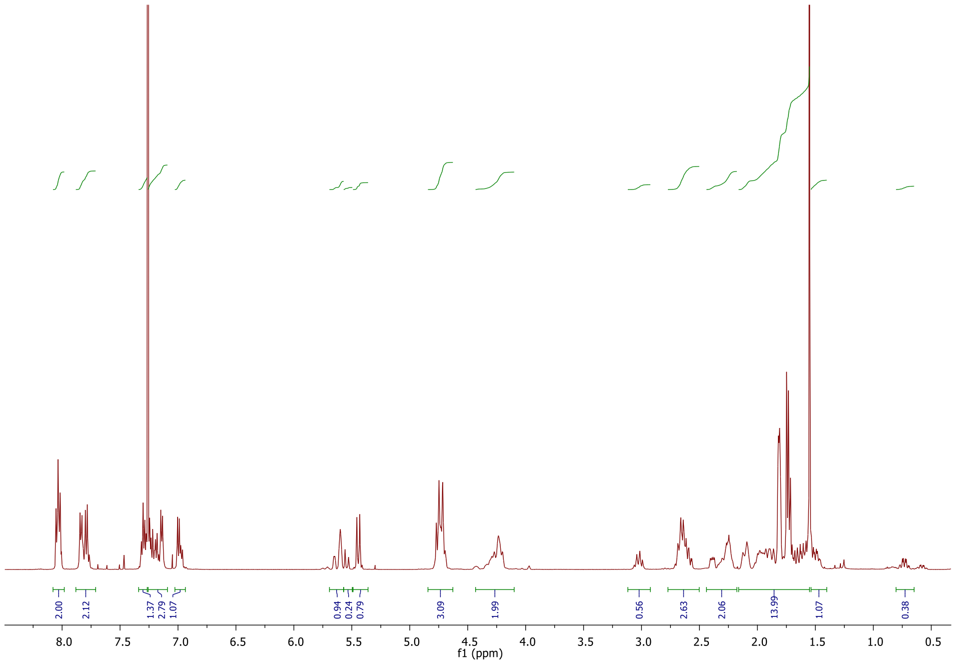

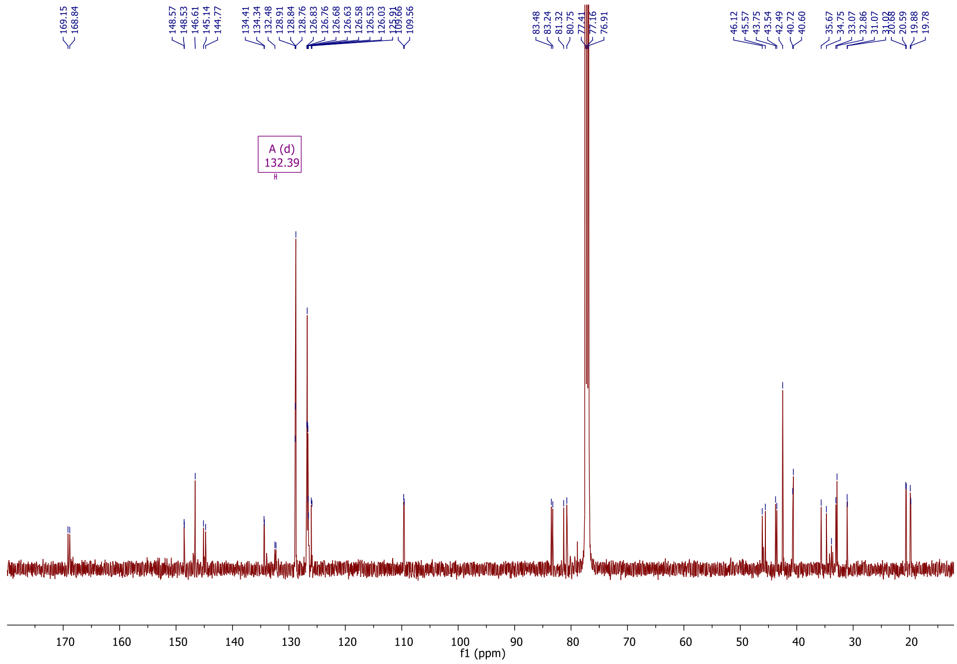

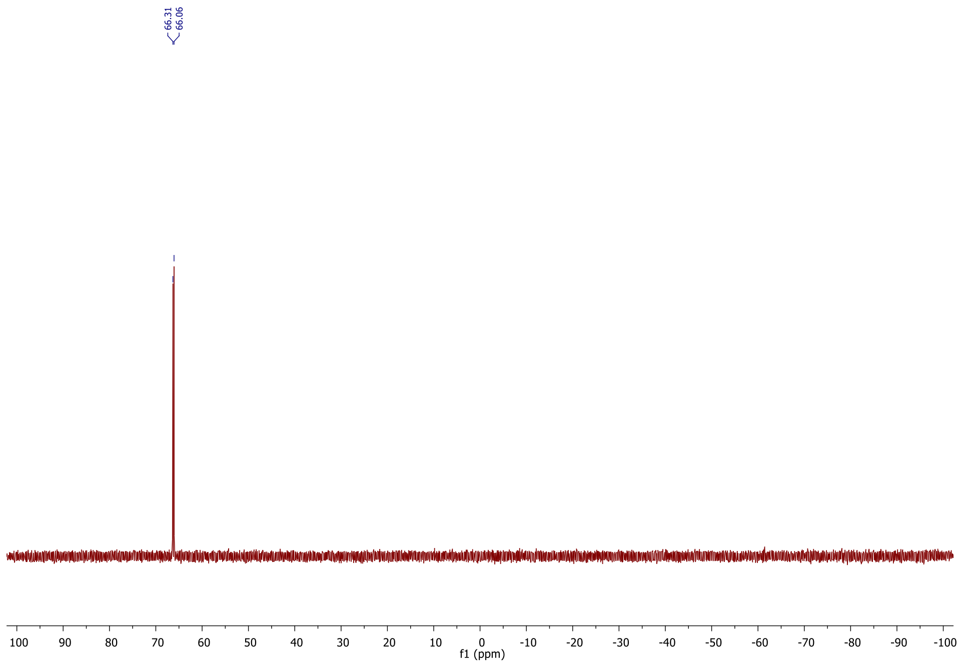
66.31
66.06
100 90 80 70 60 50 40 30 20 10 0 -10 -20 -30 -40 -50 -60 -70 -80 -90 -100
f1 (ppm)

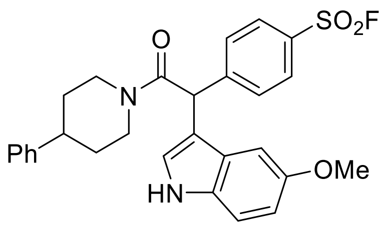

2-15a

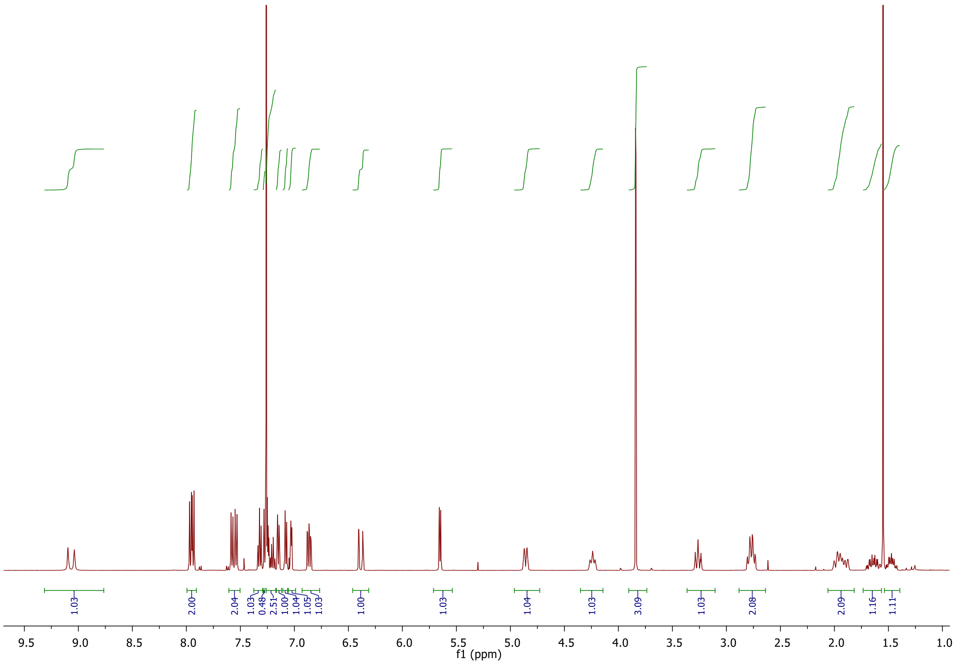

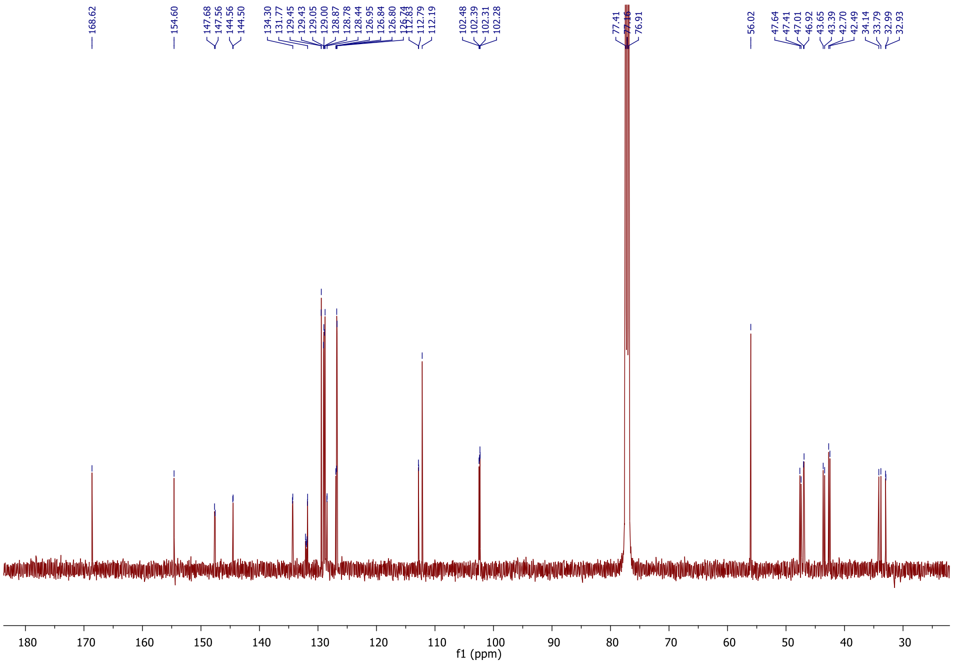

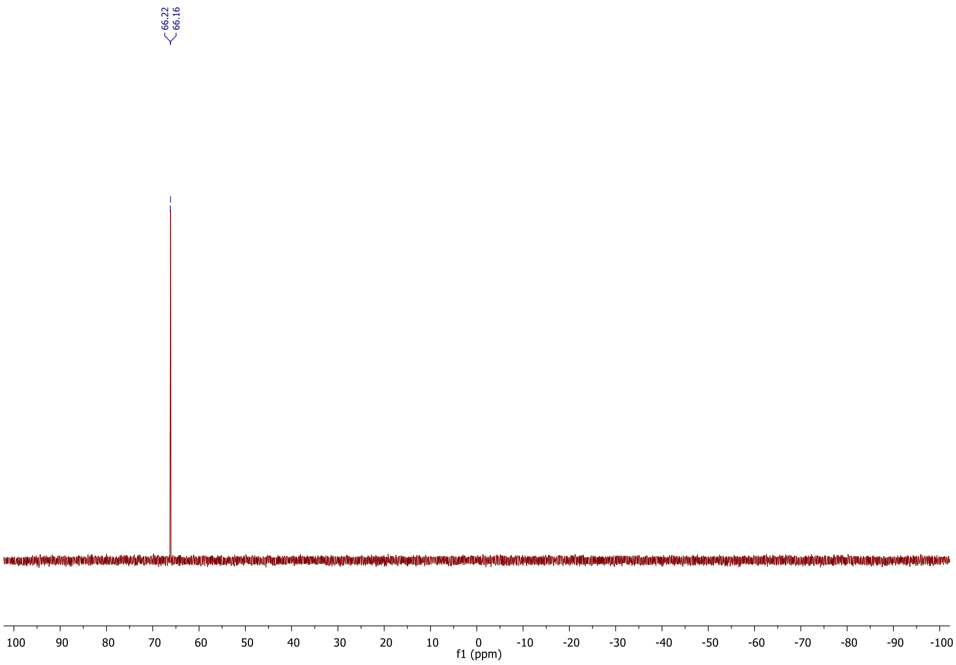
66.22
66.16
100
90
80
70
60
50
40
30
20
10
0
-10
-20
-30
-40
-50
-60
-70
-80
-90
-100
f1 (ppm)

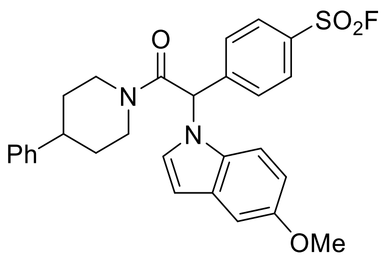
SO2F
O
N
N
Ph
OMe

2-15b

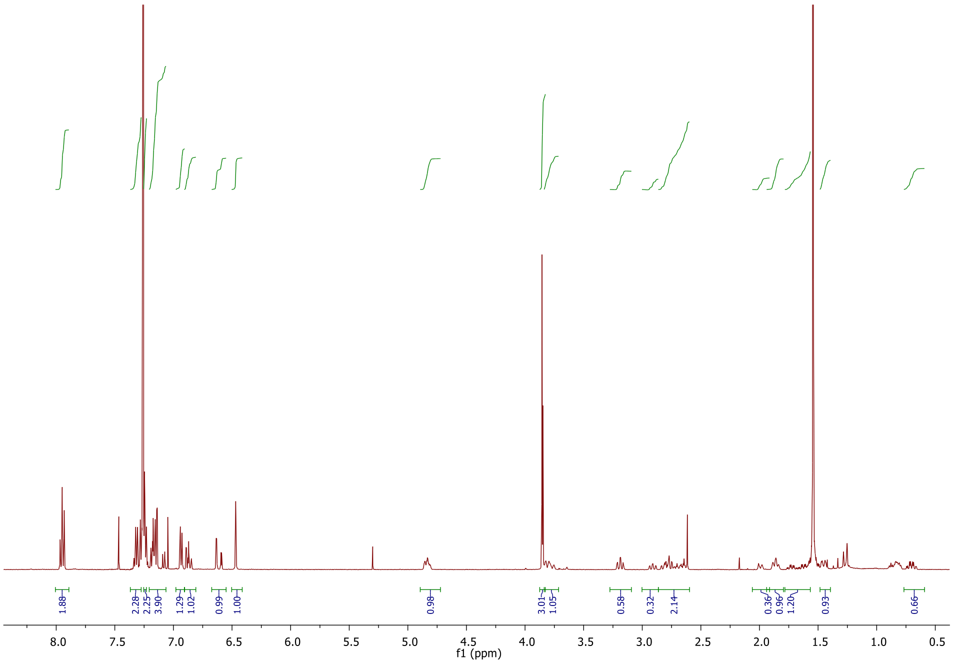
1.88
2.28
2.25
3.90
1.29
1.02
0.99
1.00
0.98
3.01
1.05
0.58
0.32
2.14
0.36
0.96
1.20
0.93
0.66
8.0 7.5 7.0 6.5 6.0 5.5 5.0 4.5 4.0 3.5 3.0 2.5 2.0 1.5 1.0 0.5
f1 (ppm)

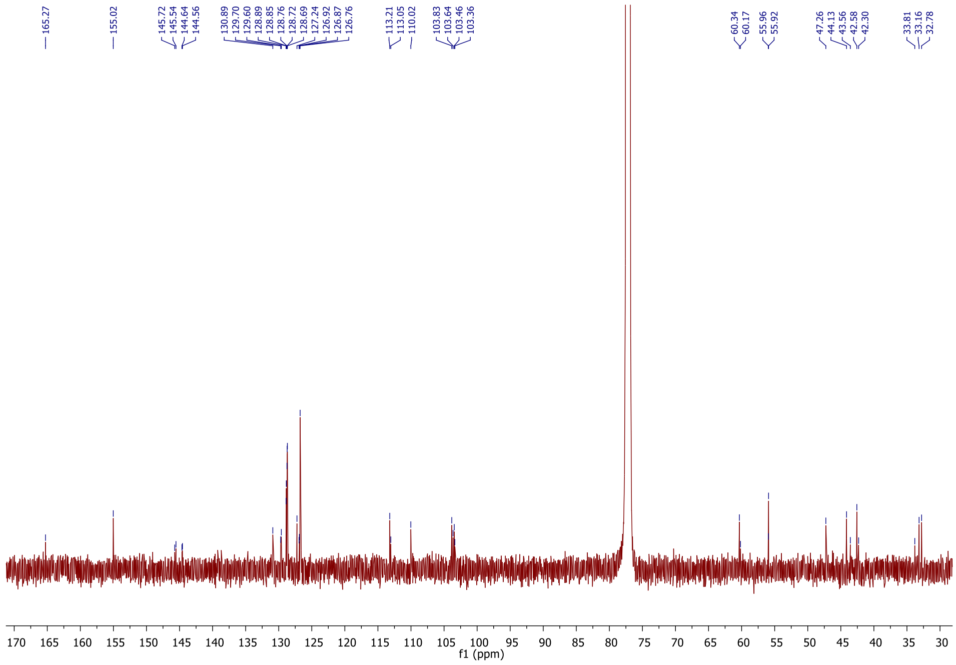
165.27
155.02
145.72
145.54
144.64
144.56
130.89
129.70
129.60
128.89
128.85
128.76
128.72
128.69
127.24
126.92
126.87
126.76
113.21
113.05
110.02
103.83
103.64
103.46
103.36
60.34
60.17
55.96
55.92
47.26
44.13
43.56
42.58
42.30
33.81
33.16
32.78
170 165 160 155 150 145 140 135 130 125 120 115 110 105 100 95 90 85 80 75 70 65 60 55 50 45 40 35 30
f1 (ppm)

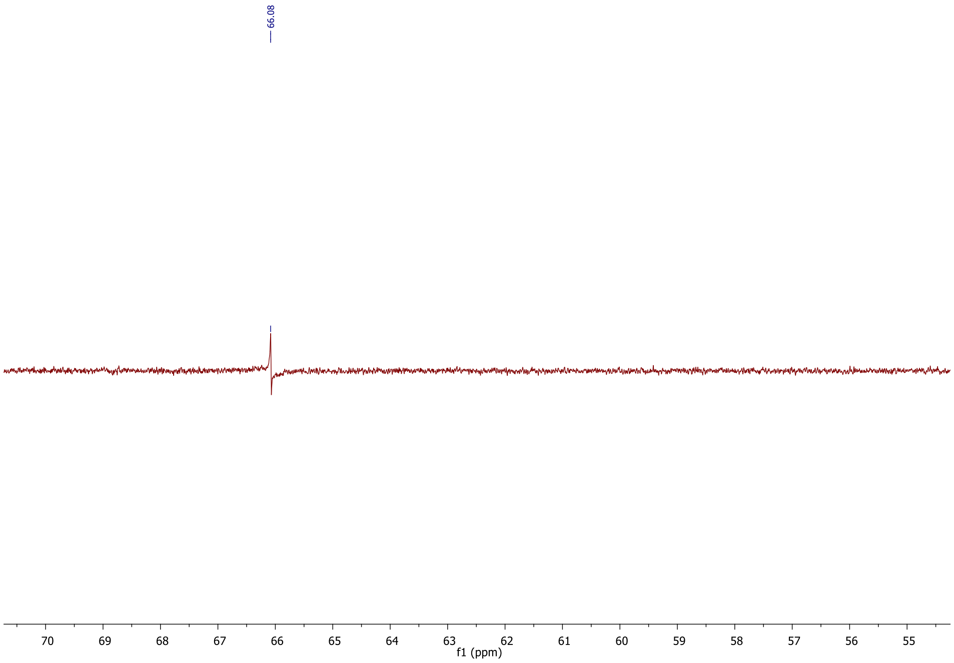
66.08
70 69 68 67 66 65 64 63 62 61 60 59 58 57 56 55
f1 (ppm)

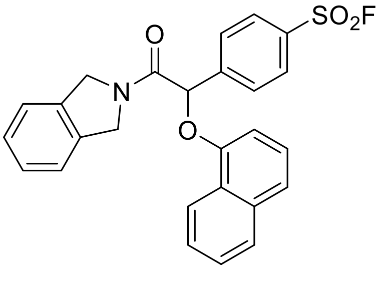

3-4a

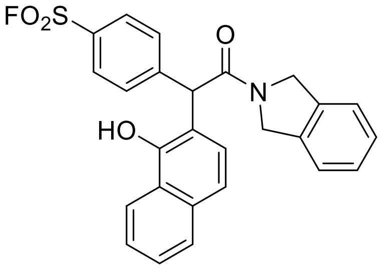

3-4b

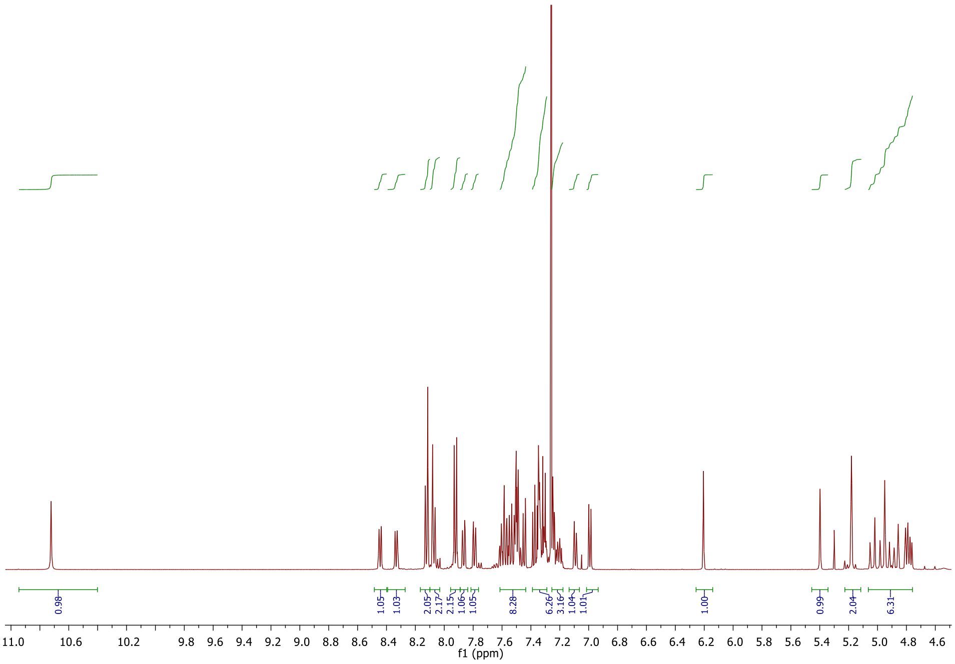

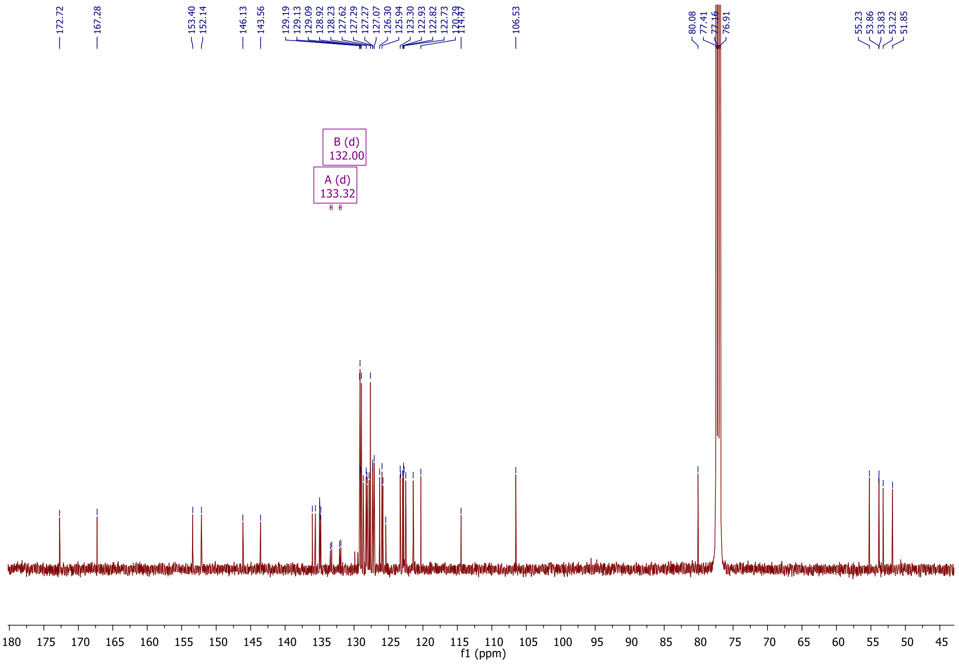

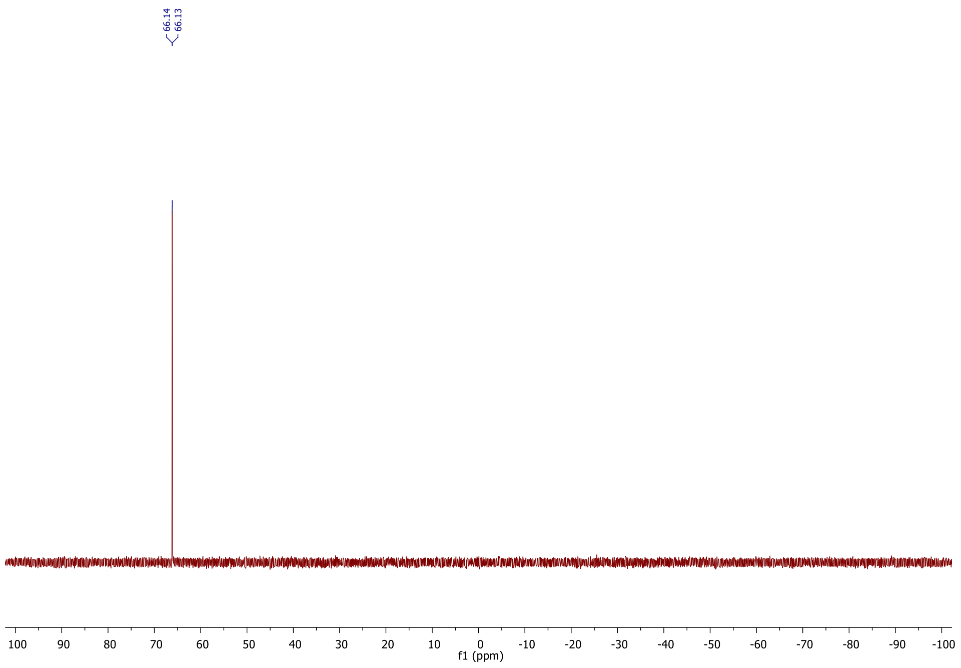
66.14
66.13
100 90 80 70 60 50 40 30 20 10 0 -10 -20 -30 -40 -50 -60 -70 -80 -90 -100
f1 (ppm)

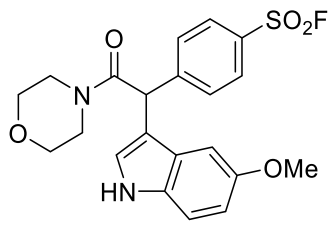

1-15a

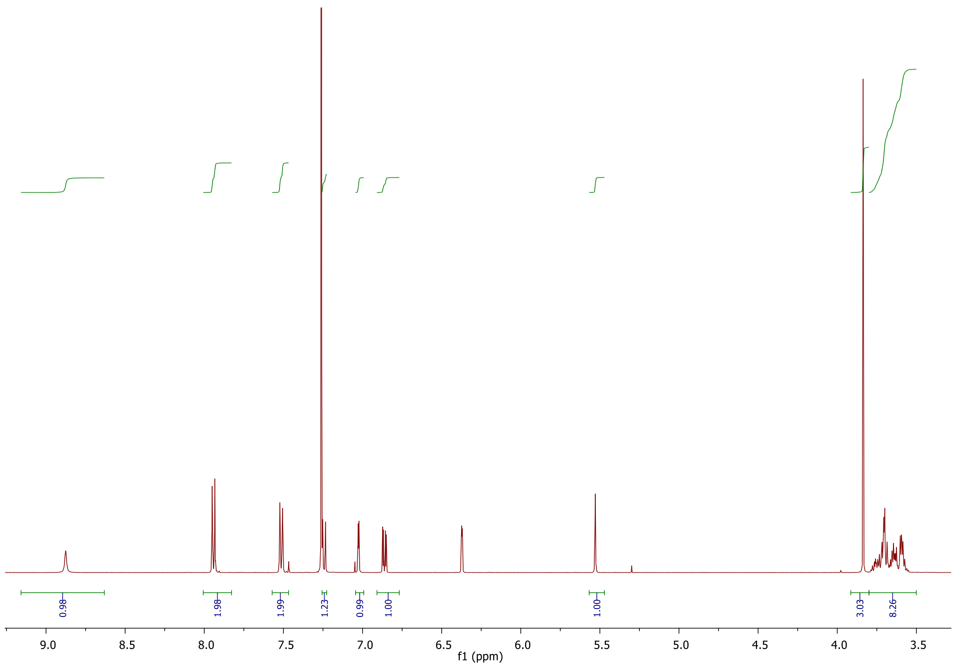

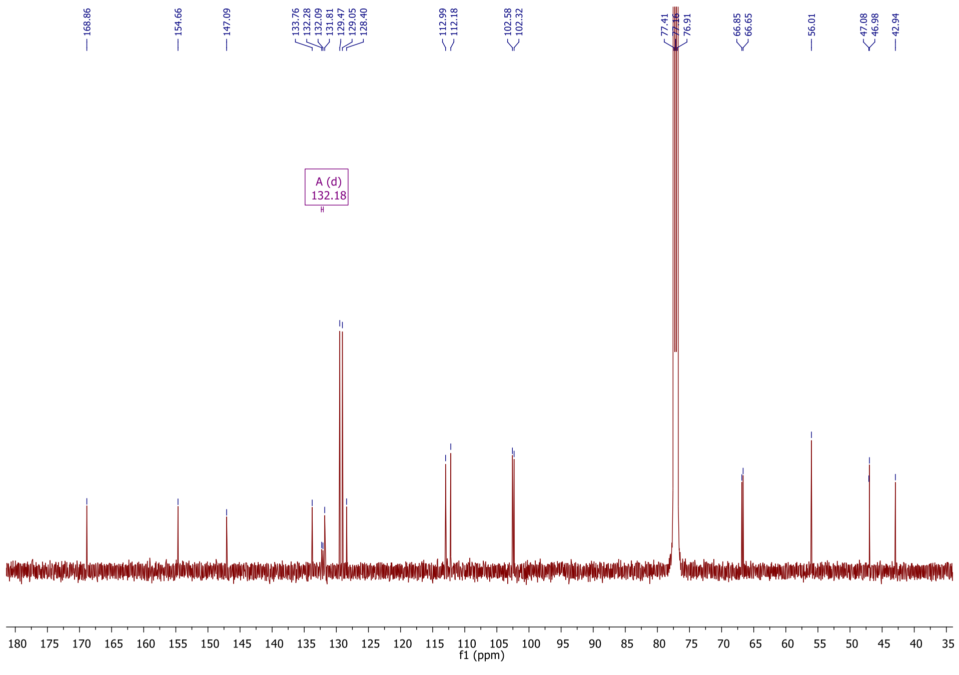

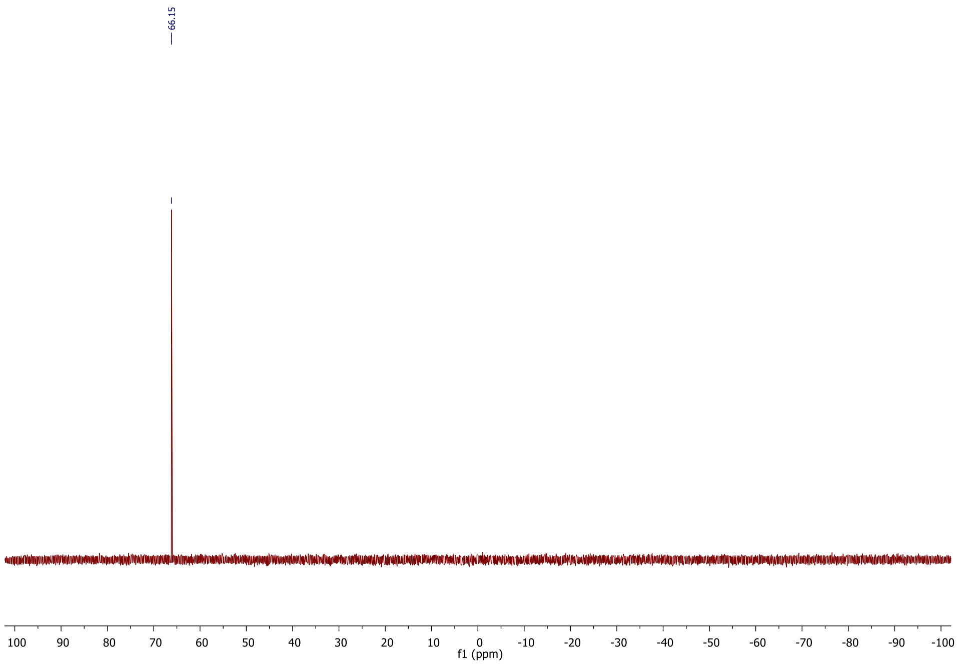
66.15
100
90
80
70
60
50
40
30
20
10
0
-10
-20
-30
-40
-50
-60
-70
-80
-90
-100
f1 (ppm)

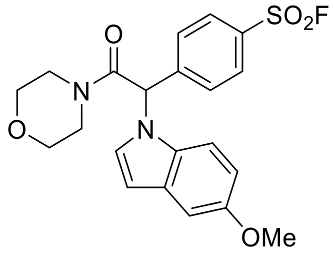

1-15b

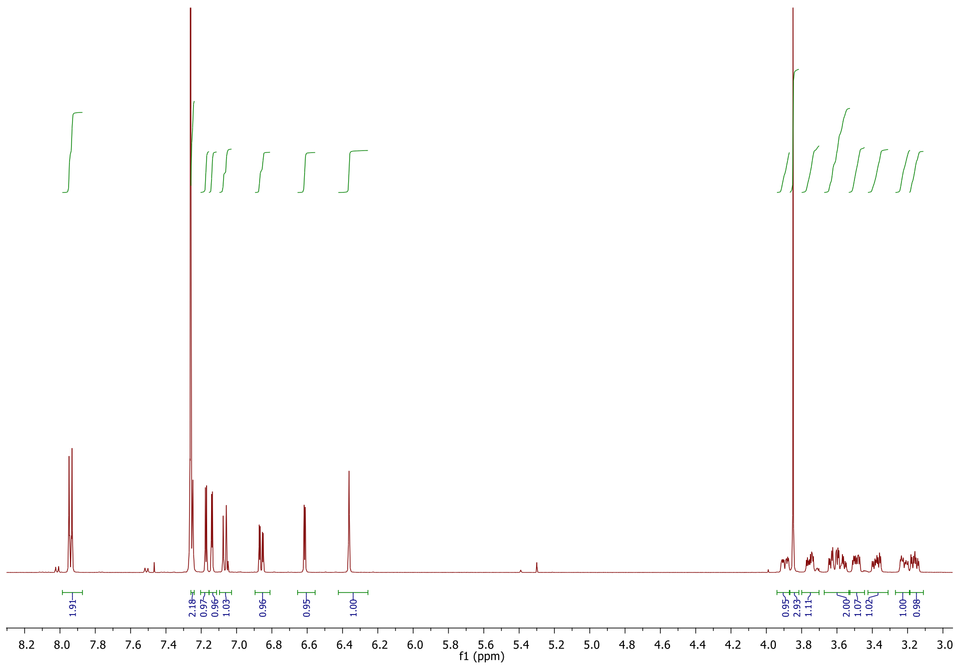

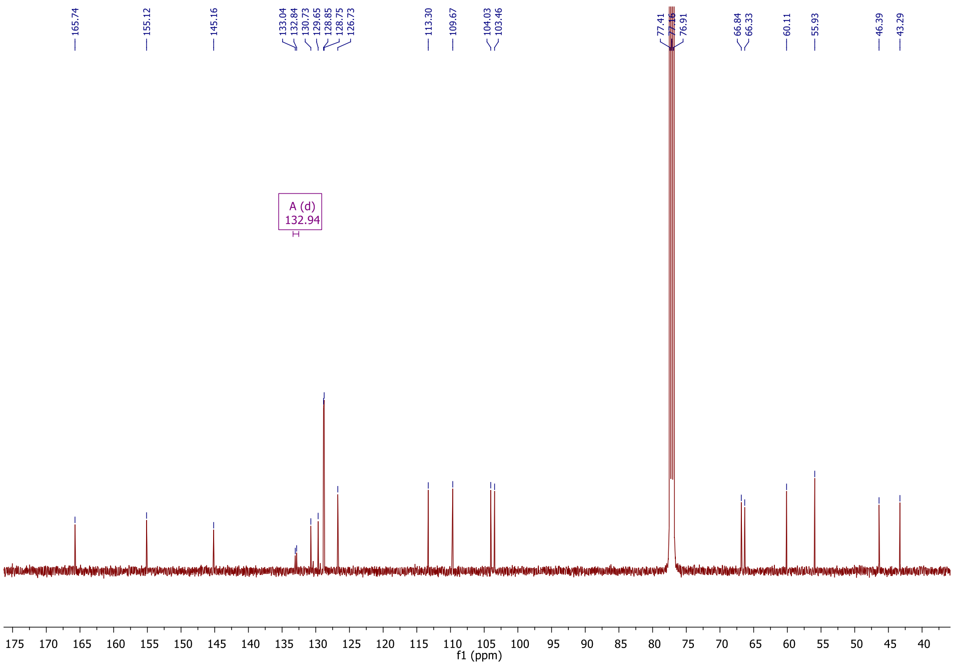

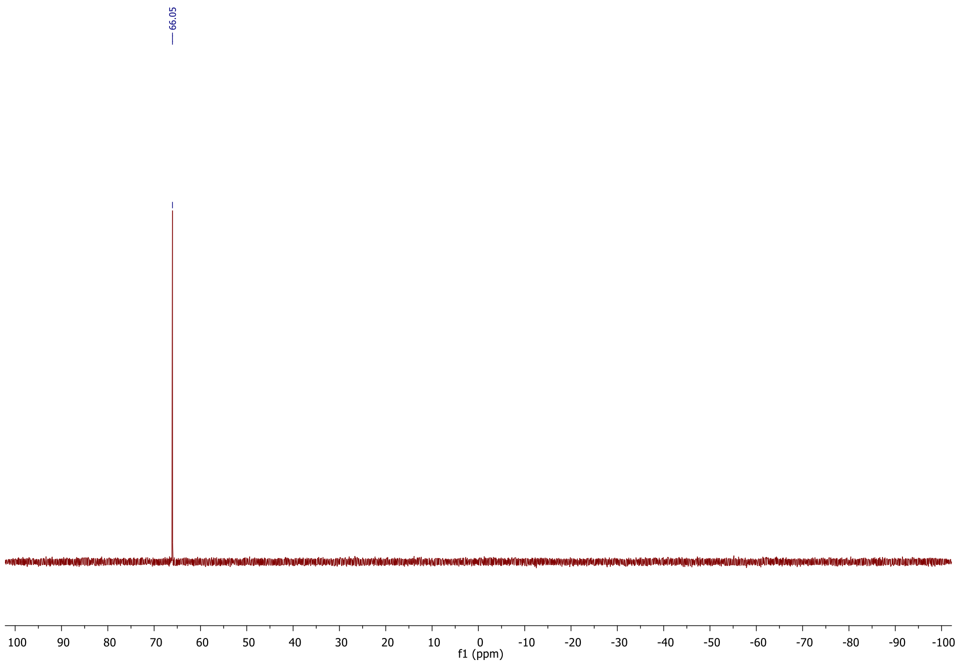
66.05
100 90 80 70 60 50 40 30 20 10 0 -10 -20 -30 -40 -50 -60 -70 -80 -90 -100
f1 (ppm)

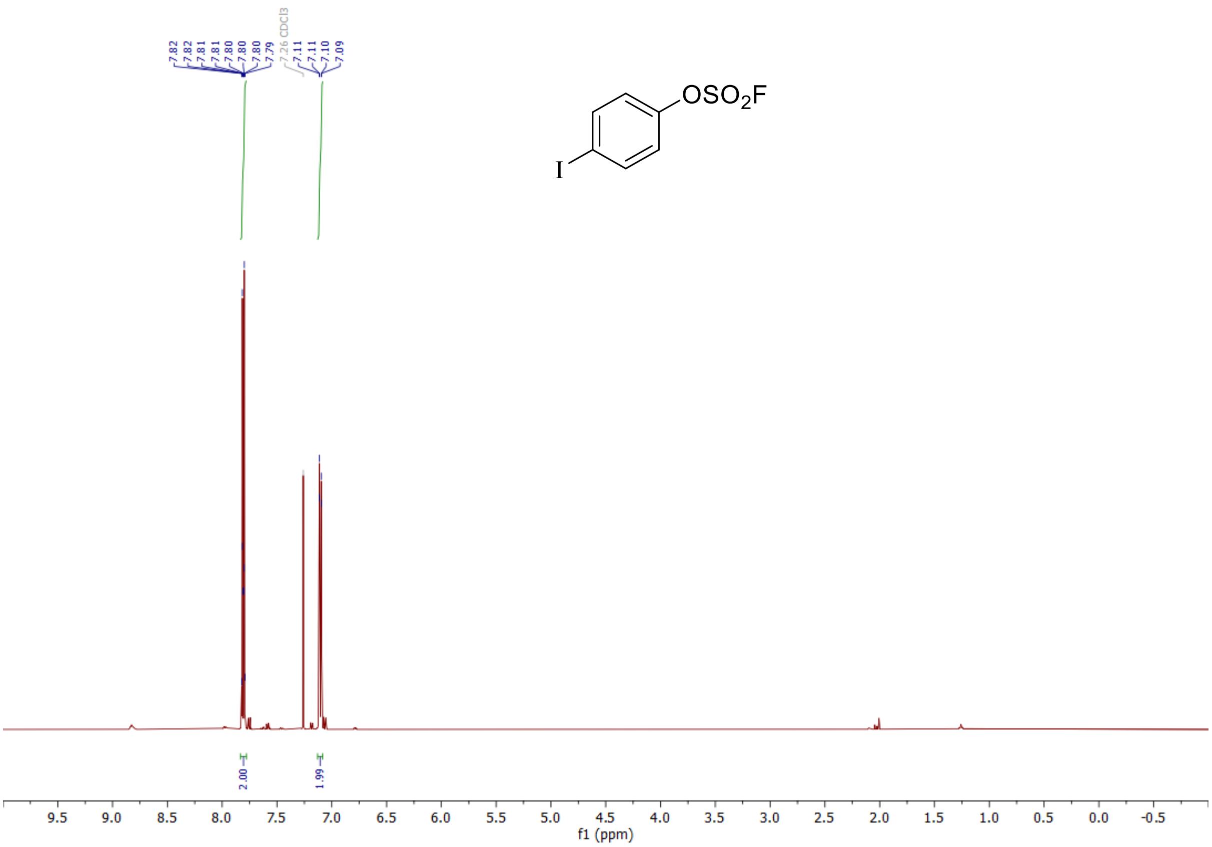
OSO2F
I
7.82
7.82
7.81
7.81
7.80
7.80
7.80
7.79
7.26 CDCl3
7.11
7.11
7.10
7.09
2.00
1.99
9.5 9.0 8.5 8.0 7.5 7.0 6.5 6.0 5.5 5.0 4.5 4.0 3.5 3.0 2.5 2.0 1.5 1.0 0.5 0.0 -0.5
f1 (ppm)

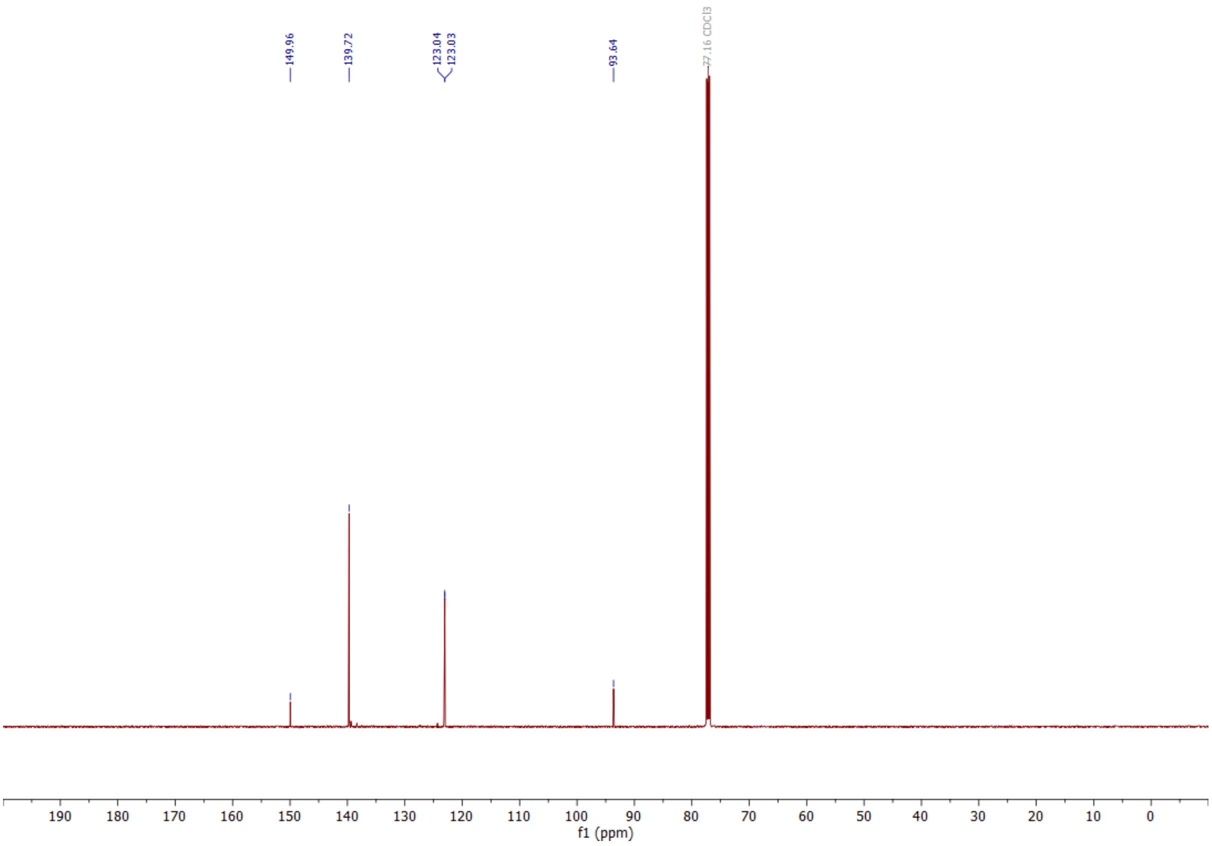
149.96
139.72
123.04
123.03
93.64
77.16 CDCl3
190 180 170 160 150 140 130 120 110 100 90 80 70 60 50 40 30 20 10 0
f1 (ppm)

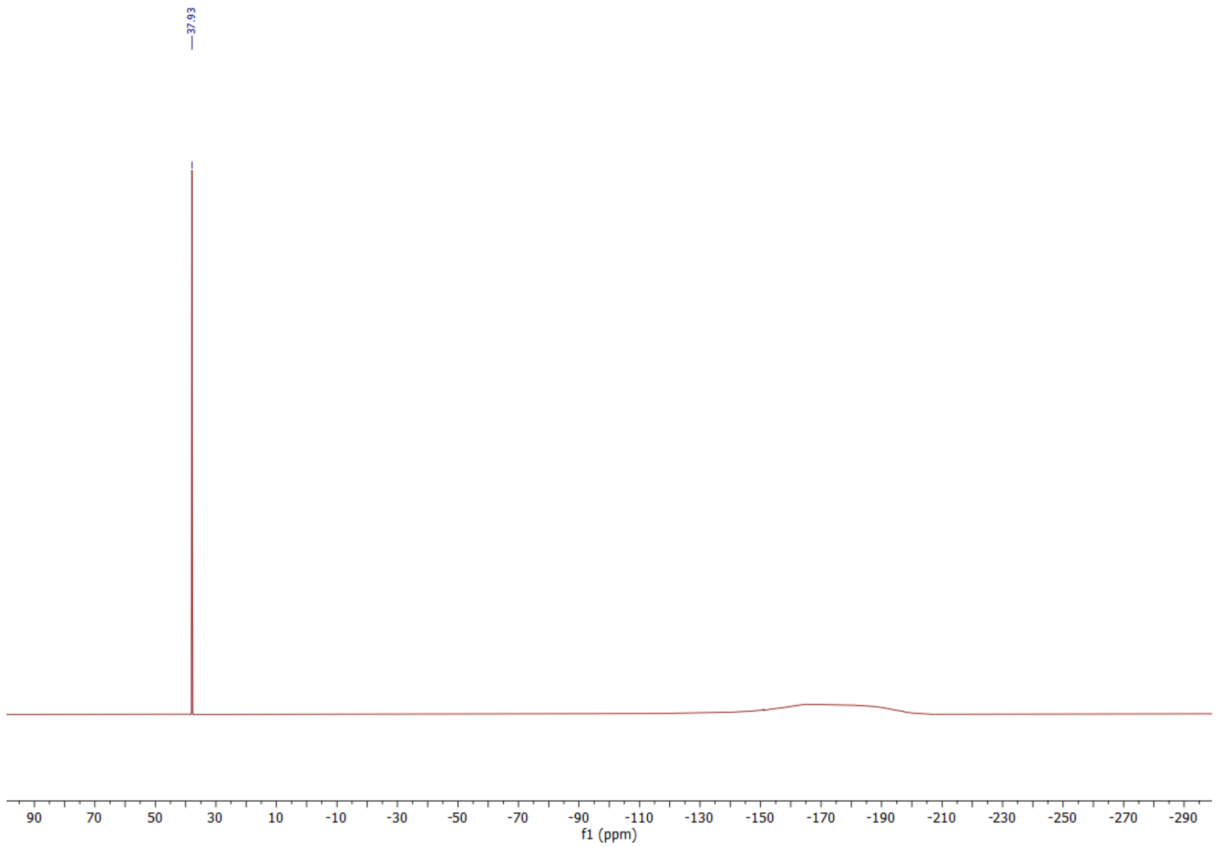

37.93
90
70
50
30
10
-10
-30
-50
-70
-90
-110
-130
-150
-170
-190
-210
-230
-250
-270
-290
f1 (ppm)

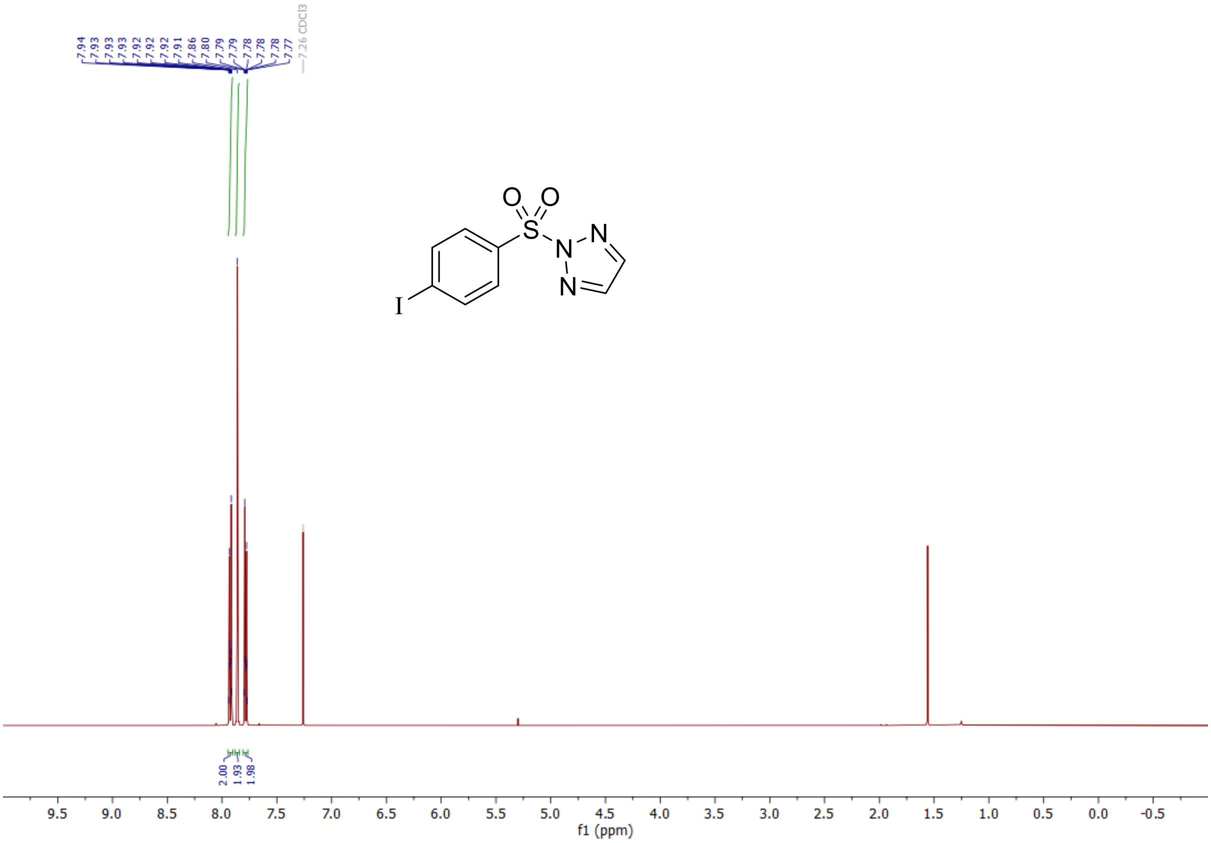
7.94
7.93
7.93
7.93
7.92
7.92
7.92
7.91
7.86
7.80
7.79
7.79
7.78
7.78
7.78
7.77
7.26 CDCl3
O
O
S
N
N
N
I
2.00
1.93
1.98
9.5 9.0 8.5 8.0 7.5 7.0 6.5 6.0 5.5 5.0 4.5 4.0 3.5 3.0 2.5 2.0 1.5 1.0 0.5 0.0 -0.5
f1 (ppm)

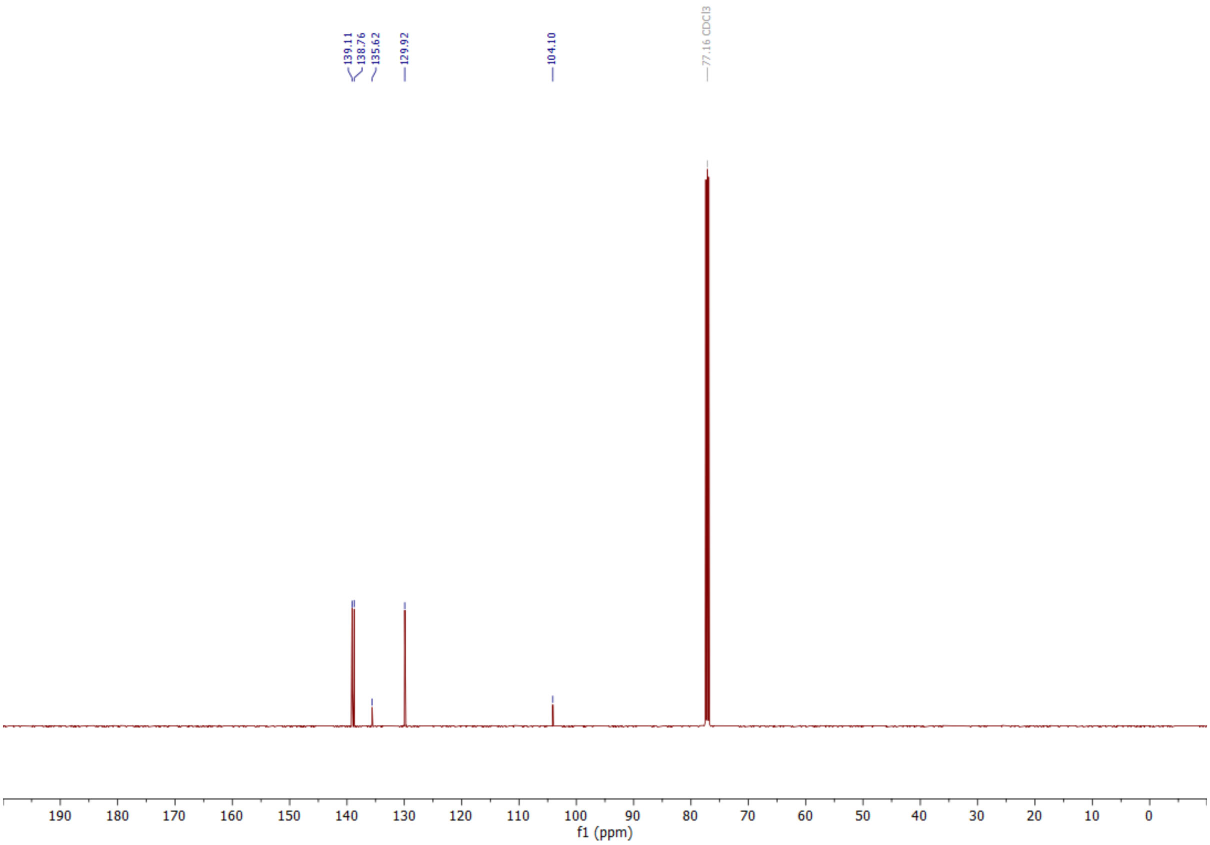
139.11
138.76
135.62
129.92
104.10
77.16 CDCl3
190 180 170 160 150 140 130 120 110 100 90 80 70 60 50 40 30 20 10 0
f1 (ppm)

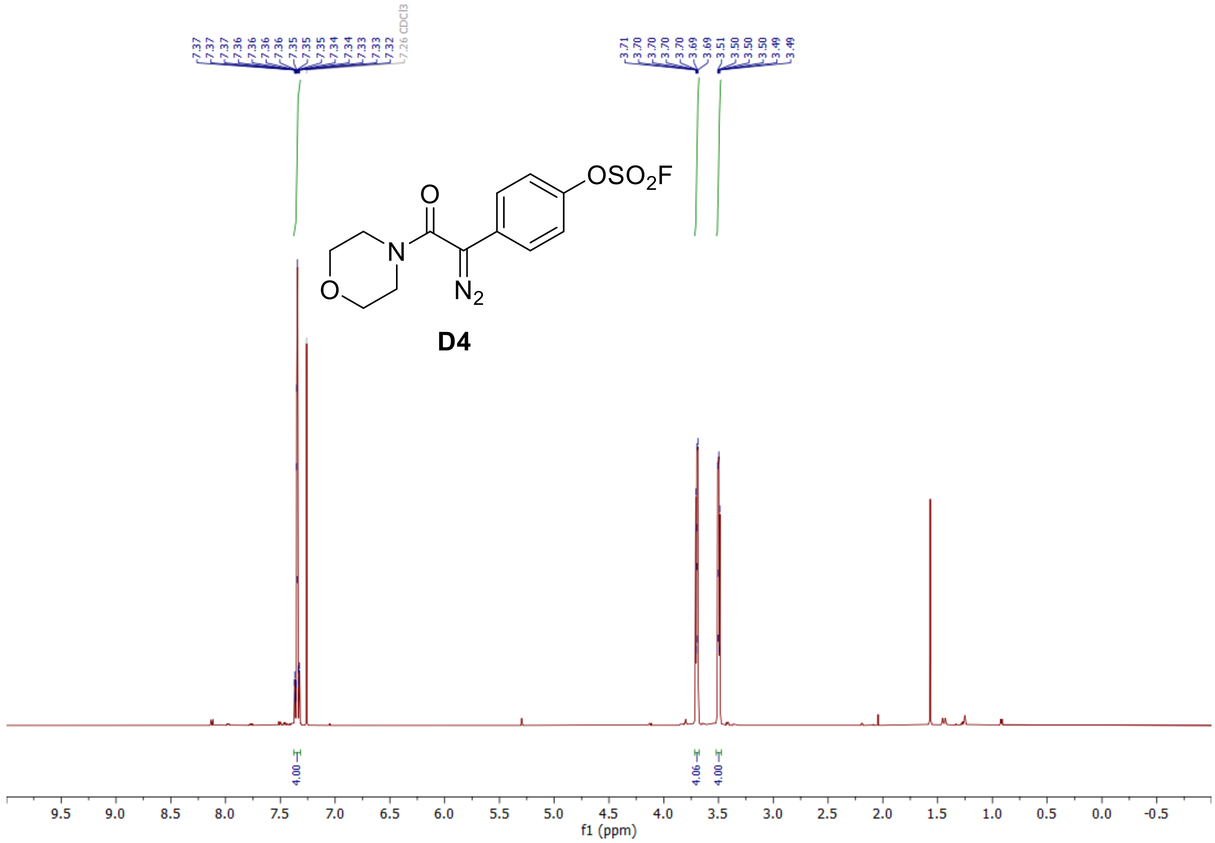
7.37
7.37
7.37
7.36
7.36
7.36
7.36
7.35
7.35
7.35
7.34
7.34
7.33
7.33
7.32
7.26 CDCl3
3.71
3.70
3.70
3.70
3.70
3.69
3.69
3.51
3.50
3.50
3.50
3.49
3.49
OSO2F
O
N
O
N2
D4
4.00
4.06
4.00
f1 (ppm)

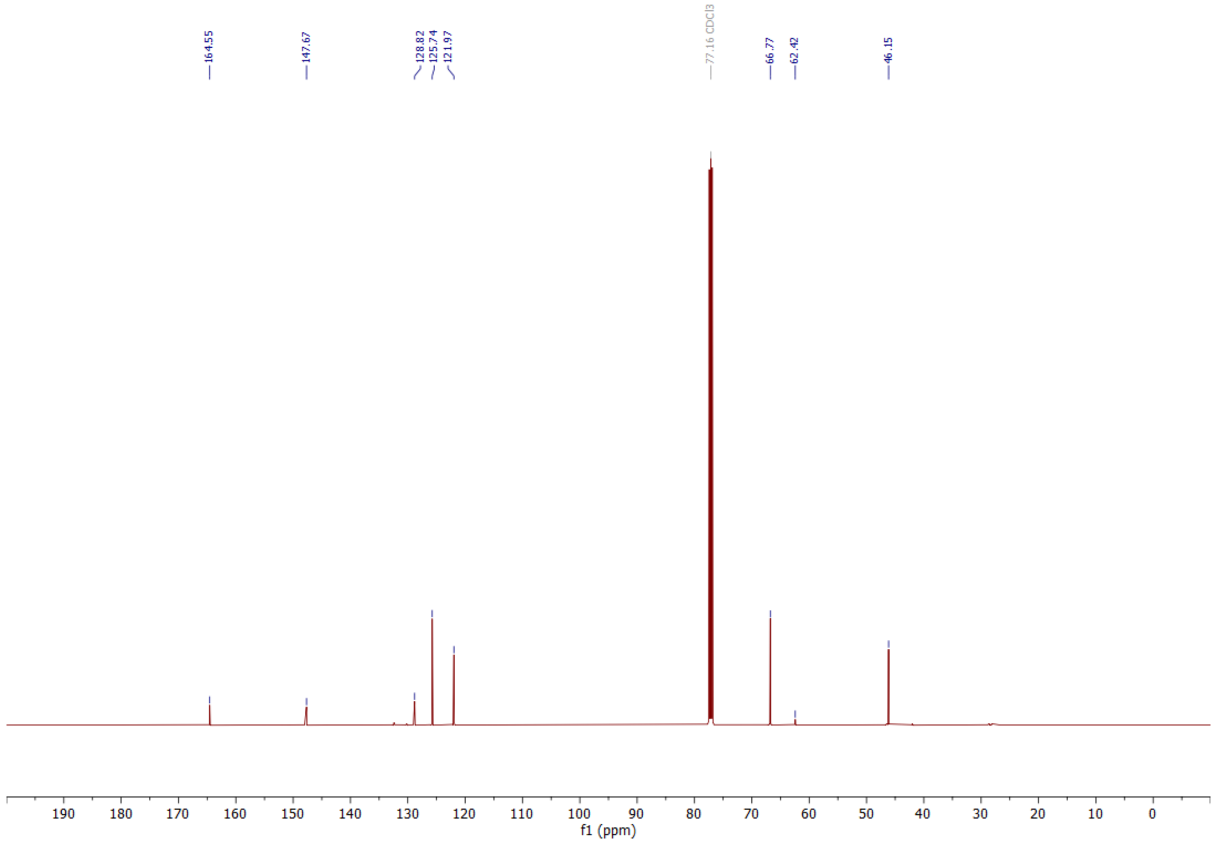
164.55
147.67
128.82
125.74
121.97
77.16 CDCl3
66.77
62.42
46.15
f1 (ppm)

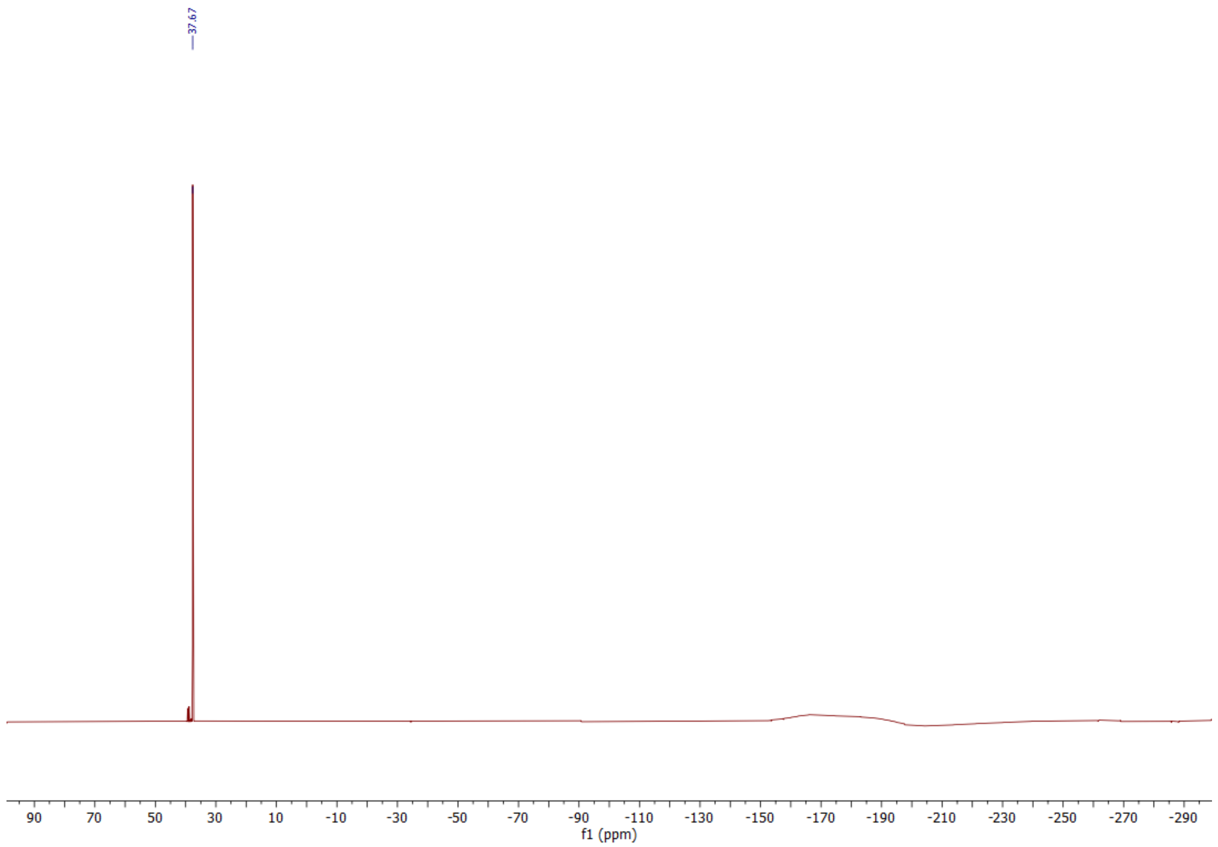

37.67
90
70
50
30
10
-10
-30
-50
-70
-90
-110
-130
-150
-170
-190
-210
-230
-250
-270
-290
f1 (ppm)

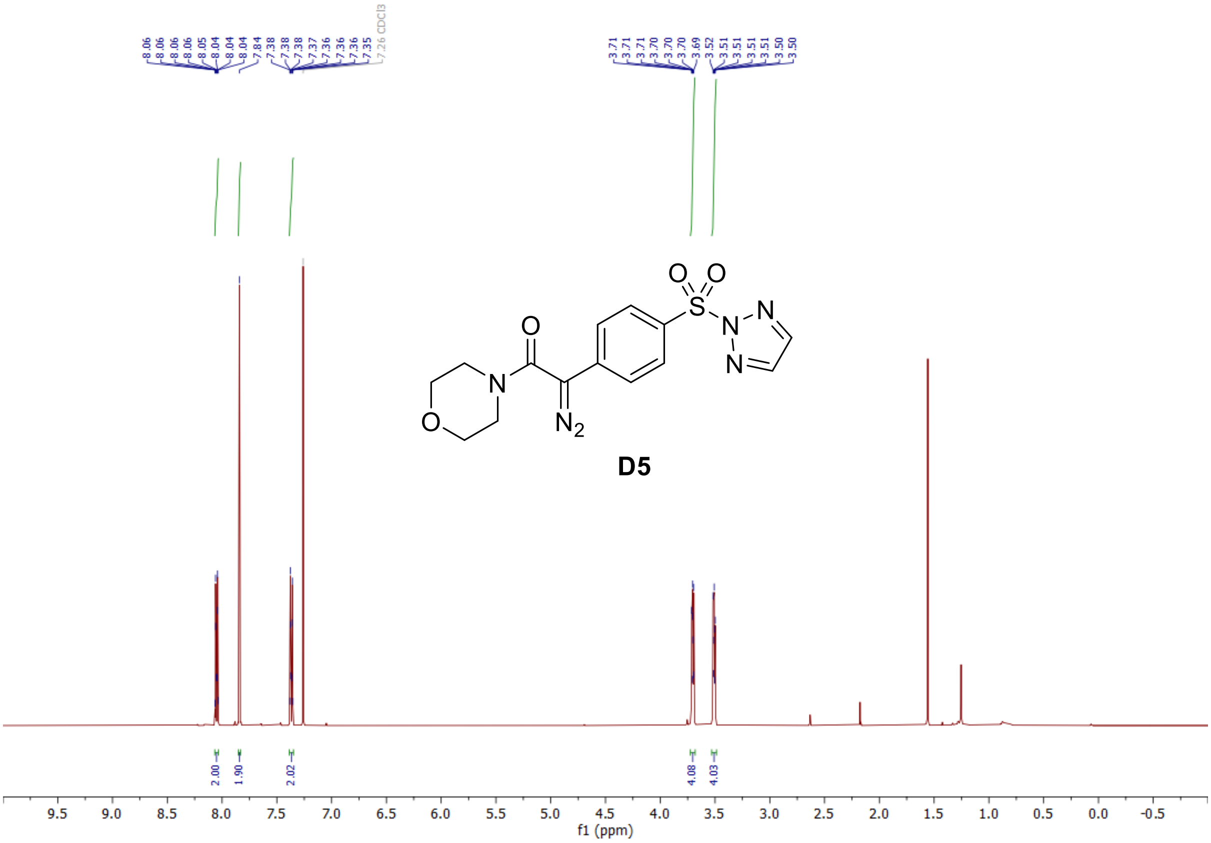
8.06 8.06 8.06 8.06 8.05 8.04 8.04 8.04 7.84 7.38 7.38 7.38 7.37 7.36 7.36 7.36 7.35 7.26 CDCl3
3.71 3.71 3.71 3.70 3.70 3.70 3.69 3.52 3.51 3.51 3.51 3.51 3.50 3.50
D5
2.00 1.90 2.02 4.08 4.03
9.5 9.0 8.5 8.0 7.5 7.0 6.5 6.0 5.5 5.0 4.5 4.0 3.5 3.0 2.5 2.0 1.5 1.0 0.5 0.0 -0.5
f1 (ppm)

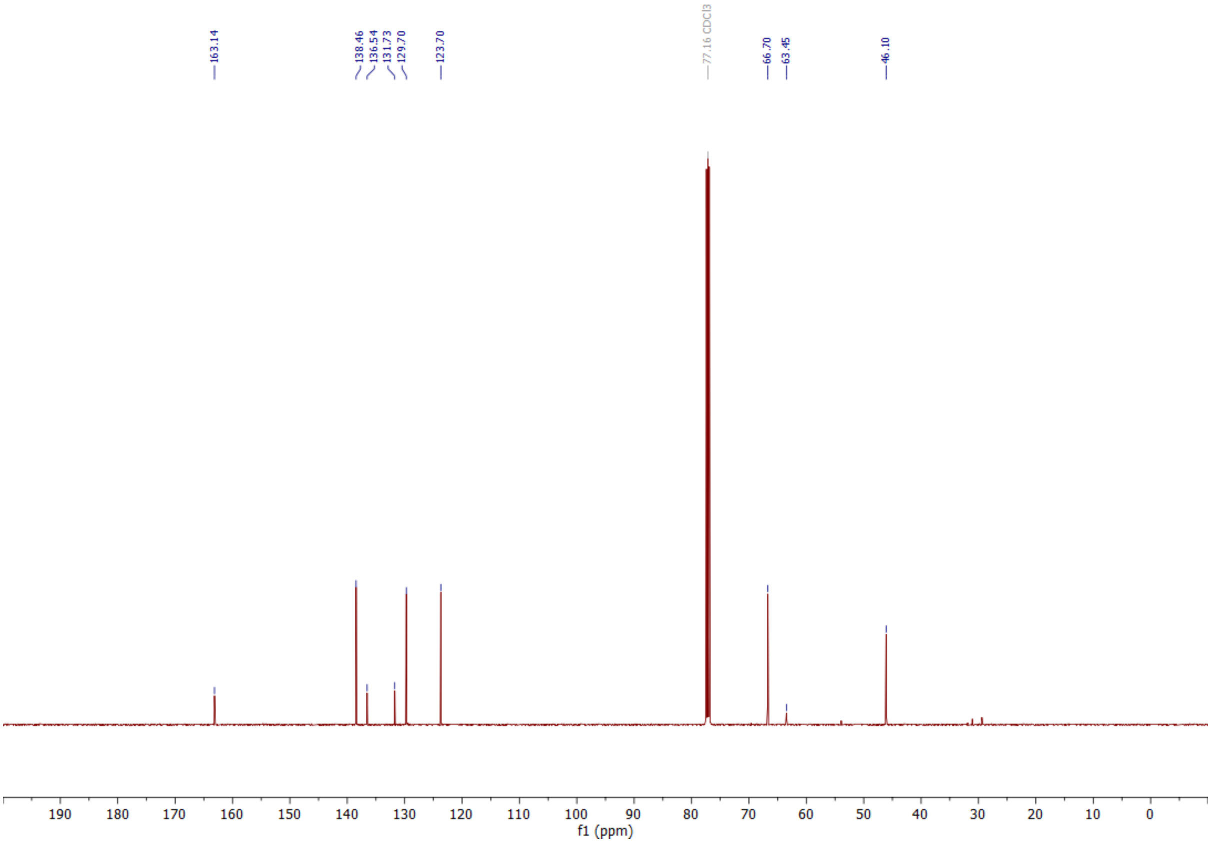
163.14
138.46 136.54 131.73 129.70
123.70
77.16 CDCl3
66.70 63.45
46.10
190 180 170 160 150 140 130 120 110 100 90 80 70 60 50 40 30 20 10 0
f1 (ppm)

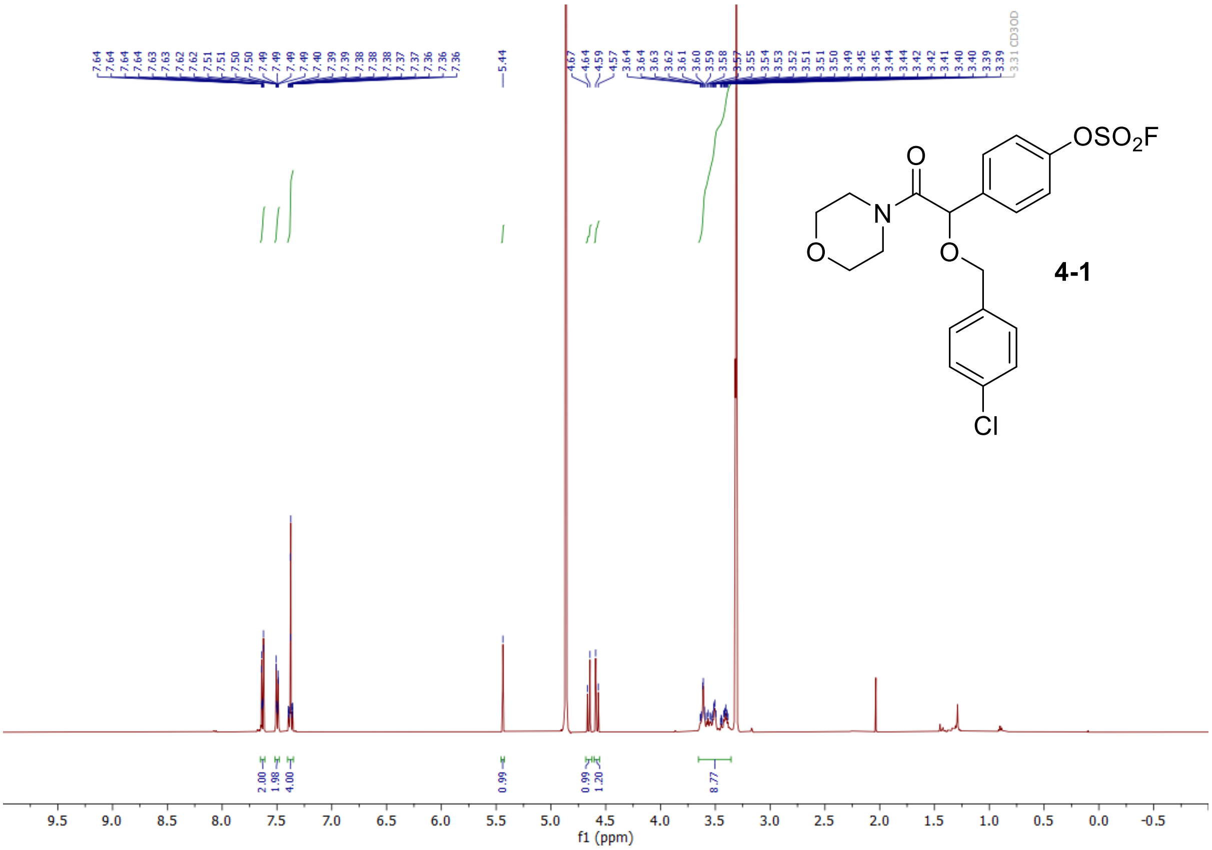
7.64 7.64 7.64 7.63 7.63 7.62 7.62 7.51 7.51 7.50 7.49 7.49 7.49 7.49 7.40 7.39 7.39 7.38 7.38 7.38 7.37 7.37 7.36 7.36
5.44
4.67 4.64 4.59 4.57
3.64 3.64 3.63 3.62 3.61 3.60 3.59 3.58 3.57 3.55 3.54 3.53 3.52 3.51 3.51 3.50 3.49 3.45 3.45 3.44 3.44 3.42 3.42 3.41 3.40 3.39 3.39
3.31 CD3OD
OSO2F
O
N
O
O
Cl
4-1
2.00 1.98 4.00
0.99
0.99 1.20
8.77
f1 (ppm)

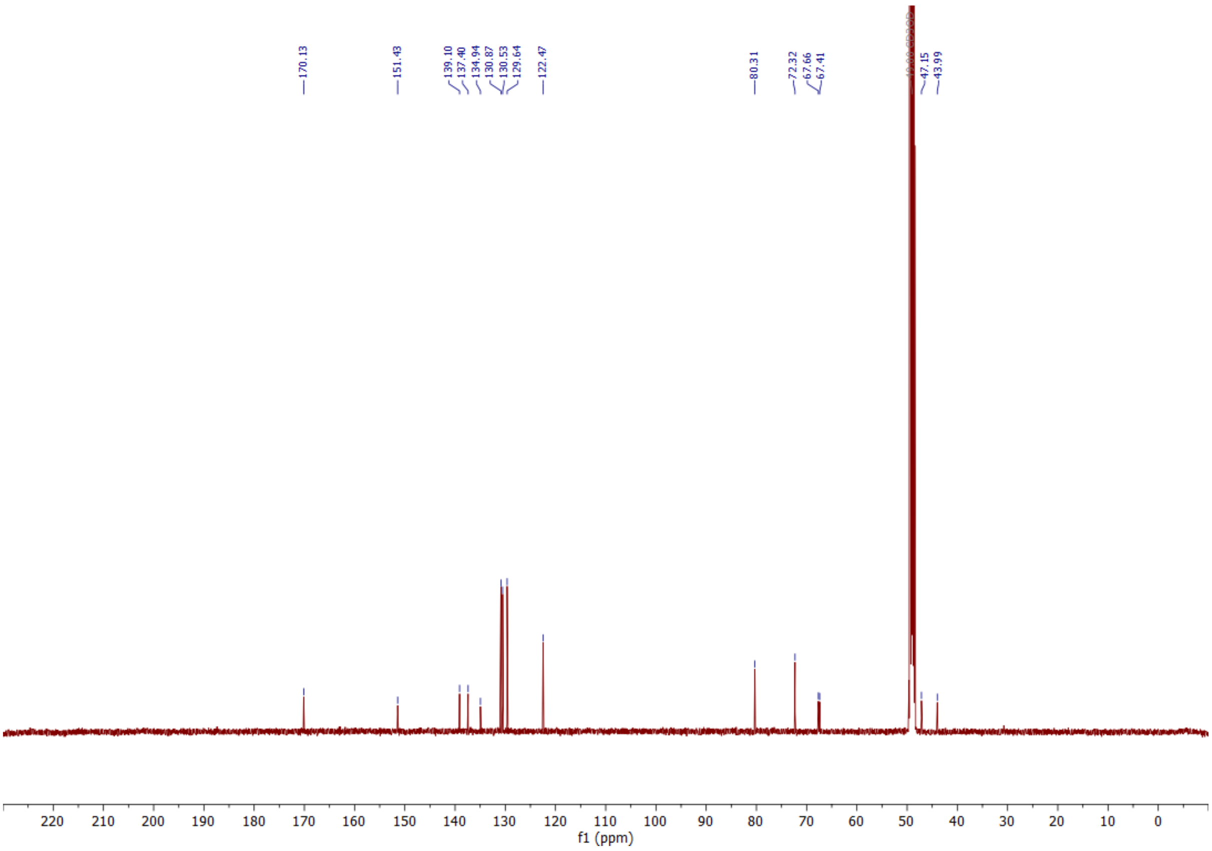
170.13
151.43
139.10 137.40 134.94 130.87 130.53 129.64
122.47
80.31
72.32 67.66 67.41
47.15 43.99
f1 (ppm)

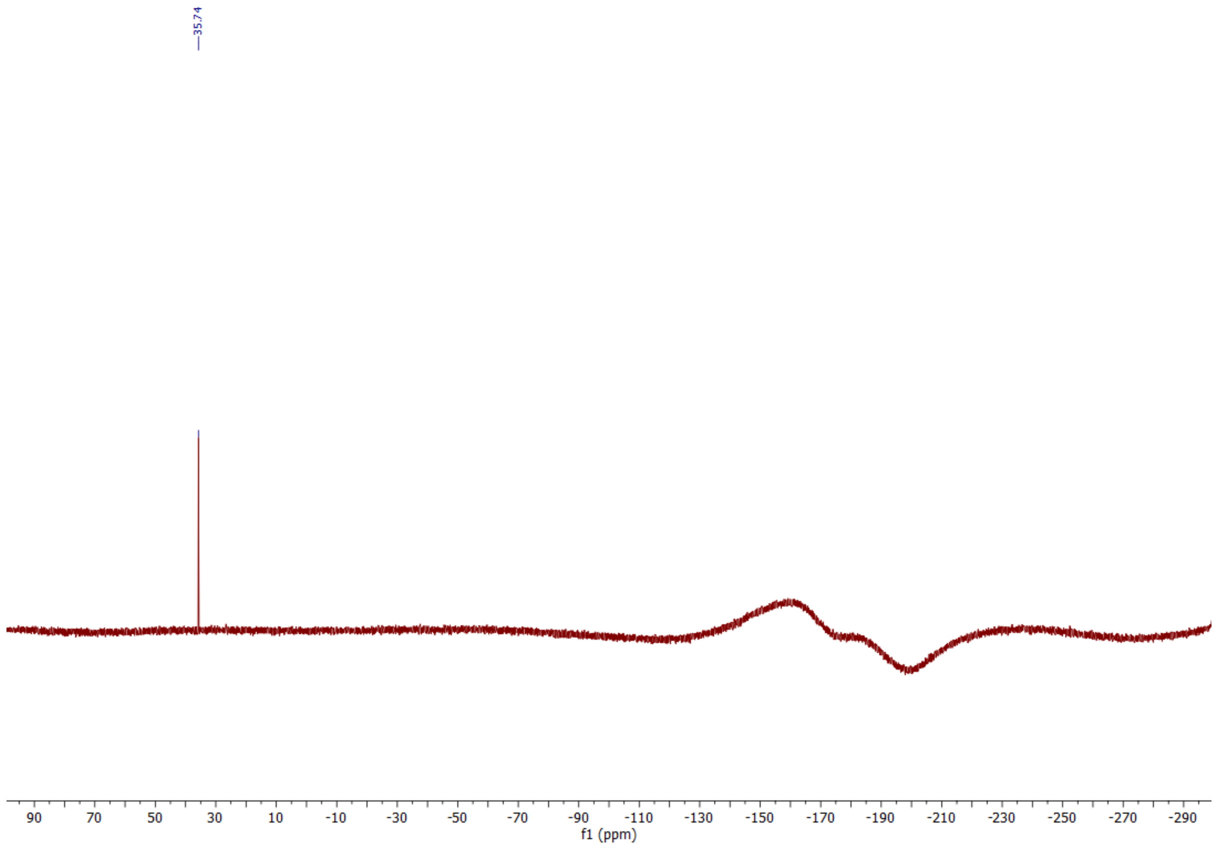

35.74
90
70
50
30
10
-10
-30
-50
-70
-90
-110
-130
-150
-170
-190
-210
-230
-250
-270
-290
f1 (ppm)

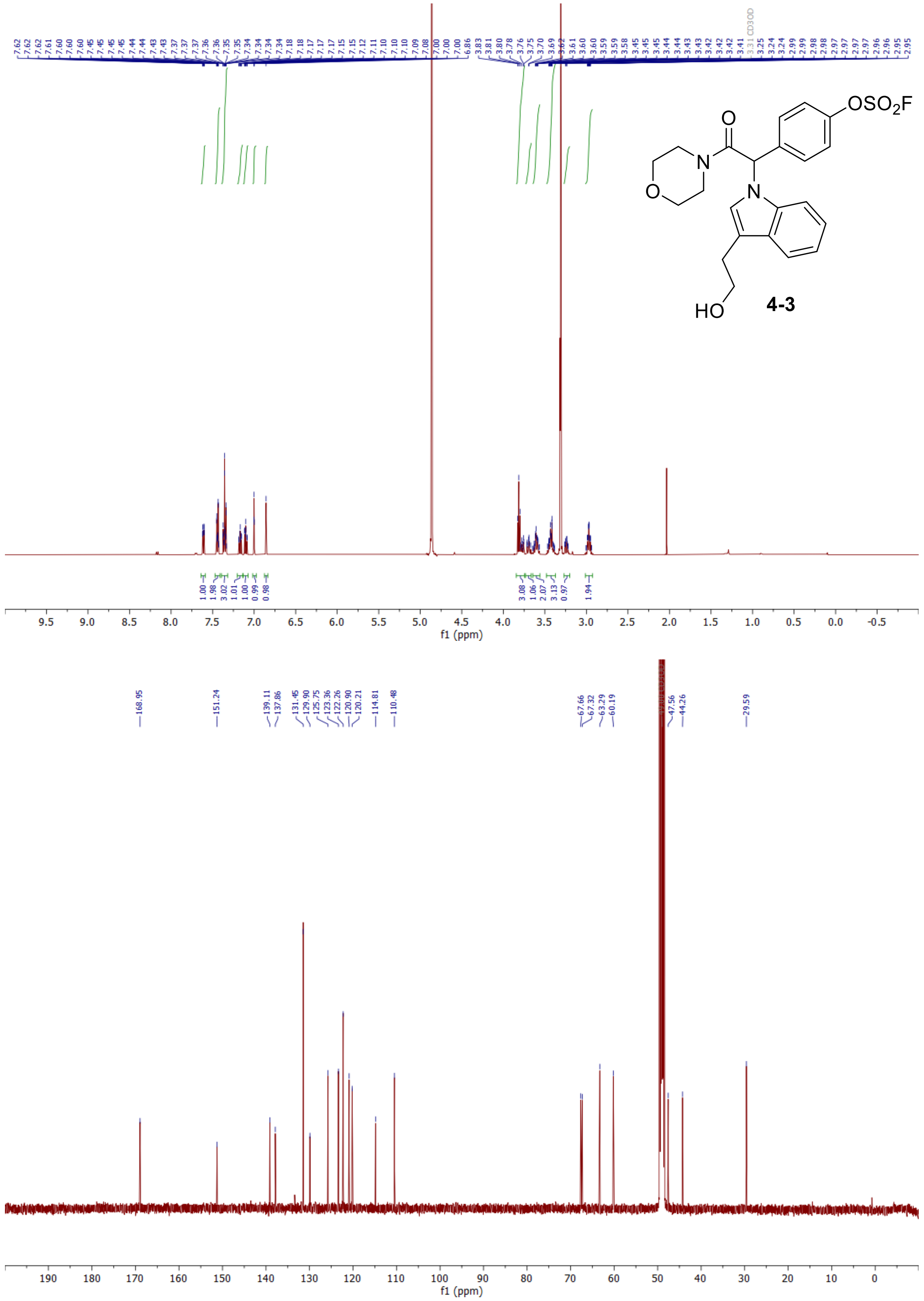

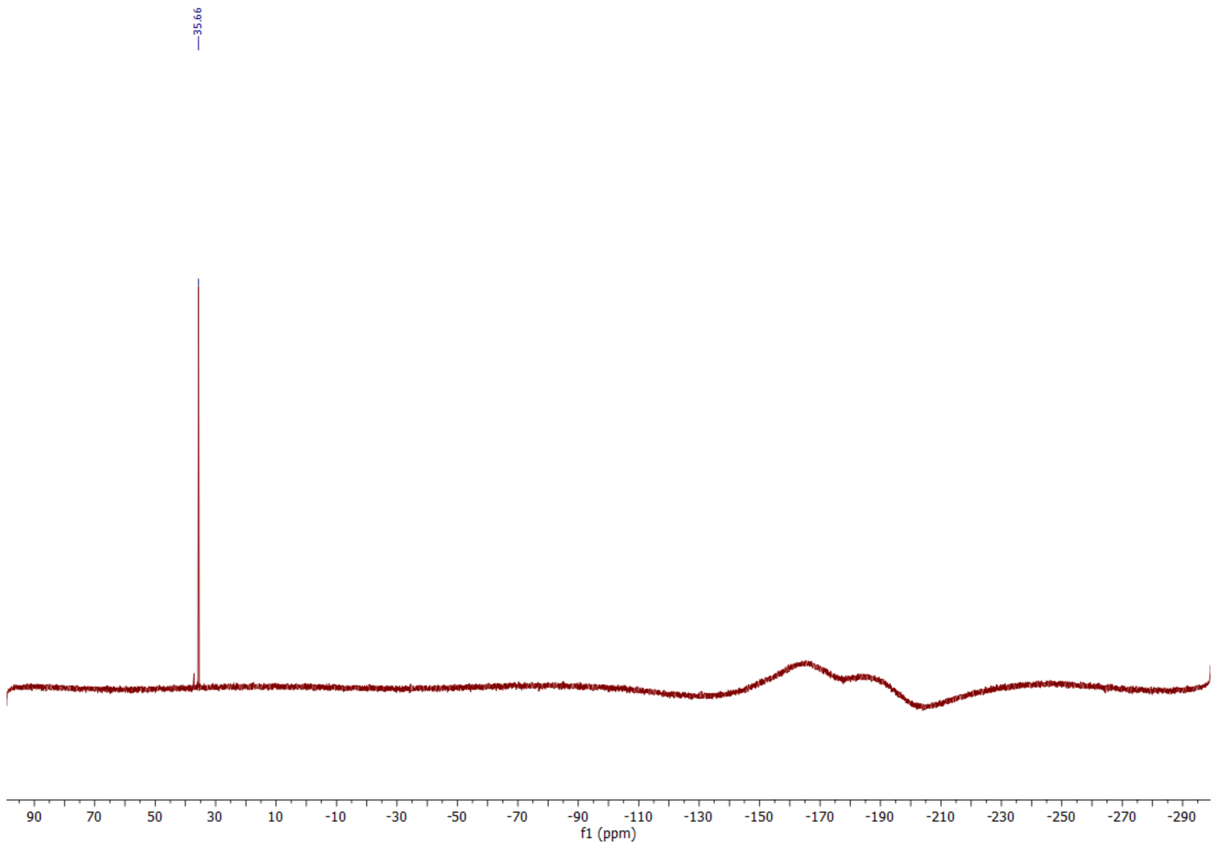
35.66
90
70
50
30
10
-10
-30
-50
-70
-90
-110
-130
-150
-170
-190
-210
-230
-250
-270
-290
f1 (ppm)

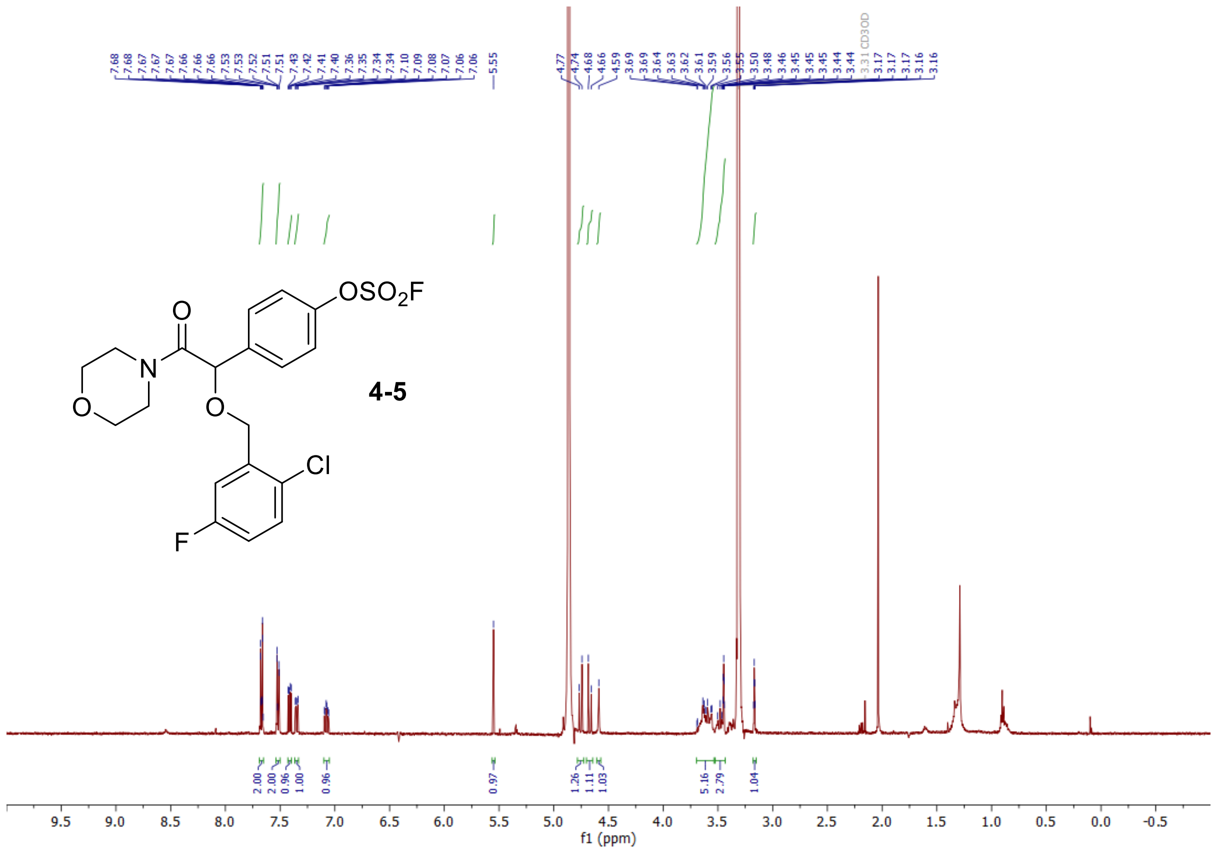
7.68
7.68
7.67
7.67
7.67
7.66
7.66
7.66
7.53
7.53
7.52
7.51
7.51
7.43
7.42
7.41
7.40
7.36
7.35
7.34
7.34
7.10
7.09
7.08
7.07
7.06
7.06
5.55
4.77
4.74
4.68
4.66
4.59
3.69
3.69
3.64
3.63
3.62
3.61
3.59
3.56
3.55
3.50
3.48
3.46
3.45
3.45
3.45
3.44
3.44
3.31 CD3OD
3.17
3.17
3.17
3.16
3.16
OSO2F
O
N
O
O
Cl
F
4-5
2.00
2.00
0.96
1.00
0.96
0.97
1.26
1.11
1.03
5.16
2.79
1.04
9.5 9.0 8.5 8.0 7.5 7.0 6.5 6.0 5.5 5.0 4.5 4.0 3.5 3.0 2.5 2.0 1.5 1.0 0.5 0.0 -0.5
f1 (ppm)

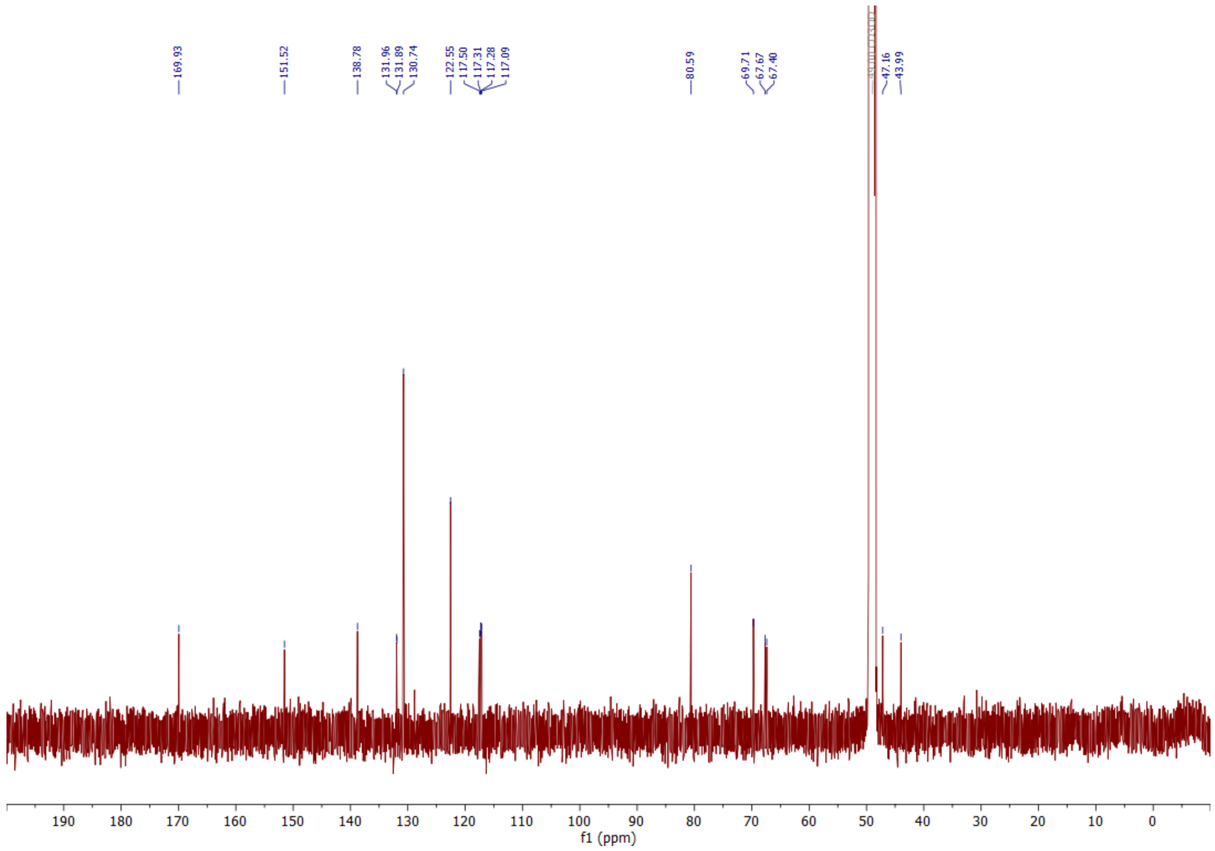
169.93
151.52
138.78
131.96
131.89
130.74
122.55
117.50
117.31
117.28
117.09
80.59
69.71
67.67
67.40
47.16
43.99
190 180 170 160 150 140 130 120 110 100 90 80 70 60 50 40 30 20 10 0
f1 (ppm)

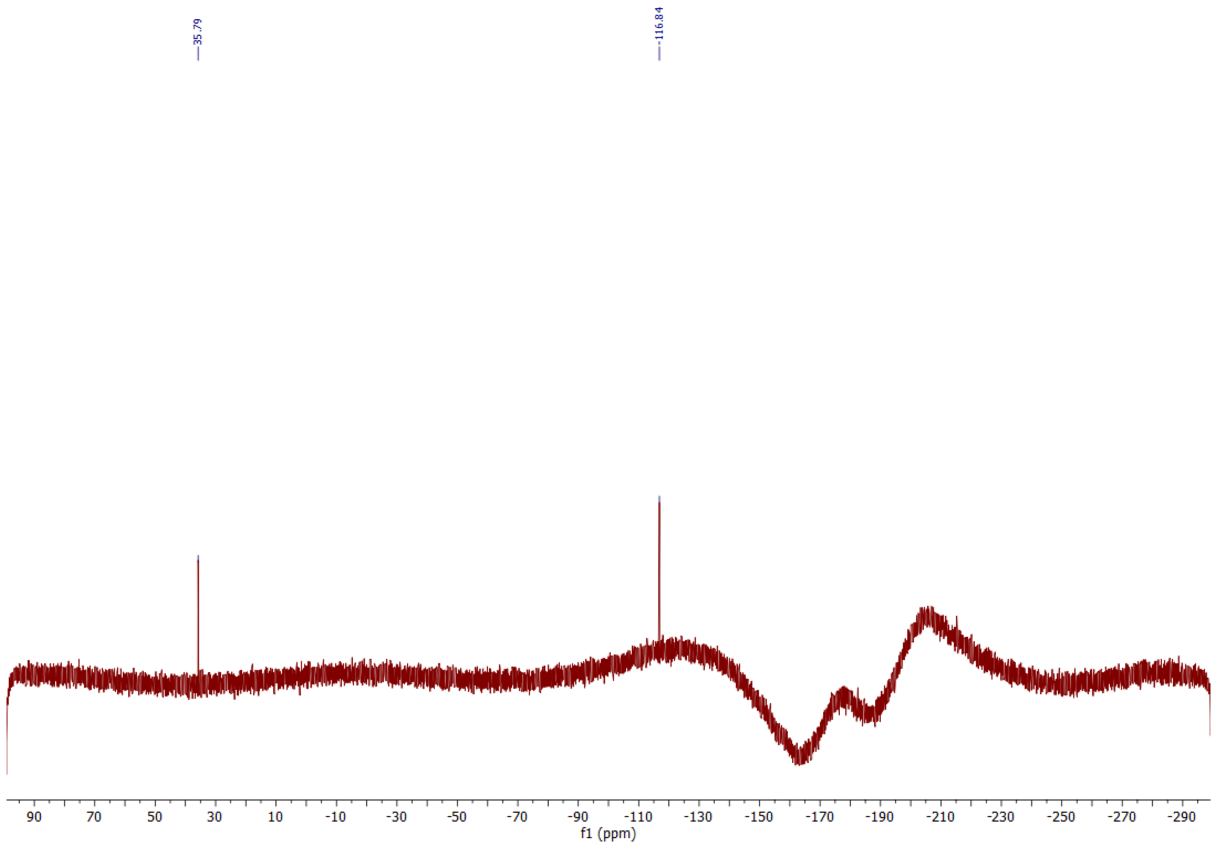
35.79
116.84
90
70
50
30
10
-10
-30
-50
-70
-90
-110
-130
-150
-170
-190
-210
-230
-250
-270
-290
f1 (ppm)

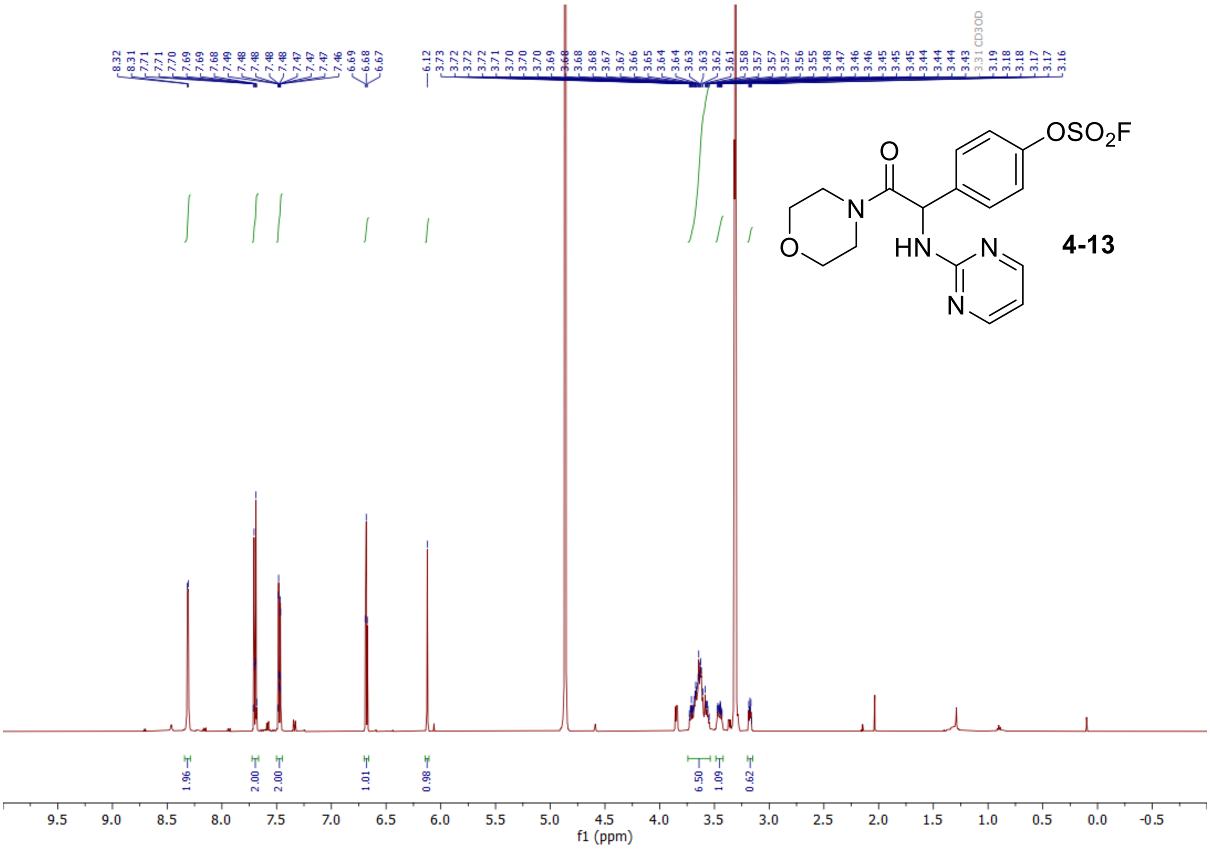
8.32 8.31 7.71 7.71 7.70 7.69 7.69 7.68 7.49 7.48 7.48 7.48 7.48 7.47 7.47 7.47 7.46 6.69 6.68 6.67
6.12 3.73 3.72 3.72 3.72 3.71 3.70 3.70 3.70 3.69 3.68 3.68 3.67 3.67 3.66 3.65 3.64 3.63 3.63 3.62 3.61 3.58 3.57 3.57 3.57 3.56 3.55 3.48 3.47 3.46 3.46 3.45 3.45 3.45 3.44 3.44 3.43 3.31 CD3OD 3.19 3.18 3.18 3.17 3.17 3.16
OSO$_2$F
O
N
O
HN
N
N
4-13
1.96 2.00 2.00 1.01 0.98 6.50 1.09 0.62
9.5 9.0 8.5 8.0 7.5 7.0 6.5 6.0 5.5 5.0 4.5 4.0 3.5 3.0 2.5 2.0 1.5 1.0 0.5 0.0 -0.5
f1 (ppm)

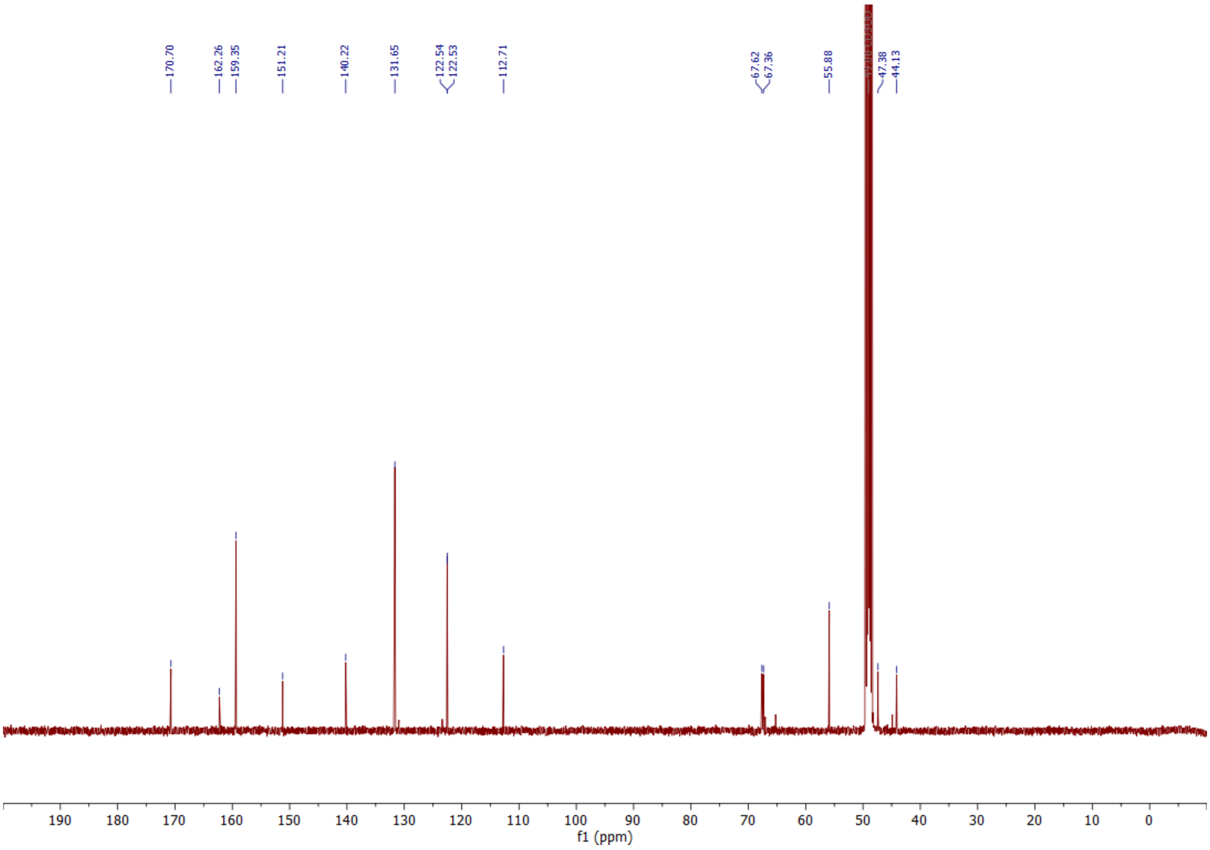
170.70 162.26 159.35 151.21 140.22 131.65 122.54 122.53 112.71
67.62 67.36 55.88 47.38 44.13
190 180 170 160 150 140 130 120 110 100 90 80 70 60 50 40 30 20 10 0
f1 (ppm)

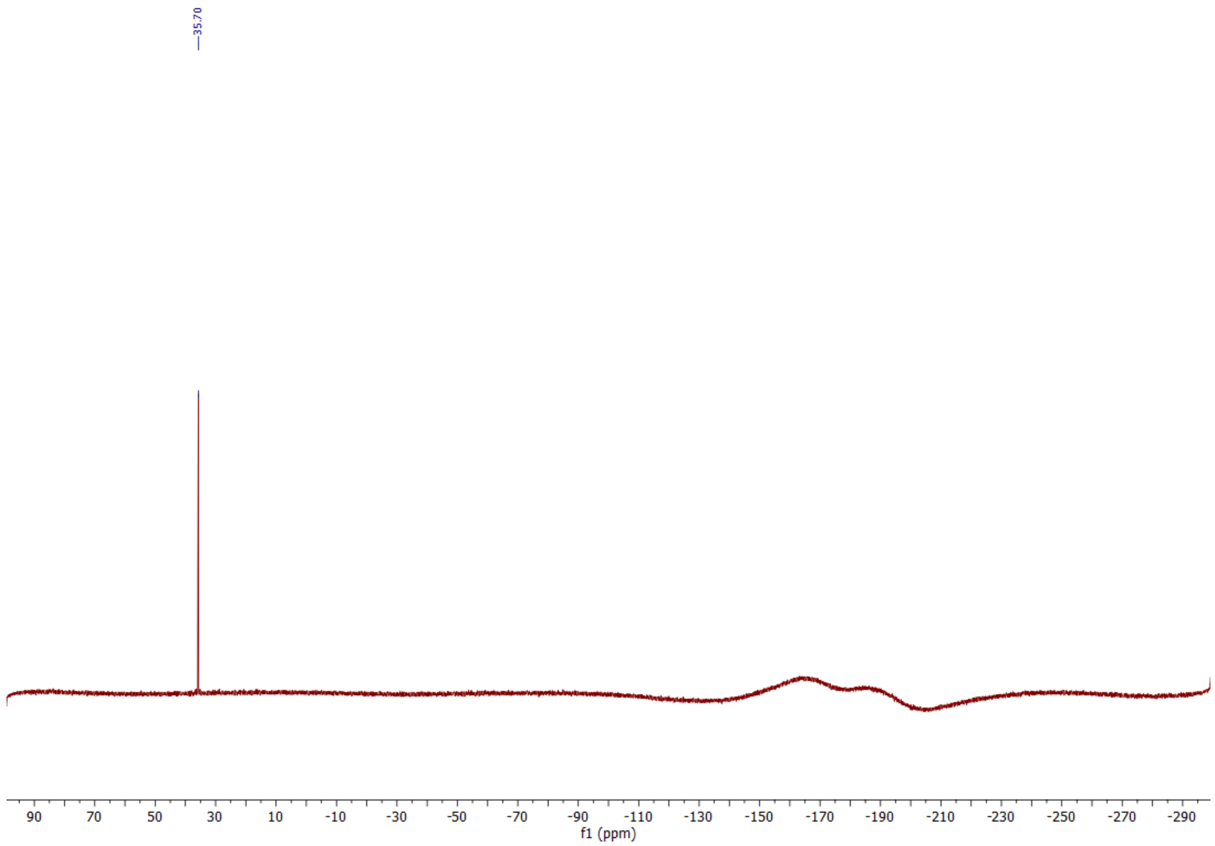

35.70
90 70 50 30 10 -10 -30 -50 -70 -90 -110 -130 -150 -170 -190 -210 -230 -250 -270 -290
f1 (ppm)

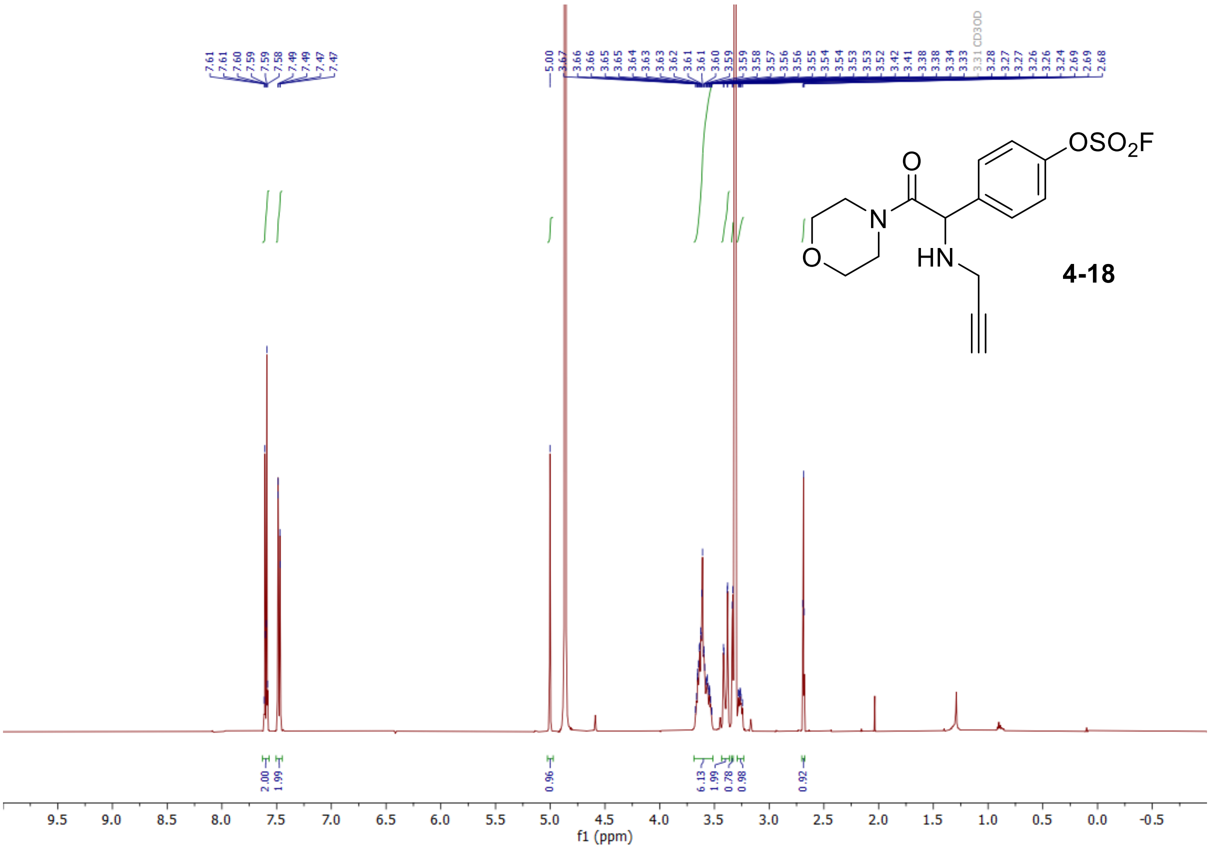

OSO2F
O
N
O
HN
4-18
f1 (ppm)

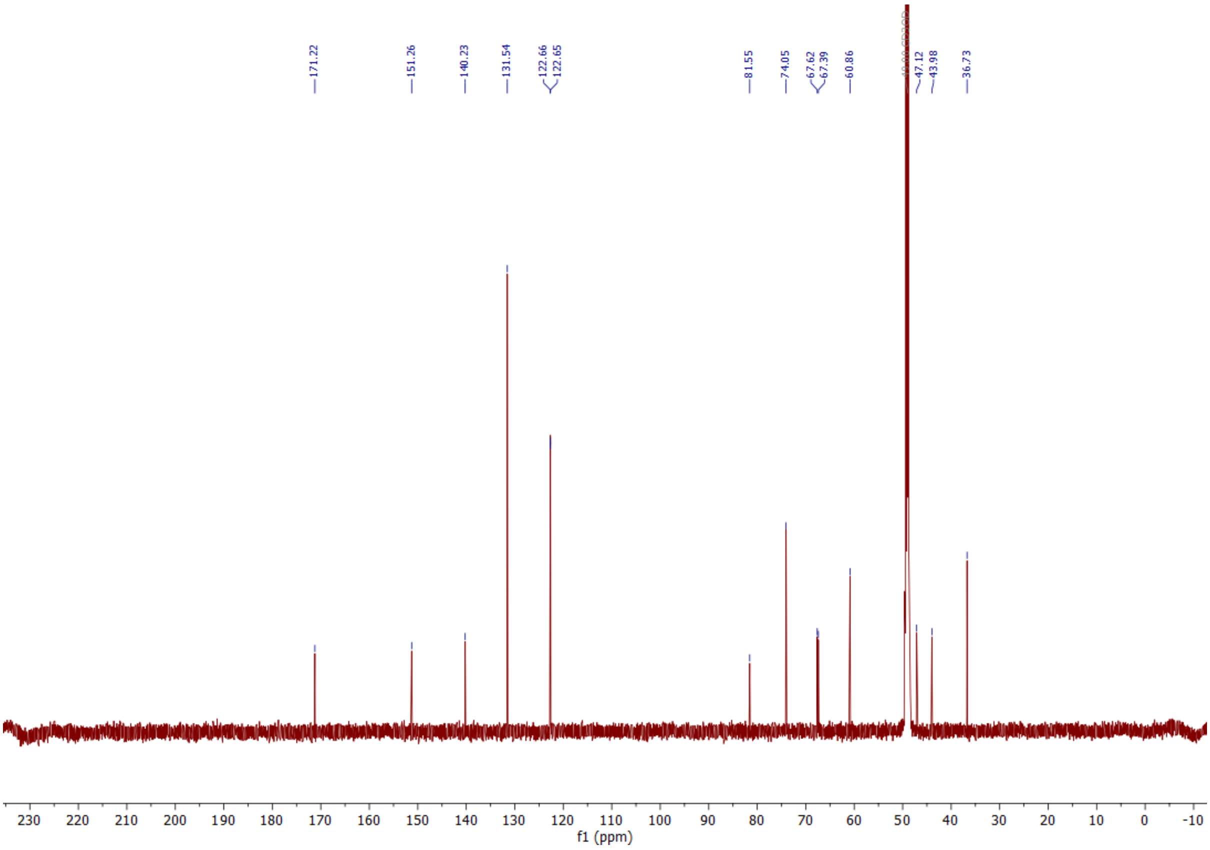

f1 (ppm)

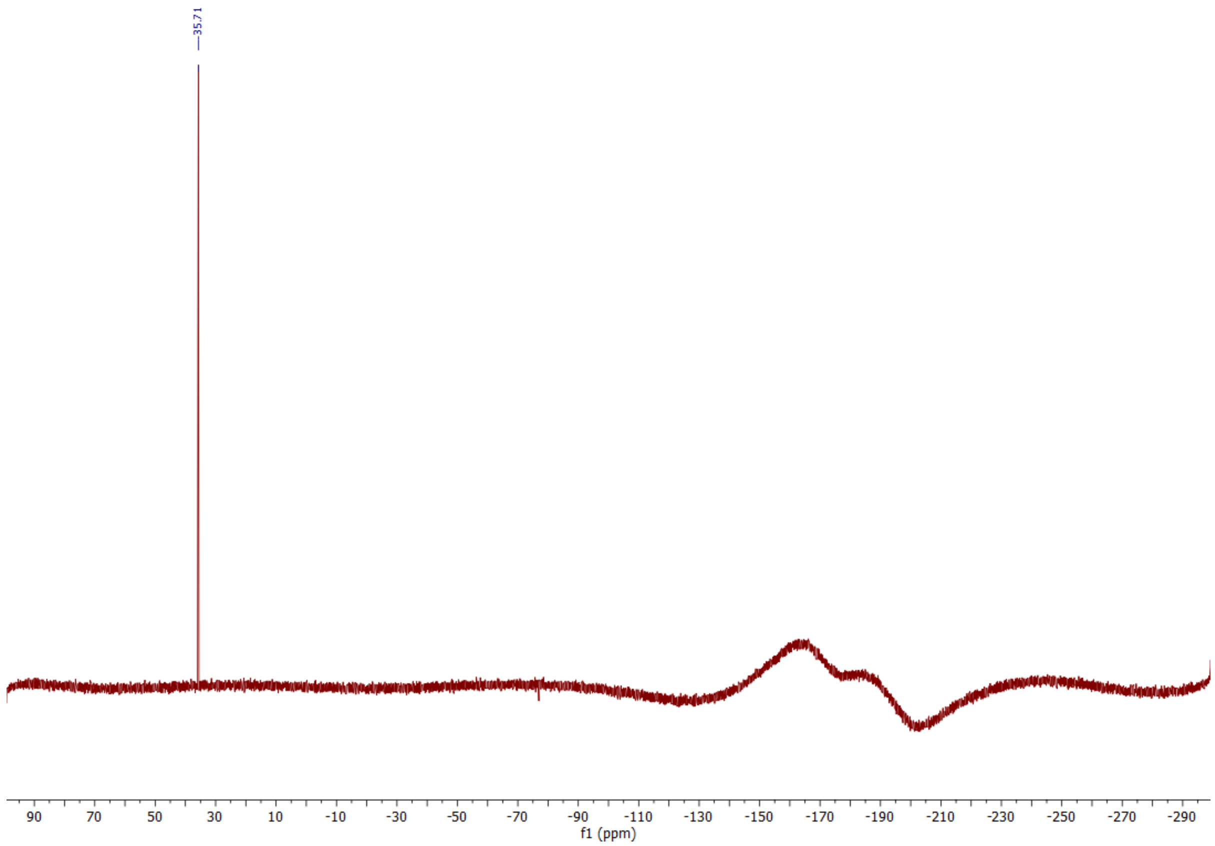

35.71
90
70
50
30
10
-10
-30
-50
-70
-90
-110
-130
-150
-170
-190
-210
-230
-250
-270
-290
f1 (ppm)

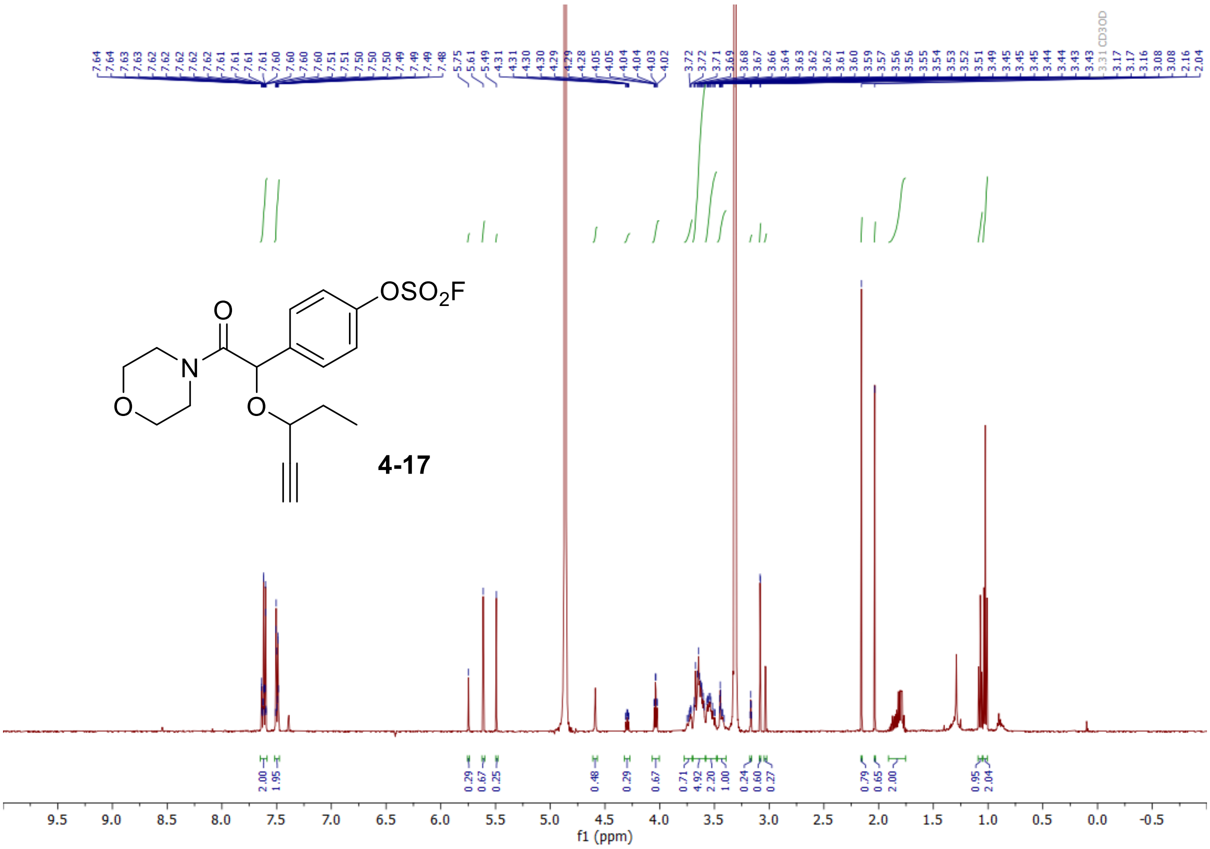

7.64
7.64
7.63
7.62
7.62
7.62
7.62
7.61
7.61
7.61
7.61
7.60
7.60
7.60
7.60
7.51
7.51
7.50
7.50
7.50
7.49
7.49
7.49
7.48
5.75
5.61
5.49
4.31
4.31
4.30
4.30
4.29
4.29
4.28
4.05
4.05
4.04
4.04
4.03
4.02
3.72
3.72
3.71
3.69
3.68
3.67
3.66
3.64
3.63
3.62
3.62
3.61
3.60
3.59
3.57
3.56
3.56
3.55
3.54
3.53
3.52
3.51
3.49
3.45
3.45
3.44
3.44
3.43
3.43
3.31 CD3OD
3.17
3.17
3.16
3.08
3.08
2.16
2.04
OSO2F
O
N
O
O
4-17
2.00
1.95
0.29
0.67
0.25
0.48
0.29
0.67
0.71
4.92
2.20
1.00
0.24
0.60
0.27
0.79
0.65
2.00
0.95
2.04
9.5 9.0 8.5 8.0 7.5 7.0 6.5 6.0 5.5 5.0 4.5 4.0 3.5 3.0 2.5 2.0 1.5 1.0 0.5 0.0 -0.5
f1 (ppm)

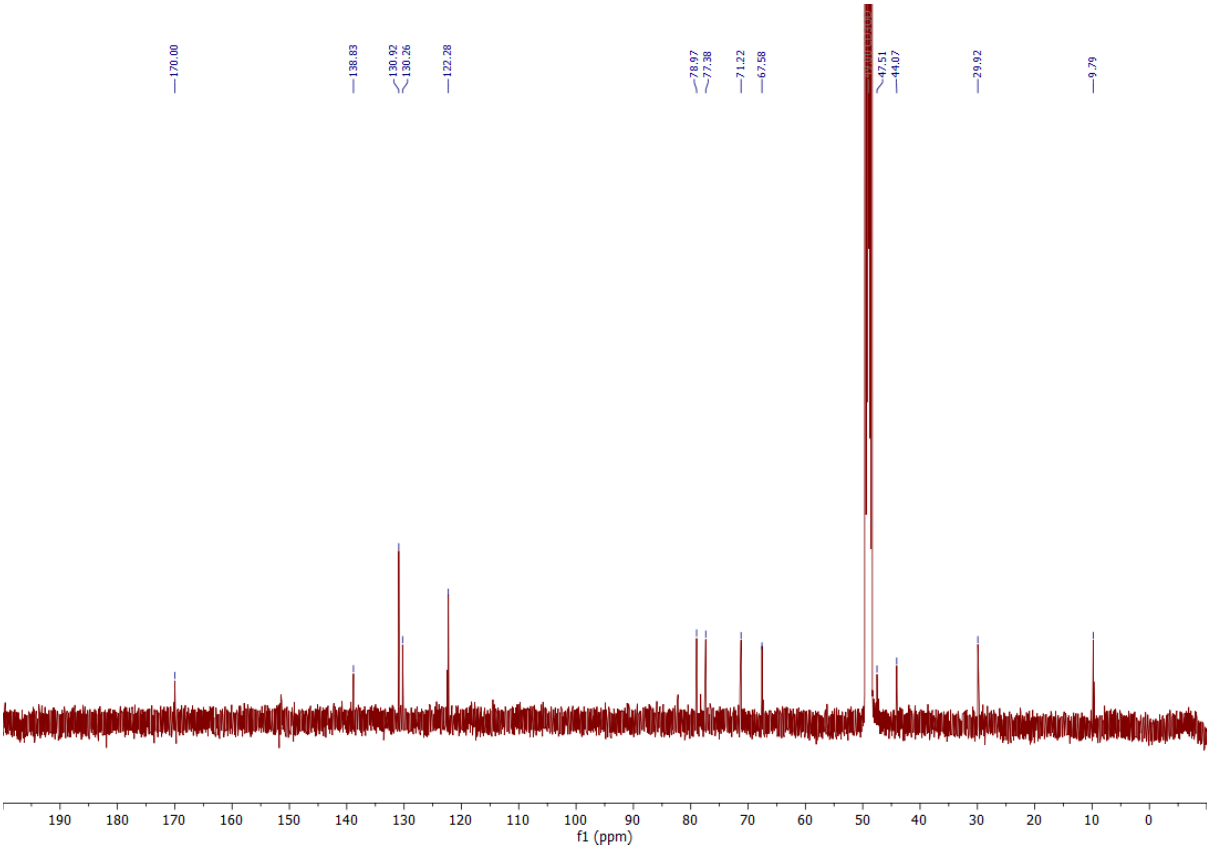

170.00
138.83
130.92
130.26
122.28
78.97
77.38
71.22
67.58
47.51
44.07
29.92
9.79
190 180 170 160 150 140 130 120 110 100 90 80 70 60 50 40 30 20 10 0
f1 (ppm)

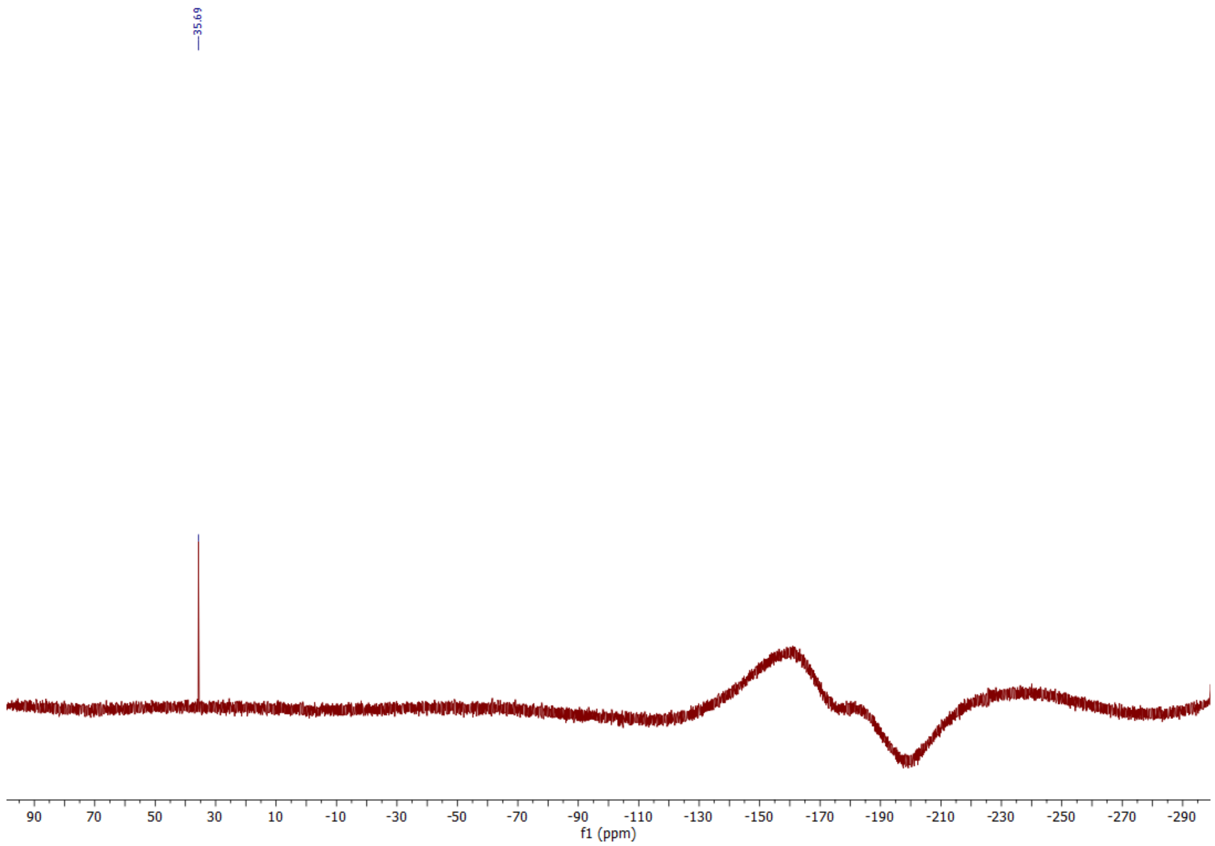

35.69
90
70
50
30
10
-10
-30
-50
-70
-90
-110
-130
-150
-170
-190
-210
-230
-250
-270
-290
f1 (ppm)

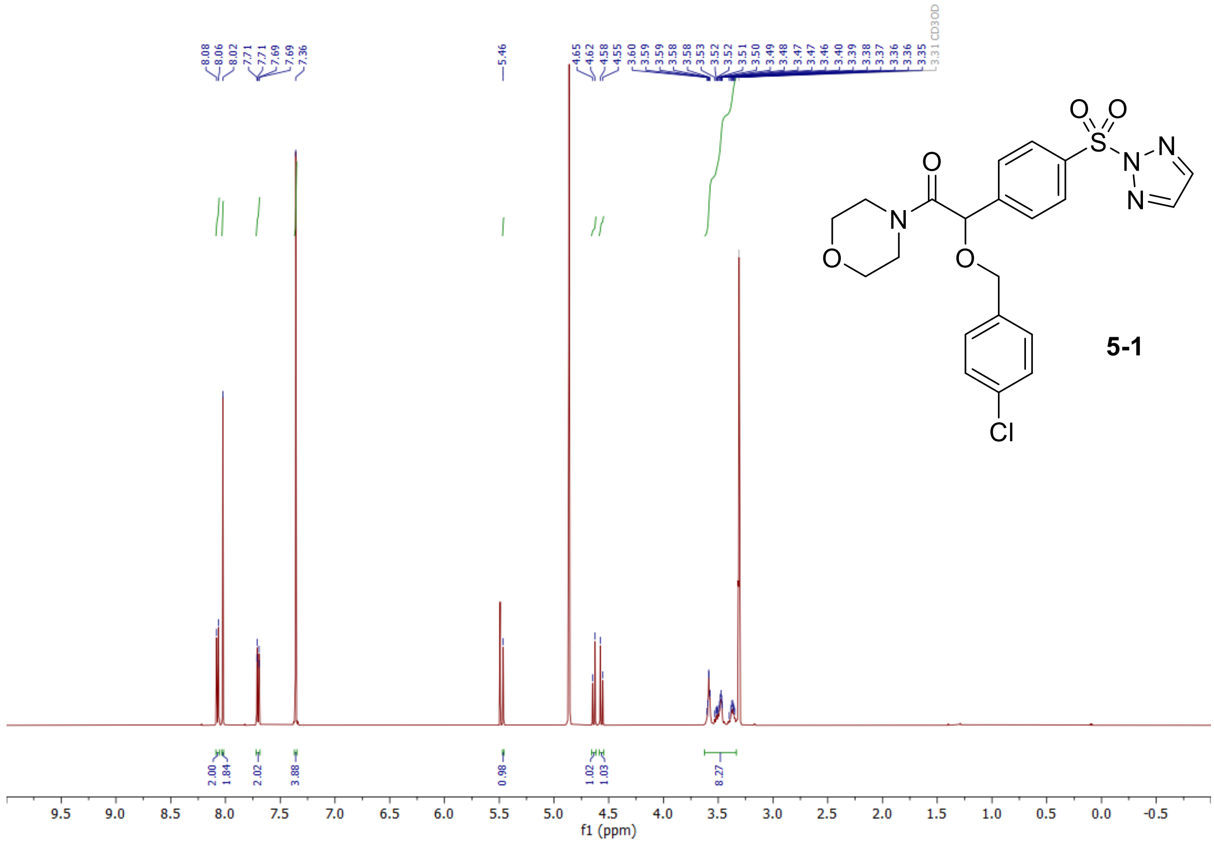
8.08
8.06
8.02
7.71
7.71
7.69
7.69
7.36
5.46
4.65
4.62
4.58
4.55
3.60
3.59
3.59
3.58
3.58
3.53
3.52
3.52
3.51
3.50
3.49
3.48
3.47
3.47
3.46
3.40
3.39
3.38
3.37
3.36
3.36
3.35
3.31 CD3OD
2.00
1.84
2.02
3.88
0.98
1.02
1.03
8.27
f1 (ppm)

5-1

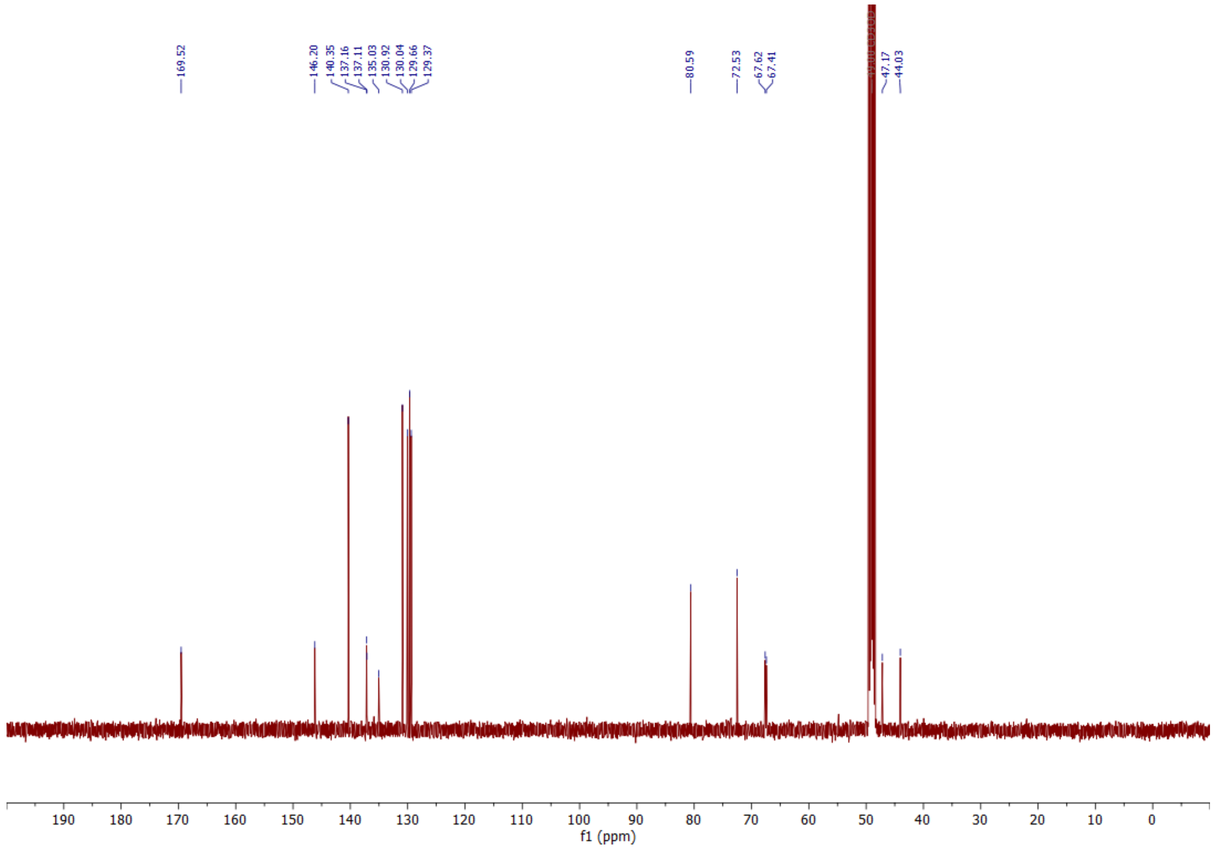
169.52
146.20
140.35
137.16
137.11
135.03
130.92
130.04
129.66
129.37
80.59
72.53
67.62
67.41
47.17
44.03
f1 (ppm)

References

1. (a) A. I. Green, F. Hobor, C. P. Tinworth, S. Warriner, A. J. Wilson and A. Nelson, *Chem. Eur. J.,* 2020, **26**, 10682-10689; (b) A. W. Squibb , M. R. Taylor B. L. Parnas, G. Williams, R. Girdler, P. Waghorn, A. G. Wright and F. Pullen F. S. *J. Chrom. A,* 2008, **1189**, 101-108; (c) S. D. Griggs, A.-S. Piticari, S. Liver, C. Arter, S. Sievers, S. P. Marsden and A. Nelson, *Chem. Commun.* 2025, **61**, 3528-3531.

2. D. Louvel, A. Chelagha, J. Rouillon, P. Payard, L. Khrouz, C. Monnereau and A. Tlili, *Chem. Eur. J.,* 2021, **27**, 8704-8708.

3. G. F. Zha, Q. Zheng, J. Leng, P. Wu, H. L. Qin and K. B. Sharpless, *Angew. Chem. Int. Ed.,* 2017, **56**, 4849-4852.

4. N. Döben, H. Yan, M. Kischkewitz, J. Mao and A. Studer, *Org. Lett.,* 2018, **20**, 7933–7936.
